# Water–glycan interactions drive the SARS-CoV-2 spike dynamics: insights into glycan-gate control and camouflage mechanisms†

**DOI:** 10.1039/d4sc04364b

**Published:** 2024-08-23

**Authors:** Marharyta Blazhynska, Louis Lagardère, Chengwen Liu, Olivier Adjoua, Pengyu Ren, Jean-Philip Piquemal

**Affiliations:** a Laboratoire de Chimie Théorique, Sorbonne Université, UMR 7616 CNRS 75005 Paris France louis.lagardere@sorbonne-universite.fr jean-philip.piquemal@sorbonne-universite.fr; b Department of Biomedical Engineering, The University of Texas at Austin Texas 78712 USA; c Qubit Pharmaceuticals 75014 Paris France

## Abstract

To develop therapeutic strategies against COVID-19, we introduce a high-resolution all-atom polarizable model capturing many-body effects of protein, glycan, solvent, and membrane components in SARS-CoV-2 spike protein open and closed states. Employing μs-long molecular dynamics simulations powered by high-performance cloud-computing and unsupervised density-driven adaptive sampling, we investigated the differences in bulk-solvent–glycan and protein–solvent–glycan interfaces between these states. We unraveled a sophisticated solvent–glycan polarization interaction network involving the N165/N343 glycan-gate patterns that provide structural support for the open state and identified key water molecules that could potentially be targeted to destabilize this configuration. In the closed state, the reduced solvent polarization diminishes the overall N165/N343 dipoles, yet internal interactions and a reorganized sugar coat stabilize this state. Despite variations, our glycan–solvent accessibility analysis reveals the glycan shield capability to conserve constant interactions with the solvent, effectively camouflaging the virus from immune detection in both states. The presented insights advance our comprehension of viral pathogenesis at an atomic level, offering potential to combat COVID-19.

## Abstract 2

We zoomed in on the SARS-CoV-2 interaction layers in open and closed states, revealing a protein–solvent–glycan polarization network supporting the open state. Besides, we showed that the glycan shield maintains viral camouflage in both states.

## Graphical abstract

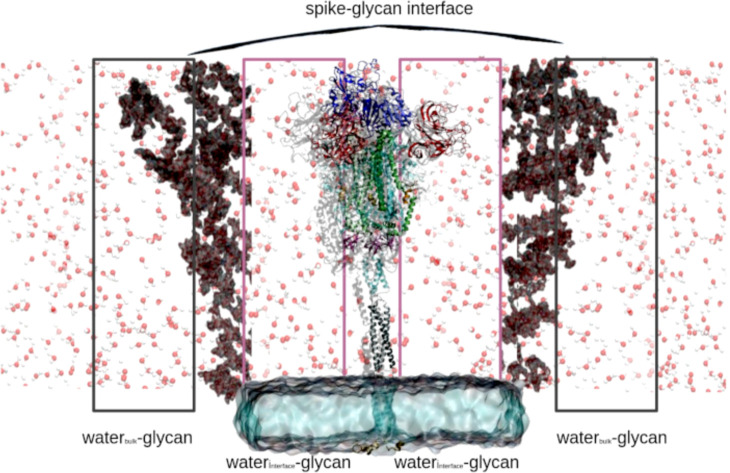

## 1 Introduction

The emergence of the COVID-19 pandemic^1^ has underscored the critical importance of comprehensively understanding the biochemical nature of the severe acute respiratory syndrome coronavirus 2 (SARS-CoV-2). Despite widespread collaborative efforts within the scientific community to develop treatments and vaccines,^2,3^ many aspects of the virus behavior remain poorly understood. Notably, elucidating the structural dynamics of the spike protein^4–7^ is of paramount importance for discerning its functional properties and identifying viable targets for the design of new therapeutic strategies.

**Fig. 1:**
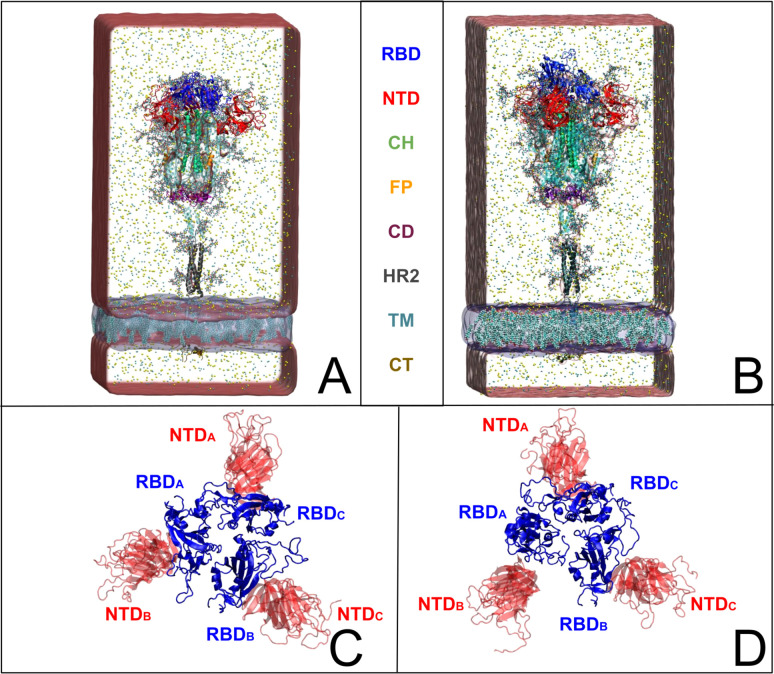
SARS-CoV-2 full spike protein structure shown in (A and C) closed and (B and D) open (one RBD_A_ up-conformation) states embedded in a water box and the viral membrane with cholesterol molecules (depicted as transparent red and blue rectangles, respectively). The receptor binding domain (RBD), the N-terminal domain (NTD), the central helix (CH), the fusion peptide (FP), the connector domain (CD), the heptad repeat 2 (HR2), the transmembrane domain (TM) and the cytoplasmic tail (CT) are represented in blue, red, green, orange, mauve, gray, cyan, and ochre, respectively. Top views (C and D) illustrate the structural reorganization of immune recognition sites on the RBD and NTD between closed (C) and open (D) states.

The coronavirus uses spike envelope glycoproteins (S-proteins comprising subunits S1 and S2) for receptor recognition in order to bind with the host, leading to membrane fusion, as well as to the entry into the host cell to initiate the viral infection.^5,6,8–10^ Structurally, the spike protein features a trimeric configuration, with each protomer composed of an N-terminal domain (NTD), a receptor-binding domain (RBD), a central helix (CH), a fusion peptide (FP), a connecting domain (CD), a heptad repeat (HR), a transmembrane domain (TM), and cytoplasmic tail (CT) (Fig. 1A and B). It is important to point out that the spike protein exhibits distinct prefusion conformations, namely the closed and open states.^4^ In the closed state, the spike protein is characterized by concealed RBDs (Fig. 1C), therefore limiting their interaction with the host-cell receptors. Conversely, the open state features one (Fig. 1D) or multiple exposed RBDs, accessible for the binding to host cells. The transition from the closed to the open state is triggered by the binding of the spike protein to host cell receptors, such as the angiotensin-converting enzyme 2 (ACE2), allowing the viral genome to initiate its viral replication and, therefore, the infection.^9,10^ Besides RBDs, glycans surround the SARS-CoV-2 spike protein and play a crucial role in modulating its structure and function, influencing various aspects of viral pathogenesis.^5,6,8,11–13^ While not being directly encoded in the viral genetic sequence,^14^ glycans act as a protective barrier, camouflaging the underlying protein structure from the host immune system, thus promoting the virus infection.^12,15,16^ Among experimental studies on elucidating the role of spike–glycan interactions,^9,10,12,13,15–24^ the work of Huang *et al.* demonstrated that enzymatic removal of glycans from the SARS-CoV-2 spike protein enhances immune responses and protection in animal models, further highlighting the significance of glycans in viral pathogenesis and vaccine development.^22^

Additionally, significant computational efforts have been dedicated to uncover intricate interactions between the spike protein and surrounding glycans.^5,6,8,25^ Thus, employing molecular dynamics (MD) simulations, Amaro *et al.*^8^ showed that during the RBD opening, the glycan present at N234 undergoes an inward rotation, filling the resultant void and contributing to the stabilization of the open configuration. Subsequently, their recent MD investigation, complemented by cryo-electron microscopy and biolayer interferometry experiments^25^ revealed that the glycans positioned at N343, N234, and N165 play a pivotal role in spike opening by sequentially interacting with multiple residues within the RBD, a mechanism referred to as “glycan gating”. It should be noted that in their studies, Amaro *et al.*^8,25^ utilized classical non-polarizable MD simulations tuning the conformational sampling to get insight into spike opening and offering valuable insights into spike-glycan dynamics. Despite being all-atom, the non-polarizable classical MD-based approaches may not fully capture some important mechanisms of charge redistribution in complex molecular systems; therefore, potential gaps in understanding the complete spectrum of spike protein behavior should be considered. Particularly, classical MD simulations may inadequately depict weak intermolecular interactions involving many-body effects,^26^ requiring the use of polarizable force fields^27^ for more nuanced exploration. In this context, the use of High-Performance Computing (HPC) *via* a distributed cloud infrastructure in conjunction with advanced sampling algorithms offers a promising avenue for the high-resolution description of intricate dynamics, especially for large and convoluted biological systems, such as the SARS-CoV-2 spike protein.

To address these questions, we leveraged the Tinker-HP simulation package^28^ employing its GPU-accelerated implementation,^29^ allowing us to conduct a series of all-atom MD simulations using the AMOEBA polarizable force field.^27,30^ Furthermore, our simulations rely on extensive density-driven adaptive conformational sampling^31^ of both open and closed configurations provided by Amaro *et al.*^8^ It is worth noting that our study marks the first realization of a parametrization of all the components of the full-length spike protein including glycans, water solvent, counter-ions, and a membrane bilayer with a polarizable force field, thus allowing us to access a more comprehensive and accurate representation of the spike complex interactions.

Our findings revealed subtle differences in protein–glycan interactions between the spike protein closed and open states. Notably, while the overall integrity of the glycan shield remained remarkably consistent in both forms, the mechanisms governing local interactions varied. In the open state, highly polarizable water molecules created dynamic hydrogen bonding that mediated specific protein–glycan interactions, particularly at residues N343 and N165, likely contributing to stabilization of the spike opening. In contrast, the closed state features loosely packed, low-polarization water molecules at the protein–glycan interface, suggesting that the stability of the protein structure in this state is maintained due to the inner reorganization of the protein and stabilization arising from the interacting glycans. The presented insights highlight the intricate balance between the SARS-CoV-2 state and the solvent interactions, indicating a sophisticated viral adaptation toward evading immune detection. Overall, our study provides a foundation for the development of targeted therapeutics aimed at disrupting viral infectiousness and enhancing host immune recognition, therefore offering promising avenues for fighting COVID-19 and other viral diseases.

## 2 Computational assays

### 2.1 Structural data and parametrization

The starting structure files of open and closed states (6VSB^8,32^ and 6VXX^8,33^ Protein Data Bank (PDB) ids, respectively) were taken from the Amaro Lab site,^34^ comprising the full-length spike protein (58 634 atoms) enveloped with 70 glycans (14 236 atoms), the membrane of POPC (1-palmitoyl-2-oleoyl-*sn*-glycero-3-phosphocholine), POPI (1-palmitoyl-2-oleoyl-*sn*-glycero-3-phosphoinositol), POPS (1-palmitoyl-2-oleoyl-*sn*-glycero-3-phosphatidylserine), POPE (1-palmitoyl-2-oleoyl-phosphatidylethanolamine), cholesterol (165 743 atoms in total), water molecules (1 451 508 atoms), and sodium (1647) and chloride ions (1366) creating a physiological concentration of 150 mM. The total rectangular box size was 204.7 × 199.4 × 408.5 Å^3^. The protein, water, and counterions were parameterized by using the AMOEBA polarizable force field by means of PDBXYZ utility available in the TINKER software package. More guidelines about the TINKER source code can be found in ref. 35. The AMOEBA parameters for the rest of the system (*i.e.*, lipids and glycans) were generated using Poltype2, a tool designed for automated parameterization of small molecules. The final parameters for the glycans used in our study have been included in the parameter files for both the open and closed states, available in ESI data files S3 and S4.† For detailed parameterization steps and outcomes specific to these glycans, refer to Data S2 in the ESI.† Although these glycan parameters are not yet part of the general AMOEBA force field, users can generate similar parameters using Poltype2 if needed. Additional information about Poltype2 is available in the public GitHub repository^36^ and in ref. 37 and 38.

### 2.2 Advanced molecular dynamics simulation details

To conduct an extensive unsupervised adaptive sampling simulation of open and closed states, we initiated our study with 10 ns conventional MD simulations employing the AMOEBA polarizable force field. Each system underwent an initial minimization process using a limited-memory Broyden–Fletcher–Goldfarb–Shanno (L-BFGS) algorithm until achieving a Root Mean Square (RMS) gradient of 1 kcal mol^−1^. Satisfied with the resultant models for both states, we proceeded with the extensive adaptive sampling.

For each MD simulation, we resorted to the BAOAB-RESPA1 integrator with an outer timestep of 10 fs,^39^ as implemented in the Tinker-HP software package,^39^ which is available for public usage in the GitHub repository^40^ or in the Tinker-HP web site.^41^ The integration scheme incorporated three time step sizes: a short time step of 1 fs, an intermediate time step of 4 fs and the largest one of 10 fs. The short time step was used to accurately capture the fastest motions within the system, while the intermediate time step addressed medium-frequency motions and interactions.

Our sampling technique, elaborately described in ref. 31, is a multi-iterative approach tailored for execution on large distributed computing resources such as supercomputers equipped with hundreds of GPU cards. Initially, we conducted nine independent 10 ns simulations, each starting from the same conformations for both states but with varied initial velocities. Subsequently, the resulting structures from these simulations were aligned with those obtained from the study by Casalino *et al.*^8^ Further fully automated structure selections were made by means of principal component analysis (PCA) based on their density in a low-dimensional space, prioritizing exploration of less-explored regions. Multiple 1.5 ns simulations were then launched from these initial structures, with each simulation state recorded at regular intervals. Cumulatively, 340 micro-iterations were performed to obtain a total simulation time of 510 ns for each state, allowing us to capture a significantly broader conformational landscape compared to traditional single trajectory sampling methods. It should be noted that for each iteration we computed a debiasing score to remove the statistical influence of the selection strategy described above, and therefore allowing us to compute accurate and unbiased observables. Table 1 provides detailed information about the adaptive sampling process, including the number of micro-iterations, the duration of each micro-iteration, and the associated speed of the simulations.

**Table 1:** Details of the adaptive sampling process

# of macro-iterations	# of micro-iterations	Length (ns/micro-iteration)	Speed (ns per day)
1	10	1.5	1.6
2	25		
3	25		
4	50		
5	50		
6	45		
7	45		
8	45		
9	45		

### 2.3 Adaptive sampling on the cloud

**Fig. 2:**
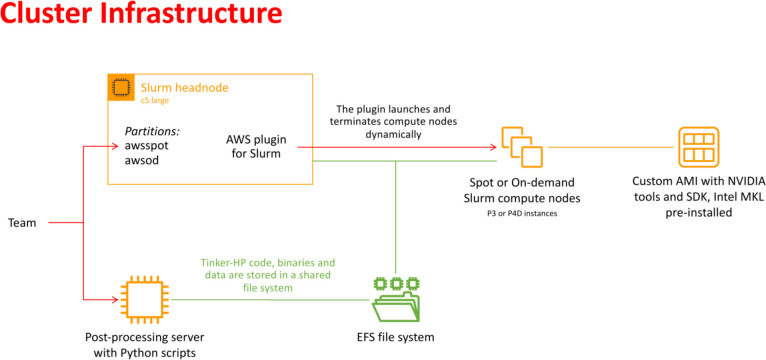
Schematic representation of the cloud-based virtual cluster used for this study, enabling the use of AWSSpot and AWSod (on demand) partitions and leveraging a shared elastic file system.

In recent years, cloud computing has emerged as an invaluable resource for handling computing demands, with providers such as Amazon Web Services (AWS), Google Cloud, and Microsoft Azure leading the way in offering flexible and secure services. Unlike traditional supercomputing clusters, where resources are allocated on an annual or bi-annual basis following rigorous scientific planning, cloud computing offers greater flexibility in resource allocation. Despite potentially higher associated costs, cloud computing provides researchers with an alternative that is well-suited to dynamic project requirements. With the support of AWS specialists, we successfully deployed a cloud-based supercomputer, utilizing Slurm for job submission management, as depicted in Fig. 2. The architecture of this supercomputer consisted of a Slurm head node based on a C5 large instance, with compute nodes utilizing spot or on-demand Nvidia GPUs (P3 or P4D instances). The P3 instances, configured with 4 V100 GPUs per node, enabled 51 micro-iterations to run in parallel, utilizing a total of 408 V100 GPUs simultaneously (*i.e.*, for both open and closed states). Although P4D instances equipped with 8 A100 GPUs per node were available, they were not employed for the AS. The setup also included customized Amazon Machine Images (AMIs) with essential pre-installed libraries. Additionally, a dedicated post-processing server equipped to execute Python-based scripts was included. Notably, all instances were interconnected through a shared Elastic File System (EFS). The distributed nature of the adaptive sampling strategy aligns seamlessly with cloud computing, as it operates without the need for synchronization during molecular dynamics trajectories. Such inherent adaptability renders the strategy independent of the specific computing resources it utilizes, making it well-suited for deployment within cloud computing environments.

## 3 Principal component analysis of the SARS-CoV-2 spike protein

**Fig. 3:**
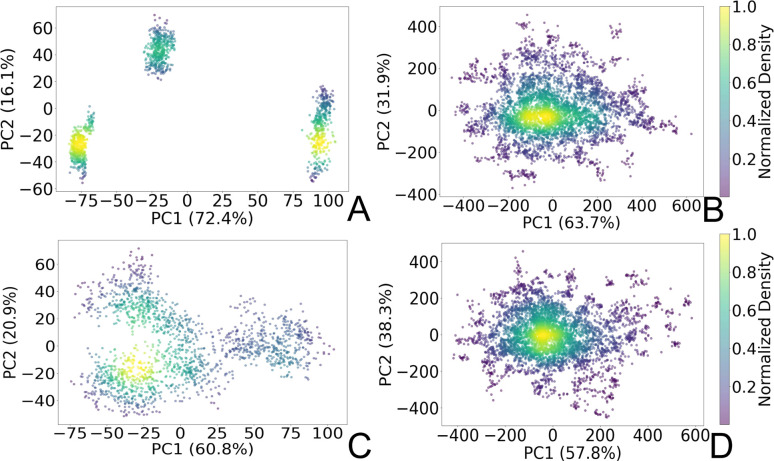
PCA plots representing two principal components (PC1 and PC2) of the SARS-CoV-2 spike C_α_ atoms in (A) open state reproduced from the Amaro laboratory enhanced sampling simulations from three replicas, (B) open state projected to Amaro laboratory PCA space (this work), (C) closed state reproduced from Amaro laboratory production simulations from three replicas, and (D) closed state projected to Amaro laboratory PCA space (this work). The points are colored based on the normalized probability densities obtained from kernel density estimation. The amount of variance of each PC is shown in %. Images are generated with the Matplotlib Python library.^42^

To gain insights into the structural dynamics governing the open and closed states of the SARS-CoV-2 spike protein, we employed the principal component analysis (PCA). Leveraging the solute-only trajectories shared by the Amaro laboratory^34^ and following their PCA calculation strategy discussed in detail in ref. 8, we replicated their PCA spaces for both open and closed conformations. Subsequently, we projected our extensively sampled simulations onto such spaces (Fig. 3). It should be noted that in the work presented by Casalino *et al.*,^8^ the Amaro laboratory implemented an enhanced sampling strategy solely for the open state, whereas exploration of the closed state was carried out through production simulations. Despite the limited conformational sampling in the latter, our analysis revealed an emergence of multiple intersecting clusters in their simulations, indicating the conformational heterogeneity within this state (Fig. 3C). Furthermore, our extensive sampling strategy applied to the closed state (Fig. 3D) enabled the delineation of distinct clusters corresponding to both highly visited regions of high probability densities (0.6–1.0), alongside capturing rare conformations of lower densities (0–0.4) within this state. In contrast, a significant difference in the PCA spaces was observed in the comparison with open-state simulations. While Amaro's data showed distinct, non-interacting clusters for this state (see Fig. 3A), our analysis depicted more intertwined clusters (Fig. 3B). Our analysis unveiled stable structural states of the protein (Fig. 3B and D), providing valuable insights into the spike dynamic behavior. It should be noted that similar improvements in configuration space sampling over conventional MD have been observed previously by our group (see ref. 31), thus underscoring the effectiveness of the implemented adaptive sampling strategy coupled with a polarizable force field.

## 4 Analysis of protein and glycan dynamics

**Fig. 4:**
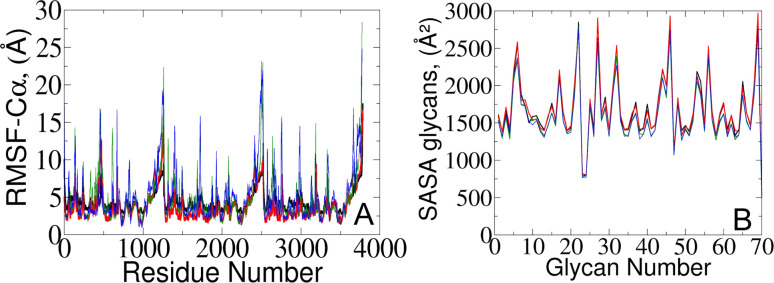
(A) RMSF of protein C_α_ atoms and (B) averaged SASA of 70 surrounding glycans obtained from the current study (represented in red and black) and from simulations reported by the Amaro lab^8^ (green and blue) for open and closed states, respectively.

To validate our models against those documented by Casalino *et al.*,^8^ we complemented our analysis by examining the root mean square fluctuations (RMSFs) of protein C_α_ atoms which elucidate the protein dynamic behavior in open and closed states (Fig. 4A). Our results revealed that the RMSF profiles derived from simulations utilizing the polarizable force field exhibit diminished fluctuations compared to the classical MD data.^8^ The observed variation likely arises from differences in how electrostatic interactions within the protein are captured, particularly in how they impact the arrangement and stability of charged groups near the C_α_ atoms. Indeed, classical force fields, which typically employ fixed charges assigned to atoms, tend to oversimplify these interactions since they do not consider many-body polarization effects. The application of the polarizable force field provides a route to include these effects *via* a more accurate representation.^27,30,43^

Additional hydrogen-bonding and salt-bridge analysis (see Fig. S1–S6 and Data S1 in the ESI†) provides further protein structural details. From our polarizable simulations, we identified only 27 common stable inner hydrogen bonds present in both the open and closed states. The closed state exhibited 77 unique stable hydrogen bonds and 4 unique salt bridges, while the open state had 45 unique stable hydrogen bonds and only 2 unique salt bridges. Such disparity indicates that the inner protein structure of the closed state is more extensively stabilized by interactions between its residues compared to the open state, suggesting a more rigid structure, which may contribute to its structural integrity until the fusion event unfolds. Conversely, the open state, with fewer stabilizing interactions, appears more plastic and adaptable, facilitating processes such as receptor binding and viral entry.

To gain further insights into potential triggers modulating SARS-CoV-2 protein conformational changes, we investigated the average solvent-accessible surface area (SASA) of the glycans covering the protein surface. The total SASA for each glycan was accumulated across all simulation frames, and the average value was determined by dividing the total SASA by the total number of frames (Fig. 4B). Remarkably, our analysis of surrounding glycans showed similar average SASA profiles in both classical and polarizable simulations. Regardless of the conformational state and the protein structural changes along the trajectories, the glycans maintained a stable level of exposure to the surrounding solvent molecules. Such stability suggests the formation of a consistent and robust protective barrier, effectively camouflaging^17–19,23^ the underlying protein structure from the external environment. Prior literature underscores the crucial role of glycan camouflage in CoV spike proteins (*i.e.*, SARS-CoV-2), impacting host attachment, immune responses, and virion.^11,16–19,44,45^ However, it was previously unknown that the glycan covering persists unchanged across different conformational states of the spike protein, suggesting a sophisticated strategy employed by the virus to limit accessibility to the spike and potentially escape detection by the immune system.

**Fig. 5:**
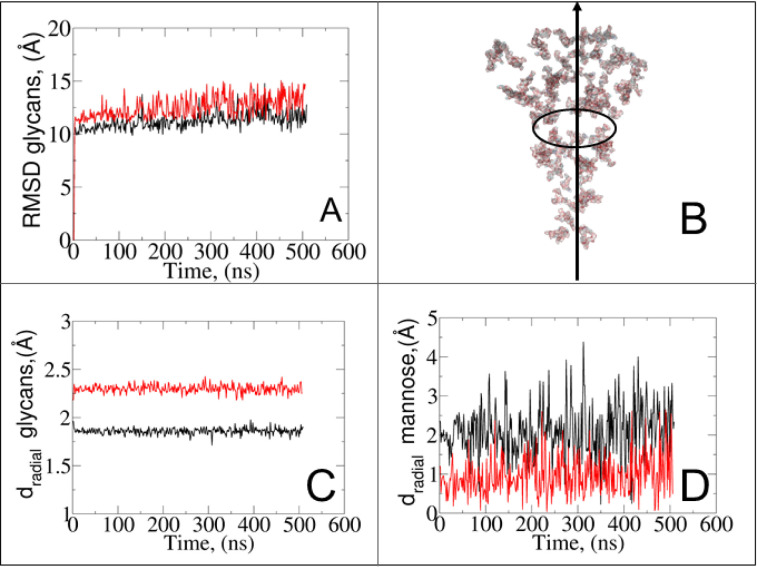
(A) RMSD of all surrounding spike glycans excluding hydrogen atoms. (B) Schematic representation of the radial distance of glycans from the *z*-axis of the protein, which is used for the graphs in panels (C) and (D). (C) Radial distances of all glycans relative to the protein. (D) Contribution of α-d-mannose residues in the total glycan radial distances. Red and black colors indicate the open and closed states, respectively.

It should be noted that despite similar glycan SASA profiles, the interaction patterns at the protein–glycan interface differ between the two states. To gain insights into glycan spatial dynamics around the spike protein, we conducted analyses of glycan root mean square deviation (RMSD) and their radial positioning relative to the protein central axis (Fig. 5A and B). The RMSD analysis revealed lower values for glycans in the closed state, indicating a more rigid and tighter packing around the protein, which likely enhances its structural integrity. Glycan radial distance analysis (Fig. 5C), along with protein–glycan dynamic cross-correlation (Fig. S7†) and relative distance evaluation (Fig. S9†), supported a closer association between glycans and the protein surface at immune recognition sites (*i.e.*, RBD and NTD) in the closed state.

Given the prevalence and role of α-d-mannose residues in viral attachment,^8,23,46–48^ we also evaluated their contribution to the glycan radial motion. In the closed state, the radial distances of these residues closely matched the total glycan radial distances (Fig. 5D), indicating their crucial role in the stabilization of the closed form. Conversely, in the open state, the smaller contribution of α-d-mannose residues (∼1 Å) to the total radial motion compared to that of all glycans (∼2.4 Å) suggests their lesser involvement in overall glycan dynamics. However, their closer radial arrangement in the open state may indicate their reorganization near the protein surface, potentially influencing viral infectivity, immune recognition, and evasion mechanisms. Our observation aligns with recent studies^49–59^ that explored lectin-based inhibitors targeting high-mannose residues on coronaviruses, suggesting a viable strategy for preventing viral entry and enhancing immune recognition.

## 5 Solvent interactions at the glycan–protein interface through radial distribution function analysis

**Fig. 6:**
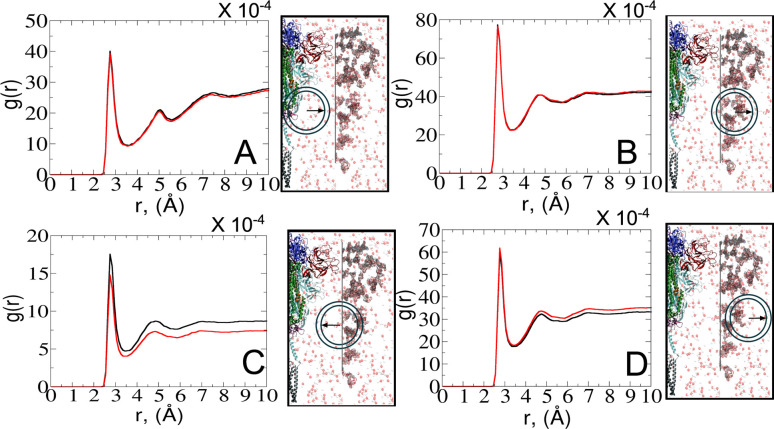
RDFs depict the interactions between water–protein and water–glycan oxygen atoms in the closed (black) and open (red) states. Each RDF plot is accompanied by a schematic representation on the right side of the graph, denoting interfaces from left to right: protein, interfacial solvent, glycan, and bulk solvent. The positions of the radial circles where the RDF calculations were performed are shown. (A) RDF between water and protein oxygen atoms. (B) RDF between water and glycan oxygen atoms. (C) RDF between water and glycan oxygen atoms adjacent to the protein interface. (D) RDF between water and glycan oxygen atoms closer to the bulk solvent.

To explore the dynamics at the glycan–protein interface, we investigated its interactions with the solvent using a radial distribution function (RDF) analysis. Initially, we computed two distinct RDFs: one between water and protein oxygen atoms, and another between water and glycan oxygen atoms. While the total intensity values of *g*(*r*) were small, our focus remained on qualitative aspects. Remarkably, the RDF profiles exhibited similarities between the open and closed states (Fig. 6A and B), mirroring the consistent glycan SASA patterns (Fig. 4B). We further refined the analysis by splitting the RDF between water and glycan oxygen atoms (Fig. 6B) into two parts: glycan oxygens adjacent to the protein interface (Fig. 6C) and those closer to the bulk solvent (Fig. 6D).

An interesting observation emerged from the RDF of water–glycan oxygen pairs oriented towards the protein interface: the closed state exhibited a more pronounced RDF compared to the open state, indicating a potentially more permissive arrangement of interfacial water molecules. Such a finding suggests that the closed conformation, characterized by a predominance of glycans near the protein surface (see Fig. S7–S9†), may create a distinct microenvironment that influences the dynamics of water molecules differently compared to the open state. Conversely, the denser packing of water molecules in the open state tends to show a tighter interaction of the solvent with the protein–glycan interface, impacting its stability and functionality. The observed water behavior is further compensated for by the local bulk solvent reorganization at the glycan interface (Fig. 6D).

## 6 Role of polarizable water in mediating protein–glycan interactions

The disparities in water behavior at the protein–glycan interface across distinct conformational states prompted an in-depth investigation into the potential presence of solvent polarization that influences their interactions. It is important to acknowledge that only a limited number of experimental studies have explored the polarizing effect of the environment on viral activity. While investigations into enveloped viruses like SARS-CoV-2, vaccinia,^60^ and influenza,^61–63^ have shed light on the role of polarization in facilitating viral fusion processes and mediating proton transport, capturing the polarizable nature of water molecules in experiments poses significant challenges. Water dipoles, which are pivotal for polarization effects, are small in magnitude and transient, rendering their detection difficult.^64–67^ Consequently, computational investigations into solvent polarizability become essential. Non-polarizable classical force fields struggle to accurately capture the dynamic behavior of water molecules,^27,68^ making the use of polarizable force fields crucial for accurately representing the behavior of water molecules and elucidating their role in viral processes.

**Fig. 7:**
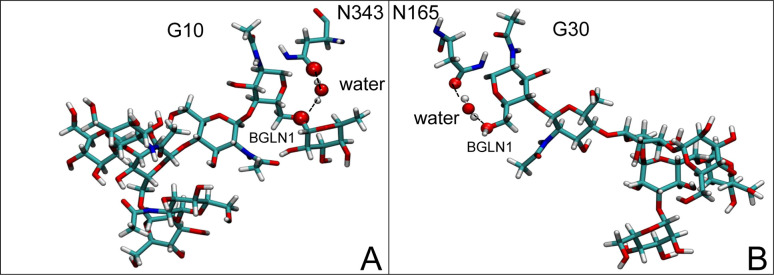
Polarizable water molecule creates bridging between (A) N343_RBD-A_ in the up-conformation and β-*N*-acetyl-d-glucosamine (BGLN1) of glycan G10, and (B) N165_NTD-B_ and BGLN1 of glycan G30 in the open state. In β-d-glucosamine, the oxygen atoms involved in this water-bridging interaction correspond to the ether oxygen (A) or hydroxyl group (B). In asparagine residues, the oxygen atoms involved in this water-bridging interaction are located in the carboxamide group. Interacting patterns are shown in van der Waals representation.

Initially, we conducted a thorough examination to identify water molecules exhibiting elevated dipole moments (*i.e.*, surpassing 2.8 D (ref. 69)) and a high occupancy (*i.e.*, exceeding 30%) proximal to the protein–glycan interface. Our analysis revealed the presence of highly polarizable water molecules at only two locations: adjacent to N343 (N343_RBD-A_) located in the RBD in the up-conformation with attached glycan G10, and adjacent to N165 (N165_NTD-B_) located in the NTD with glycan G30, exclusively in the open state (Fig. 7). Notably, these protein residues (*e.g.*, N165 (ref. 5, 8 and 20) and N343 (ref. 5, 20 and 21)) have been established as pivotal players in the spike opening. These findings are particularly significant in the context of the critical discovery of glycan gating by Sztain *et al.*^25^ While their work emphasized the importance of glycans at N343 in initiating and N234 and N165 in supporting the RBD opening, our study underscores the multifaceted nature of spike protein dynamics, integrating both glycan-mediated mechanisms and solvent interactions. Surprisingly, for N234, we observed only the reorganization of the attached high-mannose glycan G31, as depicted in contact maps comparing the open and closed states (see Fig. S9 in the ESI†), which contrasts with the solvent-mediated interactions involving N343_RBD-A_ and N165_NTD-B_ patterns.

**Fig. 8:**
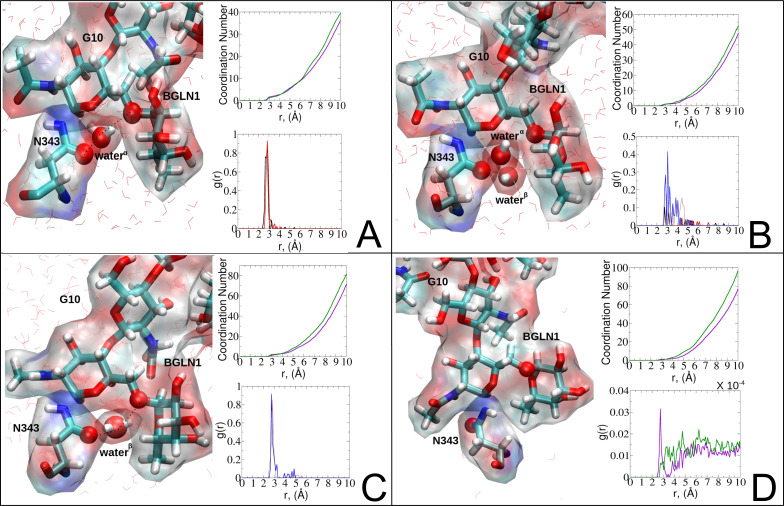
Phases of dynamic polarizable water molecule bridging formation between oxygen of N343 of RBD-A in the up-conformation and oxygen of BGLN1 of glycan G10 with the corresponding pair distribution functions (PDF), *g*(*r*), (black – N343(O)–water^α^(O), red – G10(O)–water^α^(O), brown – N343(O)–water^β^(O), and blue – G10(O)–water^β^ (O)), and coordination number corresponding to the interfacial water located between N343 and BGLN1 of G10 glycan residues (violet – N343(O)–water(O) and green – G10(O)–water(O)). (A) Bridging through a water molecule, (B) regrouping, (C) replacing, and (D) relaxation. Interacting patterns are shown in van der Waals representation. To visualize the molecular surfaces of the involved moieties we employed the MSIS (molecular surface) representation^70^ available *via* the Visual Molecular Dynamics (VMD) environment.^71^

Focusing on N343_RBD-A_ and N165_NTD-B_, we delved into the dynamics of their water-mediated interactions with glycans (*i.e.*, G10 and G30, respectively). Across all iterations of the open-state simulations, we noted clear variations in water behavior at the declared locations. Benefiting from extensive conformational sampling, we hypothesized that these dynamic water fluctuations could play an integral role in the mechanism of spike opening. We identified four key phases of water behavior, based on the average dipole moments and occupancies of the polarizable water molecules across all iterations of open-state simulations. These phases encompass bridging, clustering, replacement, and relaxation, as demonstrated on the interaction interfaces of N165-G30 and N343-G10, depicted in Fig. 8.

In the bridging phase, a specific water molecule (water^α^) actively forms hydrogen-bond interaction between the protein and glycan oxygen atoms (Fig. 8A). To be classified in this phase, water^α^ exhibits a dipole moment that exceeds 2.8 D and an occupancy of more than 30%. To strengthen our findings, we examined the total number of water molecules proximal to the protein–glycan atoms engaged in bridging, alongside analyzing the pair distribution functions (PDFs) of water^α^ with these atoms. The relatively high intensity of the PDF peak, nearing unity, indicates a significant concentration of water^α^ at a distance of approximately 2.8 Å from the N343 and G10 oxygen atoms, corresponding to the first coordination number, underscoring a strong correlation between the number of water molecules present at that distance and the peak intensity of the PDF.

Conversely, during the clustering phase, water^α^ experiences a reduction in dipole moment, falling below the threshold of 2.8 D, and a decrease in occupancy, dipping below 30%, as it conglomerates with other polarizable water molecules, thereby diminishing its individual contribution to interactions. Furthermore, within this phase, another water molecule, water^β^, exhibits a heightened dipole moment (more than 2.8 D), positioning to potentially replace water^α^ (Fig. 8B). Herein, the PDFs of water^α^ and water^β^ oxygen atoms appear notably noisy and less intensive compared to the bridging phase, indicating a competition between these water atoms.

During the replacement phase, water^α^ is finally substituted by a highly polarizable water molecule, water^β^ that exhibits a dipole moment exceeding 2.8 D and an occupancy of more than 30%, ensuring the continuity of interactions (Fig. 8C). During this phase, water^β^ undergoes an initial stabilization period, reflected in the PDF displaying peaks of low intensity of 4–5 Å.

Subsequently, upon achieving stability, water^β^ facilitates a bridging interaction between the protein and glycan residues, leading to a PDF closely resembling that observed during the bridging phase, characterized by high intensity.

Finally, the relaxation phase signifies the absence of significant water interactions at the interface (Fig. 8D). Notably, comprehensive RDFs for all water molecules proximal to the protein–glycan interface affirm the lack of pronounced interactions during this phase, with the closest interfacial water oxygen observed to be ∼3.5 Å. The loss of water mediation is attributed to the reorganization of the involved protein interacting sites, leading to changes in conformation and potentially disrupting the sites of interaction with water molecules.

To evaluate variations in the local environment and interactions experienced by the water molecules, we performed an in-depth analysis of the average dipole moments observed during each phase, along with their respective duration, across all iterations (Table 2). To compare, we also estimated the average dipole moments relative to the same group of oxygen atoms in the closed state, where no water-mediated interactions were observed. Additionally, we conducted a distribution analysis of the dipole moments for both the open and closed states separately (see Fig. S11–S14 in the ESI†). The water molecules involved in bridging interactions demonstrated higher polarity compared to other interfacial water molecules located in the proximity, thereby enhancing the stability of these interactions. As it can be noticed from Table 2, substantial variances are observed in the dipole moments of protein residues across different conformational states, indicating significant conformational changes occurring throughout the simulations, reflective of dynamic structural transitions within the protein.

Comparison of average dipole moments and duration of water-mediated interactions in open and closed states for N343_RBD-A_ and N165_NTD-B_ residues^a^

**Table:** 

Phases	N343_RBD-A_ open state					
	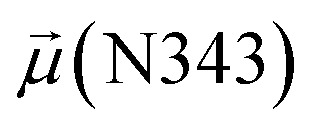 , D^a^	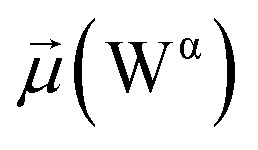 , D	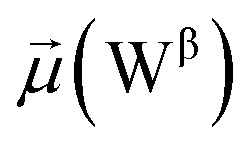 , D	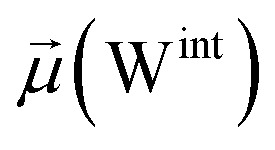 , D^b^	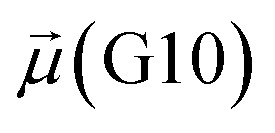 , D^c^	Duration, %
Bridging	615.4 ± 8.9	2.9 ± 0.1	—	2.7 ± 0.0	49.2 ± 0.0	17
Clustering	620.0 ± 16.5	2.7 ± 0.1	2.8 ± 0.1	2.7 ± 0.0	49.5 ± 1.4	19
Replacement	616.6 ± 13.9	—	2.9 ± 0.1	2.7 ± 0.0	49.4 ± 1.2	55
Relaxation	616.3 ± 8.9	—	—	2.7 ± 0.0	49.0 ± 0.9	10

a ^a^ and ^d^ correspond to the averaged atomic dipole moment in Debye of the oxygen atom of N343 and N165 residues, respectively, involved in hydrogen bonding with a polarizable water molecule, ^b^ refers to the interfacial water located at the proximity of 5 Å to the defined oxygen atoms of protein and glycan residues, and ^c^ and ^e^ correspond to the averaged atomic dipole moment.

**Table:** 

N343_RBD-A_ closed state					
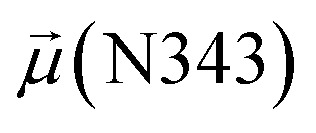 , D	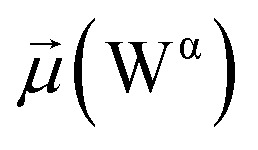 , D	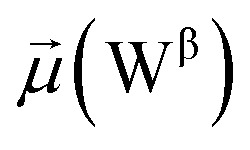 , D	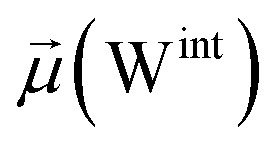 , D	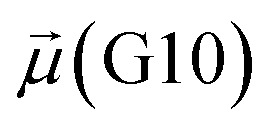 , D	Duration, %
568.0 ± 7.3	—	—	2.7 ± 0.1	46.6 ± 0.7	100

**Table:** 

Phases	N165_NTD-B_ open state					
	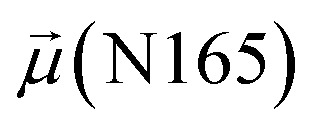 , D^d^	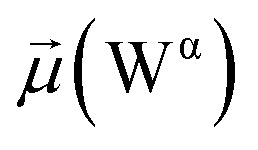 , D	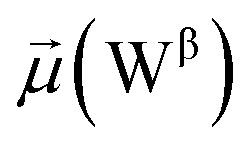 , D	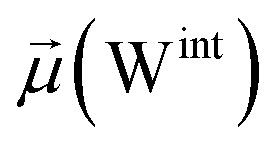 , D	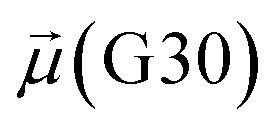 , D^e^	Duration, %
Bridging	651.0 ± 5.2	2.9 ± 0.1	—	2.8 ± 0.1	52.3 ± 0.4	7
Clustering	650.2 ± 8.6	2.7 ± 0.1	2.9 ± 0.1	2.8 ± 0.1	52.3 ± 0.6	8
Replacement	654.5 ± 9.3	—	2.9 ± 0.1	2.8 ± 0.1	52.6 ± 0.7	17
Relaxation	650.6 ± 8.3	—	—	2.7 ± 0.0	52.4 ± 0.6	68

**Table:** 

N165_NTD-B_ closed state					
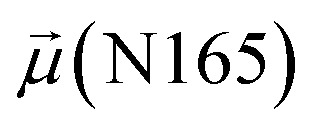 , D	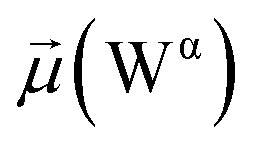 , D	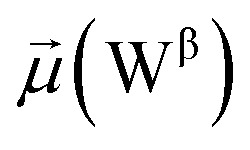 , D	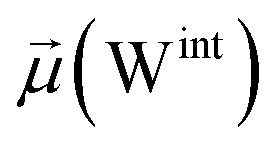 , D	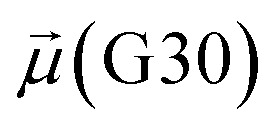 , D	Duration, %
563.9 ± 10.5	—	—	2.7 ± 0.1	45.1 ± 0.8	100

Besides, a net contrast arises from the comparison between the absolute values of the dipole moments of the oxygen of the carboxamide group of glycan-gating residues N343 and N165 in the open state, which could be attributed to the differences in their local environments at the protein–solvent–glycan interface and functional roles within the protein structure. Specifically, N343 experiences a more pronounced interaction with surrounding water molecules, which is also reflected in the cumulative duration of the bridging and replacement phases (exceeding 70%), leading to higher dipole moments compared to that of N165. In contrast, the absolute values of dipole moments for both N343 and N165 oxygen atoms in the closed state remain similar, suggesting that these residues may experience comparable degrees of involvement in stabilizing the protein structure in this particular state. Moreover, in the case of G30, the oxygen atom involved in water bridging is located in the hydroxyl group. The absence of high solvent polarizing effects in the closed state may result in fewer interactions contributing to the overall dipole moment, consequently yielding a lower value compared to the open state where such interactions are more prevalent. It should be noted that the dipole moments related to the oxygen atoms of glycans G10 and G30 exhibit relatively low variances, underscoring the consistent role of glycans in shielding protein interfaces in both states, contributing to the overall stability and function of the spike.

## 7 Conclusion

In this study, we performed high performance molecular dynamics simulations of the SARS-CoV-2 spike protein (1 693 134 atom system) using the AMOEBA polarizable force field coupled to extensive density-driven conformational sampling. Aggregating 1.2 μs of simulation data, we extensively explored the structural dynamics of the open and closed prefusion states. Our analysis reveals that, regardless of the spike protein conformational state, the glycan shield remains stable. This stability, despite the inherent variability of glycans—both across species and within individual cells—suggests a sophisticated strategy employed by the virus, tending to indicate the mechanism of viral adaptiveness at evading immune detection, enabling its persistence and spread within the host.

Contrary to the uniform glycan behavior, distinct structural properties were observed in the spike protein. The closed state maintained greater stability through robust hydrogen bonds and salt bridges, along with a tighter association between α-d-mannose-rich glycans and key immune recognition sites. In contrast, the open state experienced a reorganization of the glycans on the protein surface, hinting at their potential role in modulating viral infectivity.

Since the virus may fine-tune its immune evasion strategies, we further zoomed into how the solvent dynamics influences protein–glycan interactions. A detailed examination of the interfacial water reorganization at the protein–glycan interface showed a more dispersed arrangement of water molecules around glycans in the closed state, compared to the open one. Based on these differences, we further investigated how the polarizable nature of water molecules influences the stability and dynamics of protein–glycan interactions. We uncovered that highly polarizable interfacial water, exclusively present in the open state, plays a pivotal role in mediating interactions at glycan-gating patterns between N343 and N165 protein residues and their corresponding glycans. Additionally, the identification of distinct phases in open-state trajectories, namely bridging, clustering, replacement, and relaxation, elucidates water-mediated mechanisms at the protein–glycan interface. In contrast, the stability of the closed state may primarily result from internal protein and glycan interface reorganization rather than solvent-driven influences.

Relying on a robust computational approach, our study underscores the adaptability of the SARS-CoV-2 spike protein and the crucial role of the glycan shield in viral survival. Disrupting this shield could enhance immune detection, offering new therapeutic avenues. Additionally, understanding solvent dynamics provides a deeper insight into the spike's opening mechanism, presenting opportunities to prevent viral entry by targeting the water-mediated interactions at the glycan-gating sites that could potentially destabilize the open state and inhibit ACE2 binding.

## Data availability

All the data used in the analyses are available in the paper and/or the ESI.† Further information and requests should be directed to and will be fulfilled by the lead contact, JPP (jean-philip.piquemal@sorbonne-universite.fr).

## Author contributions

Conceptualization: MB, LL, and JPP. Methodology: OA, CL, LL, PR, and JPP. Investigation: MB and LL. Visualization: MB. Supervision: LL and JPP. Writing—original draft: MB. Writing—review & editing: LL, CL, PR, and JPP.

## Conflicts of interest

There are no conflicts to declare.

## Supplementary Material

SC-OLF-D4SC04364B-s001

SC-OLF-D4SC04364B-s002

SC-OLF-D4SC04364B-s003

SC-OLF-D4SC04364B-s004

SC-OLF-D4SC04364B-s005

SC-OLF-D4SC04364B-s006

## References

[cit1] McGowan V. J., Bambra C. (2022). Lancet Public Health.

[cit2] Yuan Y., Jiao B., Qu L., Yang D., Liu R. (2023). Front. Immunol..

[cit3] Ng T. I., Correia I., Seagal J., DeGoey D. A., Schrimpf M. R., Hardee D. J., Noey E. L., Kati W. M. (2022). Viruses.

[cit4] Cai Y., Zhang J., Xiao T., Peng H., Sterling S. M., Walsh J., Richard M., Rawson S., Rits-Volloch S., Chen B. (2020). Science.

[cit5] Pang Y. T., Acharya A., Lynch D. L., Pavlova A., Gumbart J. C. (2022). Commun. Biol..

[cit6] Zimmerman M. I., Porter J. R., Ward M. D., Singh S., Vithani N., Meller A., Mallimadugula U. L., Kuhn C. E., Borowsky J. H., Wiewiora R. P., Hurley M. F. D., Harbison A. M., Fogarty C. A., Coffland J. E., Fadda E., Voelz V. A., Chodera J. D., Bowman G. R. (2021). Nat. Chem..

[cit7] Wieczór M., Genna V., Aranda J., Badia R. M., Gelpí J. L., Gapsys V., de Groot B. L., Lindahl E., Municoy M., Hospital A., Orozco M. (2023). Wiley Interdiscip. Rev. Comput. Mol. Sci..

[cit8] Casalino L., Gaieb Z., Goldsmith J. A., Hjorth C. K., Dommer A. C., Harbison A. M., Fogarty C. A., Barros E. P., Taylor B. C., McLellan J. S., Fadda E., Amaro R. E. (2020). ACS Cent. Sci..

[cit9] Berger I., Schaffitzel C. (2020). Cell Res..

[cit10] Lu M., Uchil P. D., Li W., Zheng D., Terry D. S., Gorman J., Shi W., Zhang B., Zhou T., Ding S., Gasser R., Prévost J., Beaudoin-Bussières G., Anand S., Laumaea A., Grover J. R., Liu L., Ho D. D., Mascola J. R., Finzi A., Mothes W. (2020). Cell Host Microbe.

[cit11] Walls A. C., Tortorici M. A., Frenz B. (2016). et al.. Nat. Struct. Mol. Biol..

[cit12] Harvey W. T., Carabelli A. M., Jackson B., Gupta R. K., Thomson E. C., Harrison E. M., Ludden C., Reeve R., Rambaut A., COVID-19 Genomics UK (COG-UK) Consortium, Peacock S. J., Robertson D. L. (2021). Nat. Rev. Microbiol..

[cit13] Newby M. L., Fogarty C. A., Allen J. D., Butler J., Fadda E., Crispin M. (2023). J. Mol. Biol..

[cit14] Campos D., Girgis M., Sanda M. (2022). Proteomics.

[cit15] Chmielewski D., Wilson E. A., Pintilie G., Zhao P., Chen M., Schmid M. F., Simmons G., Wells L., Jin J., Singharoy A., Chiu W. (2023). Nat. Commun..

[cit16] Watanabe Y., Berndsen Z. T., Raghwani J., Seabright G. E., Allen J. D., Pybus O. G., McLellan J. S., Wilson I. A., Bowden T. A., Ward A. B., Crispin M. (2020). Nat. Commun..

[cit17] Yang T. J., Chang Y. C., Ko T. P., Draczkowski P., Chien Y. C., Chang Y. C., Wu K. P., Khoo K. H., Chang H. W., Hsu S. D. (2020). Proc. Natl. Acad. Sci. U. S. A..

[cit18] Sun Z., Ren K., Zhang X., Chen J., Jiang Z., Jiang J., Ji F., Ouyang X., Li L. (2021). Engineering.

[cit19] Sun Z., Zheng X., Ji F., Zhou M., Su X., Ren K., Li L. (2021). Infect. Microbes Dis..

[cit20] Watanabe Y., Allen J. D., Wrapp D., McLellan J. S., Crispin M. (2020). Science.

[cit21] Zheng L., Ma Y., Chen M., Wu G., Yan C., Zhang X.-E. (2021). Biochem. Biophys. Res. Commun..

[cit22] Huang H.-Y., Liao H.-Y., Chen X., Wang S.-W., Cheng C.-W., Shahed-Al-Mahmud M., Liu Y.-M., Mohapatra A., Chen T.-H., Lo J. M., Wu Y.-M., Ma H.-H., Chang Y.-H., Tsai H.-Y., Chou Y.-C., Hsueh Y.-P., Tsai C.-Y., Huang P.-Y., Chang S.-Y., Chao T.-L., Kao H.-C., Tsai Y.-M., Chen Y.-H., Wu C.-Y., Jan J.-T., Cheng T.-J. R., Lin K.-I., Ma C., Wong C.-H. (2022). Sci. Transl. Med..

[cit23] Hsu Y.-P., Frank M., Mukherjee D., Shchurik V., Makarov A., Mann B. F. (2023). Glycobiol.

[cit24] Stiving A. Q., Foreman D. J., VanAernum Z. L., Durr E., Wang S., Vlasak J., Galli J., Kafader J. O., Tsukidate T., Li X., Schuessler H. A., Richardson D. D. (2024). J. Am. Soc. Mass Spectrom..

[cit25] Sztain T., Ahn S.-H., Bogetti A. T., Casalino L., Goldsmith J. A., Seitz E., McCool R. S., Kearns F. L., Acosta-Reyes F., Maji S., Mashayekhi G., McCammon J. A., Ourmazd A., Frank J., McLellan J. S., Chong L. T., Amaro R. E. (2021). Nat. Chem..

[cit26] Klesse G., Rao S., Tucker S. J., Sansom M. S. (2020). J. Am. Chem. Soc..

[cit27] Ponder J. W., Wu C., Ren P., Pande V. S., Chodera J. D., Schnieders M. J., Haque I., Mobley D. L., Lambrecht D. S., DiStasio R. A., Head-Gordon M., Clark G. N. I., Johnson M. E., Head-Gordon T. (2010). J. Phys. Chem. B.

[cit28] Lagardère L., Jolly L.-H., Lipparini F., Aviat F., Stamm B., Jing Z. F., Harger M., Torabifard H., Cisneros G. A., Schnieders M. J., Gresh N., Maday Y., Ren P. Y., Ponder J. W., Piquemal J.-P. (2018). Chem. Sci..

[cit29] Adjoua O., Lagardère L., Jolly L.-H., Durocher A., Very T., Dupays I., Wang Z., Inizan T. J., Célerse F., Ren P., Ponder J. W., Piquemal J.-P. (2021). J. Chem. Theory Comput..

[cit30] Zhang C., Lu C., Jing Z., Wu C., Piquemal J.-P., Ponder J. W., Ren P. (2018). J. Chem. Theory Comput..

[cit31] Jaffrelot Inizan T., Célerse F., Adjoua O., El Ahdab D., Jolly L.-H., Liu C., Ren P., Montes M., Lagarde N., Lagardère L., Monmarché P., Piquemal J.-P. (2021). Chem. Sci..

[cit32] Wrapp D., Wang N., Corbett K. S., Goldsmith J. A., Hsieh C.-L., Abiona O., Graham B. S., McLellan J. S. (2020). Science.

[cit33] Walls A. C., Park Y.-J., Tortorici M. A., Wall A., McGuire A. T., Veesler D. (2020). Cell.

[cit34] Amaro R. E. , Amaro Lab – COVID-19, https://amarolab.ucsd.edu/covid19.php

[cit35] Podler J. , Jay Podler Lab – Tinker Molecular Modeling Package, https://dasher.wustl.edu/tinker/distribution/doc/tinker-guide.pdf

[cit36] Liu C. , Poltype 2: Automated Parameterization and Free Energy Prediction for AMOEBA , https://github.com/TinkerTools/poltype2

[cit37] Walker B., Liu C., Wait E., Ren P. (2022). J. Comput. Chem..

[cit38] Wu J. J., Chattree G., Ren P. (2012). Theor. Chem. Acc..

[cit39] Lagardère L., Aviat F., Piquemal J.-P. (2019). J. Phys. Chem. Lett..

[cit40] Adjoua O. , Tinker-HP: High-Performance Massively Parallel Evolution of Tinker on CPUs & GPUs, https://github.com/TinkerTools/Tinker-HP

[cit41] Piquemal J.-P. , Lagardère L., Adjoua O. and Jolly L.-H., Tinker-HP: High-Performance Massively Parallel Evolution of Tinker on CPUs & GPUs, https://tinker-hp.org/

[cit42] Hunter J. D. (2007). Comput. Sci. Eng..

[cit43] Shi Y., Xia Z., Zhang J., Best R., Wu C., Ponder J. W., Ren P. (2013). J. Chem. Theory Comput..

[cit44] Chang D., Zaia J. (2019). Mol. Cell. Proteomics.

[cit45] de Groot R. (2006). Glycoconjugate J..

[cit46] Calvaresi V., Wrobel A. G., Toporowska J., Hammerschmid D., Doores K. J., Bradshaw R. T., Parsons R. B., Benton D. J., Roustan C., Reading E., Malim M. H., Gamblin S. J., Politis A. (2023). Nat. Commun..

[cit47] Zhang Y., Zhao W., Mao Y., Chen Y., Wang S., Zhong Y., Su T., Gong M., Du D., Lu X., Cheng J., Yang H. (2021). Mol. Cell. Proteomics.

[cit48] Zhou D., Tian X., Qi R., Peng C., Zhang W. (2021). Glycobiology.

[cit49] Barre A., Van Damme E. J. M., Simplicien M., Le Poder S., Klonjkowski B., Benoist H., Peyrade D., Rougé P. (2021). Cells.

[cit50] Nangarlia A., Hassen F. F., Canziani G., Bandi P., Talukder C., Zhang F., Krauth D., Gary E. N., Weiner D. B., Bieniasz P., Navas-Martin S., O'Keefe B. R., Ang C. G., Chaiken I. (2023). Biochemistry.

[cit51] Tabynov K., Solomadin M., Turebekov N., Babayeva M., Fomin G., Yadagiri G., Renu S., Yerubayev T., Petrovsky N., Renukaradhya G. J., Tabynov K. (2023). Sci. Rep..

[cit52] Grosche V. R., Souza L. P. F., Ferreira G. M., Guevara-Vega M., Carvalho T., Silva R. R. D. S., Batista K. L. R., Abuna R. P. F., Silva J. S., Calmon M. F., Rahal P., da Silva L. C. N., Andrade B. S., Teixeira C. S., Sabino-Silva R., Jardim A. C. G. (2023). Viruses.

[cit53] Nazmul T., Lawal-Ayinde B. M., Morita T., Yoshimoto R., Higashiura A., Yamamoto A., Nomura T., Nakano Y., Hirayama M., Kurokawa H., Kitamura Y., Hori K., Sakaguchi T. (2023). Microbiol. Immunol..

[cit54] Li Y., Xu S., Ye Q., Chi H., Guo Z., Chen J., Wu M., Fan B., Li B., Qin C.-F., Liu Z. (2023). Adv. Sci..

[cit55] Stravalaci M., Pagani I., Zhong H., Sironi M., Bondesan S., Barzaghi F., Carrera P., Izzo F., Zoia E., Bottazzi B., Asselta R., Casari G., Aiuti A., Mantovani A., Garlanda C. (2023). Immunobiology.

[cit56] Sutta A., González-García B., Pérez-Alós L., Rosbjerg A., Garred P., Bayarri-Olmos R. (2023). Immunobiology.

[cit57] Albertini G., Pisani L., Bolzoni A., Annunziata M., Porta C., Pastorelli L. (2024). J. Crohns Colitis.

[cit58] Cramer J., Lakkaichi A., Aliu B., Jakob R. P., Klein S., Cattaneo I., Jiang X., Rabbani S., Schwardt O., Zimmer G., Ciancaglini M., Abreu Mota T., Maier T., Ernst B. (2021). J. Am. Chem. Soc..

[cit59] Gupta A., Yadav K., Yadav A., Ahmad R., Srivastava A., Kumar D., Khan M. A., Dwivedi U. N. (2024). Glycoconjugate J..

[cit60] Gray R. D. M., Albrecht D., Beerli C., Huttunen M., Cohen G. H., White I. J., Burden J. J., Henriques R., Mercer J. (2019). Nat. Microbiol..

[cit61] Thomaston J. L., Woldeyes R. A., Nakane T., Yamashita A., Tanaka T., Koiwai K., Brewster A. S., Barad B. A., Chen Y., Lemmin T. (2017). et al.. Proc. Natl. Acad. Sci. U.S.A..

[cit62] Williams J. K., Tietze D., Lee M., Wang J., Hong M. (2016). J. Am. Chem. Soc..

[cit63] Shepelenko S. O., Salnikov A. S., Rak S. V., Goncharova E. P., Ryzhikov A. B. (2009). J. Phys.: Conf. Ser..

[cit64] Svergun D. I., Richard S., Koch M. H. J., Sayers Z., Kuprin S., Zaccai G. (1998). Proc. Natl. Acad. Sci. U.S.A..

[cit65] Halle B., Nilsson L. (2009). J. Phys. Chem. B.

[cit66] Mukherjee S., Mondal S., Bagchi B. (2017). J. Chem. Phys..

[cit67] Ebbinghaus S., Kim S. J., Heyden M., Yu X., Heugen U., Gruebele M., Leitner D. M., Havenith M. (2007). Proc. Natl. Acad. Sci. U.S.A..

[cit68] Fuentes-Azcatl R., Alejandre J. (2014). J. Phys. Chem. B.

[cit69] Ren P. Y., Ponder J. W. (2003). J. Phys. Chem..

[cit70] Sanner M. F., Olson A. J., Spehner J.-C. (1996). Biopolymers.

[cit71] Humphrey W., Dalke A., Schulten K. (1996). J. Mol. Graphics.

