# Supplementary figures and images for: Water–glycan interactions drive the SARS-CoV-2 spike dynamics: insights into glycan-gate control and camouflage mechanisms (part 2 of 4)
Source: Chem Sci. 2024 Aug 23;15(35):14177–87. doi: 10.1039/d4sc04364b (PMC11359970; doi:10.1039/d4sc04364b)

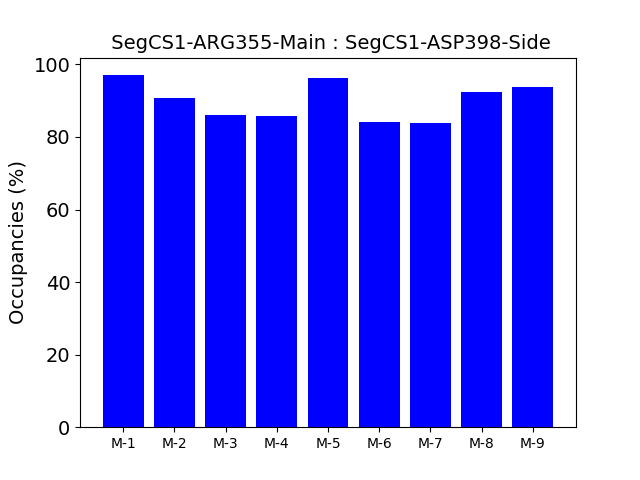

Supplement: SC-015-D4SC04364B-s001 [file SC-015-D4SC04364B-s001.zip › Inner_h_bonds_states/open/SegCS1-ARG355-Main_SegCS1-ASP398-Side.png]

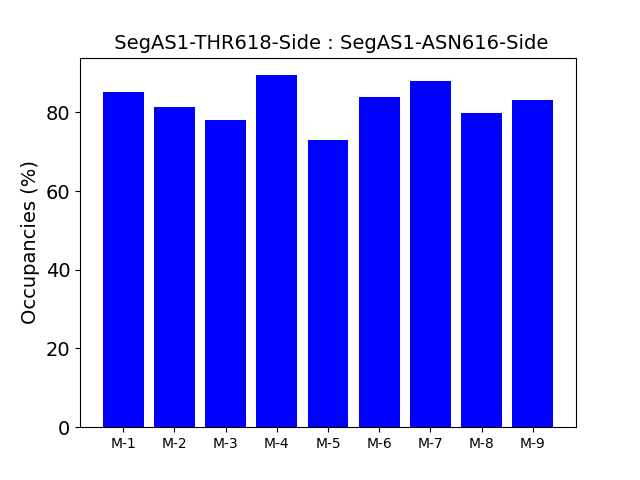

Supplement: SC-015-D4SC04364B-s001 [file SC-015-D4SC04364B-s001.zip › Inner_h_bonds_states/open/SegAS1-THR618-Side_SegAS1-ASN616-Side.png]

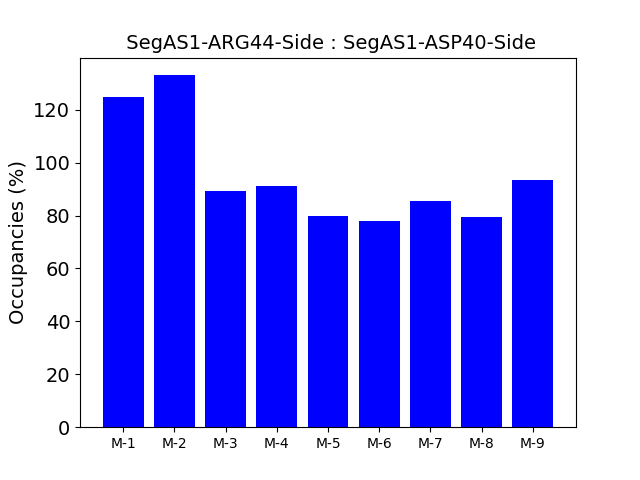

Supplement: SC-015-D4SC04364B-s001 [file SC-015-D4SC04364B-s001.zip › Inner_h_bonds_states/open/SegAS1-ARG44-Side_SegAS1-ASP40-Side.png]

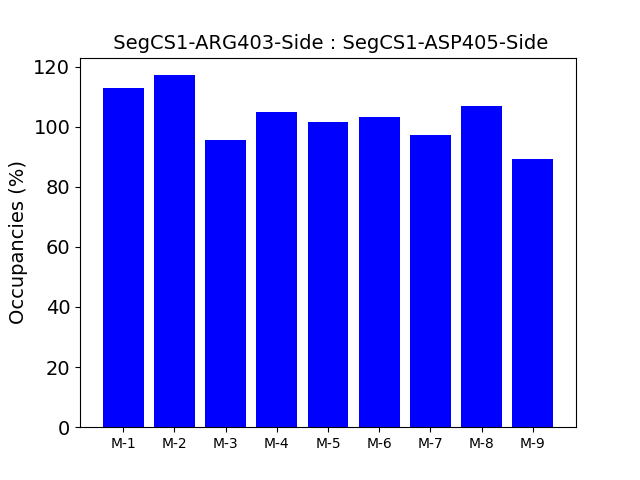

Supplement: SC-015-D4SC04364B-s001 [file SC-015-D4SC04364B-s001.zip › Inner_h_bonds_states/open/SegCS1-ARG403-Side_SegCS1-ASP405-Side.png]

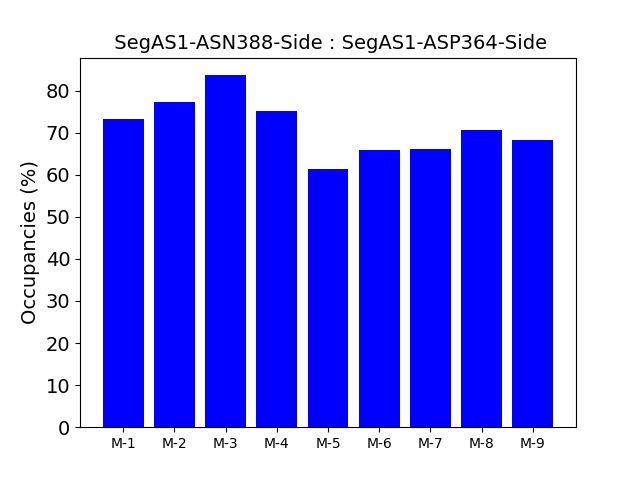

Supplement: SC-015-D4SC04364B-s001 [file SC-015-D4SC04364B-s001.zip › Inner_h_bonds_states/closed/SegAS1-ASN388-Side_SegAS1-ASP364-Side.png]

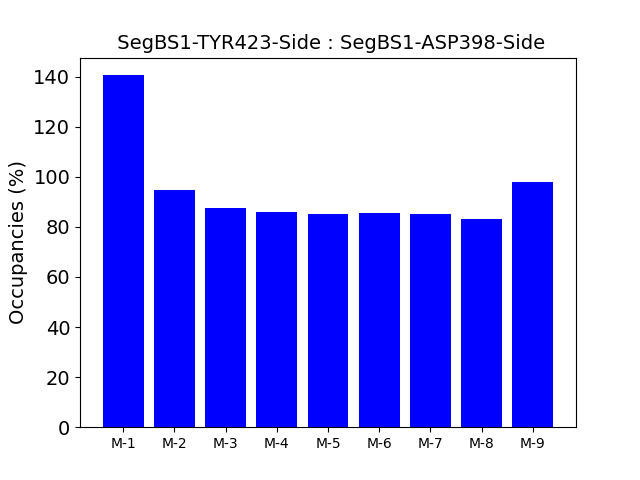

Supplement: SC-015-D4SC04364B-s001 [file SC-015-D4SC04364B-s001.zip › Inner_h_bonds_states/closed/SegBS1-TYR423-Side_SegBS1-ASP398-Side.png]

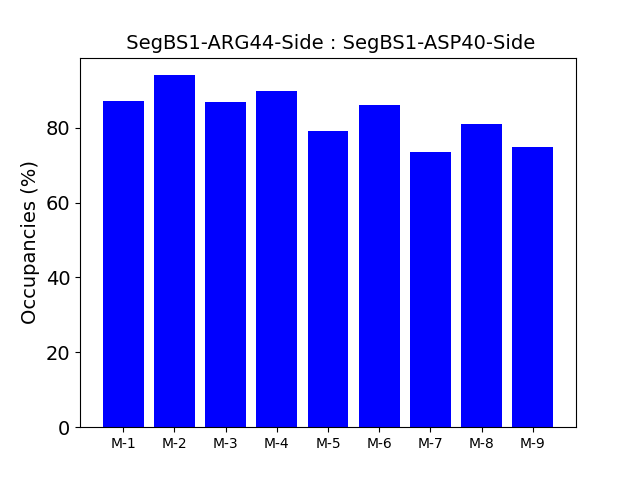

Supplement: SC-015-D4SC04364B-s001 [file SC-015-D4SC04364B-s001.zip › Inner_h_bonds_states/closed/SegBS1-ARG44-Side_SegBS1-ASP40-Side.png]

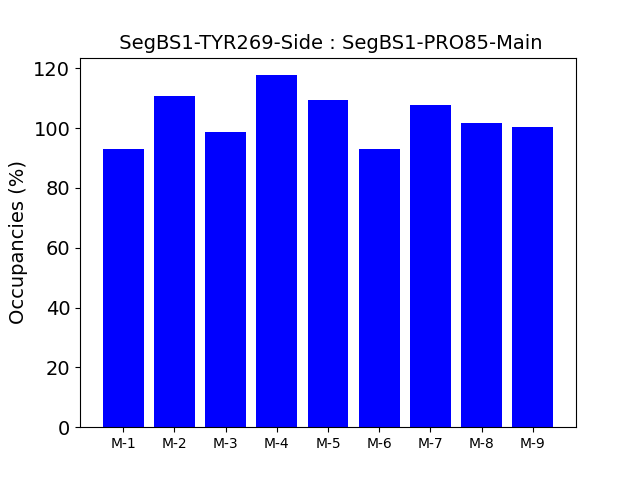

Supplement: SC-015-D4SC04364B-s001 [file SC-015-D4SC04364B-s001.zip › Inner_h_bonds_states/closed/SegBS1-TYR269-Side_SegBS1-PRO85-Main.png]

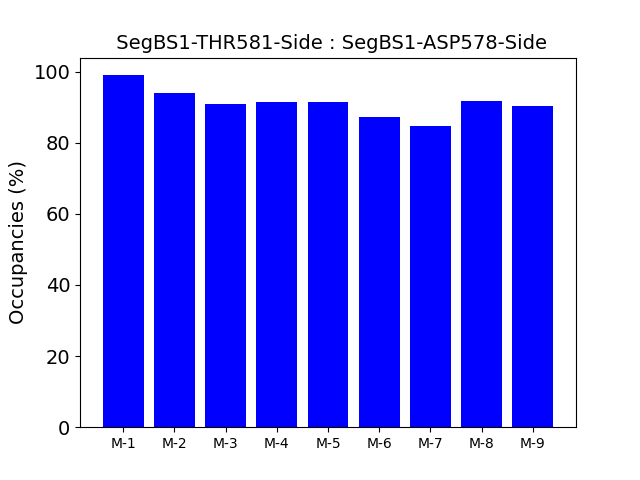

Supplement: SC-015-D4SC04364B-s001 [file SC-015-D4SC04364B-s001.zip › Inner_h_bonds_states/closed/SegBS1-THR581-Side_SegBS1-ASP578-Side.png]

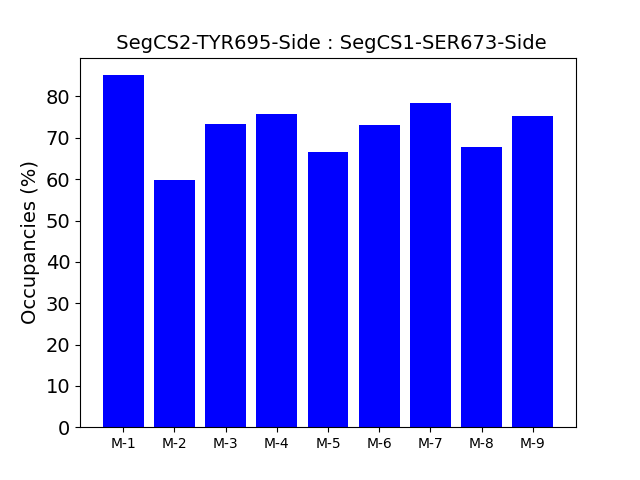

Supplement: SC-015-D4SC04364B-s001 [file SC-015-D4SC04364B-s001.zip › Inner_h_bonds_states/closed/SegCS2-TYR695-Side_SegCS1-SER673-Side.png]

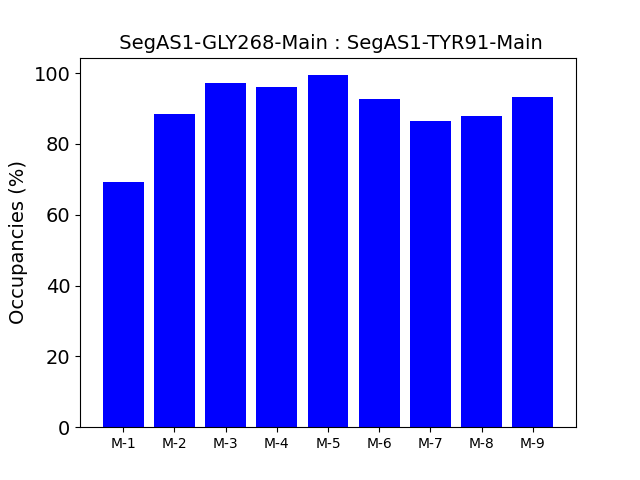

Supplement: SC-015-D4SC04364B-s001 [file SC-015-D4SC04364B-s001.zip › Inner_h_bonds_states/closed/SegAS1-GLY268-Main_SegAS1-TYR91-Main.png]

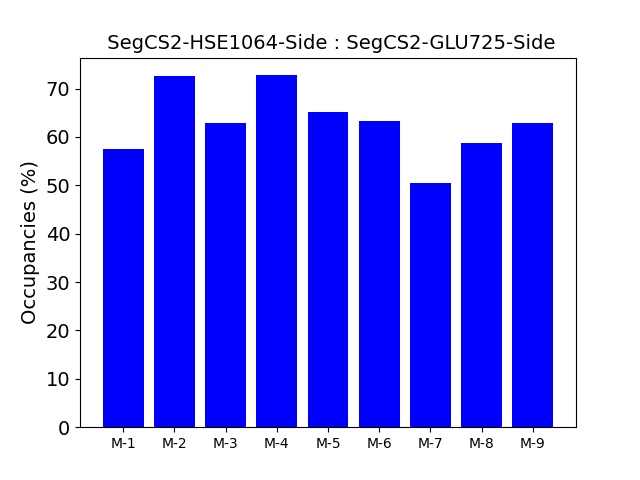

Supplement: SC-015-D4SC04364B-s001 [file SC-015-D4SC04364B-s001.zip › Inner_h_bonds_states/closed/SegCS2-HSE1064-Side_SegCS2-GLU725-Side.png]

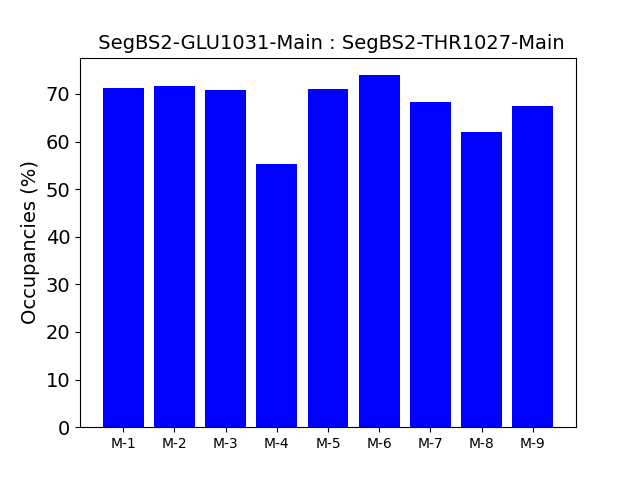

Supplement: SC-015-D4SC04364B-s001 [file SC-015-D4SC04364B-s001.zip › Inner_h_bonds_states/closed/SegBS2-GLU1031-Main_SegBS2-THR1027-Main.png]

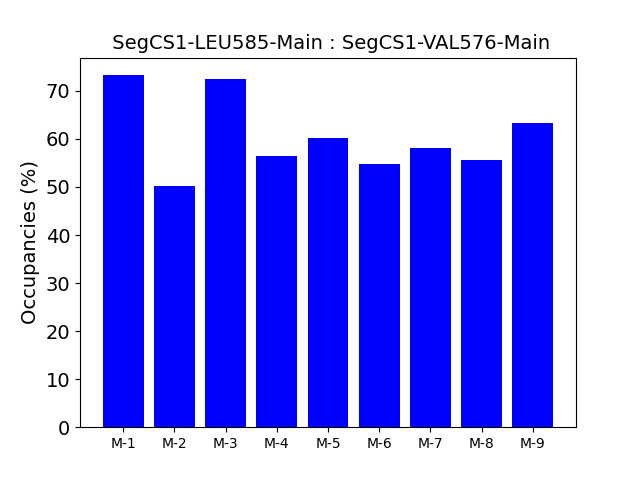

Supplement: SC-015-D4SC04364B-s001 [file SC-015-D4SC04364B-s001.zip › Inner_h_bonds_states/closed/SegCS1-LEU585-Main_SegCS1-VAL576-Main.png]

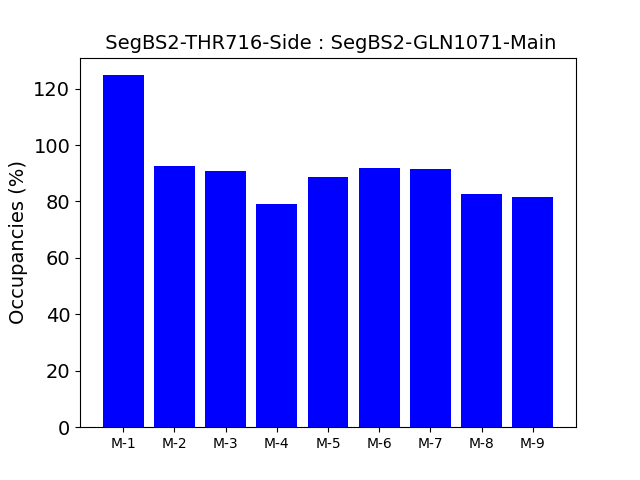

Supplement: SC-015-D4SC04364B-s001 [file SC-015-D4SC04364B-s001.zip › Inner_h_bonds_states/closed/SegBS2-THR716-Side_SegBS2-GLN1071-Main.png]

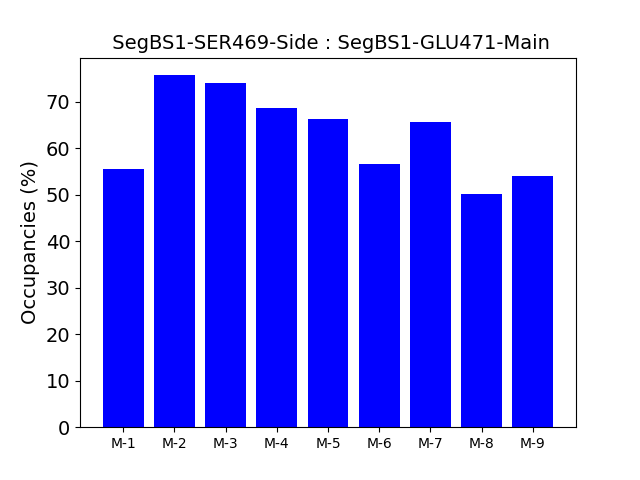

Supplement: SC-015-D4SC04364B-s001 [file SC-015-D4SC04364B-s001.zip › Inner_h_bonds_states/closed/SegBS1-SER469-Side_SegBS1-GLU471-Main.png]

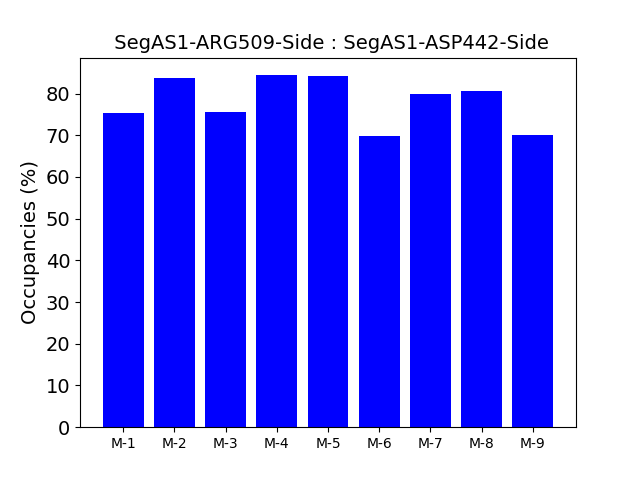

Supplement: SC-015-D4SC04364B-s001 [file SC-015-D4SC04364B-s001.zip › Inner_h_bonds_states/closed/SegAS1-ARG509-Side_SegAS1-ASP442-Side.png]

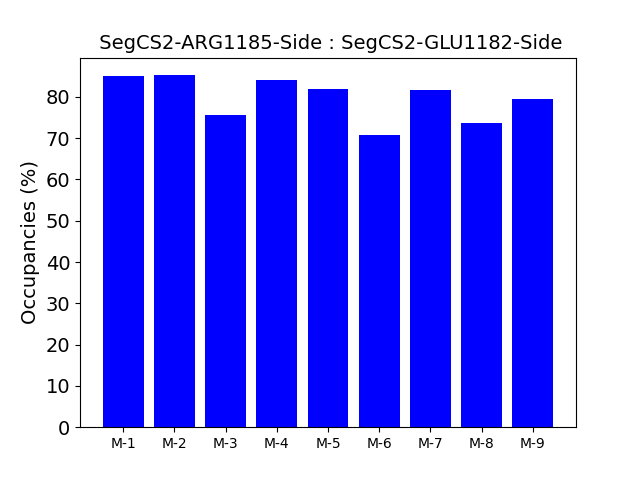

Supplement: SC-015-D4SC04364B-s001 [file SC-015-D4SC04364B-s001.zip › Inner_h_bonds_states/closed/SegCS2-ARG1185-Side_SegCS2-GLU1182-Side.png]

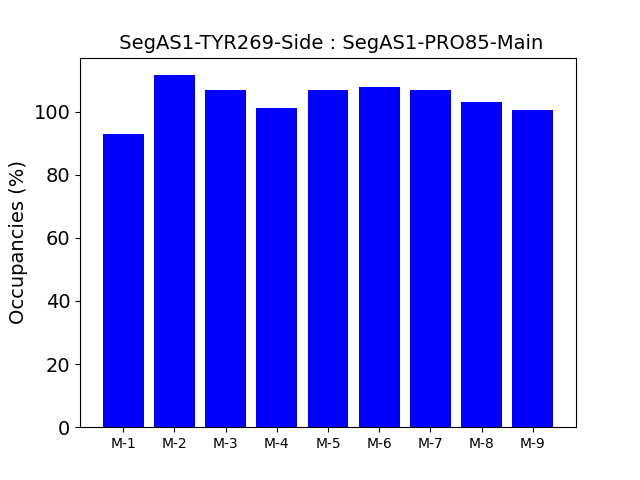

Supplement: SC-015-D4SC04364B-s001 [file SC-015-D4SC04364B-s001.zip › Inner_h_bonds_states/closed/SegAS1-TYR269-Side_SegAS1-PRO85-Main.png]

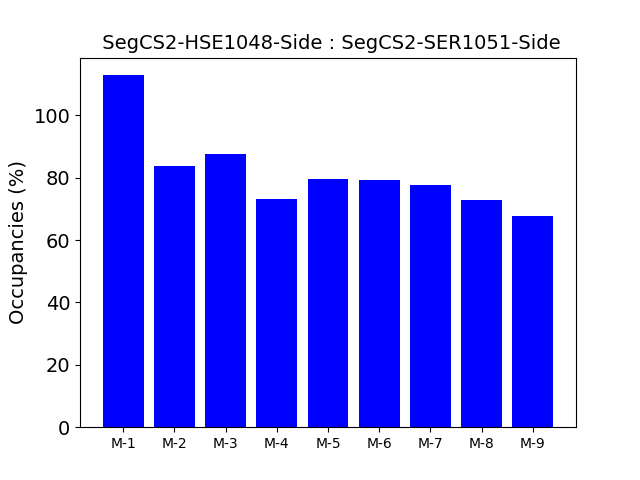

Supplement: SC-015-D4SC04364B-s001 [file SC-015-D4SC04364B-s001.zip › Inner_h_bonds_states/closed/SegCS2-HSE1048-Side_SegCS2-SER1051-Side.png]

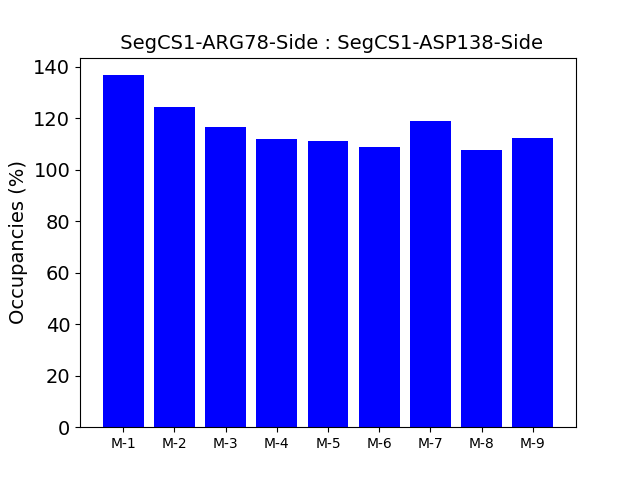

Supplement: SC-015-D4SC04364B-s001 [file SC-015-D4SC04364B-s001.zip › Inner_h_bonds_states/closed/SegCS1-ARG78-Side_SegCS1-ASP138-Side.png]

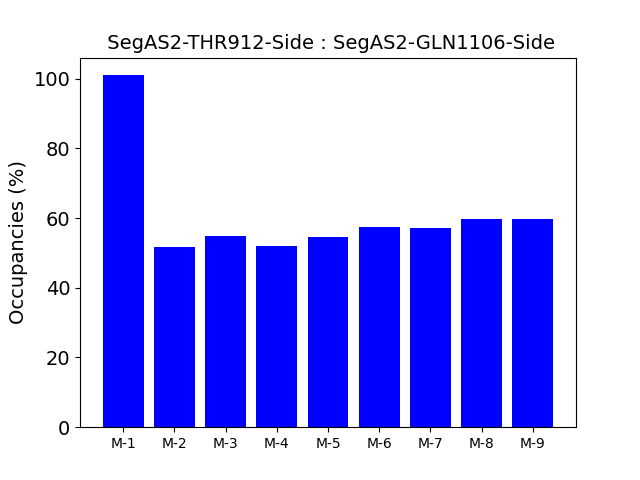

Supplement: SC-015-D4SC04364B-s001 [file SC-015-D4SC04364B-s001.zip › Inner_h_bonds_states/closed/SegAS2-THR912-Side_SegAS2-GLN1106-Side.png]

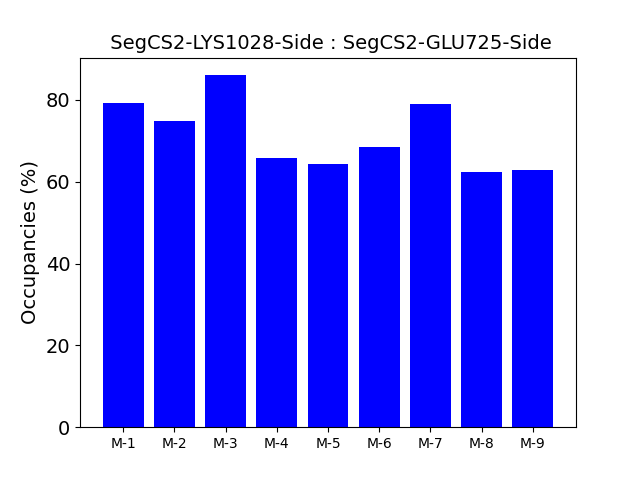

Supplement: SC-015-D4SC04364B-s001 [file SC-015-D4SC04364B-s001.zip › Inner_h_bonds_states/closed/SegCS2-LYS1028-Side_SegCS2-GLU725-Side.png]

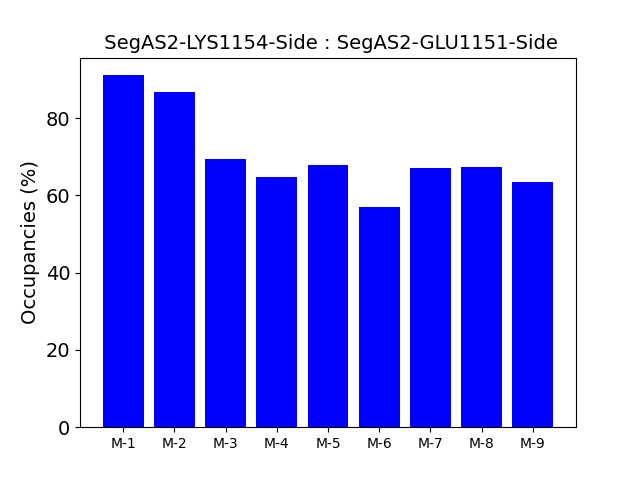

Supplement: SC-015-D4SC04364B-s001 [file SC-015-D4SC04364B-s001.zip › Inner_h_bonds_states/closed/SegAS2-LYS1154-Side_SegAS2-GLU1151-Side.png]

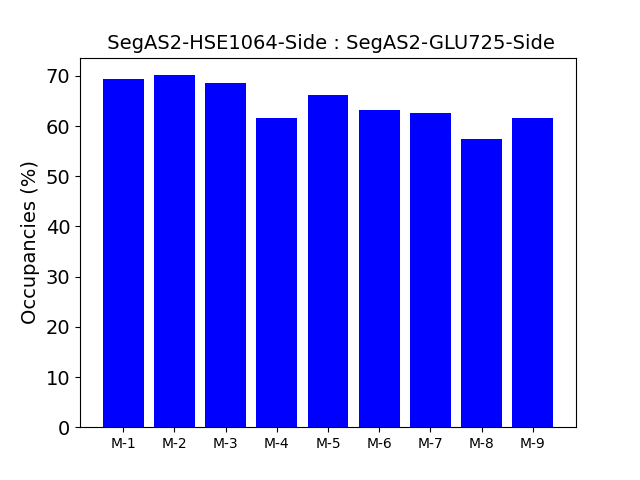

Supplement: SC-015-D4SC04364B-s001 [file SC-015-D4SC04364B-s001.zip › Inner_h_bonds_states/closed/SegAS2-HSE1064-Side_SegAS2-GLU725-Side.png]

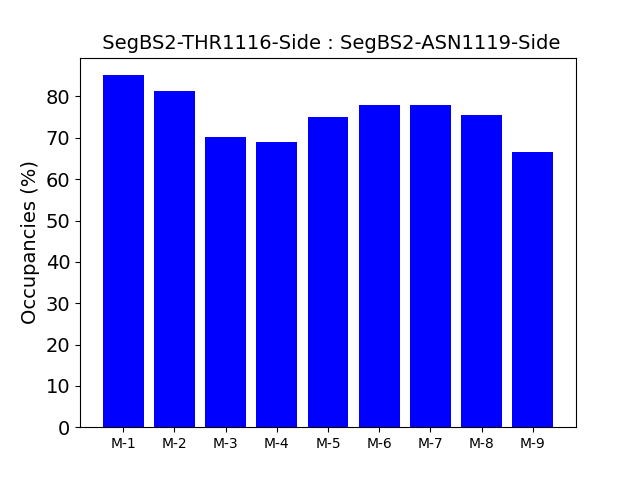

Supplement: SC-015-D4SC04364B-s001 [file SC-015-D4SC04364B-s001.zip › Inner_h_bonds_states/closed/SegBS2-THR1116-Side_SegBS2-ASN1119-Side.png]

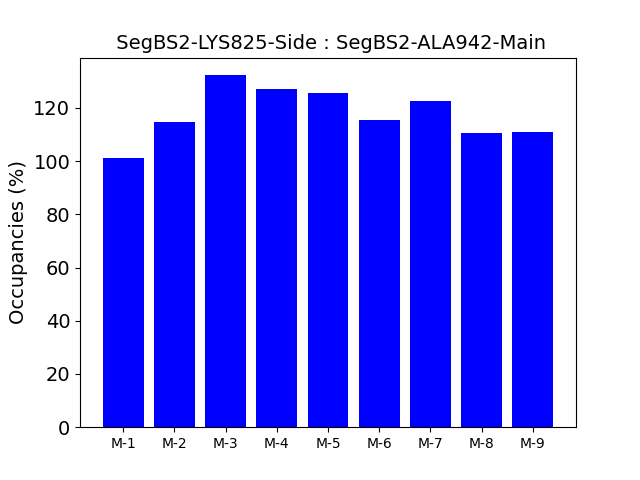

Supplement: SC-015-D4SC04364B-s001 [file SC-015-D4SC04364B-s001.zip › Inner_h_bonds_states/closed/SegBS2-LYS825-Side_SegBS2-ALA942-Main.png]

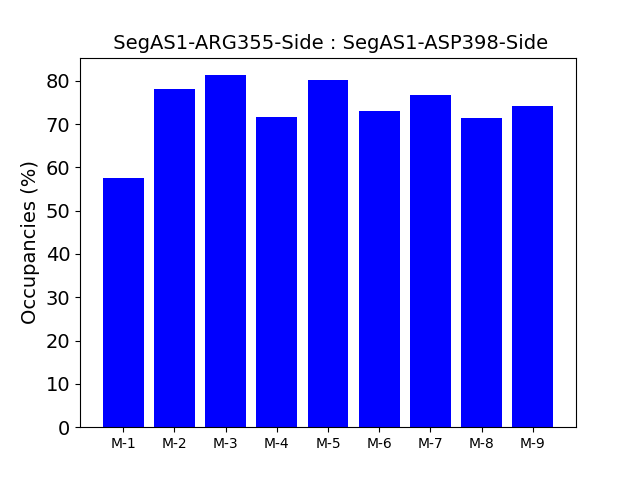

Supplement: SC-015-D4SC04364B-s001 [file SC-015-D4SC04364B-s001.zip › Inner_h_bonds_states/closed/SegAS1-ARG355-Side_SegAS1-ASP398-Side.png]

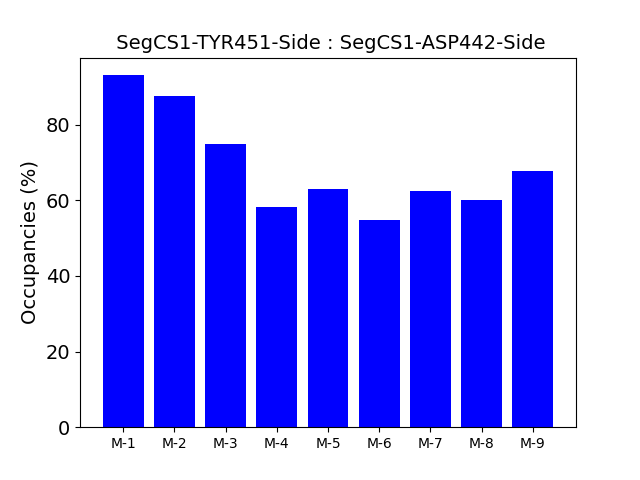

Supplement: SC-015-D4SC04364B-s001 [file SC-015-D4SC04364B-s001.zip › Inner_h_bonds_states/closed/SegCS1-TYR451-Side_SegCS1-ASP442-Side.png]

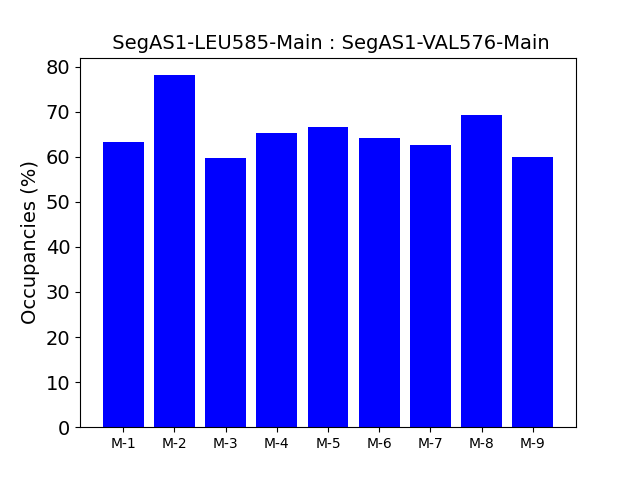

Supplement: SC-015-D4SC04364B-s001 [file SC-015-D4SC04364B-s001.zip › Inner_h_bonds_states/closed/SegAS1-LEU585-Main_SegAS1-VAL576-Main.png]

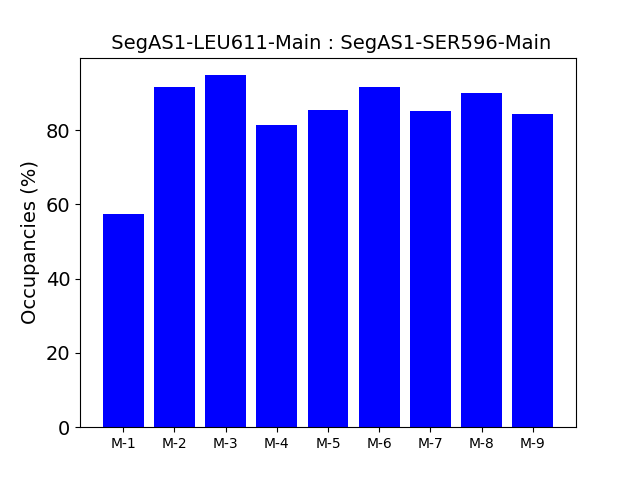

Supplement: SC-015-D4SC04364B-s001 [file SC-015-D4SC04364B-s001.zip › Inner_h_bonds_states/closed/SegAS1-LEU611-Main_SegAS1-SER596-Main.png]

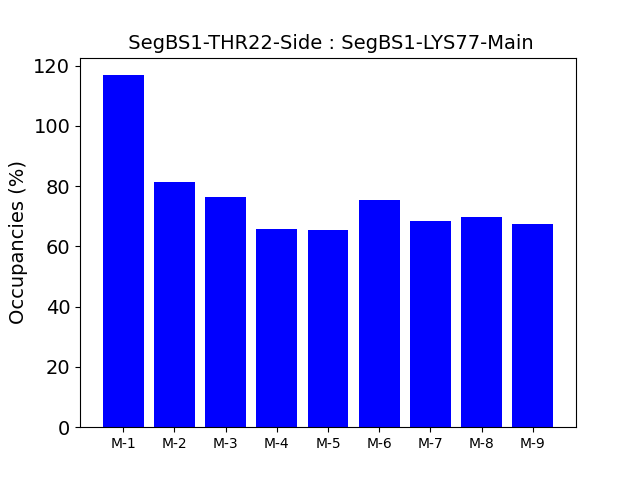

Supplement: SC-015-D4SC04364B-s001 [file SC-015-D4SC04364B-s001.zip › Inner_h_bonds_states/closed/SegBS1-THR22-Side_SegBS1-LYS77-Main.png]

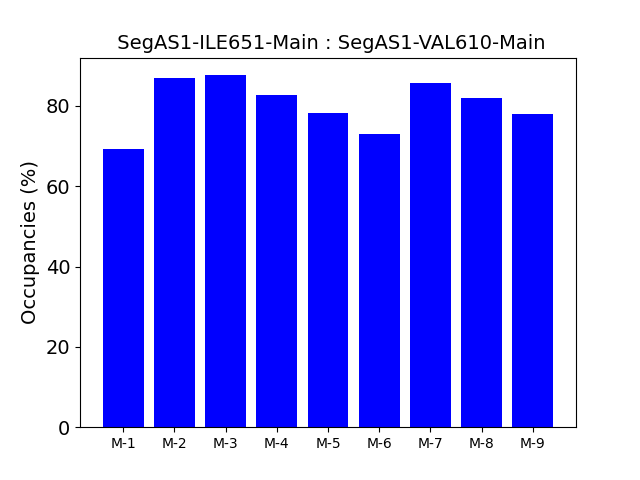

Supplement: SC-015-D4SC04364B-s001 [file SC-015-D4SC04364B-s001.zip › Inner_h_bonds_states/closed/SegAS1-ILE651-Main_SegAS1-VAL610-Main.png]

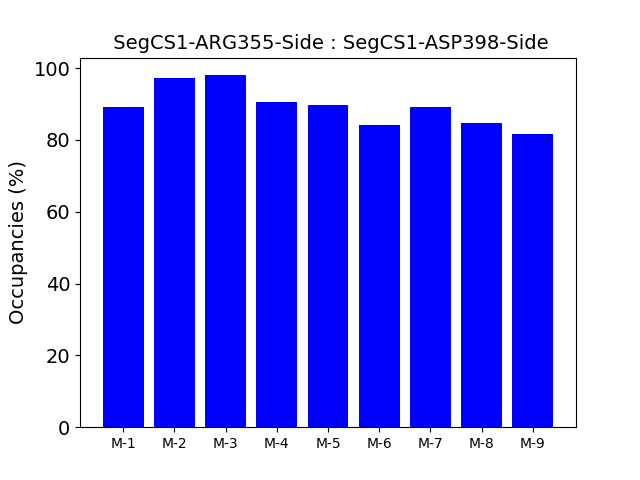

Supplement: SC-015-D4SC04364B-s001 [file SC-015-D4SC04364B-s001.zip › Inner_h_bonds_states/closed/SegCS1-ARG355-Side_SegCS1-ASP398-Side.png]

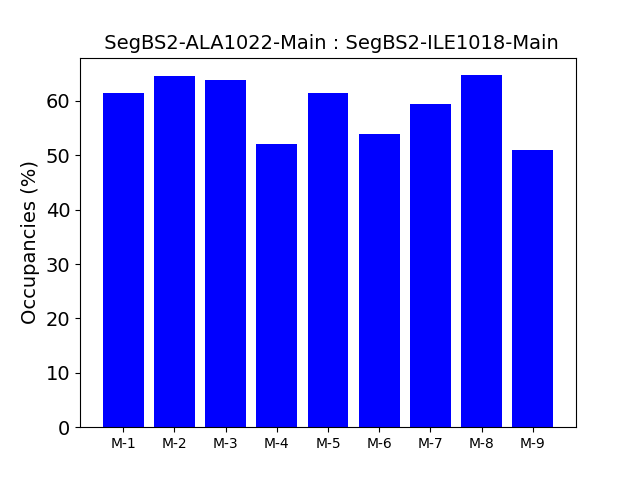

Supplement: SC-015-D4SC04364B-s001 [file SC-015-D4SC04364B-s001.zip › Inner_h_bonds_states/closed/SegBS2-ALA1022-Main_SegBS2-ILE1018-Main.png]

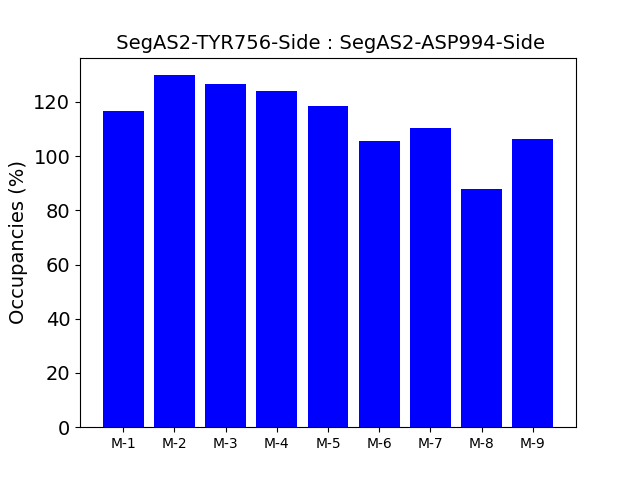

Supplement: SC-015-D4SC04364B-s001 [file SC-015-D4SC04364B-s001.zip › Inner_h_bonds_states/closed/SegAS2-TYR756-Side_SegAS2-ASP994-Side.png]

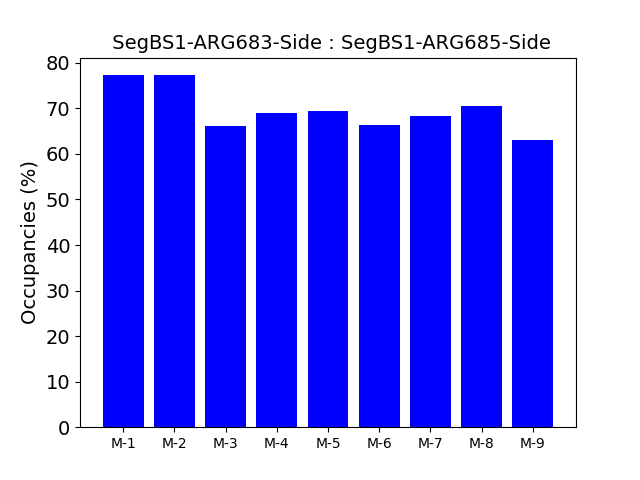

Supplement: SC-015-D4SC04364B-s001 [file SC-015-D4SC04364B-s001.zip › Inner_h_bonds_states/closed/SegBS1-ARG683-Side_SegBS1-ARG685-Side.png]

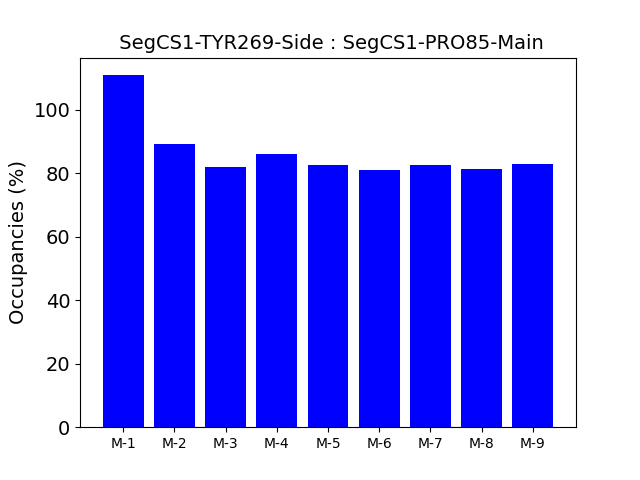

Supplement: SC-015-D4SC04364B-s001 [file SC-015-D4SC04364B-s001.zip › Inner_h_bonds_states/closed/SegCS1-TYR269-Side_SegCS1-PRO85-Main.png]

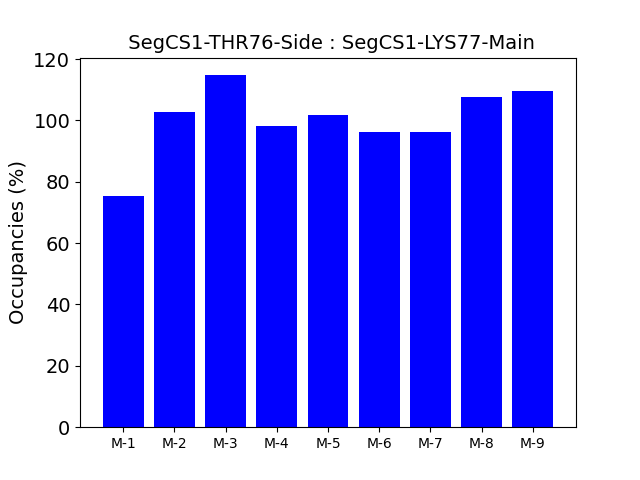

Supplement: SC-015-D4SC04364B-s001 [file SC-015-D4SC04364B-s001.zip › Inner_h_bonds_states/closed/SegCS1-THR76-Side_SegCS1-LYS77-Main.png]

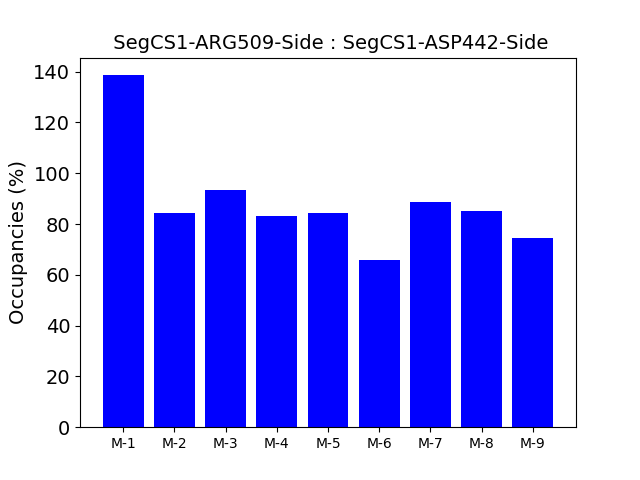

Supplement: SC-015-D4SC04364B-s001 [file SC-015-D4SC04364B-s001.zip › Inner_h_bonds_states/closed/SegCS1-ARG509-Side_SegCS1-ASP442-Side.png]

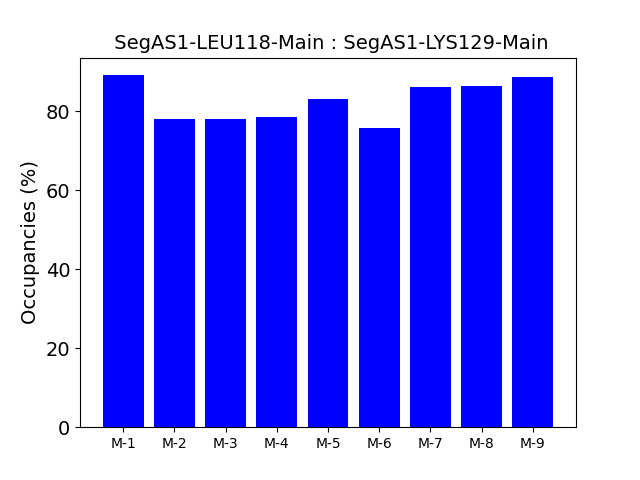

Supplement: SC-015-D4SC04364B-s001 [file SC-015-D4SC04364B-s001.zip › Inner_h_bonds_states/closed/SegAS1-LEU118-Main_SegAS1-LYS129-Main.png]

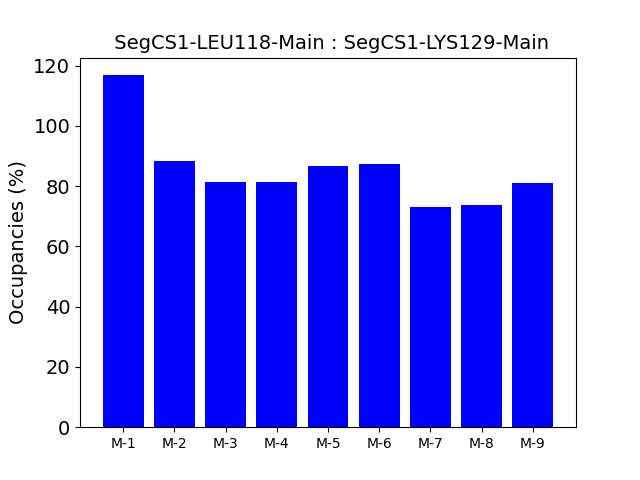

Supplement: SC-015-D4SC04364B-s001 [file SC-015-D4SC04364B-s001.zip › Inner_h_bonds_states/closed/SegCS1-LEU118-Main_SegCS1-LYS129-Main.png]

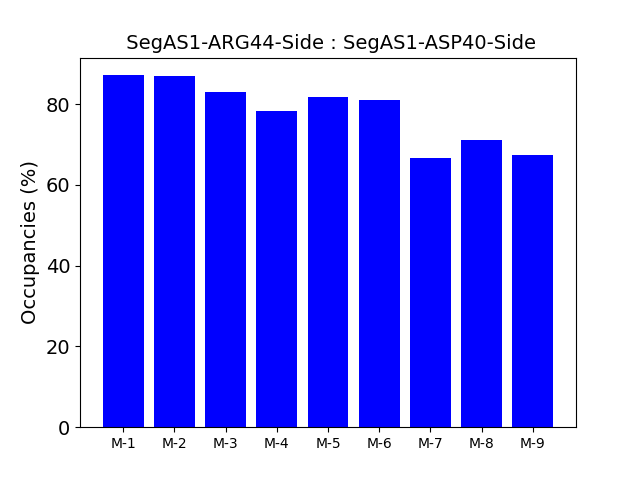

Supplement: SC-015-D4SC04364B-s001 [file SC-015-D4SC04364B-s001.zip › Inner_h_bonds_states/closed/SegAS1-ARG44-Side_SegAS1-ASP40-Side.png]

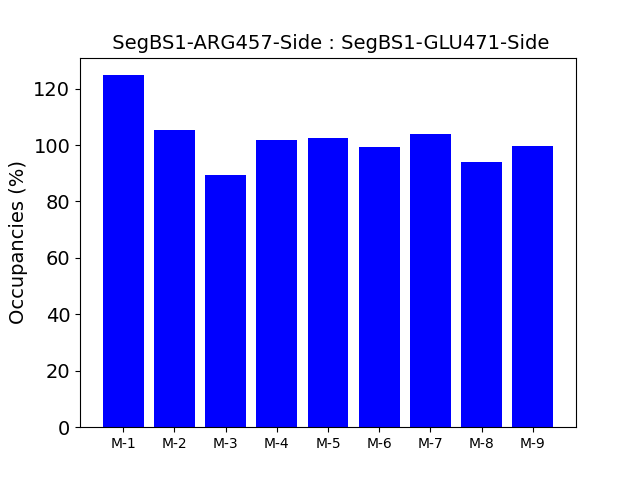

Supplement: SC-015-D4SC04364B-s001 [file SC-015-D4SC04364B-s001.zip › Inner_h_bonds_states/closed/SegBS1-ARG457-Side_SegBS1-GLU471-Side.png]

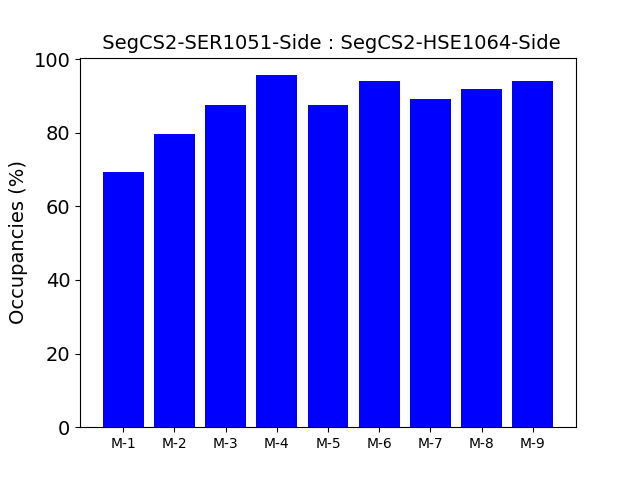

Supplement: SC-015-D4SC04364B-s001 [file SC-015-D4SC04364B-s001.zip › Inner_h_bonds_states/closed/SegCS2-SER1051-Side_SegCS2-HSE1064-Side.png]

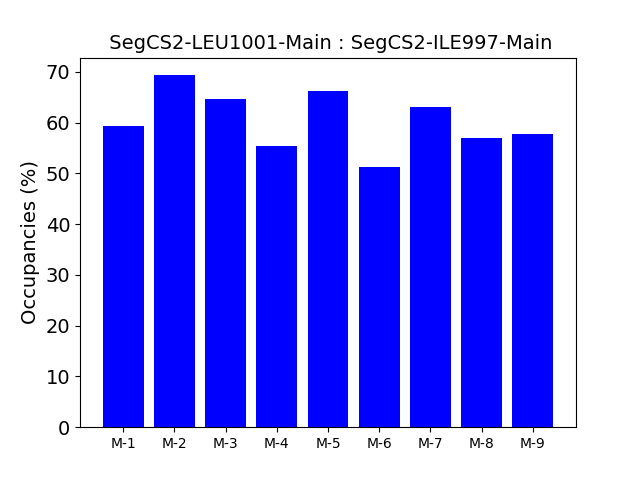

Supplement: SC-015-D4SC04364B-s001 [file SC-015-D4SC04364B-s001.zip › Inner_h_bonds_states/closed/SegCS2-LEU1001-Main_SegCS2-ILE997-Main.png]

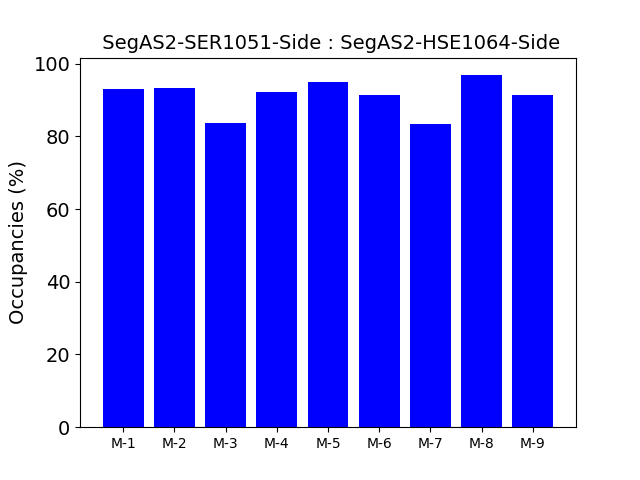

Supplement: SC-015-D4SC04364B-s001 [file SC-015-D4SC04364B-s001.zip › Inner_h_bonds_states/closed/SegAS2-SER1051-Side_SegAS2-HSE1064-Side.png]

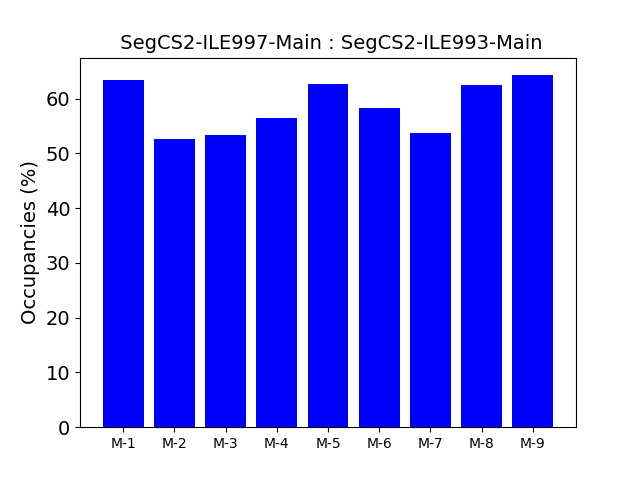

Supplement: SC-015-D4SC04364B-s001 [file SC-015-D4SC04364B-s001.zip › Inner_h_bonds_states/closed/SegCS2-ILE997-Main_SegCS2-ILE993-Main.png]

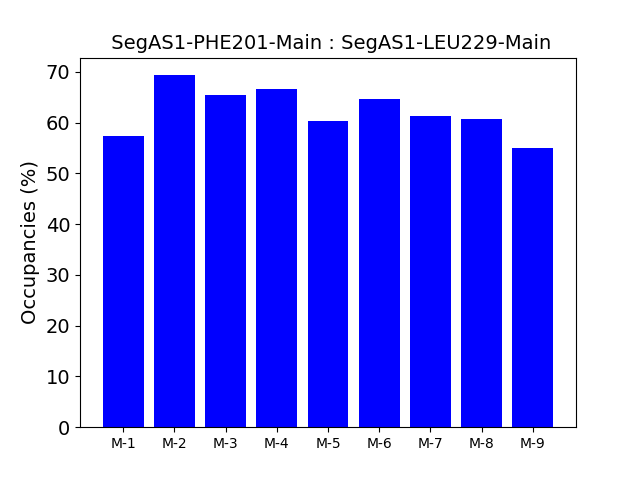

Supplement: SC-015-D4SC04364B-s001 [file SC-015-D4SC04364B-s001.zip › Inner_h_bonds_states/closed/SegAS1-PHE201-Main_SegAS1-LEU229-Main.png]

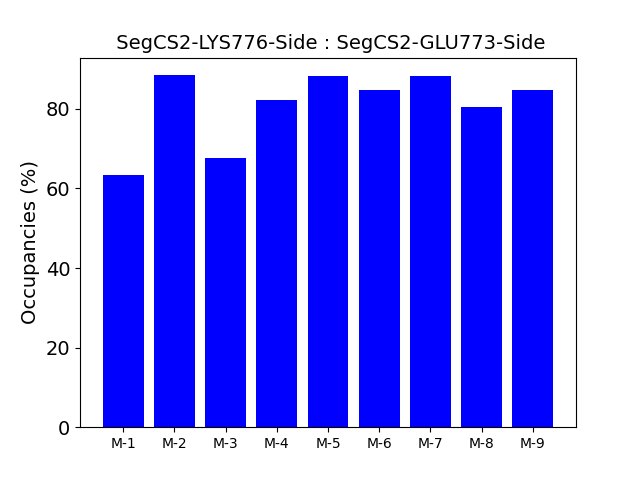

Supplement: SC-015-D4SC04364B-s001 [file SC-015-D4SC04364B-s001.zip › Inner_h_bonds_states/closed/SegCS2-LYS776-Side_SegCS2-GLU773-Side.png]

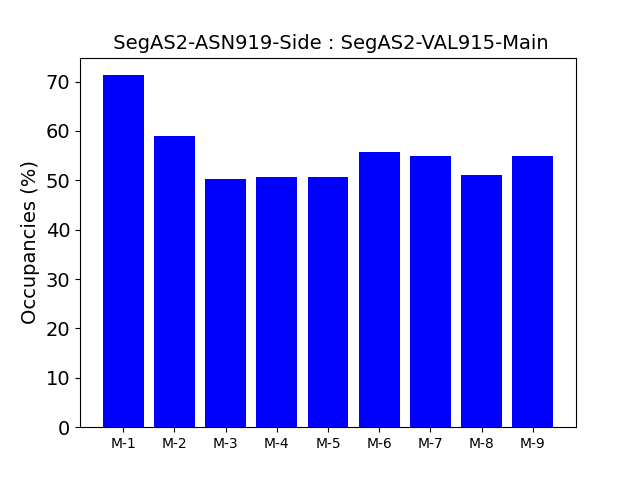

Supplement: SC-015-D4SC04364B-s001 [file SC-015-D4SC04364B-s001.zip › Inner_h_bonds_states/closed/SegAS2-ASN919-Side_SegAS2-VAL915-Main.png]

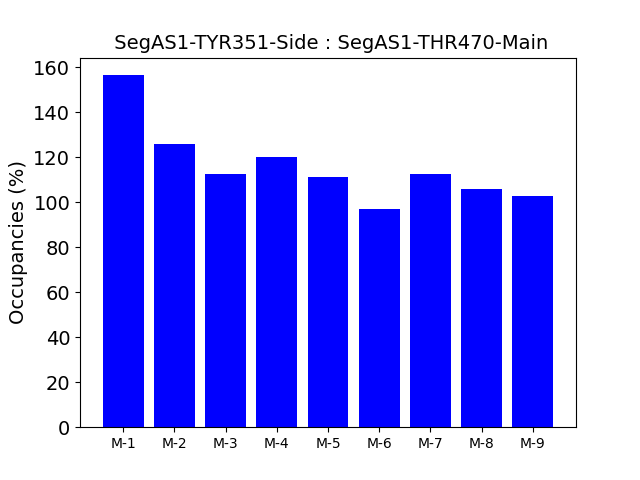

Supplement: SC-015-D4SC04364B-s001 [file SC-015-D4SC04364B-s001.zip › Inner_h_bonds_states/closed/SegAS1-TYR351-Side_SegAS1-THR470-Main.png]

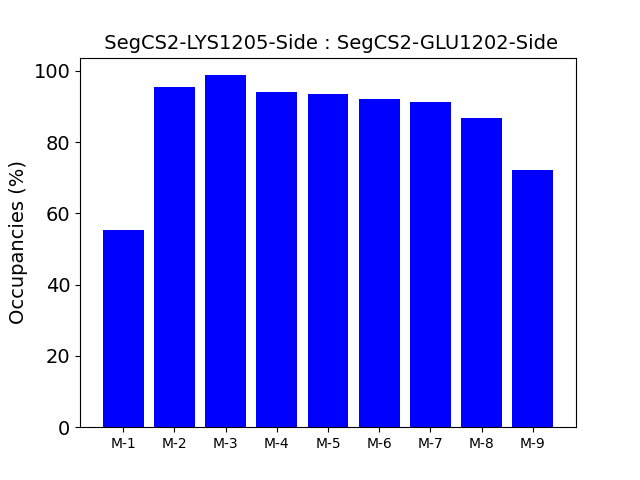

Supplement: SC-015-D4SC04364B-s001 [file SC-015-D4SC04364B-s001.zip › Inner_h_bonds_states/closed/SegCS2-LYS1205-Side_SegCS2-GLU1202-Side.png]

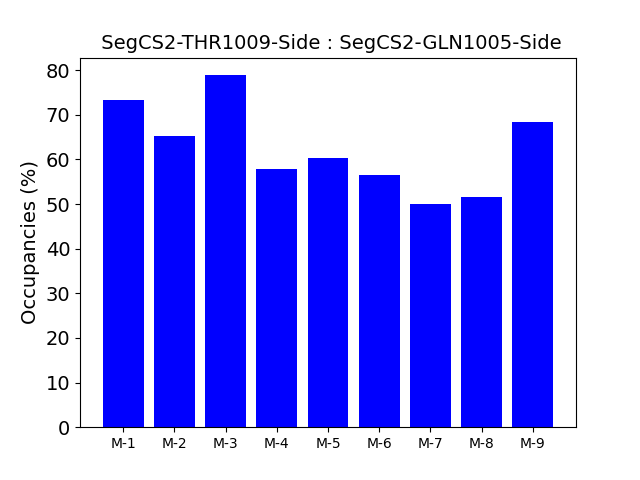

Supplement: SC-015-D4SC04364B-s001 [file SC-015-D4SC04364B-s001.zip › Inner_h_bonds_states/closed/SegCS2-THR1009-Side_SegCS2-GLN1005-Side.png]

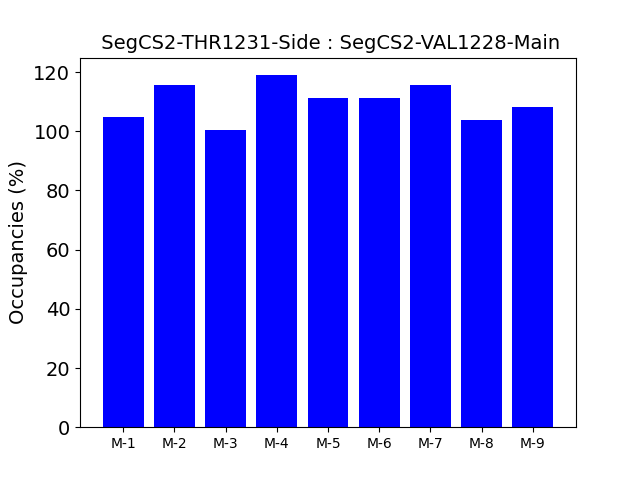

Supplement: SC-015-D4SC04364B-s001 [file SC-015-D4SC04364B-s001.zip › Inner_h_bonds_states/closed/SegCS2-THR1231-Side_SegCS2-VAL1228-Main.png]

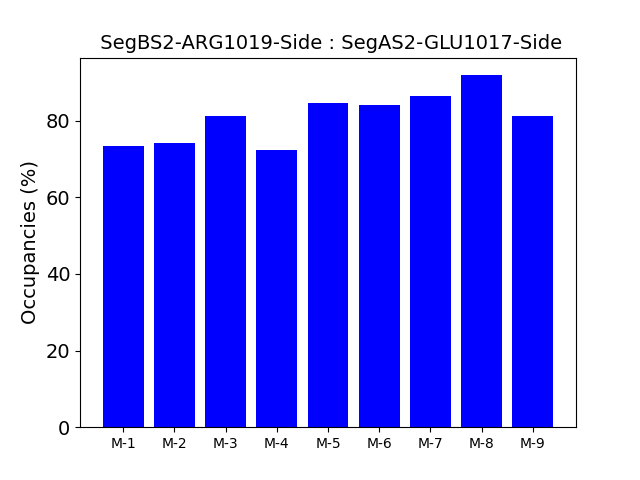

Supplement: SC-015-D4SC04364B-s001 [file SC-015-D4SC04364B-s001.zip › Inner_h_bonds_states/closed/SegBS2-ARG1019-Side_SegAS2-GLU1017-Side.png]

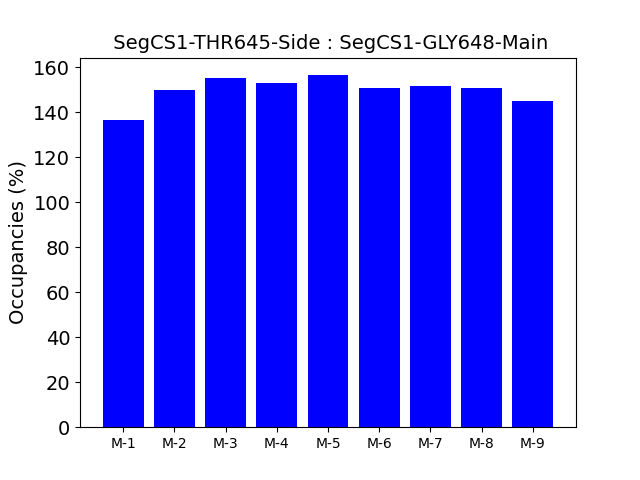

Supplement: SC-015-D4SC04364B-s001 [file SC-015-D4SC04364B-s001.zip › Inner_h_bonds_states/closed/SegCS1-THR645-Side_SegCS1-GLY648-Main.png]

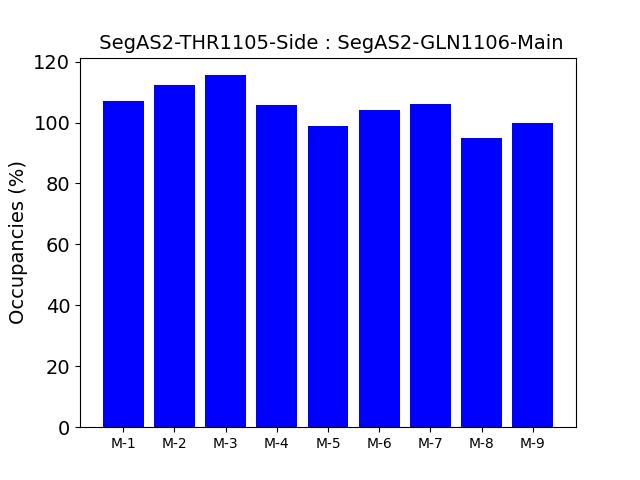

Supplement: SC-015-D4SC04364B-s001 [file SC-015-D4SC04364B-s001.zip › Inner_h_bonds_states/closed/SegAS2-THR1105-Side_SegAS2-GLN1106-Main.png]

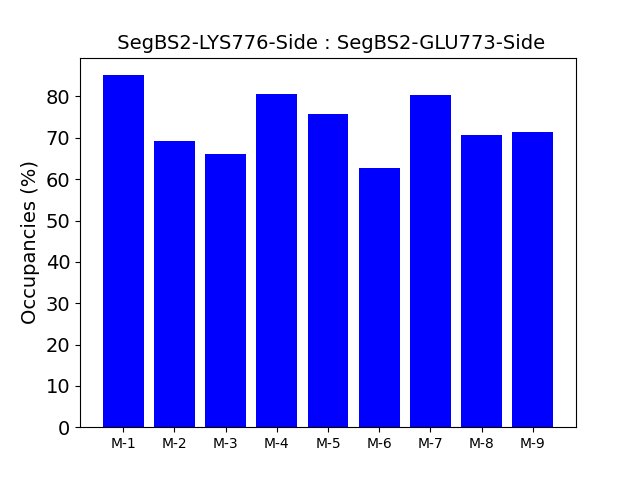

Supplement: SC-015-D4SC04364B-s001 [file SC-015-D4SC04364B-s001.zip › Inner_h_bonds_states/closed/SegBS2-LYS776-Side_SegBS2-GLU773-Side.png]

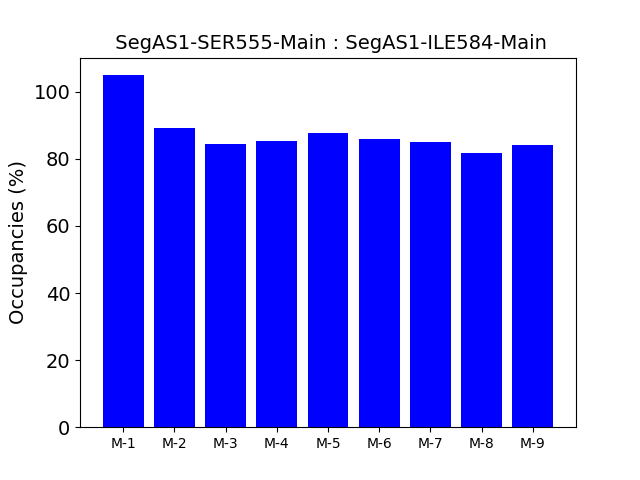

Supplement: SC-015-D4SC04364B-s001 [file SC-015-D4SC04364B-s001.zip › Inner_h_bonds_states/closed/SegAS1-SER555-Main_SegAS1-ILE584-Main.png]

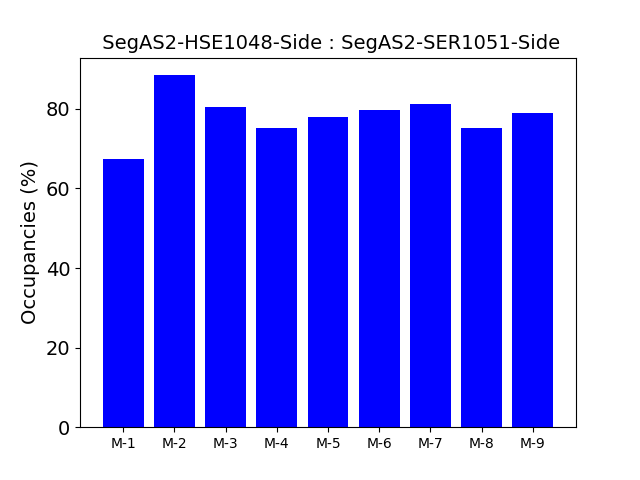

Supplement: SC-015-D4SC04364B-s001 [file SC-015-D4SC04364B-s001.zip › Inner_h_bonds_states/closed/SegAS2-HSE1048-Side_SegAS2-SER1051-Side.png]

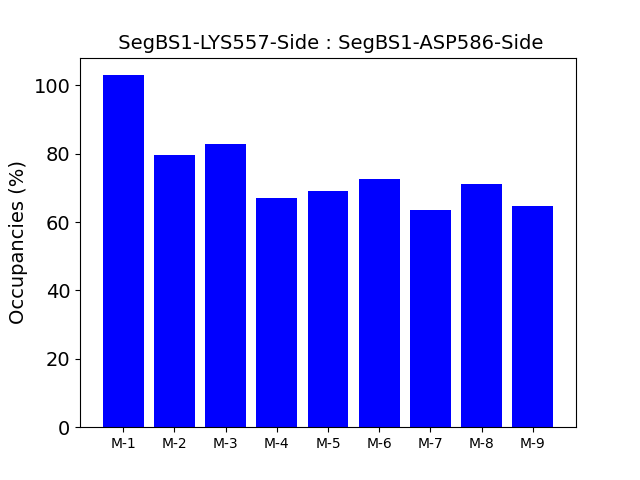

Supplement: SC-015-D4SC04364B-s001 [file SC-015-D4SC04364B-s001.zip › Inner_h_bonds_states/closed/SegBS1-LYS557-Side_SegBS1-ASP586-Side.png]

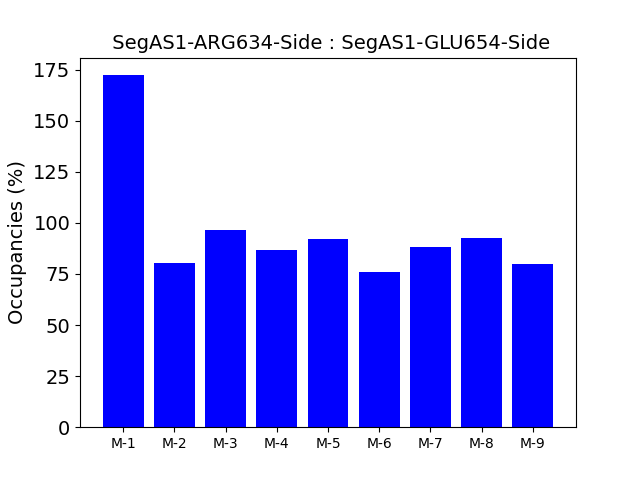

Supplement: SC-015-D4SC04364B-s001 [file SC-015-D4SC04364B-s001.zip › Inner_h_bonds_states/closed/SegAS1-ARG634-Side_SegAS1-GLU654-Side.png]

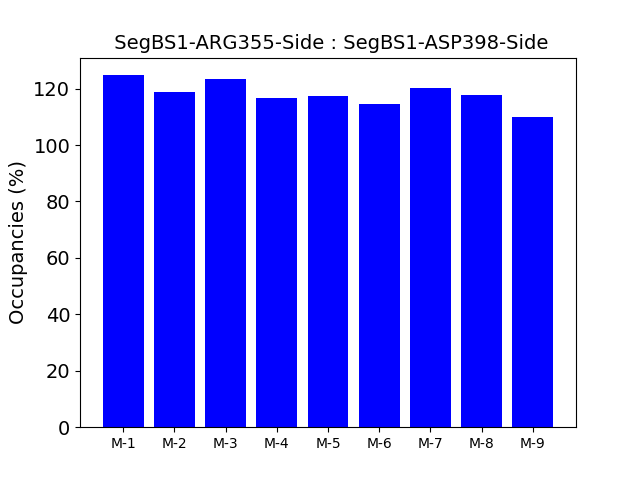

Supplement: SC-015-D4SC04364B-s001 [file SC-015-D4SC04364B-s001.zip › Inner_h_bonds_states/closed/SegBS1-ARG355-Side_SegBS1-ASP398-Side.png]

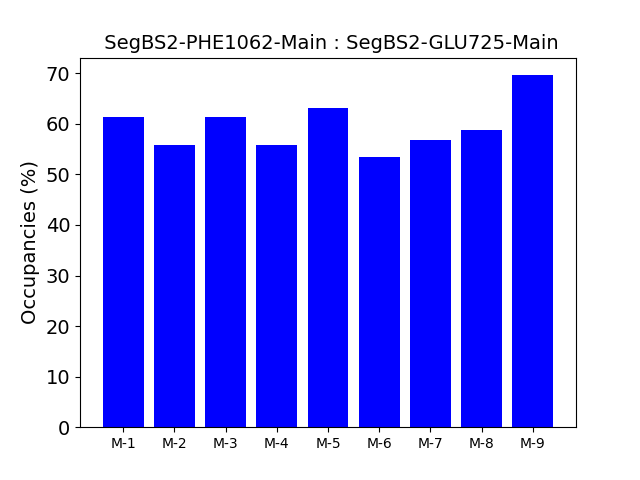

Supplement: SC-015-D4SC04364B-s001 [file SC-015-D4SC04364B-s001.zip › Inner_h_bonds_states/closed/SegBS2-PHE1062-Main_SegBS2-GLU725-Main.png]

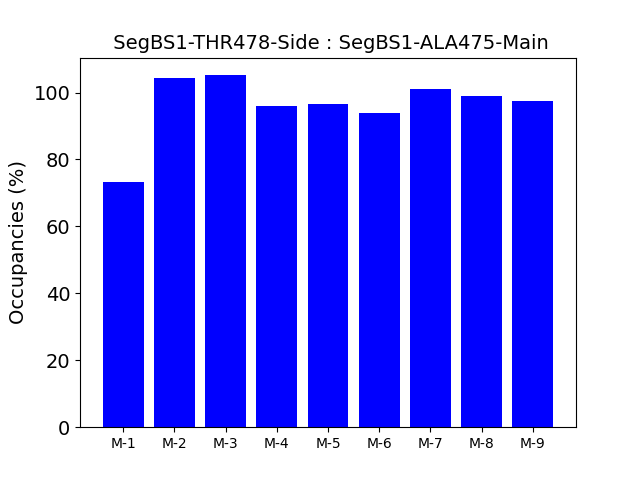

Supplement: SC-015-D4SC04364B-s001 [file SC-015-D4SC04364B-s001.zip › Inner_h_bonds_states/closed/SegBS1-THR478-Side_SegBS1-ALA475-Main.png]

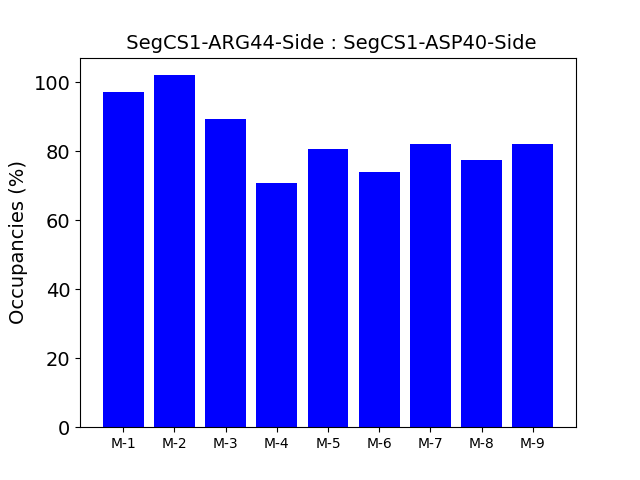

Supplement: SC-015-D4SC04364B-s001 [file SC-015-D4SC04364B-s001.zip › Inner_h_bonds_states/closed/SegCS1-ARG44-Side_SegCS1-ASP40-Side.png]

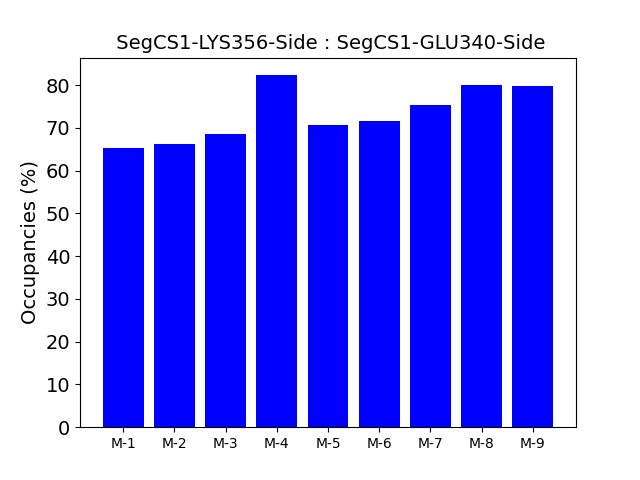

Supplement: SC-015-D4SC04364B-s001 [file SC-015-D4SC04364B-s001.zip › Inner_h_bonds_states/closed/SegCS1-LYS356-Side_SegCS1-GLU340-Side.png]

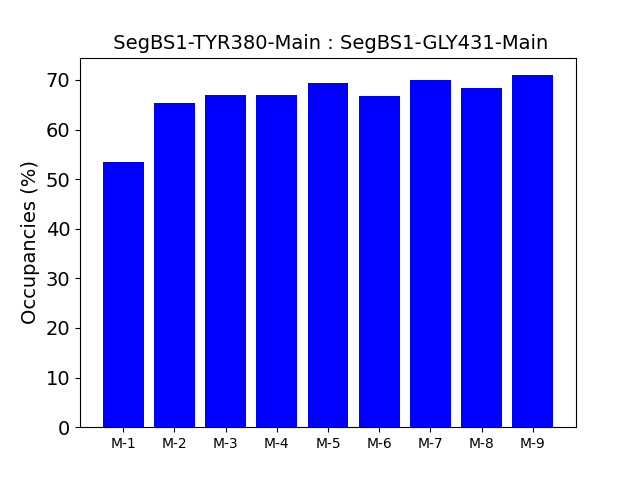

Supplement: SC-015-D4SC04364B-s001 [file SC-015-D4SC04364B-s001.zip › Inner_h_bonds_states/closed/SegBS1-TYR380-Main_SegBS1-GLY431-Main.png]

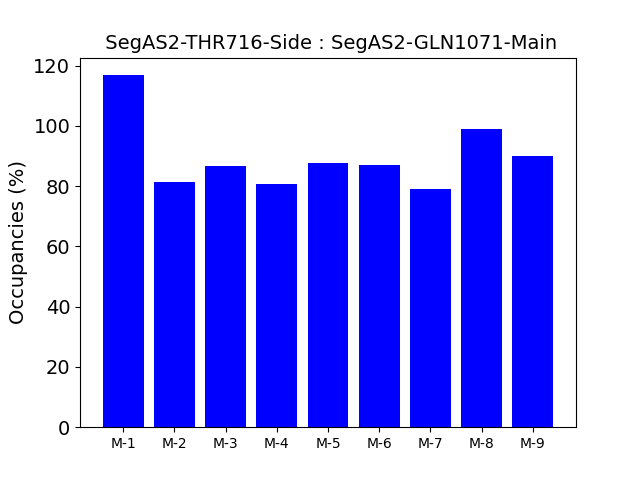

Supplement: SC-015-D4SC04364B-s001 [file SC-015-D4SC04364B-s001.zip › Inner_h_bonds_states/closed/SegAS2-THR716-Side_SegAS2-GLN1071-Main.png]

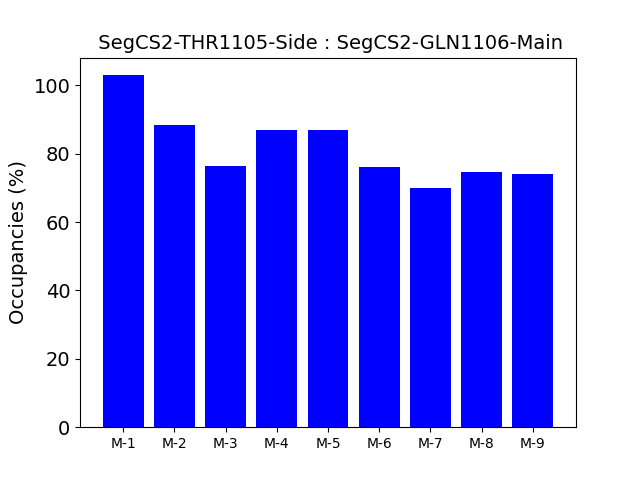

Supplement: SC-015-D4SC04364B-s001 [file SC-015-D4SC04364B-s001.zip › Inner_h_bonds_states/closed/SegCS2-THR1105-Side_SegCS2-GLN1106-Main.png]

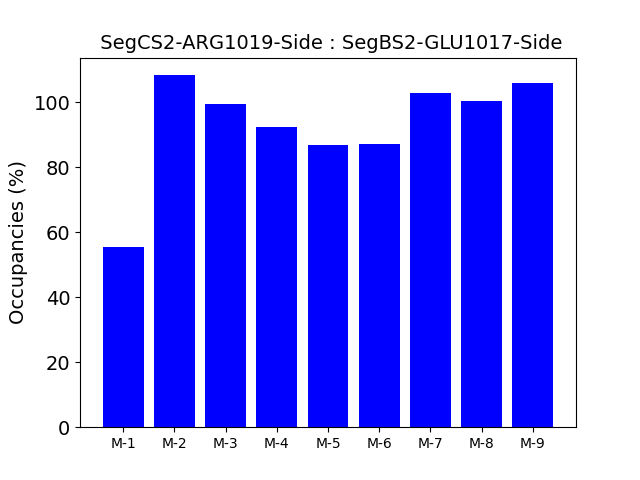

Supplement: SC-015-D4SC04364B-s001 [file SC-015-D4SC04364B-s001.zip › Inner_h_bonds_states/closed/SegCS2-ARG1019-Side_SegBS2-GLU1017-Side.png]

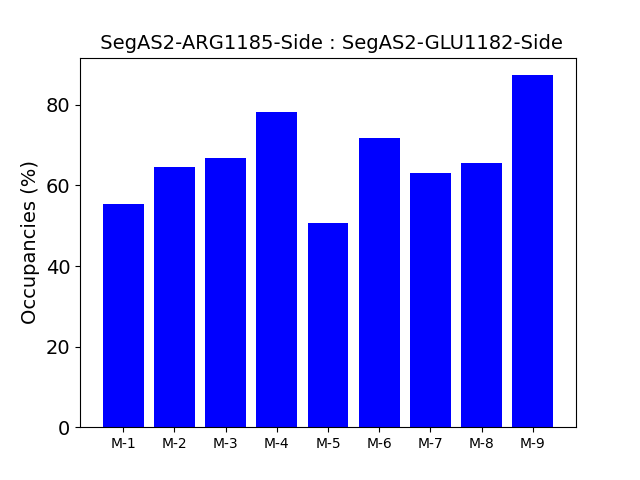

Supplement: SC-015-D4SC04364B-s001 [file SC-015-D4SC04364B-s001.zip › Inner_h_bonds_states/closed/SegAS2-ARG1185-Side_SegAS2-GLU1182-Side.png]

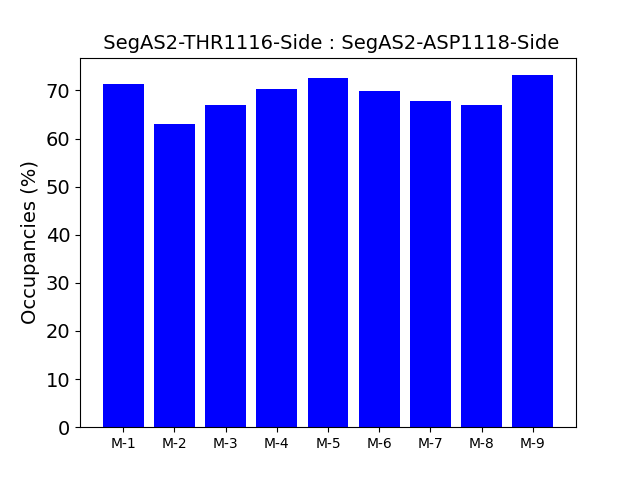

Supplement: SC-015-D4SC04364B-s001 [file SC-015-D4SC04364B-s001.zip › Inner_h_bonds_states/closed/SegAS2-THR1116-Side_SegAS2-ASP1118-Side.png]

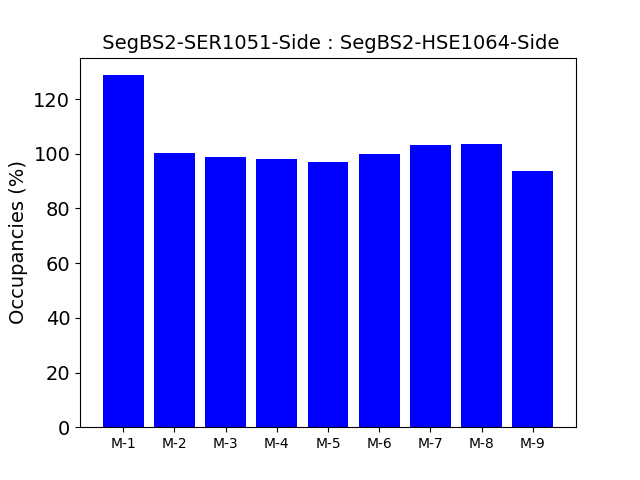

Supplement: SC-015-D4SC04364B-s001 [file SC-015-D4SC04364B-s001.zip › Inner_h_bonds_states/closed/SegBS2-SER1051-Side_SegBS2-HSE1064-Side.png]

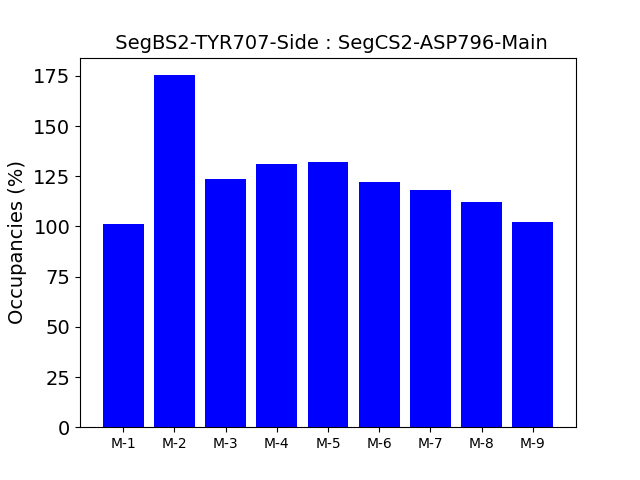

Supplement: SC-015-D4SC04364B-s001 [file SC-015-D4SC04364B-s001.zip › Inner_h_bonds_states/closed/SegBS2-TYR707-Side_SegCS2-ASP796-Main.png]

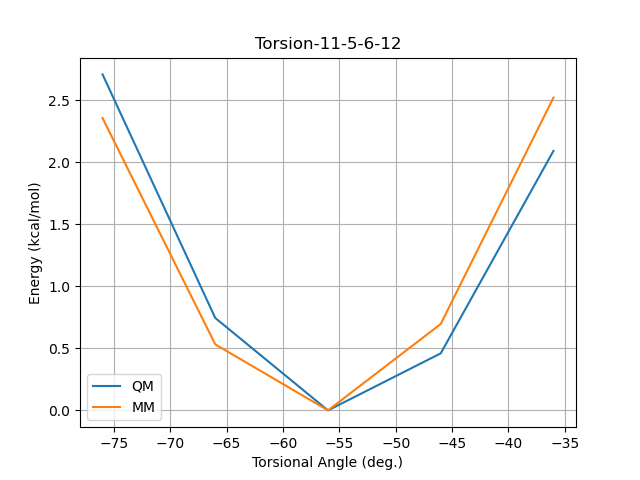

Supplement: SC-015-D4SC04364B-s002 [file SC-015-D4SC04364B-s002.zip › torsion_fit/AFUC/torsion_fitting-11-5-6-12.png]

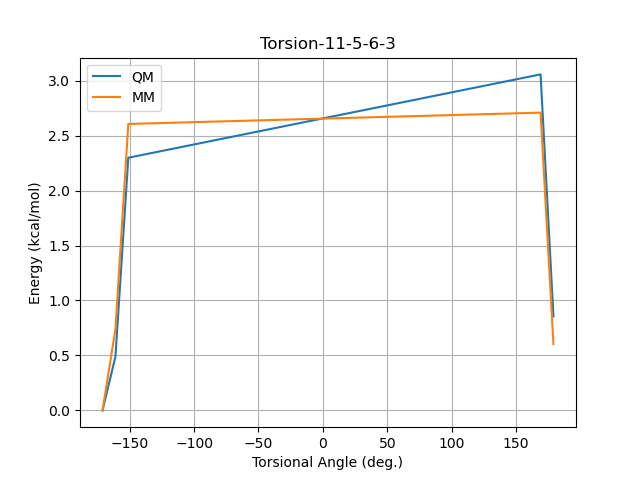

Supplement: SC-015-D4SC04364B-s002 [file SC-015-D4SC04364B-s002.zip › torsion_fit/AFUC/torsion_fitting-11-5-6-3.png]

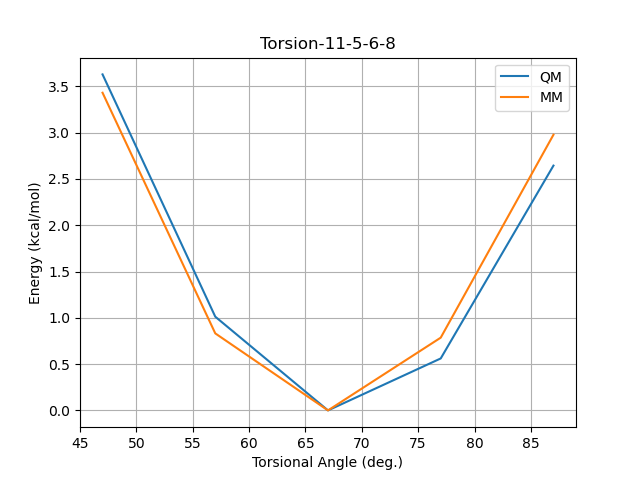

Supplement: SC-015-D4SC04364B-s002 [file SC-015-D4SC04364B-s002.zip › torsion_fit/AFUC/torsion_fitting-11-5-6-8.png]

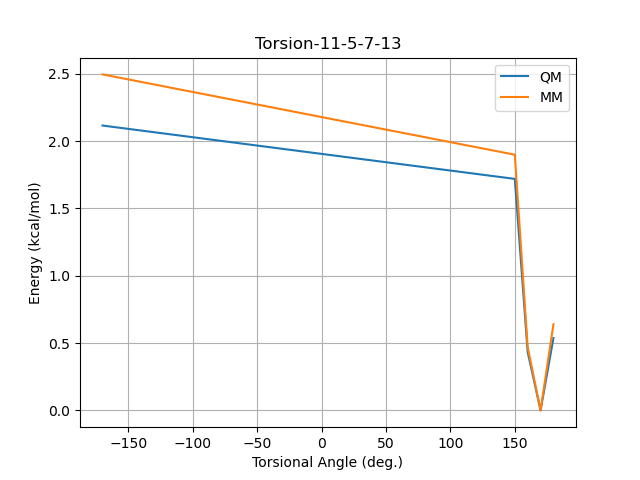

Supplement: SC-015-D4SC04364B-s002 [file SC-015-D4SC04364B-s002.zip › torsion_fit/AFUC/torsion_fitting-11-5-7-13.png]

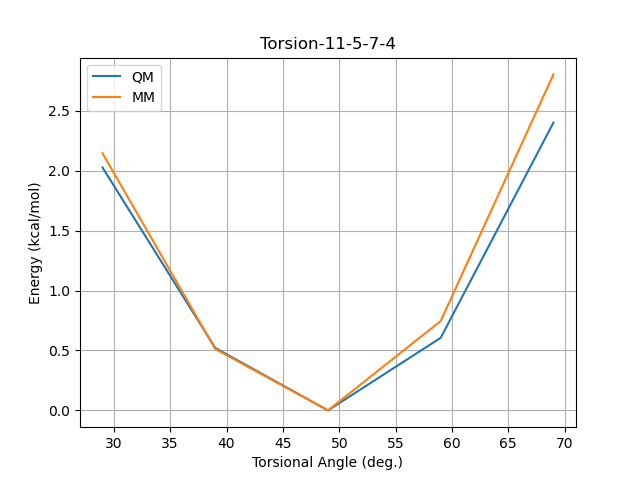

Supplement: SC-015-D4SC04364B-s002 [file SC-015-D4SC04364B-s002.zip › torsion_fit/AFUC/torsion_fitting-11-5-7-4.png]

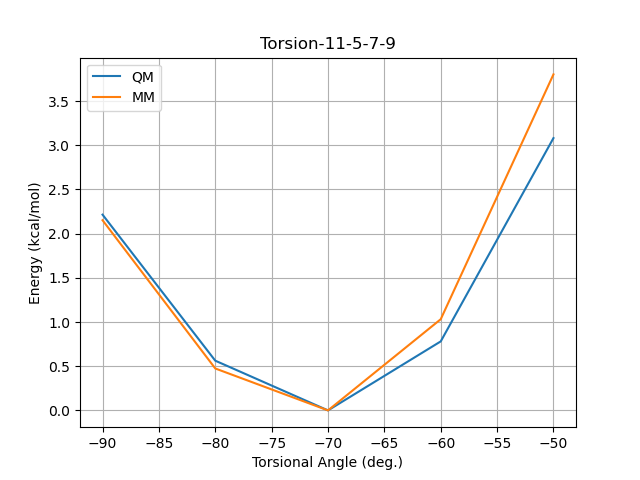

Supplement: SC-015-D4SC04364B-s002 [file SC-015-D4SC04364B-s002.zip › torsion_fit/AFUC/torsion_fitting-11-5-7-9.png]

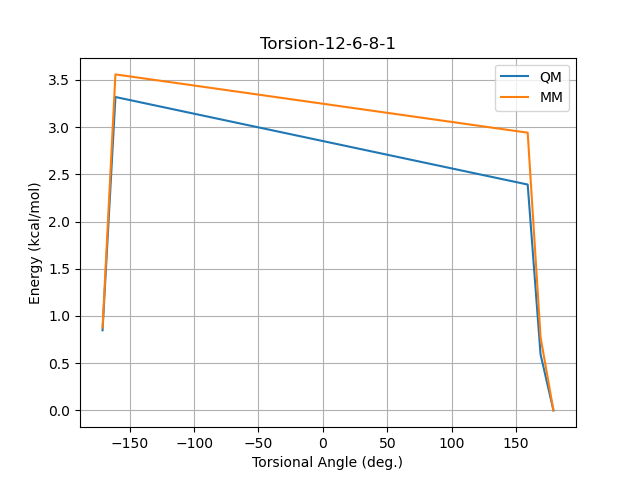

Supplement: SC-015-D4SC04364B-s002 [file SC-015-D4SC04364B-s002.zip › torsion_fit/AFUC/torsion_fitting-12-6-8-1.png]

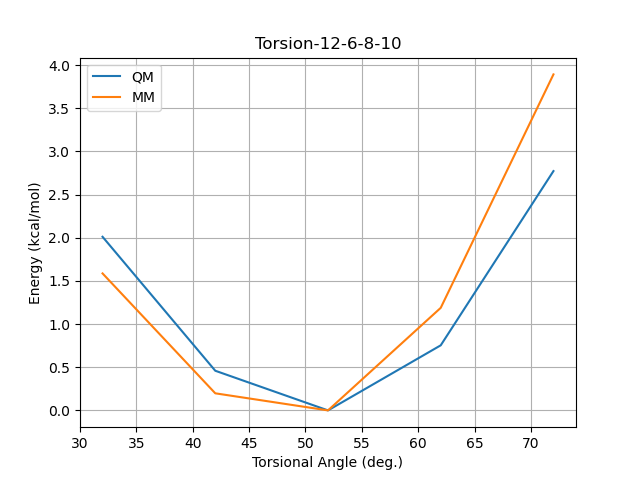

Supplement: SC-015-D4SC04364B-s002 [file SC-015-D4SC04364B-s002.zip › torsion_fit/AFUC/torsion_fitting-12-6-8-10.png]

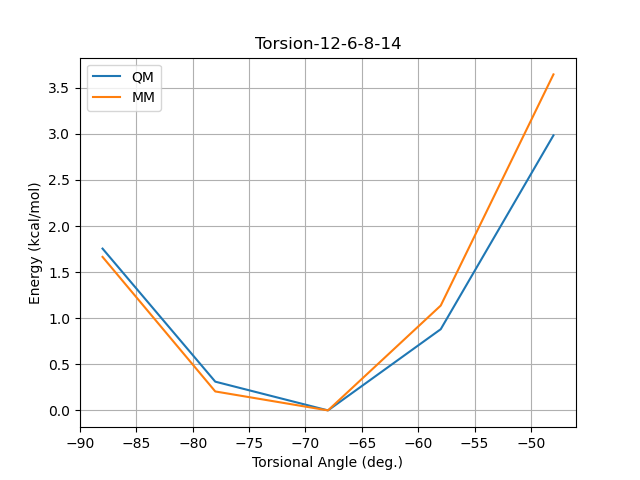

Supplement: SC-015-D4SC04364B-s002 [file SC-015-D4SC04364B-s002.zip › torsion_fit/AFUC/torsion_fitting-12-6-8-14.png]

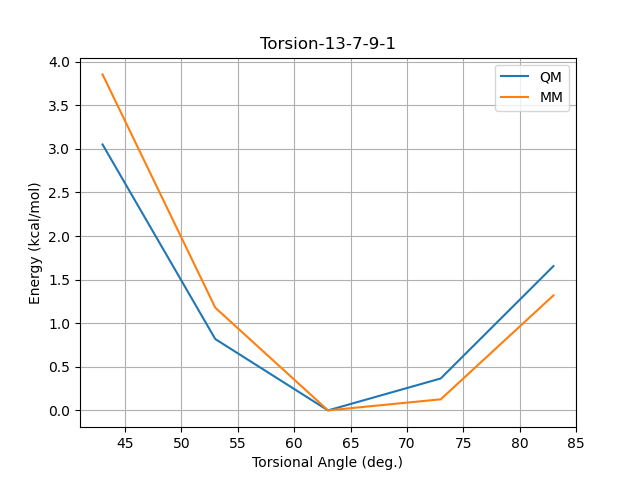

Supplement: SC-015-D4SC04364B-s002 [file SC-015-D4SC04364B-s002.zip › torsion_fit/AFUC/torsion_fitting-13-7-9-1.png]

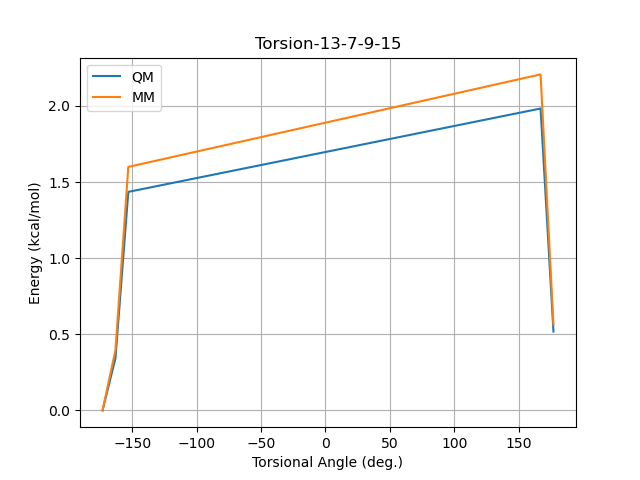

Supplement: SC-015-D4SC04364B-s002 [file SC-015-D4SC04364B-s002.zip › torsion_fit/AFUC/torsion_fitting-13-7-9-15.png]

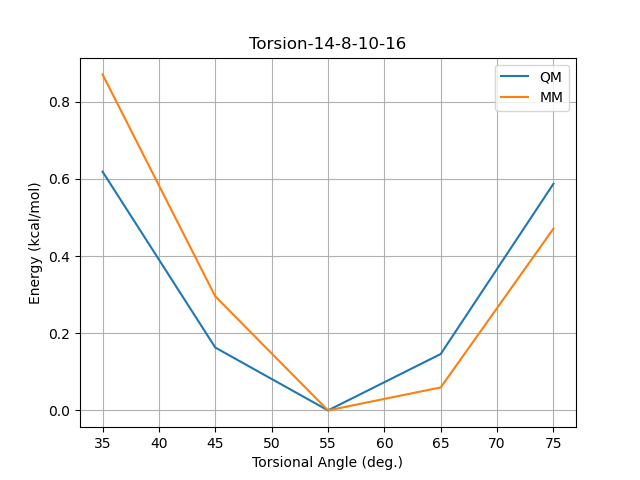

Supplement: SC-015-D4SC04364B-s002 [file SC-015-D4SC04364B-s002.zip › torsion_fit/AFUC/torsion_fitting-14-8-10-16.png]

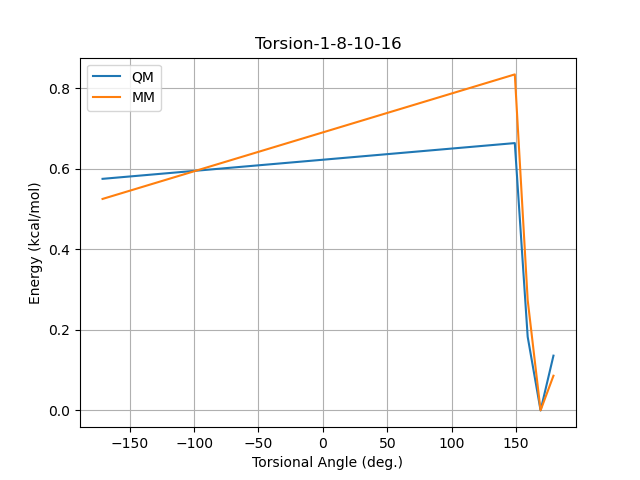

Supplement: SC-015-D4SC04364B-s002 [file SC-015-D4SC04364B-s002.zip › torsion_fit/AFUC/torsion_fitting-1-8-10-16.png]

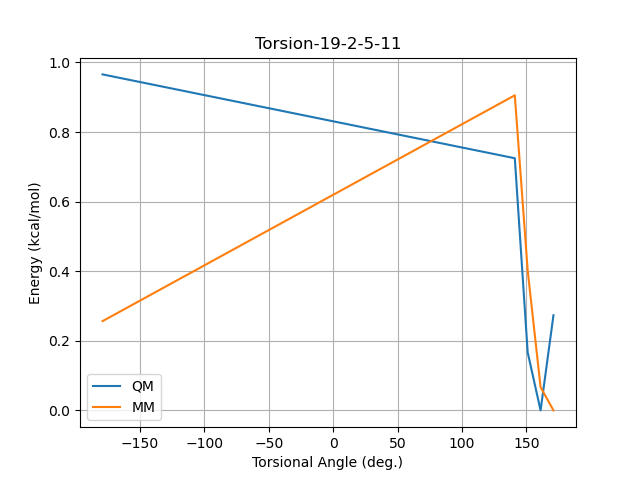

Supplement: SC-015-D4SC04364B-s002 [file SC-015-D4SC04364B-s002.zip › torsion_fit/AFUC/torsion_fitting-19-2-5-11.png]

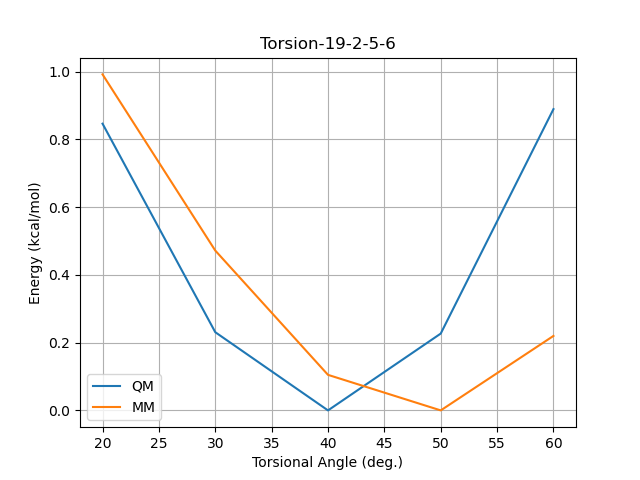

Supplement: SC-015-D4SC04364B-s002 [file SC-015-D4SC04364B-s002.zip › torsion_fit/AFUC/torsion_fitting-19-2-5-6.png]

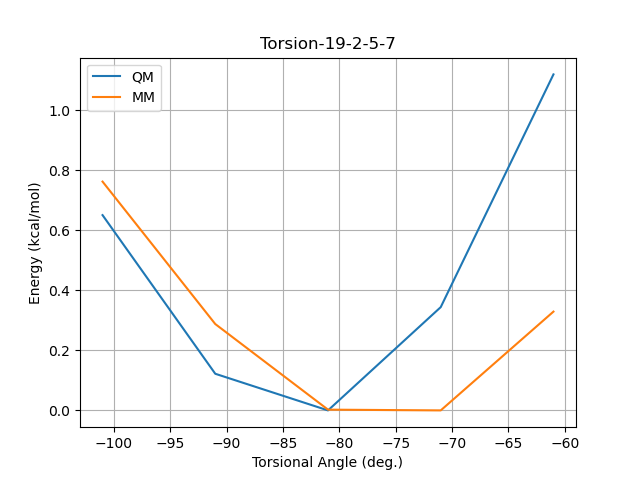

Supplement: SC-015-D4SC04364B-s002 [file SC-015-D4SC04364B-s002.zip › torsion_fit/AFUC/torsion_fitting-19-2-5-7.png]

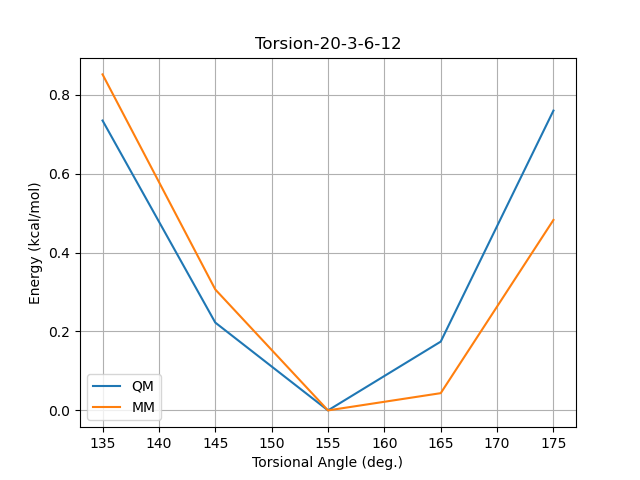

Supplement: SC-015-D4SC04364B-s002 [file SC-015-D4SC04364B-s002.zip › torsion_fit/AFUC/torsion_fitting-20-3-6-12.png]

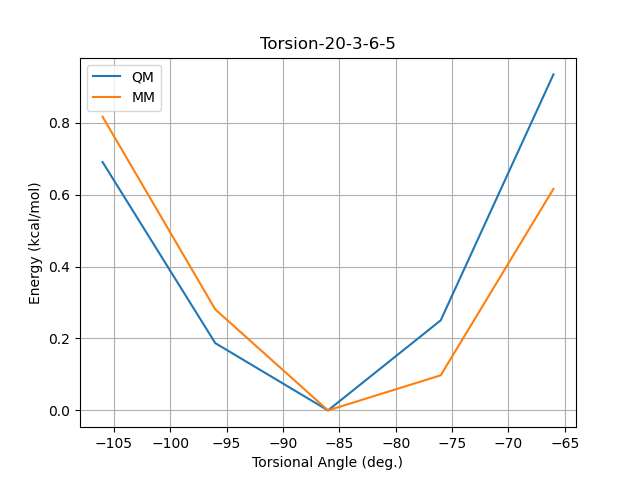

Supplement: SC-015-D4SC04364B-s002 [file SC-015-D4SC04364B-s002.zip › torsion_fit/AFUC/torsion_fitting-20-3-6-5.png]

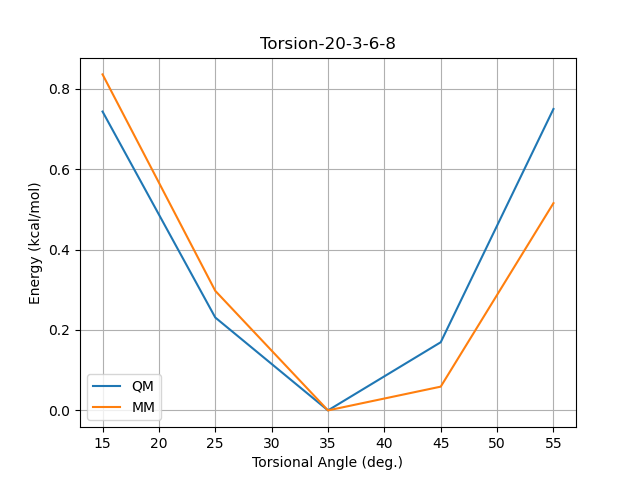

Supplement: SC-015-D4SC04364B-s002 [file SC-015-D4SC04364B-s002.zip › torsion_fit/AFUC/torsion_fitting-20-3-6-8.png]

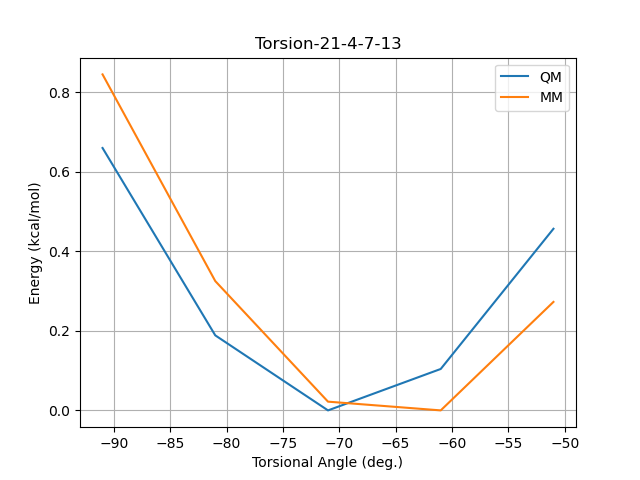

Supplement: SC-015-D4SC04364B-s002 [file SC-015-D4SC04364B-s002.zip › torsion_fit/AFUC/torsion_fitting-21-4-7-13.png]

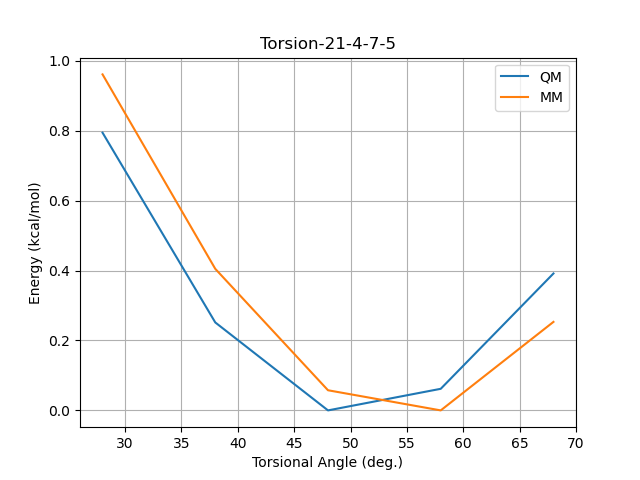

Supplement: SC-015-D4SC04364B-s002 [file SC-015-D4SC04364B-s002.zip › torsion_fit/AFUC/torsion_fitting-21-4-7-5.png]

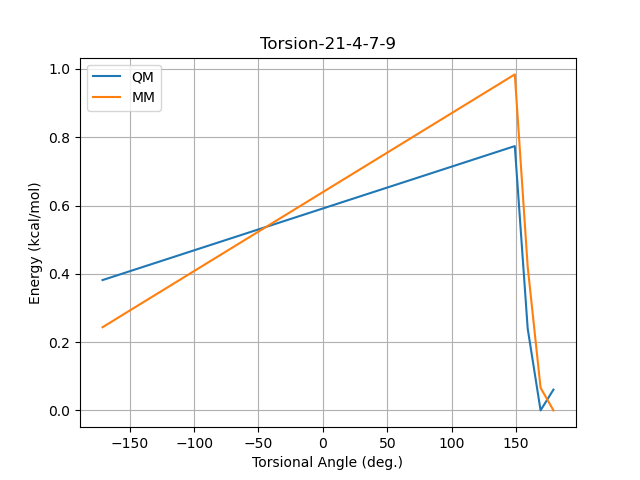

Supplement: SC-015-D4SC04364B-s002 [file SC-015-D4SC04364B-s002.zip › torsion_fit/AFUC/torsion_fitting-21-4-7-9.png]

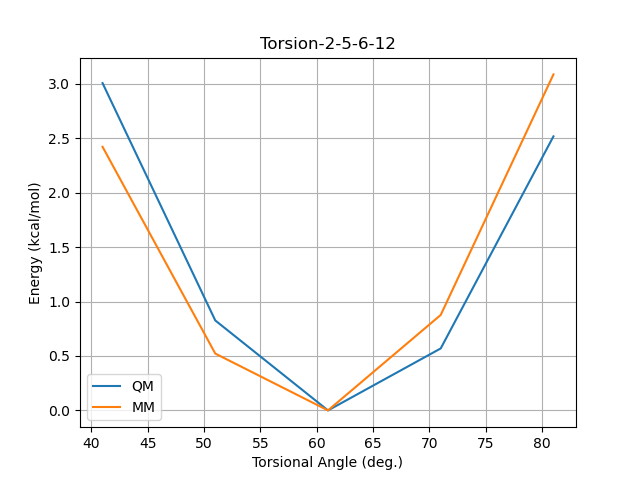

Supplement: SC-015-D4SC04364B-s002 [file SC-015-D4SC04364B-s002.zip › torsion_fit/AFUC/torsion_fitting-2-5-6-12.png]

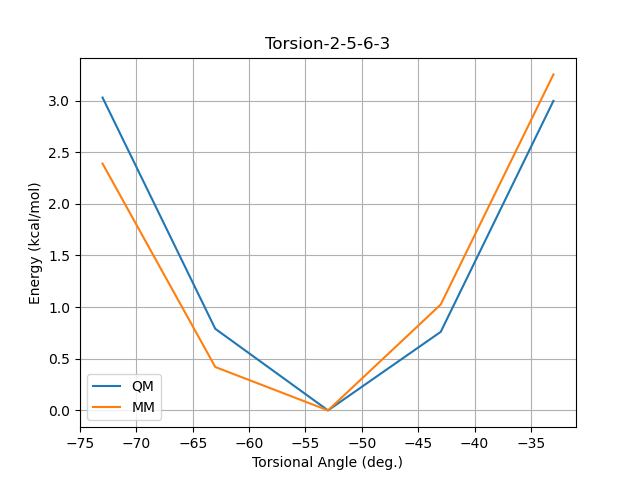

Supplement: SC-015-D4SC04364B-s002 [file SC-015-D4SC04364B-s002.zip › torsion_fit/AFUC/torsion_fitting-2-5-6-3.png]
