# Supplementary figures and images for: Water–glycan interactions drive the SARS-CoV-2 spike dynamics: insights into glycan-gate control and camouflage mechanisms (part 1 of 4)
Source: Chem Sci. 2024 Aug 23;15(35):14177–87. doi: 10.1039/d4sc04364b (PMC11359970; doi:10.1039/d4sc04364b)

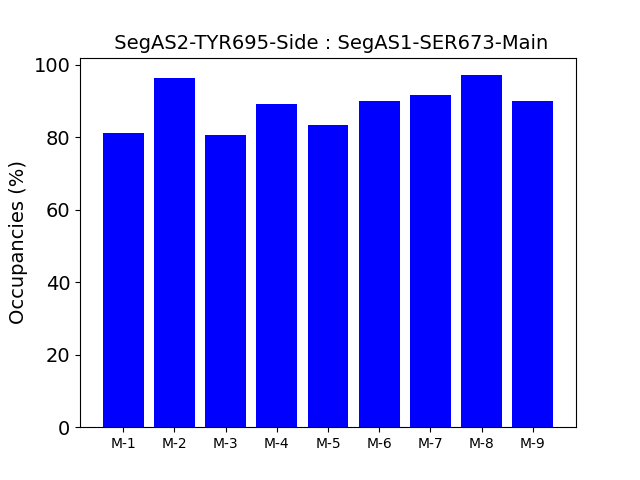

Supplement: SC-015-D4SC04364B-s001 [file SC-015-D4SC04364B-s001.zip › Inner_h_bonds_states/open/SegAS2-TYR695-Side_SegAS1-SER673-Main.png]

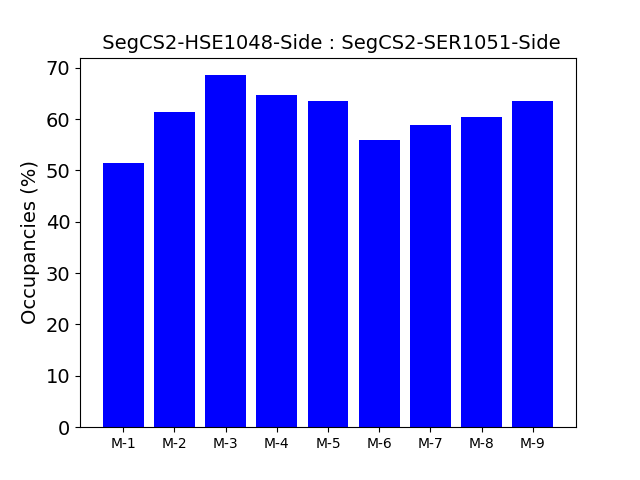

Supplement: SC-015-D4SC04364B-s001 [file SC-015-D4SC04364B-s001.zip › Inner_h_bonds_states/open/SegCS2-HSE1048-Side_SegCS2-SER1051-Side.png]

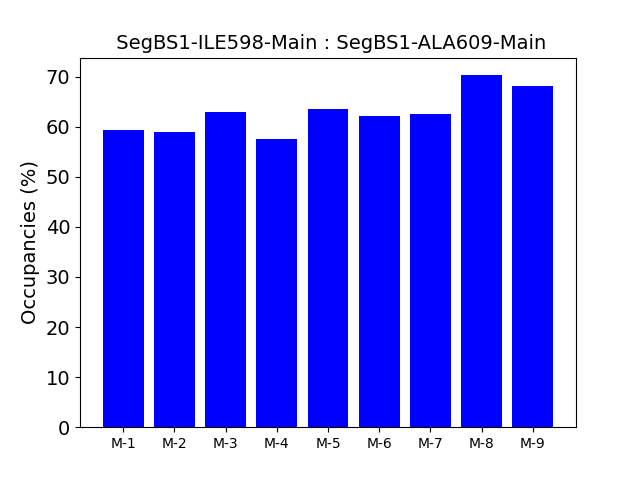

Supplement: SC-015-D4SC04364B-s001 [file SC-015-D4SC04364B-s001.zip › Inner_h_bonds_states/open/SegBS1-ILE598-Main_SegBS1-ALA609-Main.png]

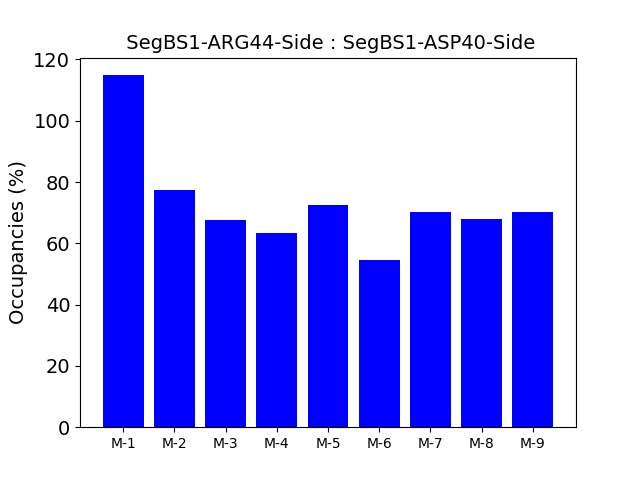

Supplement: SC-015-D4SC04364B-s001 [file SC-015-D4SC04364B-s001.zip › Inner_h_bonds_states/open/SegBS1-ARG44-Side_SegBS1-ASP40-Side.png]

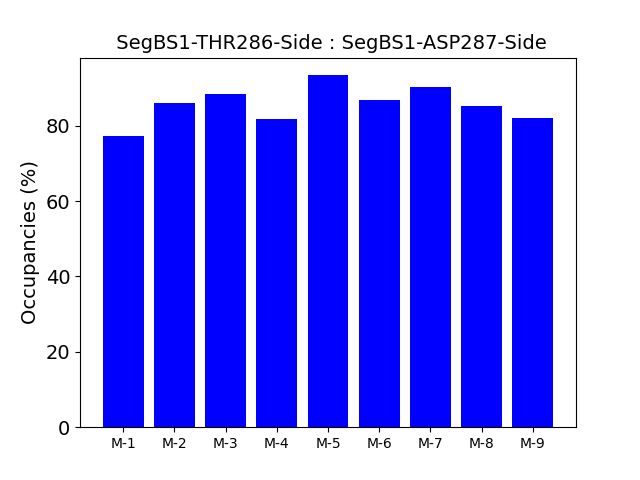

Supplement: SC-015-D4SC04364B-s001 [file SC-015-D4SC04364B-s001.zip › Inner_h_bonds_states/open/SegBS1-THR286-Side_SegBS1-ASP287-Side.png]

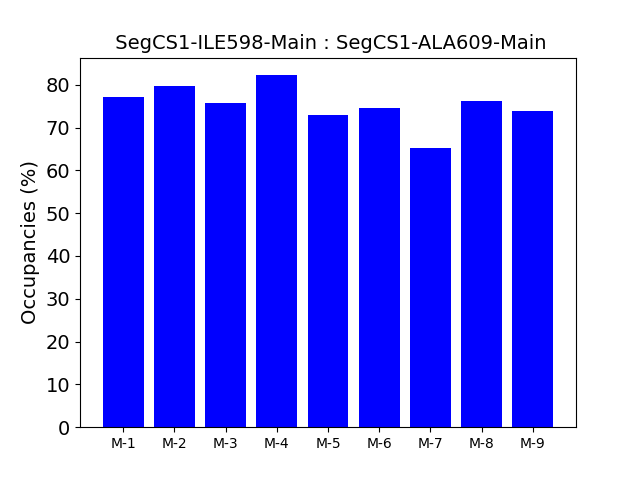

Supplement: SC-015-D4SC04364B-s001 [file SC-015-D4SC04364B-s001.zip › Inner_h_bonds_states/open/SegCS1-ILE598-Main_SegCS1-ALA609-Main.png]

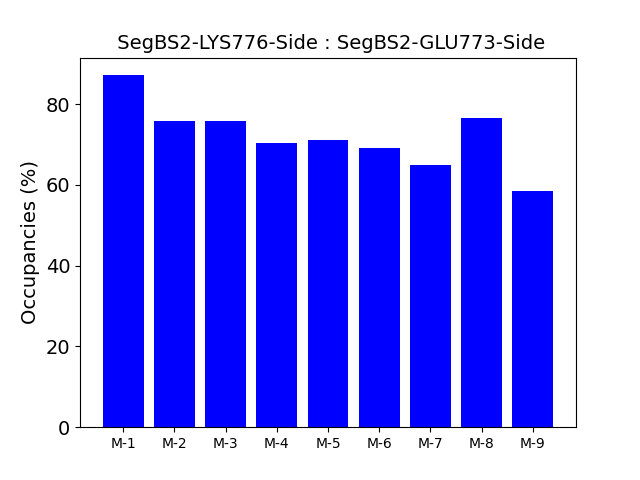

Supplement: SC-015-D4SC04364B-s001 [file SC-015-D4SC04364B-s001.zip › Inner_h_bonds_states/open/SegBS2-LYS776-Side_SegBS2-GLU773-Side.png]

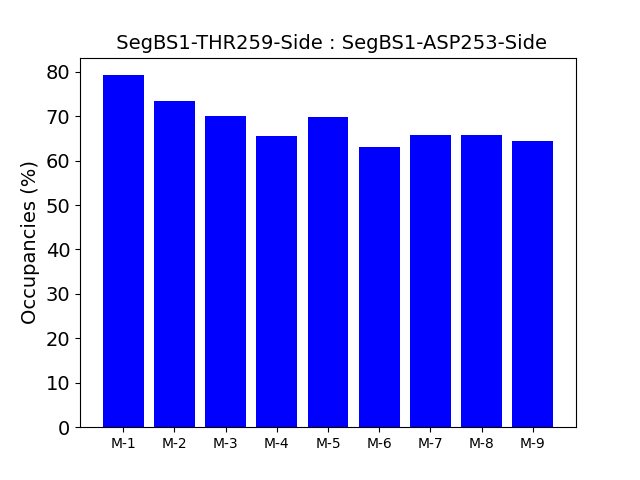

Supplement: SC-015-D4SC04364B-s001 [file SC-015-D4SC04364B-s001.zip › Inner_h_bonds_states/open/SegBS1-THR259-Side_SegBS1-ASP253-Side.png]

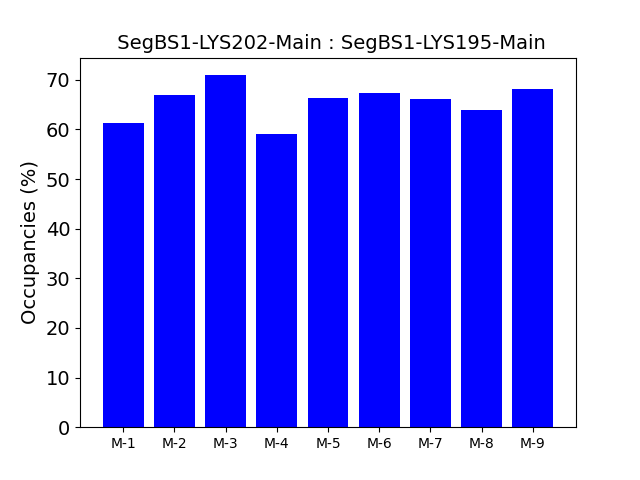

Supplement: SC-015-D4SC04364B-s001 [file SC-015-D4SC04364B-s001.zip › Inner_h_bonds_states/open/SegBS1-LYS202-Main_SegBS1-LYS195-Main.png]

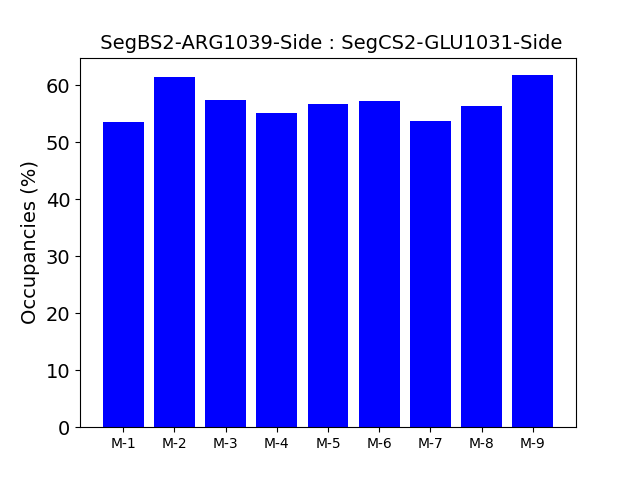

Supplement: SC-015-D4SC04364B-s001 [file SC-015-D4SC04364B-s001.zip › Inner_h_bonds_states/open/SegBS2-ARG1039-Side_SegCS2-GLU1031-Side.png]

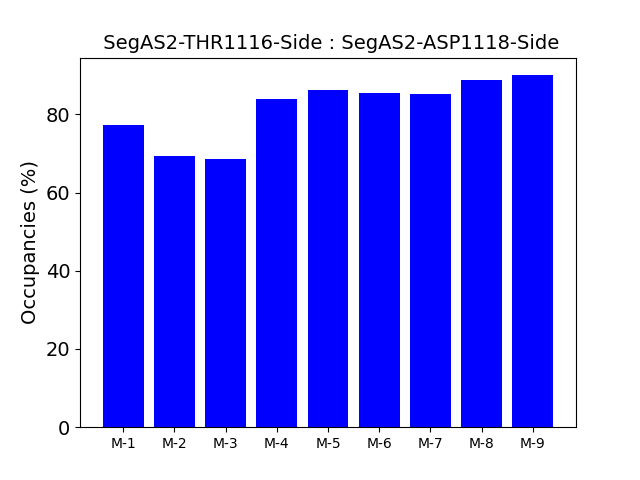

Supplement: SC-015-D4SC04364B-s001 [file SC-015-D4SC04364B-s001.zip › Inner_h_bonds_states/open/SegAS2-THR1116-Side_SegAS2-ASP1118-Side.png]

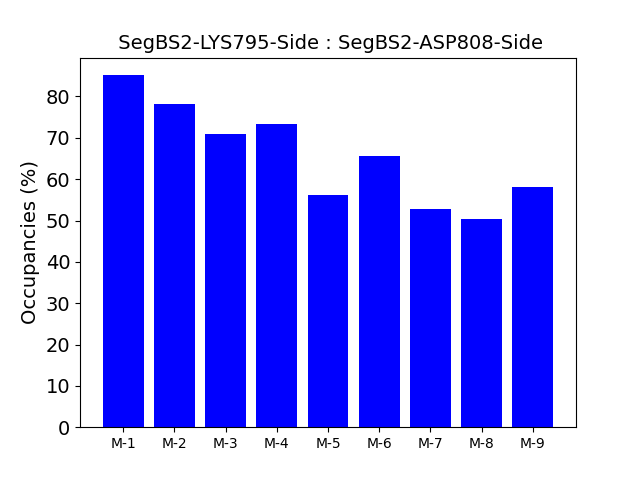

Supplement: SC-015-D4SC04364B-s001 [file SC-015-D4SC04364B-s001.zip › Inner_h_bonds_states/open/SegBS2-LYS795-Side_SegBS2-ASP808-Side.png]

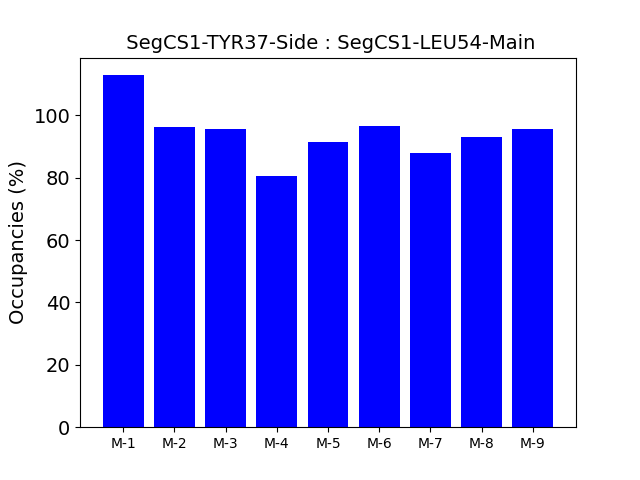

Supplement: SC-015-D4SC04364B-s001 [file SC-015-D4SC04364B-s001.zip › Inner_h_bonds_states/open/SegCS1-TYR37-Side_SegCS1-LEU54-Main.png]

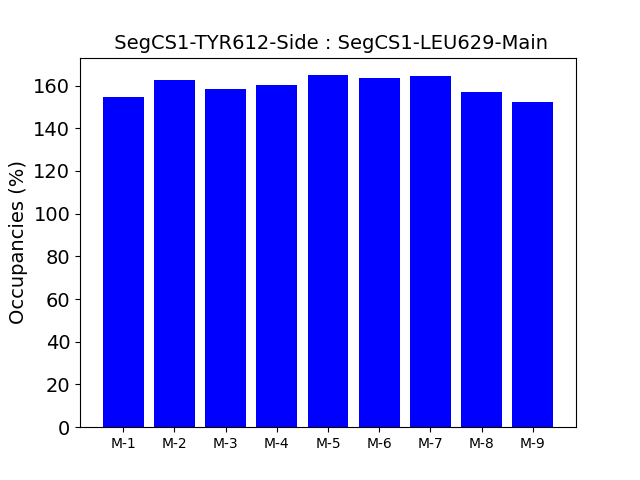

Supplement: SC-015-D4SC04364B-s001 [file SC-015-D4SC04364B-s001.zip › Inner_h_bonds_states/open/SegCS1-TYR612-Side_SegCS1-LEU629-Main.png]

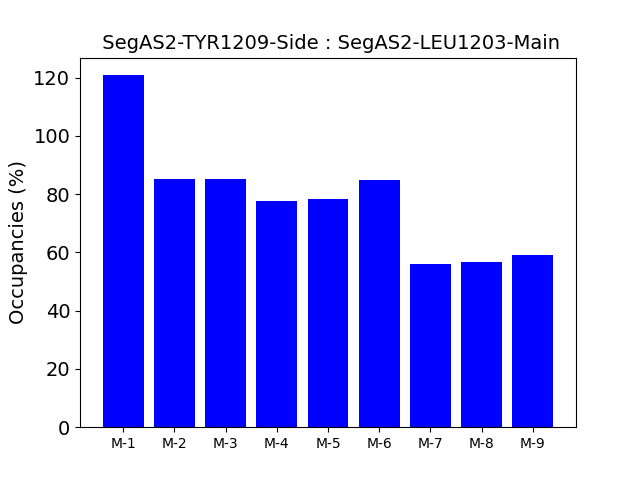

Supplement: SC-015-D4SC04364B-s001 [file SC-015-D4SC04364B-s001.zip › Inner_h_bonds_states/open/SegAS2-TYR1209-Side_SegAS2-LEU1203-Main.png]

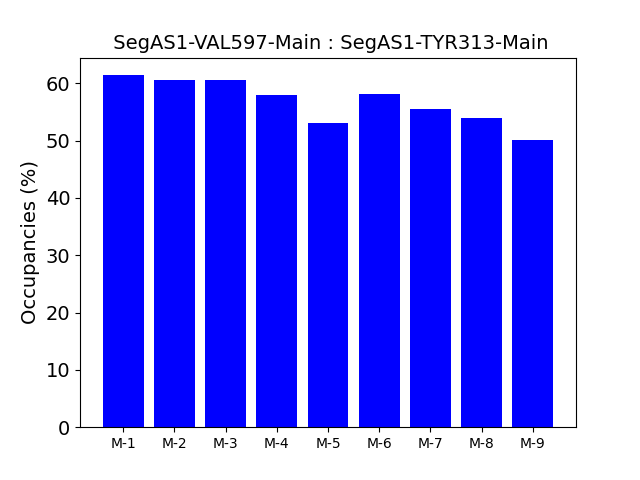

Supplement: SC-015-D4SC04364B-s001 [file SC-015-D4SC04364B-s001.zip › Inner_h_bonds_states/open/SegAS1-VAL597-Main_SegAS1-TYR313-Main.png]

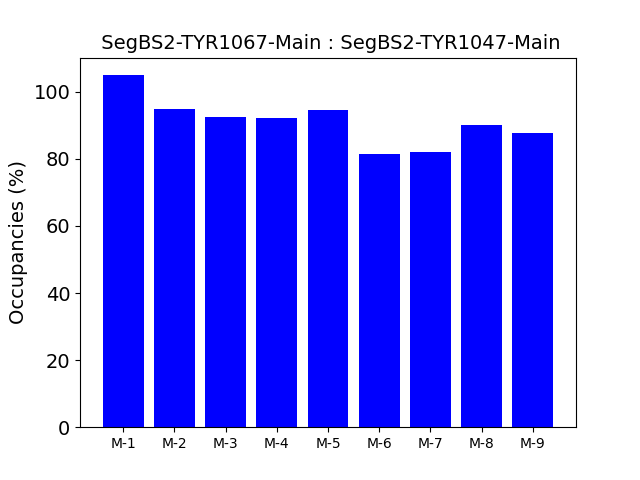

Supplement: SC-015-D4SC04364B-s001 [file SC-015-D4SC04364B-s001.zip › Inner_h_bonds_states/open/SegBS2-TYR1067-Main_SegBS2-TYR1047-Main.png]

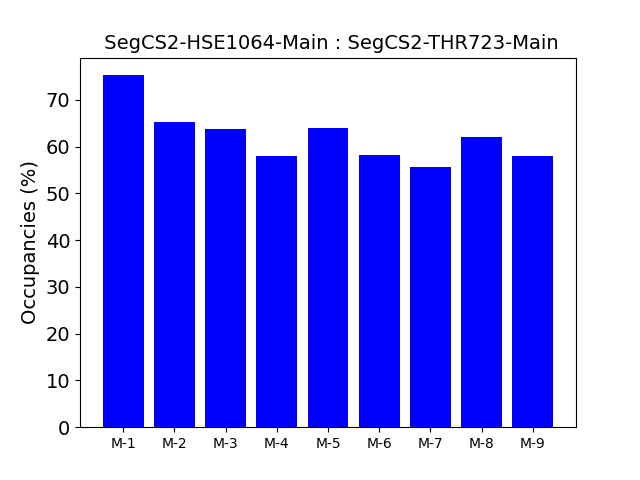

Supplement: SC-015-D4SC04364B-s001 [file SC-015-D4SC04364B-s001.zip › Inner_h_bonds_states/open/SegCS2-HSE1064-Main_SegCS2-THR723-Main.png]

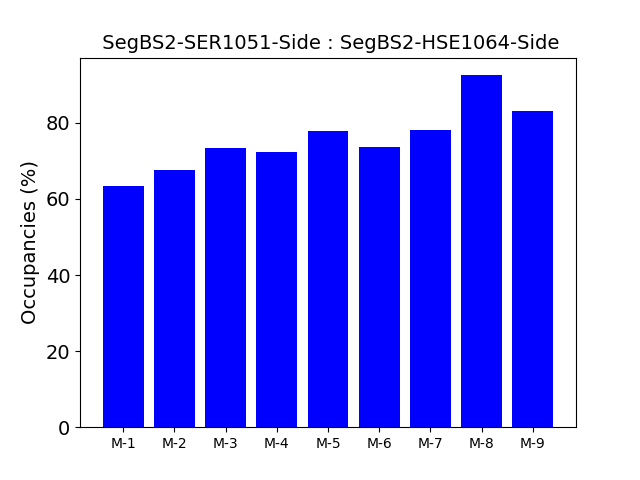

Supplement: SC-015-D4SC04364B-s001 [file SC-015-D4SC04364B-s001.zip › Inner_h_bonds_states/open/SegBS2-SER1051-Side_SegBS2-HSE1064-Side.png]

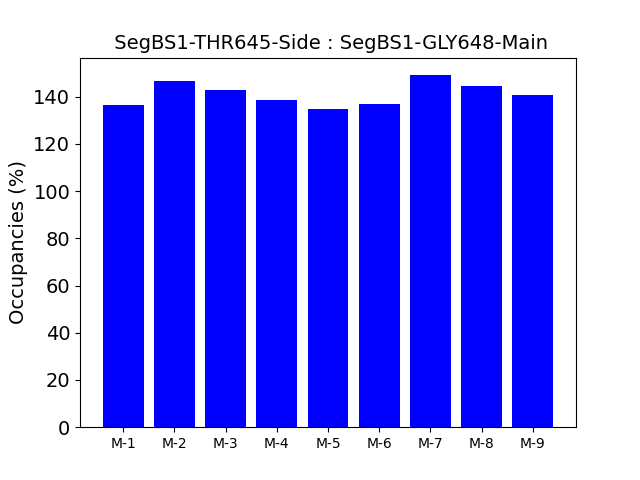

Supplement: SC-015-D4SC04364B-s001 [file SC-015-D4SC04364B-s001.zip › Inner_h_bonds_states/open/SegBS1-THR645-Side_SegBS1-GLY648-Main.png]

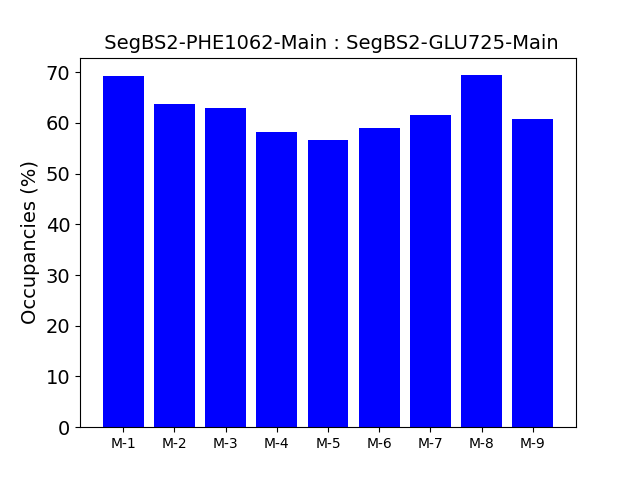

Supplement: SC-015-D4SC04364B-s001 [file SC-015-D4SC04364B-s001.zip › Inner_h_bonds_states/open/SegBS2-PHE1062-Main_SegBS2-GLU725-Main.png]

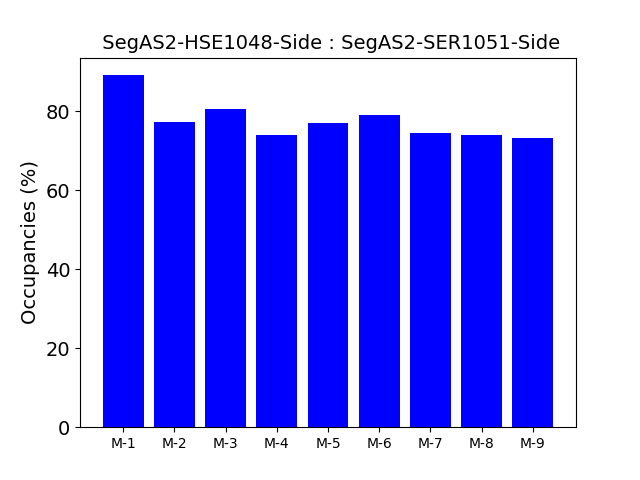

Supplement: SC-015-D4SC04364B-s001 [file SC-015-D4SC04364B-s001.zip › Inner_h_bonds_states/open/SegAS2-HSE1048-Side_SegAS2-SER1051-Side.png]

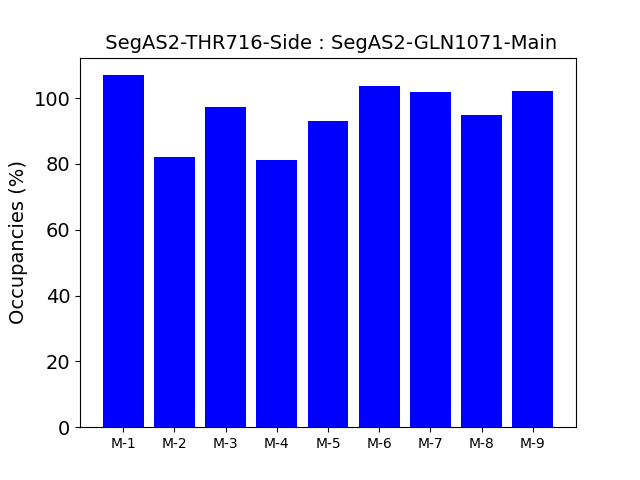

Supplement: SC-015-D4SC04364B-s001 [file SC-015-D4SC04364B-s001.zip › Inner_h_bonds_states/open/SegAS2-THR716-Side_SegAS2-GLN1071-Main.png]

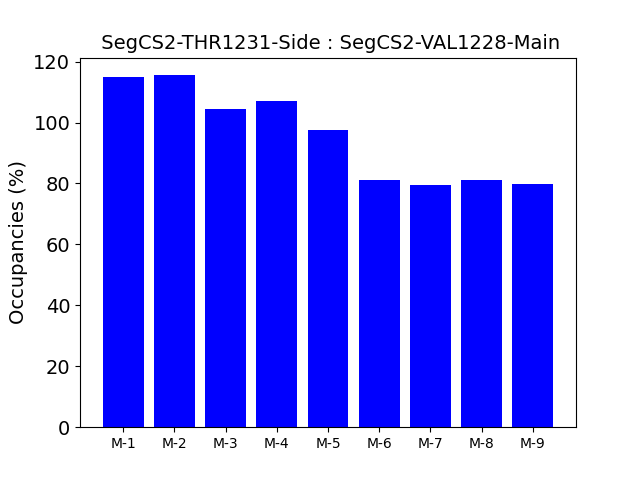

Supplement: SC-015-D4SC04364B-s001 [file SC-015-D4SC04364B-s001.zip › Inner_h_bonds_states/open/SegCS2-THR1231-Side_SegCS2-VAL1228-Main.png]

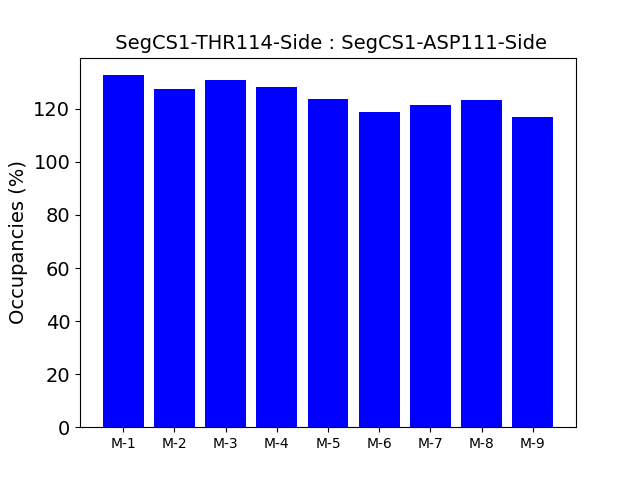

Supplement: SC-015-D4SC04364B-s001 [file SC-015-D4SC04364B-s001.zip › Inner_h_bonds_states/open/SegCS1-THR114-Side_SegCS1-ASP111-Side.png]

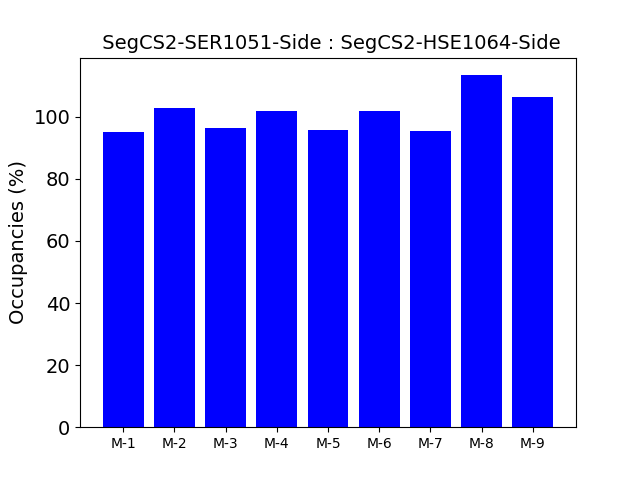

Supplement: SC-015-D4SC04364B-s001 [file SC-015-D4SC04364B-s001.zip › Inner_h_bonds_states/open/SegCS2-SER1051-Side_SegCS2-HSE1064-Side.png]

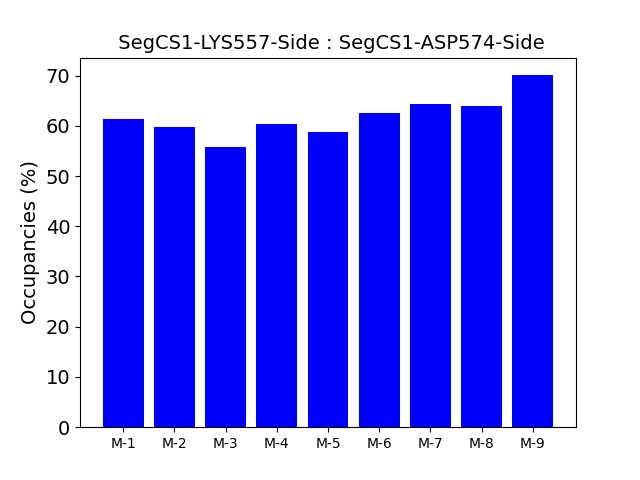

Supplement: SC-015-D4SC04364B-s001 [file SC-015-D4SC04364B-s001.zip › Inner_h_bonds_states/open/SegCS1-LYS557-Side_SegCS1-ASP574-Side.png]

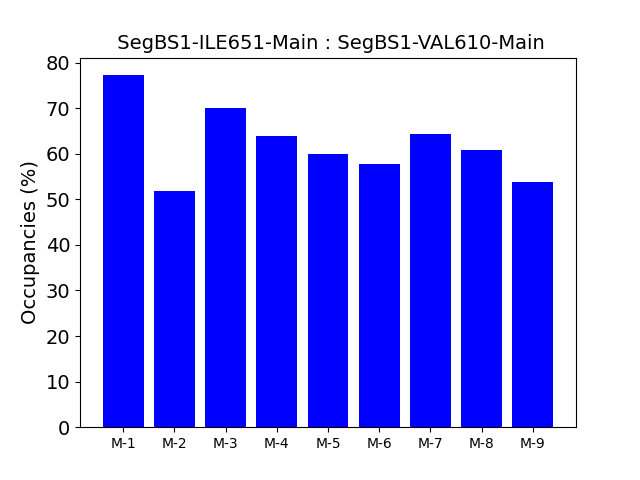

Supplement: SC-015-D4SC04364B-s001 [file SC-015-D4SC04364B-s001.zip › Inner_h_bonds_states/open/SegBS1-ILE651-Main_SegBS1-VAL610-Main.png]

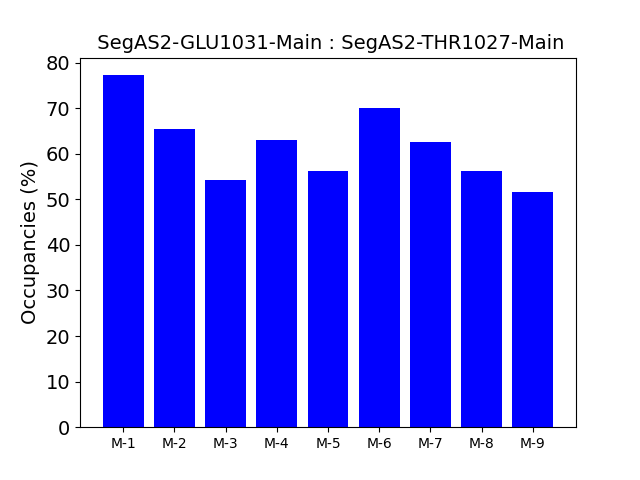

Supplement: SC-015-D4SC04364B-s001 [file SC-015-D4SC04364B-s001.zip › Inner_h_bonds_states/open/SegAS2-GLU1031-Main_SegAS2-THR1027-Main.png]

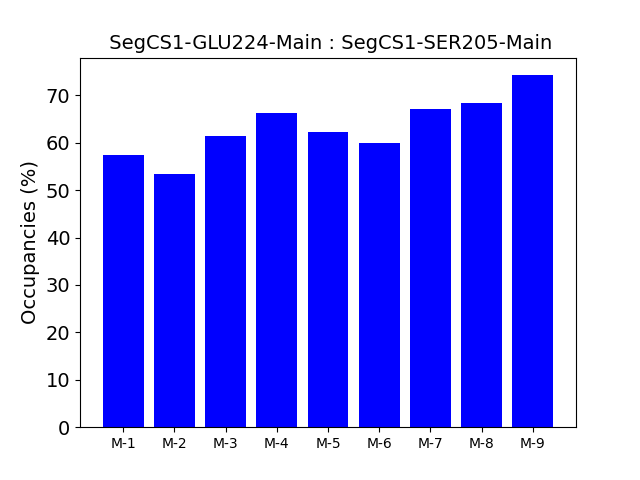

Supplement: SC-015-D4SC04364B-s001 [file SC-015-D4SC04364B-s001.zip › Inner_h_bonds_states/open/SegCS1-GLU224-Main_SegCS1-SER205-Main.png]

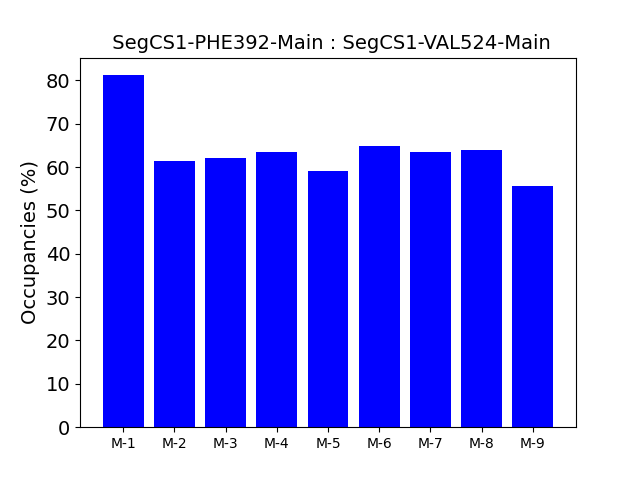

Supplement: SC-015-D4SC04364B-s001 [file SC-015-D4SC04364B-s001.zip › Inner_h_bonds_states/open/SegCS1-PHE392-Main_SegCS1-VAL524-Main.png]

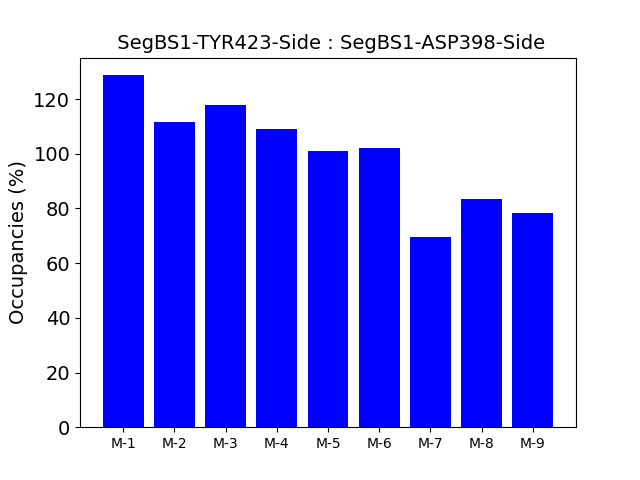

Supplement: SC-015-D4SC04364B-s001 [file SC-015-D4SC04364B-s001.zip › Inner_h_bonds_states/open/SegBS1-TYR423-Side_SegBS1-ASP398-Side.png]

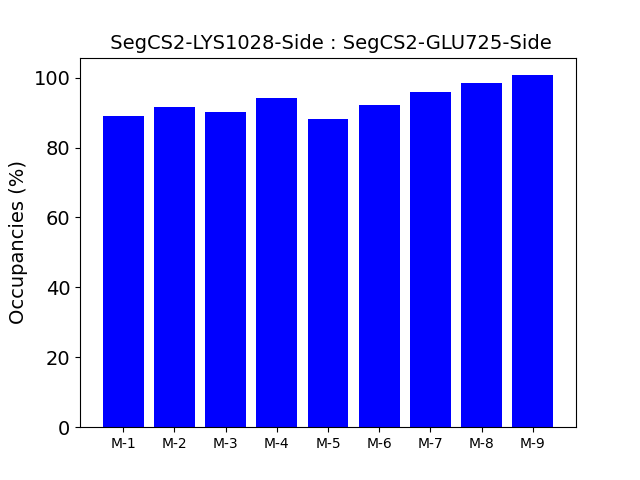

Supplement: SC-015-D4SC04364B-s001 [file SC-015-D4SC04364B-s001.zip › Inner_h_bonds_states/open/SegCS2-LYS1028-Side_SegCS2-GLU725-Side.png]

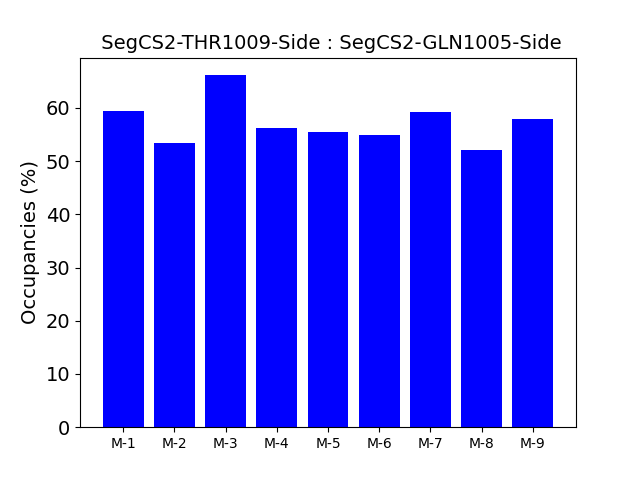

Supplement: SC-015-D4SC04364B-s001 [file SC-015-D4SC04364B-s001.zip › Inner_h_bonds_states/open/SegCS2-THR1009-Side_SegCS2-GLN1005-Side.png]

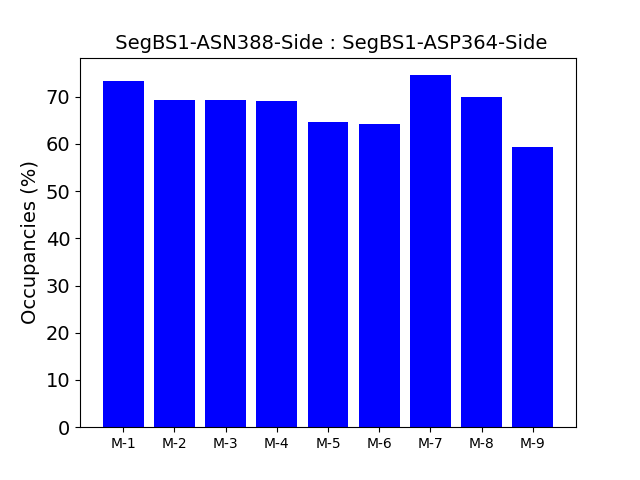

Supplement: SC-015-D4SC04364B-s001 [file SC-015-D4SC04364B-s001.zip › Inner_h_bonds_states/open/SegBS1-ASN388-Side_SegBS1-ASP364-Side.png]

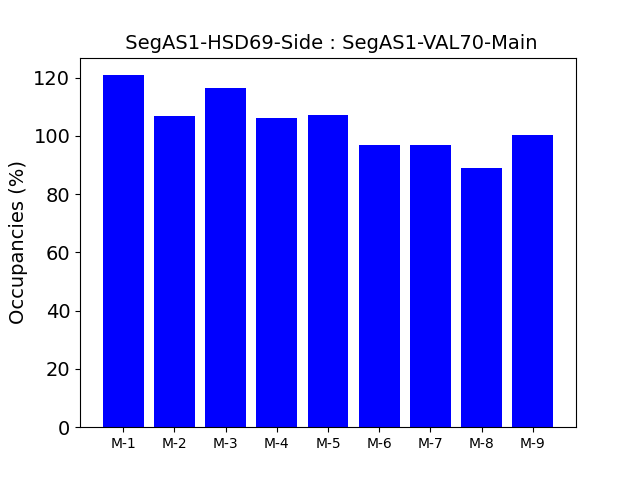

Supplement: SC-015-D4SC04364B-s001 [file SC-015-D4SC04364B-s001.zip › Inner_h_bonds_states/open/SegAS1-HSD69-Side_SegAS1-VAL70-Main.png]

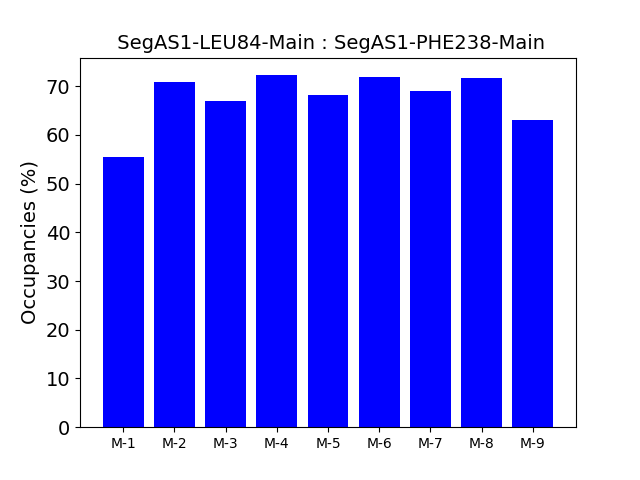

Supplement: SC-015-D4SC04364B-s001 [file SC-015-D4SC04364B-s001.zip › Inner_h_bonds_states/open/SegAS1-LEU84-Main_SegAS1-PHE238-Main.png]

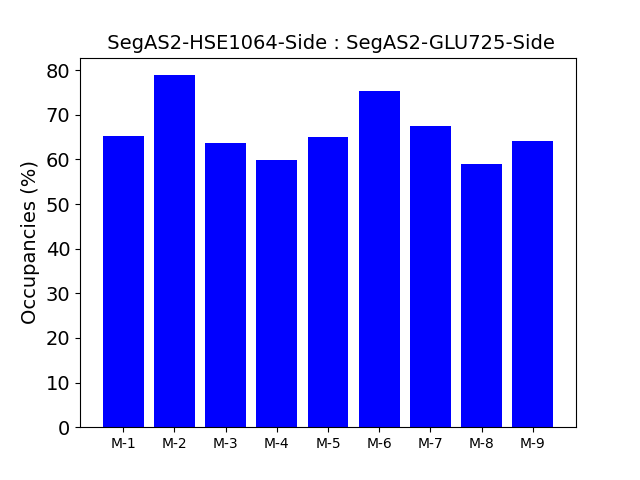

Supplement: SC-015-D4SC04364B-s001 [file SC-015-D4SC04364B-s001.zip › Inner_h_bonds_states/open/SegAS2-HSE1064-Side_SegAS2-GLU725-Side.png]

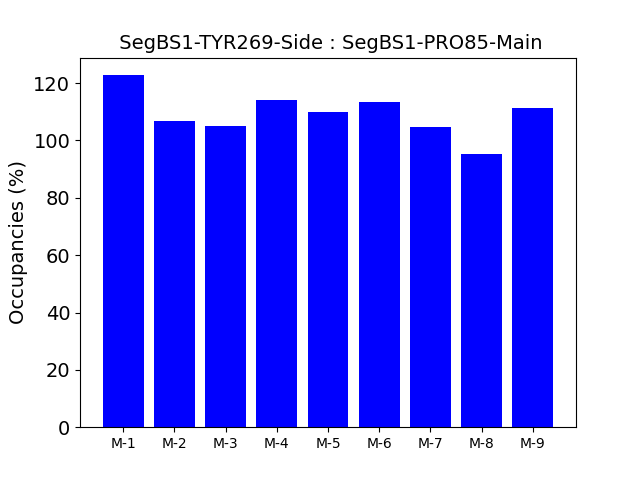

Supplement: SC-015-D4SC04364B-s001 [file SC-015-D4SC04364B-s001.zip › Inner_h_bonds_states/open/SegBS1-TYR269-Side_SegBS1-PRO85-Main.png]

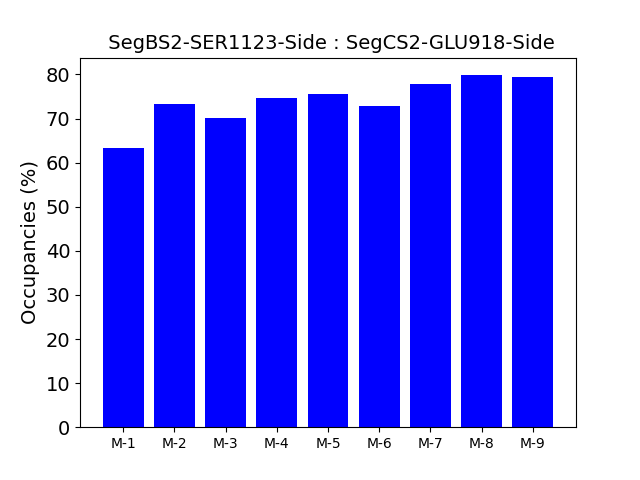

Supplement: SC-015-D4SC04364B-s001 [file SC-015-D4SC04364B-s001.zip › Inner_h_bonds_states/open/SegBS2-SER1123-Side_SegCS2-GLU918-Side.png]

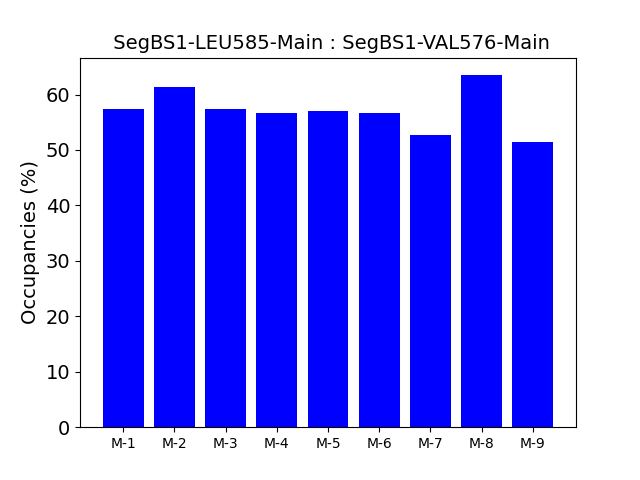

Supplement: SC-015-D4SC04364B-s001 [file SC-015-D4SC04364B-s001.zip › Inner_h_bonds_states/open/SegBS1-LEU585-Main_SegBS1-VAL576-Main.png]

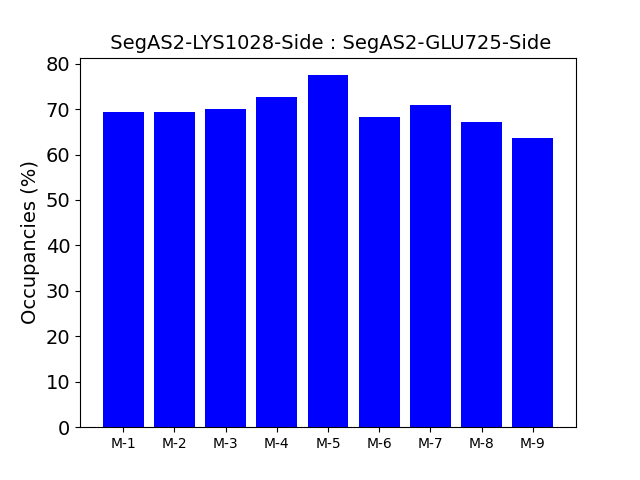

Supplement: SC-015-D4SC04364B-s001 [file SC-015-D4SC04364B-s001.zip › Inner_h_bonds_states/open/SegAS2-LYS1028-Side_SegAS2-GLU725-Side.png]

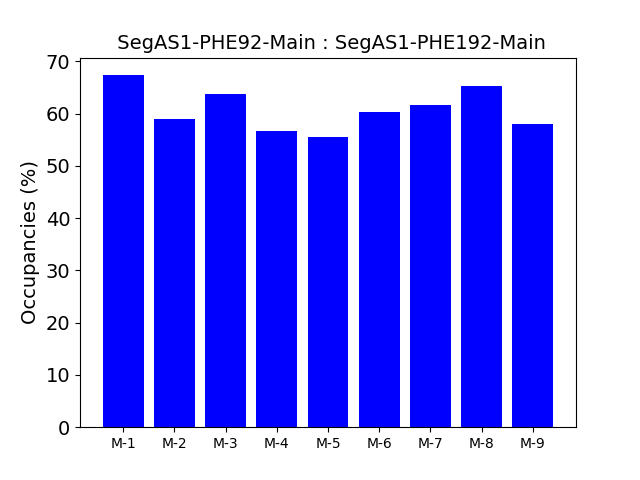

Supplement: SC-015-D4SC04364B-s001 [file SC-015-D4SC04364B-s001.zip › Inner_h_bonds_states/open/SegAS1-PHE92-Main_SegAS1-PHE192-Main.png]

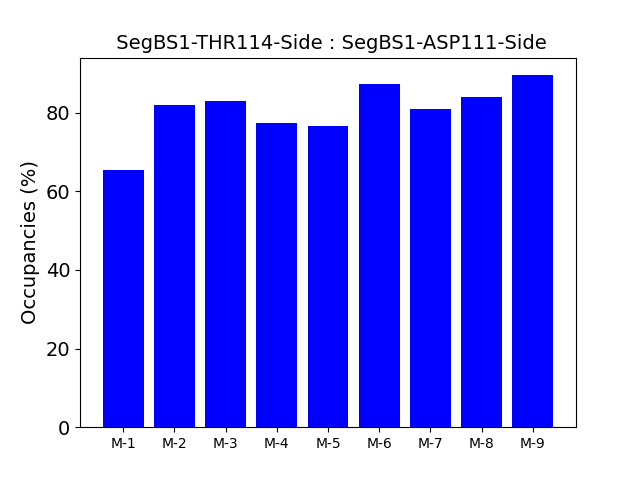

Supplement: SC-015-D4SC04364B-s001 [file SC-015-D4SC04364B-s001.zip › Inner_h_bonds_states/open/SegBS1-THR114-Side_SegBS1-ASP111-Side.png]

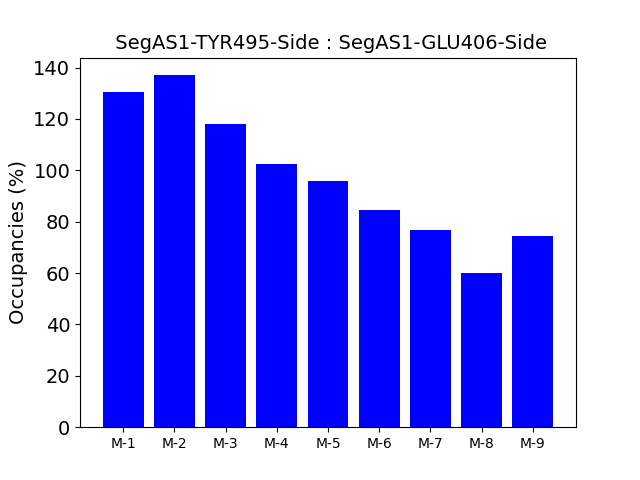

Supplement: SC-015-D4SC04364B-s001 [file SC-015-D4SC04364B-s001.zip › Inner_h_bonds_states/open/SegAS1-TYR495-Side_SegAS1-GLU406-Side.png]

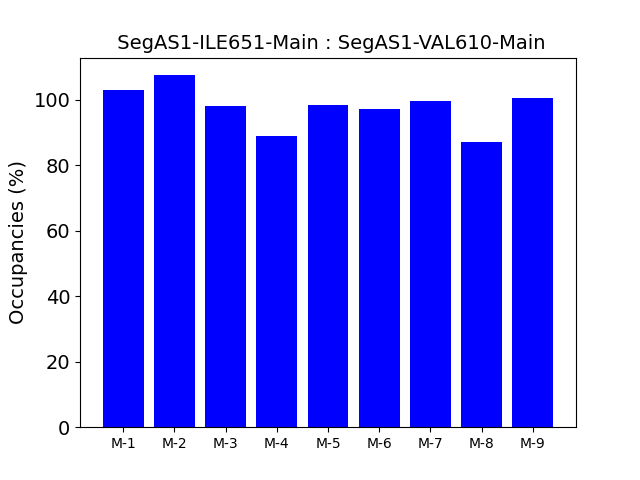

Supplement: SC-015-D4SC04364B-s001 [file SC-015-D4SC04364B-s001.zip › Inner_h_bonds_states/open/SegAS1-ILE651-Main_SegAS1-VAL610-Main.png]

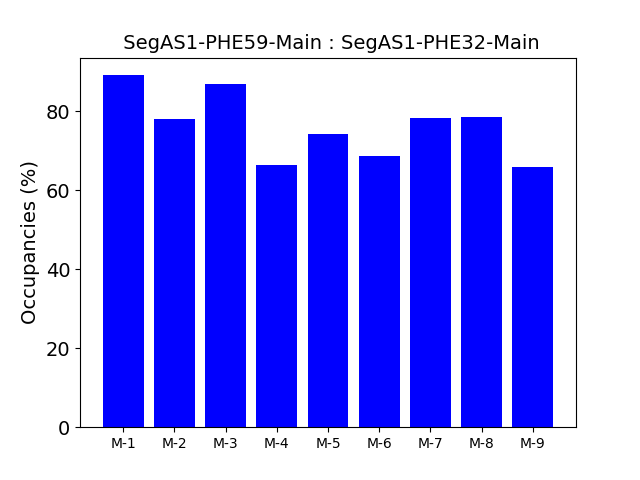

Supplement: SC-015-D4SC04364B-s001 [file SC-015-D4SC04364B-s001.zip › Inner_h_bonds_states/open/SegAS1-PHE59-Main_SegAS1-PHE32-Main.png]

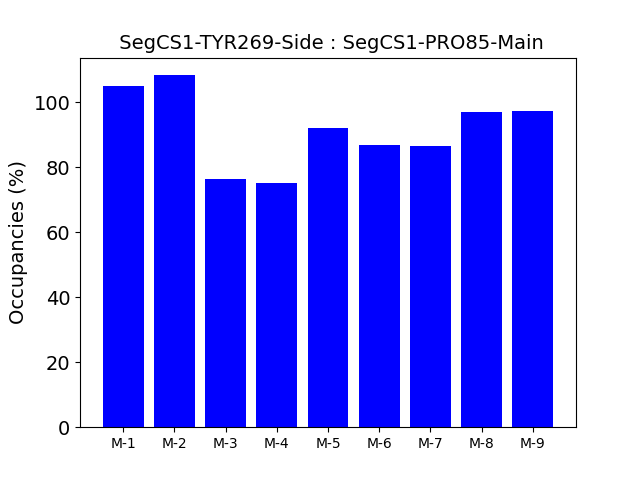

Supplement: SC-015-D4SC04364B-s001 [file SC-015-D4SC04364B-s001.zip › Inner_h_bonds_states/open/SegCS1-TYR269-Side_SegCS1-PRO85-Main.png]

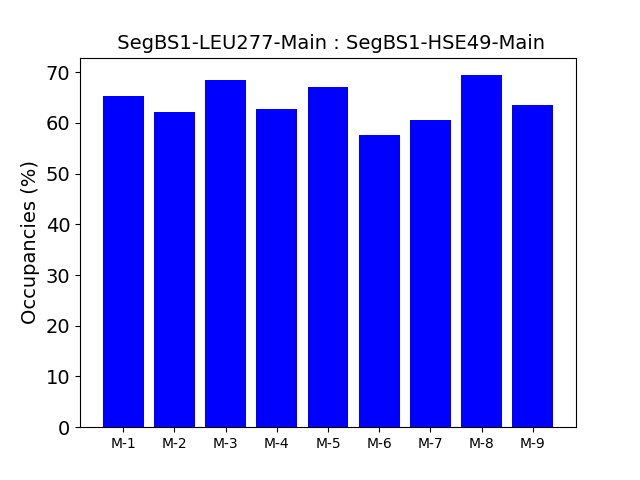

Supplement: SC-015-D4SC04364B-s001 [file SC-015-D4SC04364B-s001.zip › Inner_h_bonds_states/open/SegBS1-LEU277-Main_SegBS1-HSE49-Main.png]

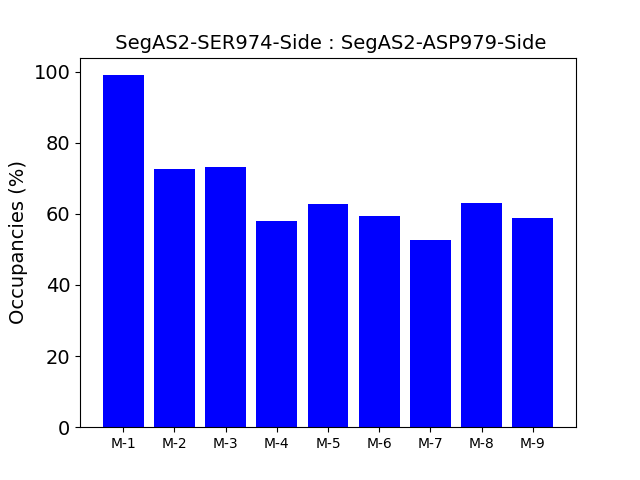

Supplement: SC-015-D4SC04364B-s001 [file SC-015-D4SC04364B-s001.zip › Inner_h_bonds_states/open/SegAS2-SER974-Side_SegAS2-ASP979-Side.png]

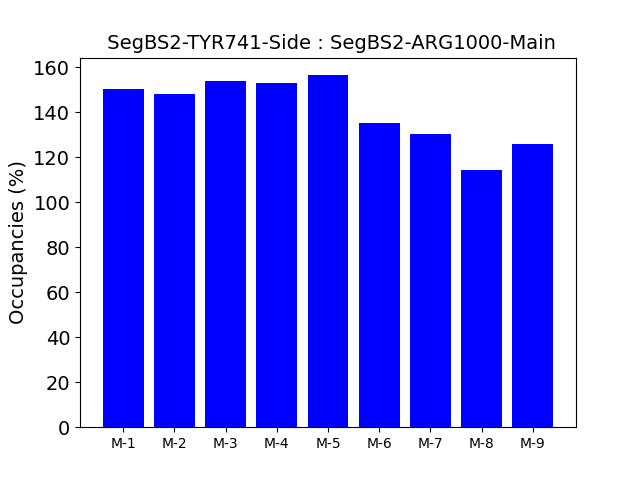

Supplement: SC-015-D4SC04364B-s001 [file SC-015-D4SC04364B-s001.zip › Inner_h_bonds_states/open/SegBS2-TYR741-Side_SegBS2-ARG1000-Main.png]

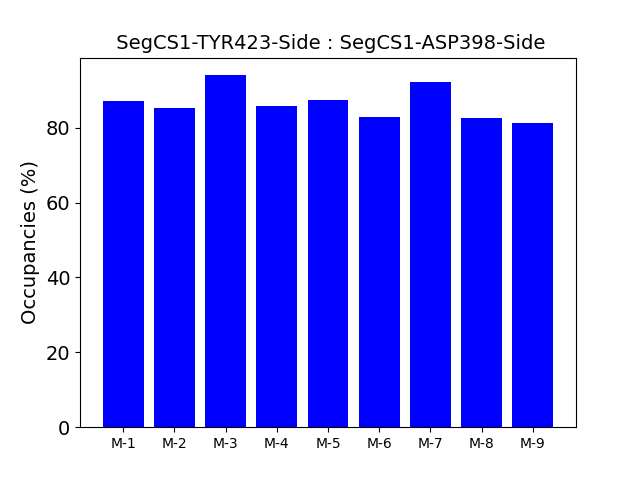

Supplement: SC-015-D4SC04364B-s001 [file SC-015-D4SC04364B-s001.zip › Inner_h_bonds_states/open/SegCS1-TYR423-Side_SegCS1-ASP398-Side.png]

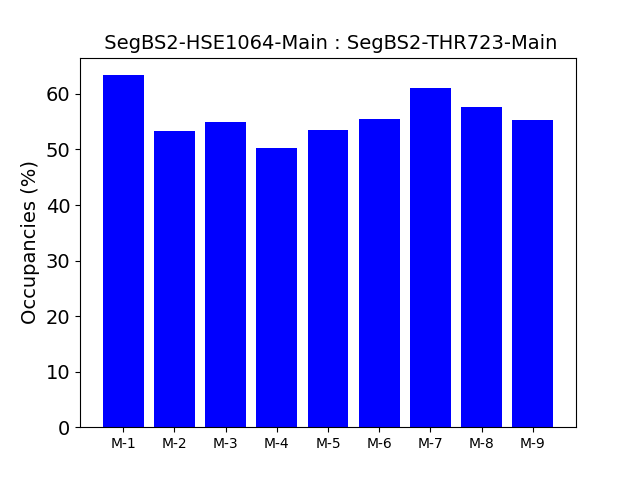

Supplement: SC-015-D4SC04364B-s001 [file SC-015-D4SC04364B-s001.zip › Inner_h_bonds_states/open/SegBS2-HSE1064-Main_SegBS2-THR723-Main.png]

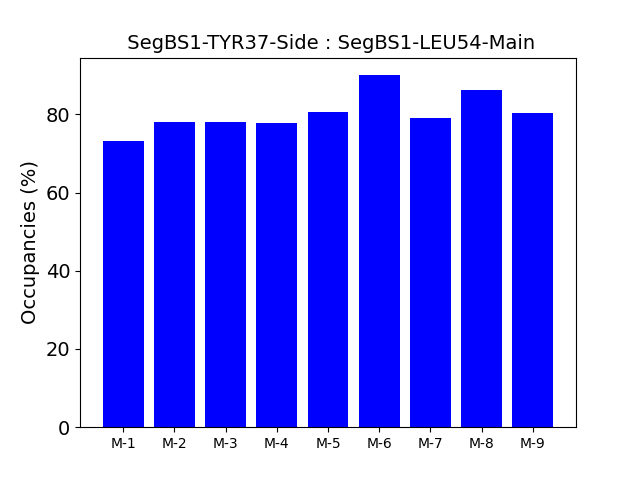

Supplement: SC-015-D4SC04364B-s001 [file SC-015-D4SC04364B-s001.zip › Inner_h_bonds_states/open/SegBS1-TYR37-Side_SegBS1-LEU54-Main.png]

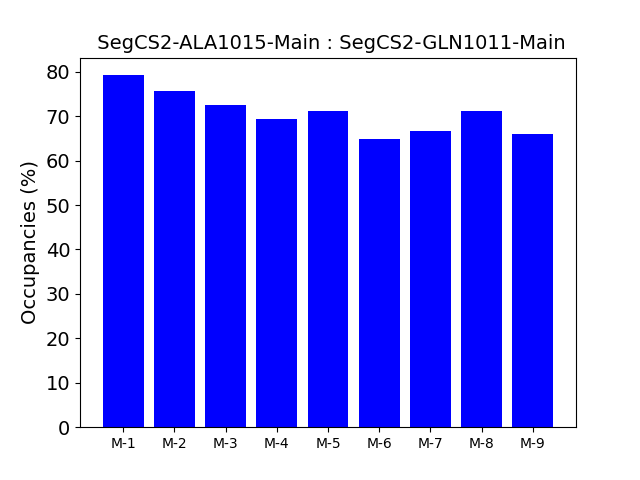

Supplement: SC-015-D4SC04364B-s001 [file SC-015-D4SC04364B-s001.zip › Inner_h_bonds_states/open/SegCS2-ALA1015-Main_SegCS2-GLN1011-Main.png]

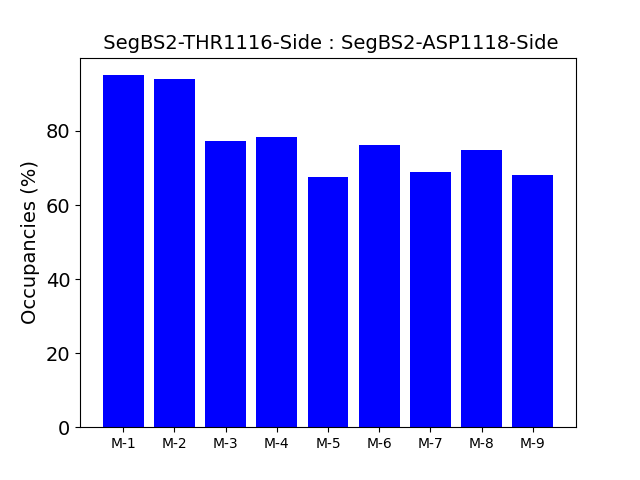

Supplement: SC-015-D4SC04364B-s001 [file SC-015-D4SC04364B-s001.zip › Inner_h_bonds_states/open/SegBS2-THR1116-Side_SegBS2-ASP1118-Side.png]

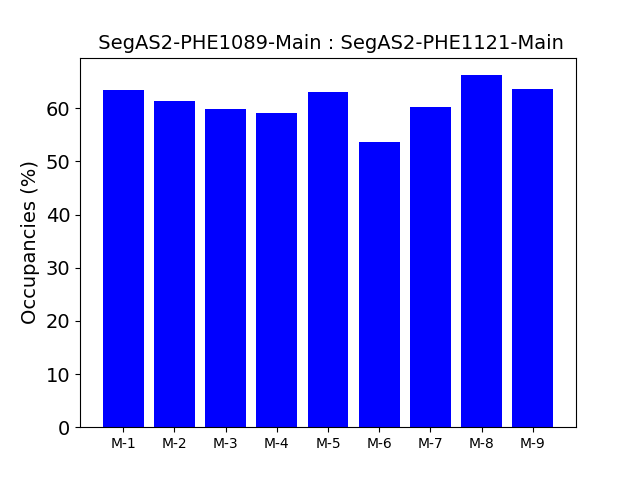

Supplement: SC-015-D4SC04364B-s001 [file SC-015-D4SC04364B-s001.zip › Inner_h_bonds_states/open/SegAS2-PHE1089-Main_SegAS2-PHE1121-Main.png]

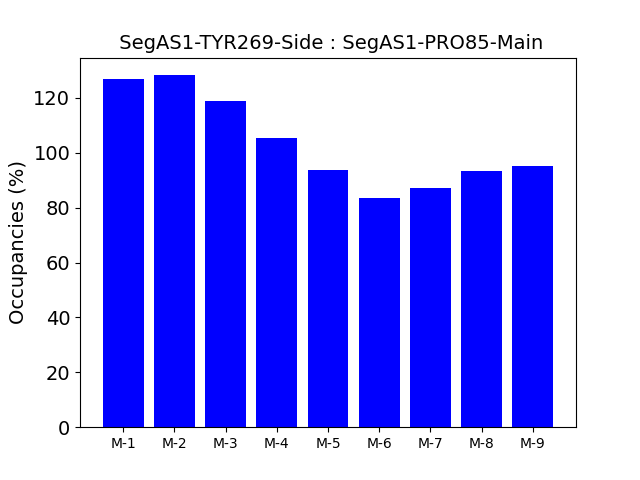

Supplement: SC-015-D4SC04364B-s001 [file SC-015-D4SC04364B-s001.zip › Inner_h_bonds_states/open/SegAS1-TYR269-Side_SegAS1-PRO85-Main.png]

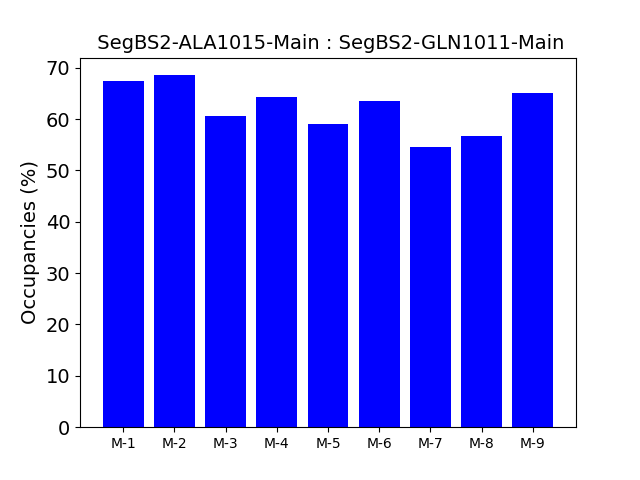

Supplement: SC-015-D4SC04364B-s001 [file SC-015-D4SC04364B-s001.zip › Inner_h_bonds_states/open/SegBS2-ALA1015-Main_SegBS2-GLN1011-Main.png]

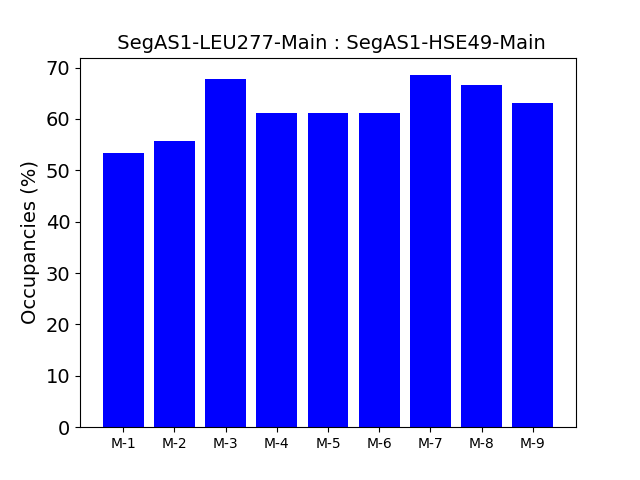

Supplement: SC-015-D4SC04364B-s001 [file SC-015-D4SC04364B-s001.zip › Inner_h_bonds_states/open/SegAS1-LEU277-Main_SegAS1-HSE49-Main.png]

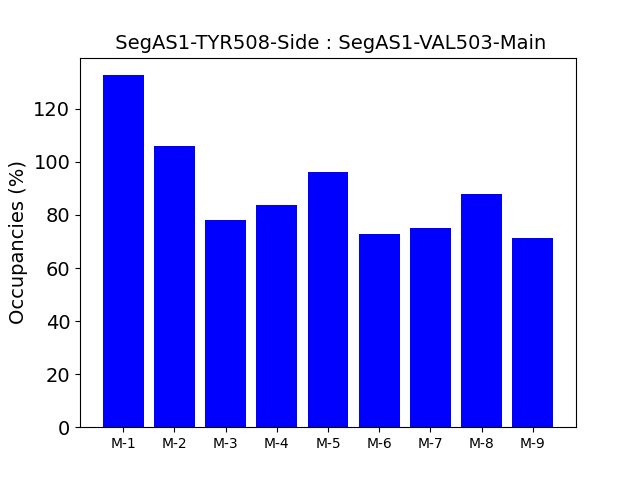

Supplement: SC-015-D4SC04364B-s001 [file SC-015-D4SC04364B-s001.zip › Inner_h_bonds_states/open/SegAS1-TYR508-Side_SegAS1-VAL503-Main.png]

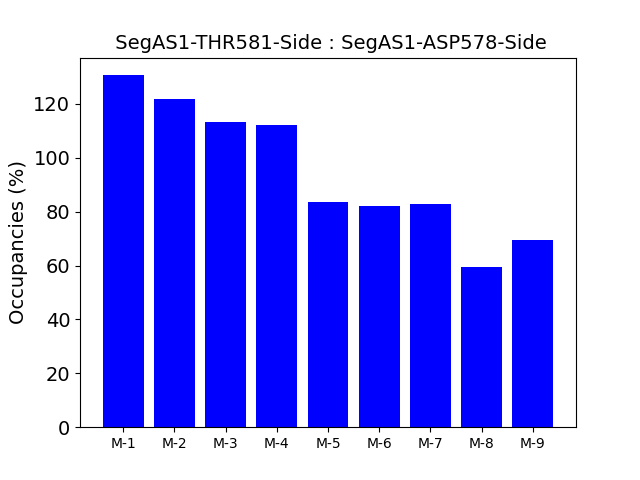

Supplement: SC-015-D4SC04364B-s001 [file SC-015-D4SC04364B-s001.zip › Inner_h_bonds_states/open/SegAS1-THR581-Side_SegAS1-ASP578-Side.png]

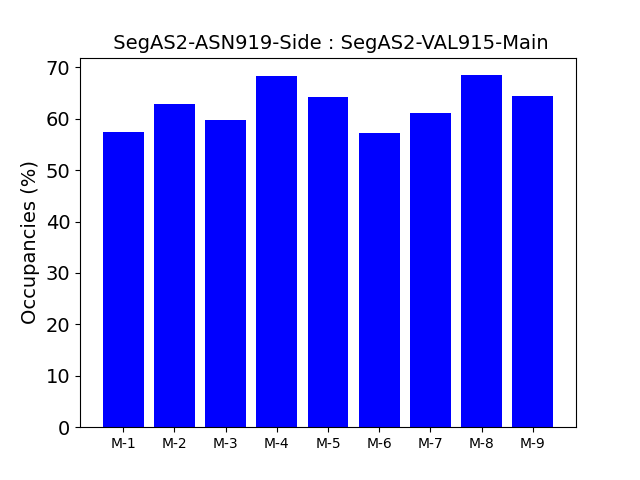

Supplement: SC-015-D4SC04364B-s001 [file SC-015-D4SC04364B-s001.zip › Inner_h_bonds_states/open/SegAS2-ASN919-Side_SegAS2-VAL915-Main.png]

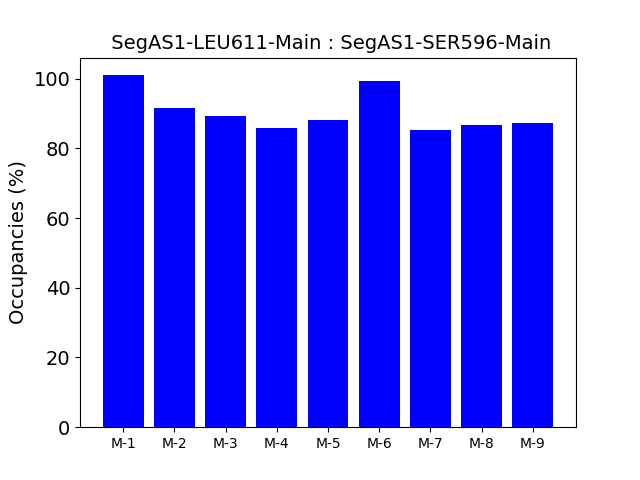

Supplement: SC-015-D4SC04364B-s001 [file SC-015-D4SC04364B-s001.zip › Inner_h_bonds_states/open/SegAS1-LEU611-Main_SegAS1-SER596-Main.png]

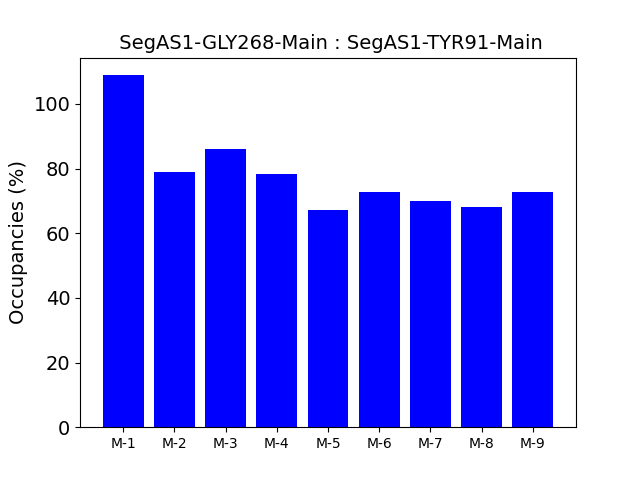

Supplement: SC-015-D4SC04364B-s001 [file SC-015-D4SC04364B-s001.zip › Inner_h_bonds_states/open/SegAS1-GLY268-Main_SegAS1-TYR91-Main.png]

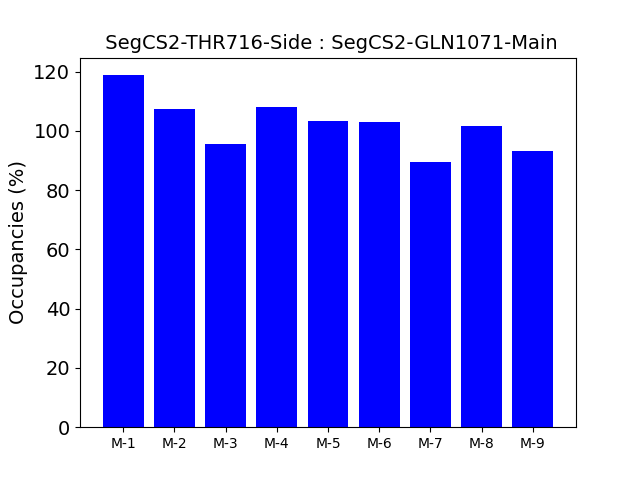

Supplement: SC-015-D4SC04364B-s001 [file SC-015-D4SC04364B-s001.zip › Inner_h_bonds_states/open/SegCS2-THR716-Side_SegCS2-GLN1071-Main.png]

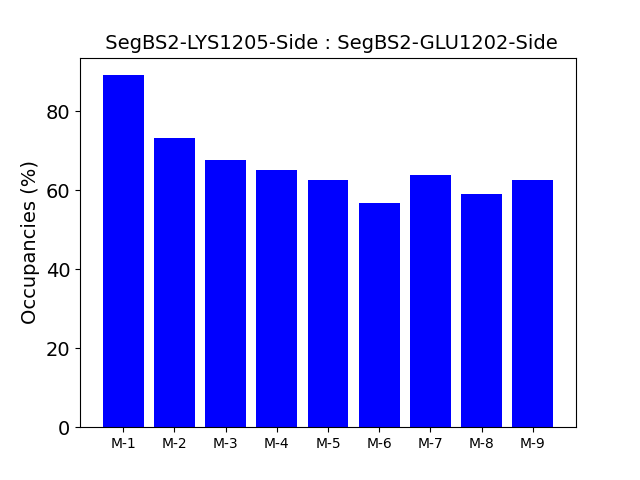

Supplement: SC-015-D4SC04364B-s001 [file SC-015-D4SC04364B-s001.zip › Inner_h_bonds_states/open/SegBS2-LYS1205-Side_SegBS2-GLU1202-Side.png]

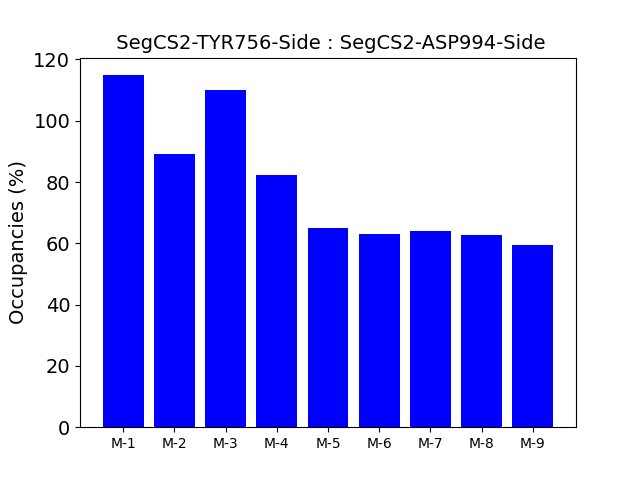

Supplement: SC-015-D4SC04364B-s001 [file SC-015-D4SC04364B-s001.zip › Inner_h_bonds_states/open/SegCS2-TYR756-Side_SegCS2-ASP994-Side.png]

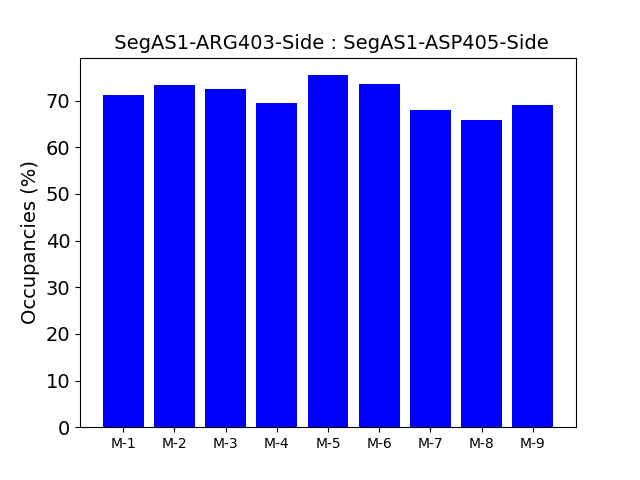

Supplement: SC-015-D4SC04364B-s001 [file SC-015-D4SC04364B-s001.zip › Inner_h_bonds_states/open/SegAS1-ARG403-Side_SegAS1-ASP405-Side.png]

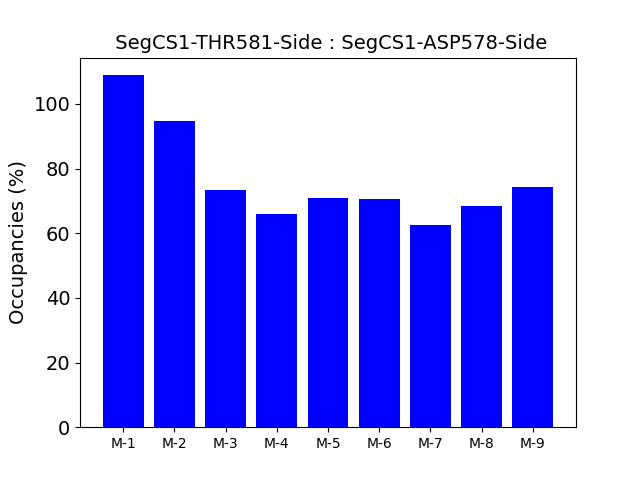

Supplement: SC-015-D4SC04364B-s001 [file SC-015-D4SC04364B-s001.zip › Inner_h_bonds_states/open/SegCS1-THR581-Side_SegCS1-ASP578-Side.png]

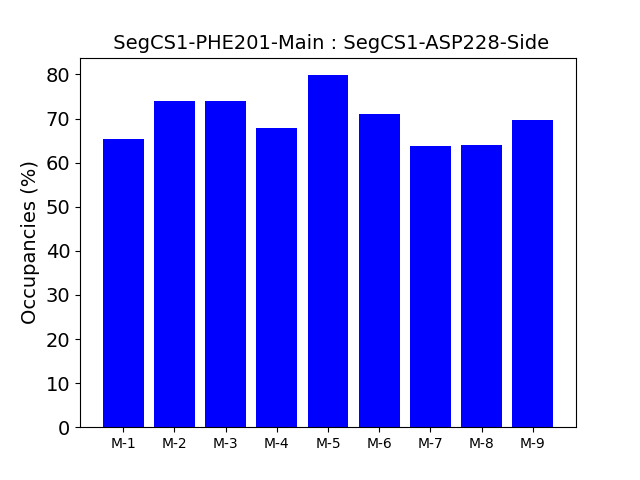

Supplement: SC-015-D4SC04364B-s001 [file SC-015-D4SC04364B-s001.zip › Inner_h_bonds_states/open/SegCS1-PHE201-Main_SegCS1-ASP228-Side.png]

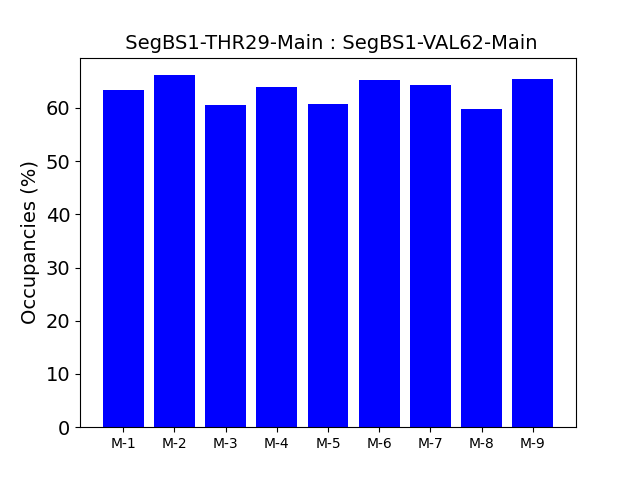

Supplement: SC-015-D4SC04364B-s001 [file SC-015-D4SC04364B-s001.zip › Inner_h_bonds_states/open/SegBS1-THR29-Main_SegBS1-VAL62-Main.png]

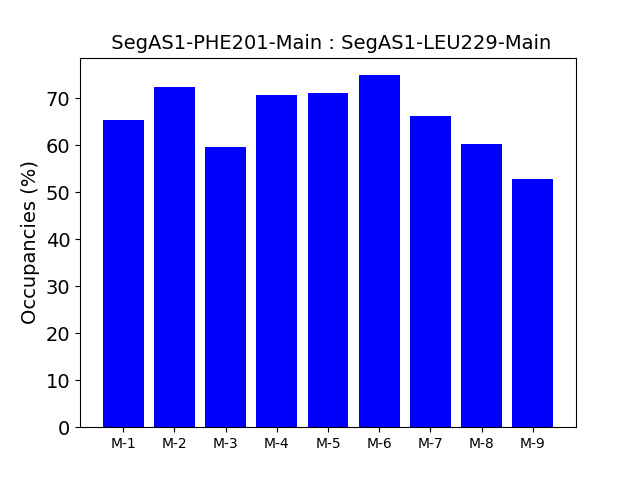

Supplement: SC-015-D4SC04364B-s001 [file SC-015-D4SC04364B-s001.zip › Inner_h_bonds_states/open/SegAS1-PHE201-Main_SegAS1-LEU229-Main.png]

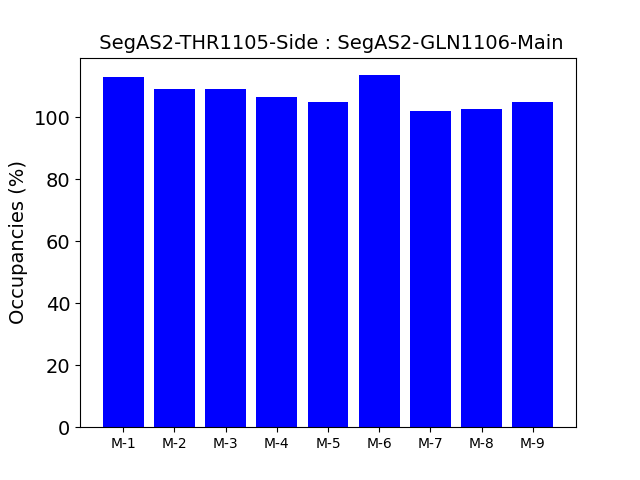

Supplement: SC-015-D4SC04364B-s001 [file SC-015-D4SC04364B-s001.zip › Inner_h_bonds_states/open/SegAS2-THR1105-Side_SegAS2-GLN1106-Main.png]

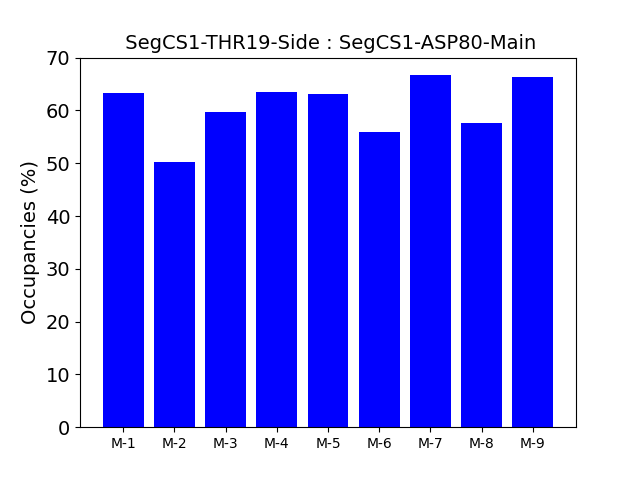

Supplement: SC-015-D4SC04364B-s001 [file SC-015-D4SC04364B-s001.zip › Inner_h_bonds_states/open/SegCS1-THR19-Side_SegCS1-ASP80-Main.png]

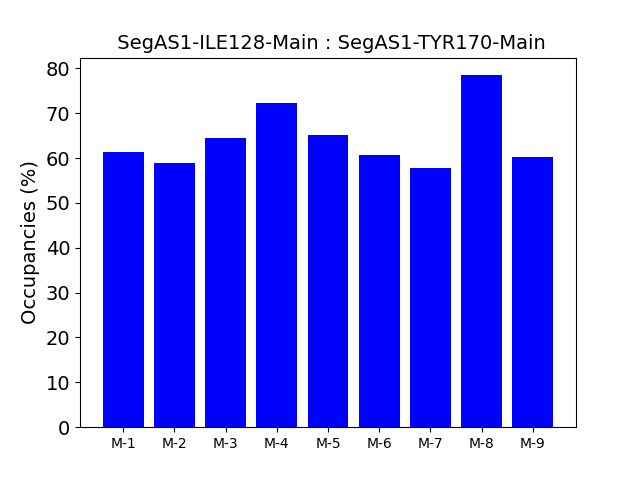

Supplement: SC-015-D4SC04364B-s001 [file SC-015-D4SC04364B-s001.zip › Inner_h_bonds_states/open/SegAS1-ILE128-Main_SegAS1-TYR170-Main.png]

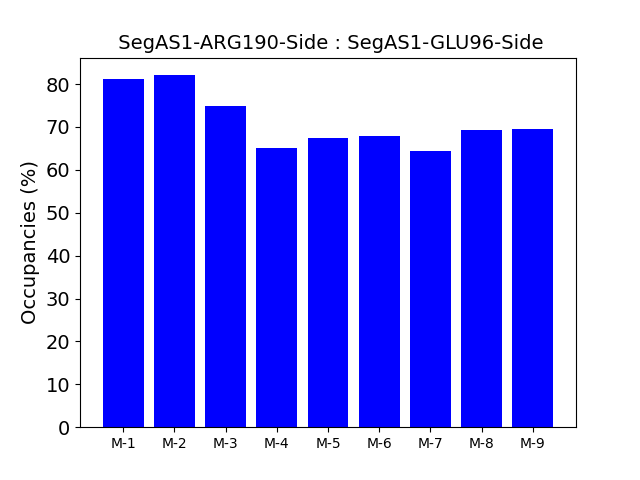

Supplement: SC-015-D4SC04364B-s001 [file SC-015-D4SC04364B-s001.zip › Inner_h_bonds_states/open/SegAS1-ARG190-Side_SegAS1-GLU96-Side.png]

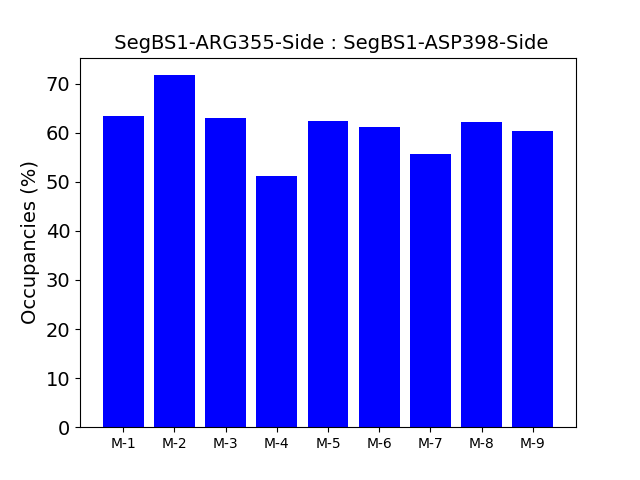

Supplement: SC-015-D4SC04364B-s001 [file SC-015-D4SC04364B-s001.zip › Inner_h_bonds_states/open/SegBS1-ARG355-Side_SegBS1-ASP398-Side.png]

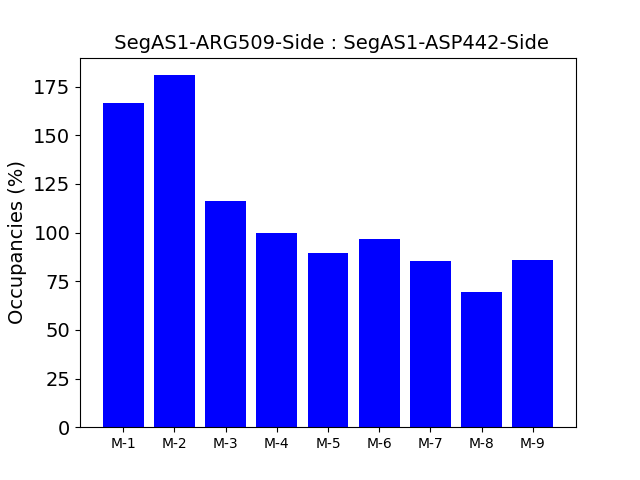

Supplement: SC-015-D4SC04364B-s001 [file SC-015-D4SC04364B-s001.zip › Inner_h_bonds_states/open/SegAS1-ARG509-Side_SegAS1-ASP442-Side.png]

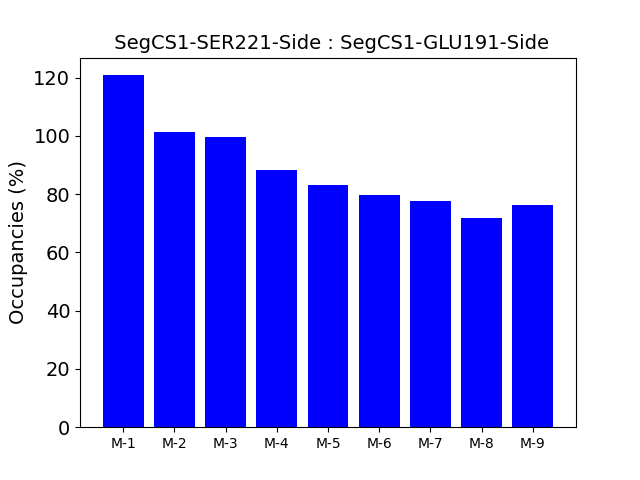

Supplement: SC-015-D4SC04364B-s001 [file SC-015-D4SC04364B-s001.zip › Inner_h_bonds_states/open/SegCS1-SER221-Side_SegCS1-GLU191-Side.png]

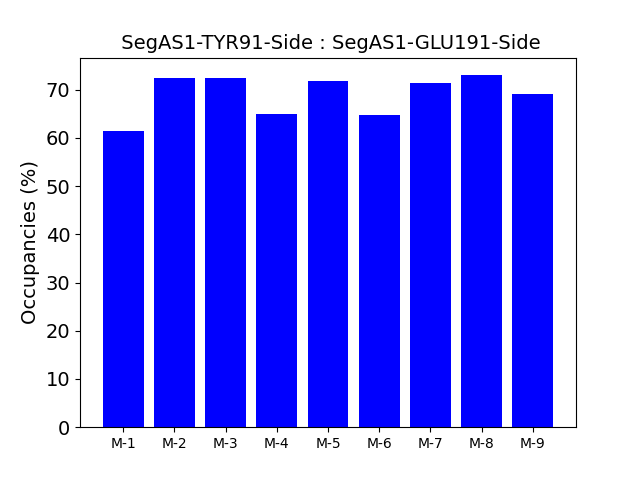

Supplement: SC-015-D4SC04364B-s001 [file SC-015-D4SC04364B-s001.zip › Inner_h_bonds_states/open/SegAS1-TYR91-Side_SegAS1-GLU191-Side.png]

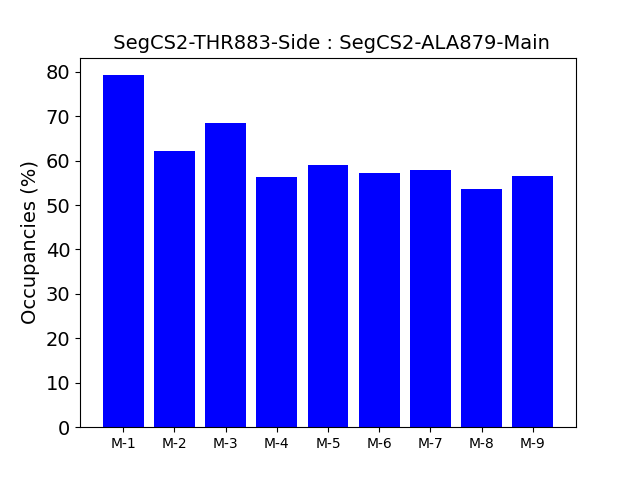

Supplement: SC-015-D4SC04364B-s001 [file SC-015-D4SC04364B-s001.zip › Inner_h_bonds_states/open/SegCS2-THR883-Side_SegCS2-ALA879-Main.png]

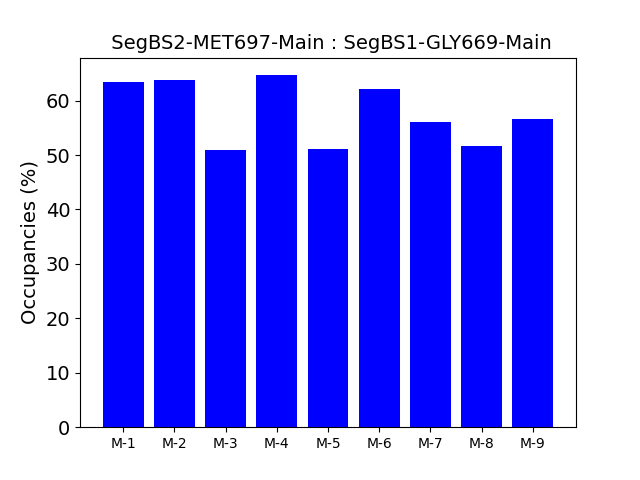

Supplement: SC-015-D4SC04364B-s001 [file SC-015-D4SC04364B-s001.zip › Inner_h_bonds_states/open/SegBS2-MET697-Main_SegBS1-GLY669-Main.png]

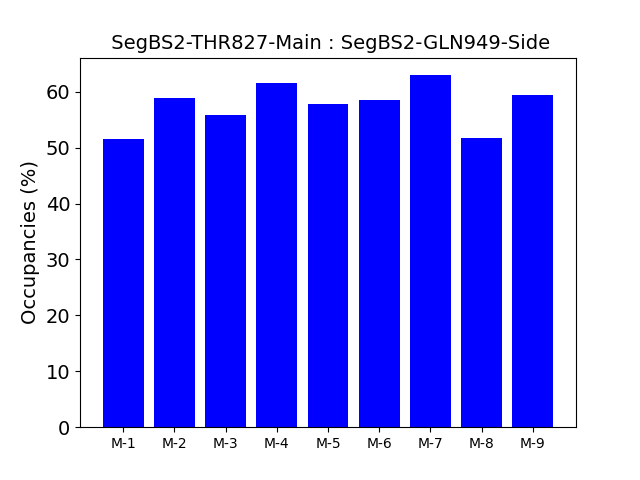

Supplement: SC-015-D4SC04364B-s001 [file SC-015-D4SC04364B-s001.zip › Inner_h_bonds_states/open/SegBS2-THR827-Main_SegBS2-GLN949-Side.png]

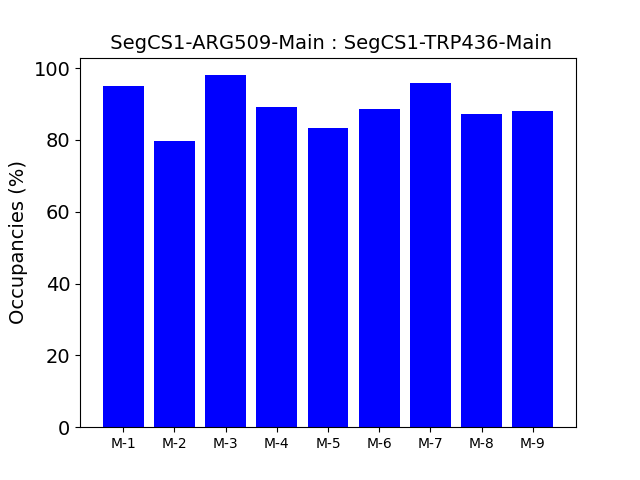

Supplement: SC-015-D4SC04364B-s001 [file SC-015-D4SC04364B-s001.zip › Inner_h_bonds_states/open/SegCS1-ARG509-Main_SegCS1-TRP436-Main.png]

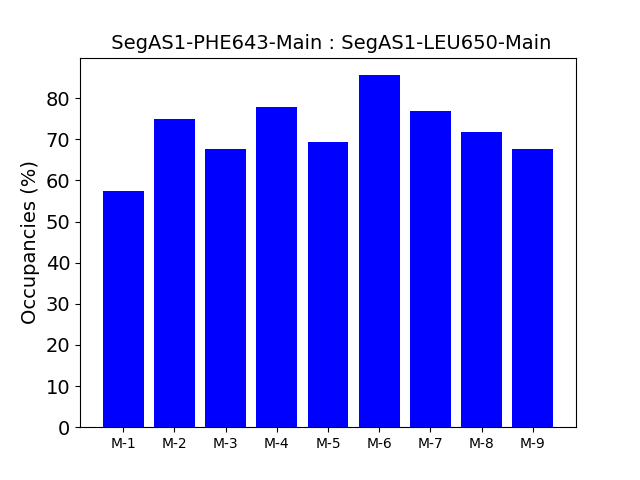

Supplement: SC-015-D4SC04364B-s001 [file SC-015-D4SC04364B-s001.zip › Inner_h_bonds_states/open/SegAS1-PHE643-Main_SegAS1-LEU650-Main.png]

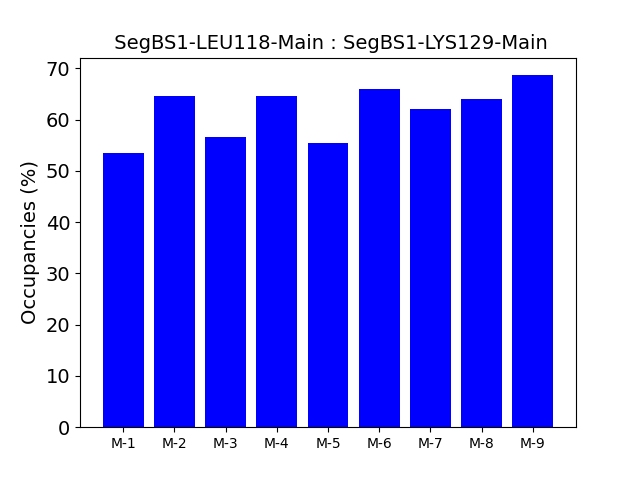

Supplement: SC-015-D4SC04364B-s001 [file SC-015-D4SC04364B-s001.zip › Inner_h_bonds_states/open/SegBS1-LEU118-Main_SegBS1-LYS129-Main.png]

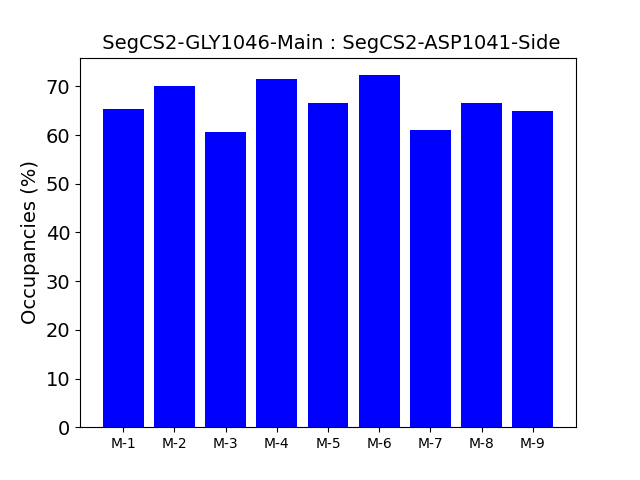

Supplement: SC-015-D4SC04364B-s001 [file SC-015-D4SC04364B-s001.zip › Inner_h_bonds_states/open/SegCS2-GLY1046-Main_SegCS2-ASP1041-Side.png]

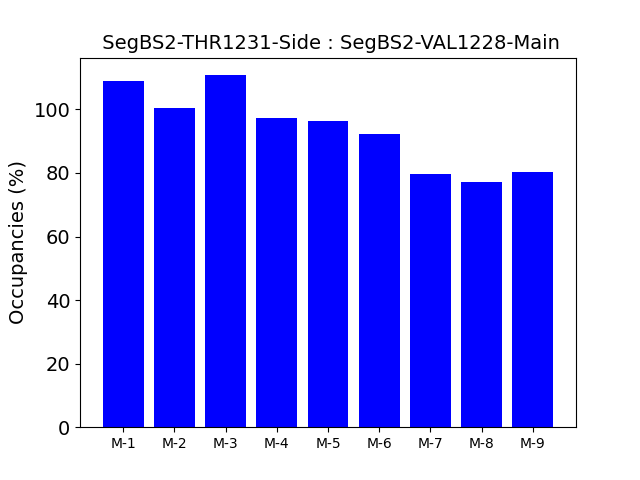

Supplement: SC-015-D4SC04364B-s001 [file SC-015-D4SC04364B-s001.zip › Inner_h_bonds_states/open/SegBS2-THR1231-Side_SegBS2-VAL1228-Main.png]

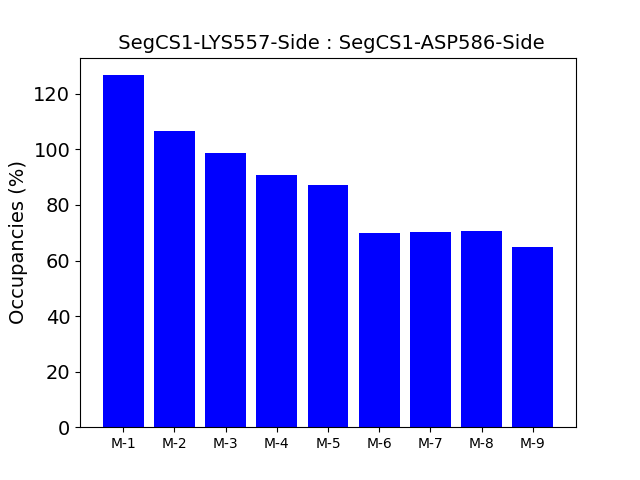

Supplement: SC-015-D4SC04364B-s001 [file SC-015-D4SC04364B-s001.zip › Inner_h_bonds_states/open/SegCS1-LYS557-Side_SegCS1-ASP586-Side.png]

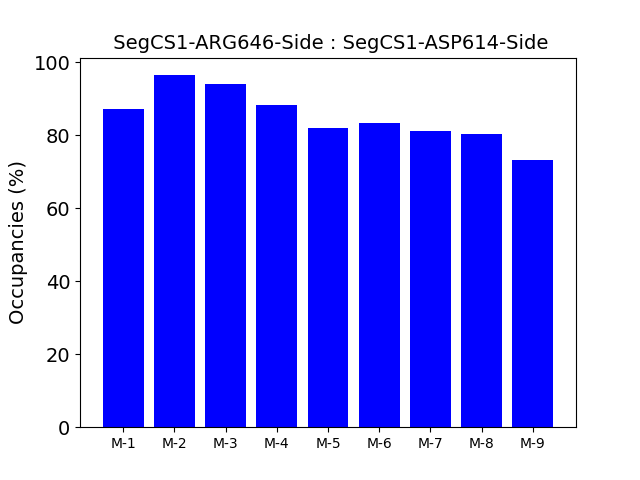

Supplement: SC-015-D4SC04364B-s001 [file SC-015-D4SC04364B-s001.zip › Inner_h_bonds_states/open/SegCS1-ARG646-Side_SegCS1-ASP614-Side.png]

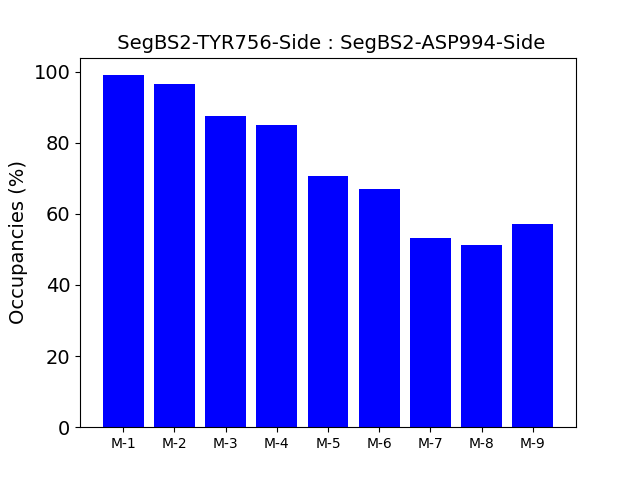

Supplement: SC-015-D4SC04364B-s001 [file SC-015-D4SC04364B-s001.zip › Inner_h_bonds_states/open/SegBS2-TYR756-Side_SegBS2-ASP994-Side.png]

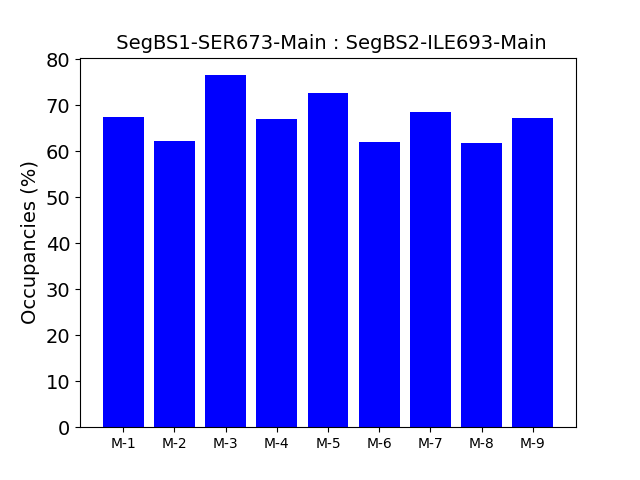

Supplement: SC-015-D4SC04364B-s001 [file SC-015-D4SC04364B-s001.zip › Inner_h_bonds_states/open/SegBS1-SER673-Main_SegBS2-ILE693-Main.png]

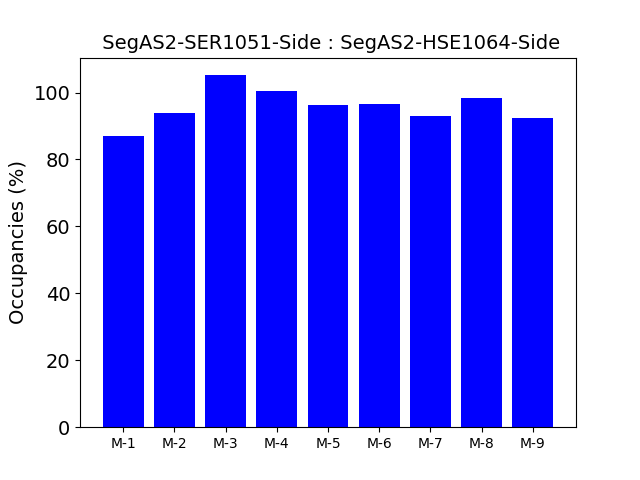

Supplement: SC-015-D4SC04364B-s001 [file SC-015-D4SC04364B-s001.zip › Inner_h_bonds_states/open/SegAS2-SER1051-Side_SegAS2-HSE1064-Side.png]

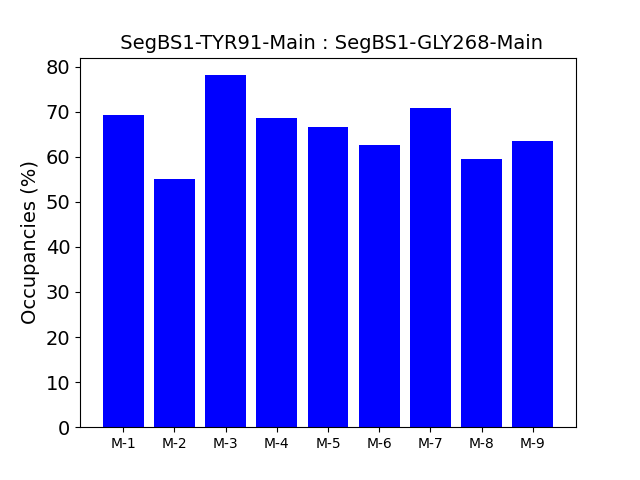

Supplement: SC-015-D4SC04364B-s001 [file SC-015-D4SC04364B-s001.zip › Inner_h_bonds_states/open/SegBS1-TYR91-Main_SegBS1-GLY268-Main.png]

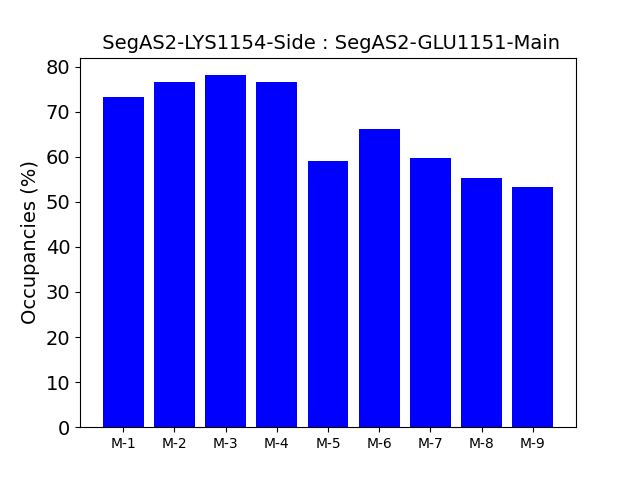

Supplement: SC-015-D4SC04364B-s001 [file SC-015-D4SC04364B-s001.zip › Inner_h_bonds_states/open/SegAS2-LYS1154-Side_SegAS2-GLU1151-Main.png]

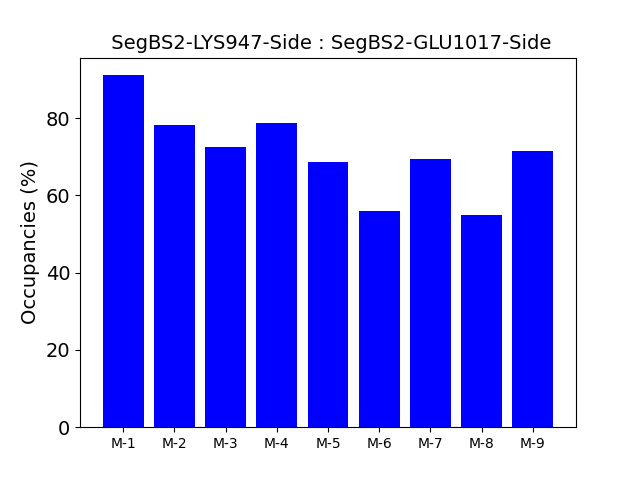

Supplement: SC-015-D4SC04364B-s001 [file SC-015-D4SC04364B-s001.zip › Inner_h_bonds_states/open/SegBS2-LYS947-Side_SegBS2-GLU1017-Side.png]

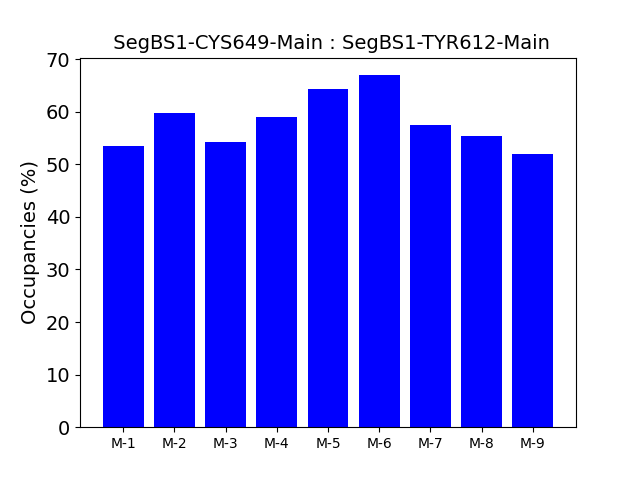

Supplement: SC-015-D4SC04364B-s001 [file SC-015-D4SC04364B-s001.zip › Inner_h_bonds_states/open/SegBS1-CYS649-Main_SegBS1-TYR612-Main.png]

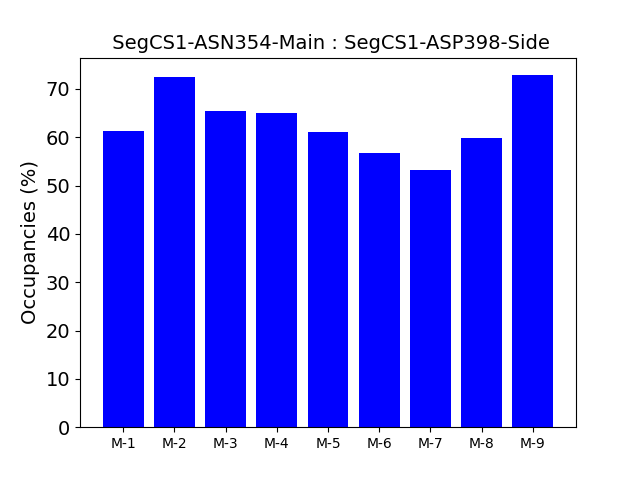

Supplement: SC-015-D4SC04364B-s001 [file SC-015-D4SC04364B-s001.zip › Inner_h_bonds_states/open/SegCS1-ASN354-Main_SegCS1-ASP398-Side.png]

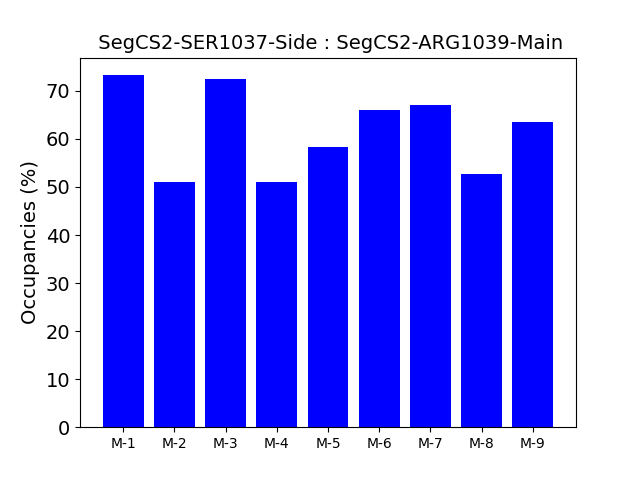

Supplement: SC-015-D4SC04364B-s001 [file SC-015-D4SC04364B-s001.zip › Inner_h_bonds_states/open/SegCS2-SER1037-Side_SegCS2-ARG1039-Main.png]
