# Supplementary figures and images for: Water–glycan interactions drive the SARS-CoV-2 spike dynamics: insights into glycan-gate control and camouflage mechanisms (part 3 of 4)
Source: Chem Sci. 2024 Aug 23;15(35):14177–87. doi: 10.1039/d4sc04364b (PMC11359970; doi:10.1039/d4sc04364b)

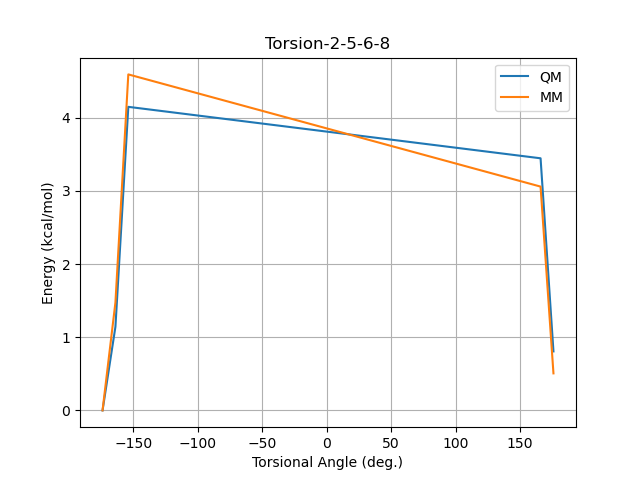

Supplement: SC-015-D4SC04364B-s002 [file SC-015-D4SC04364B-s002.zip › torsion_fit/AFUC/torsion_fitting-2-5-6-8.png]

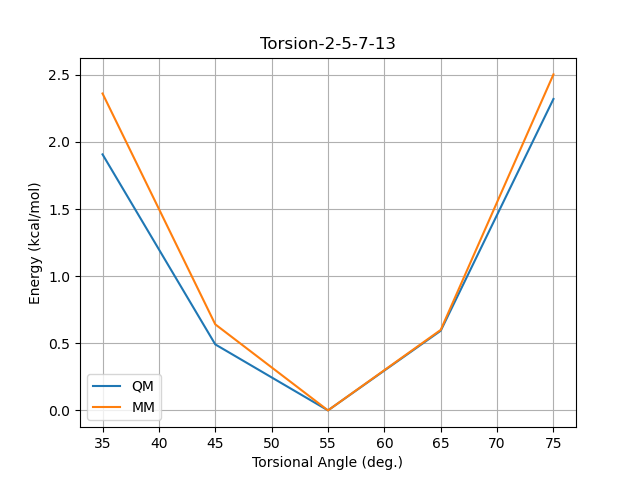

Supplement: SC-015-D4SC04364B-s002 [file SC-015-D4SC04364B-s002.zip › torsion_fit/AFUC/torsion_fitting-2-5-7-13.png]

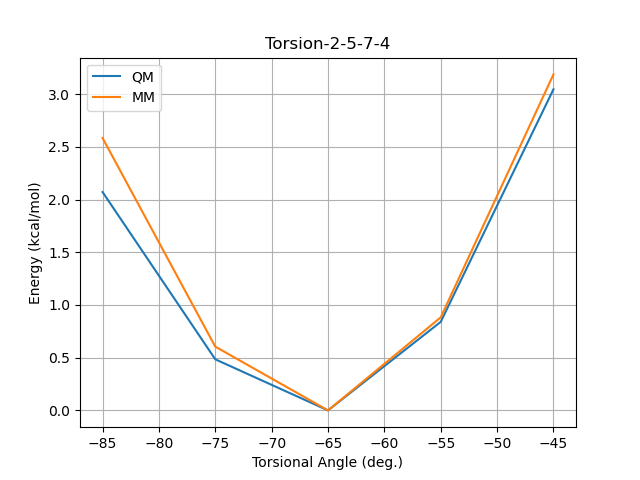

Supplement: SC-015-D4SC04364B-s002 [file SC-015-D4SC04364B-s002.zip › torsion_fit/AFUC/torsion_fitting-2-5-7-4.png]

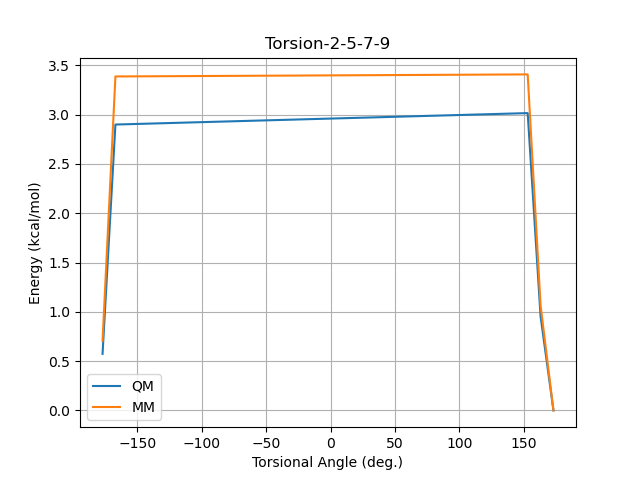

Supplement: SC-015-D4SC04364B-s002 [file SC-015-D4SC04364B-s002.zip › torsion_fit/AFUC/torsion_fitting-2-5-7-9.png]

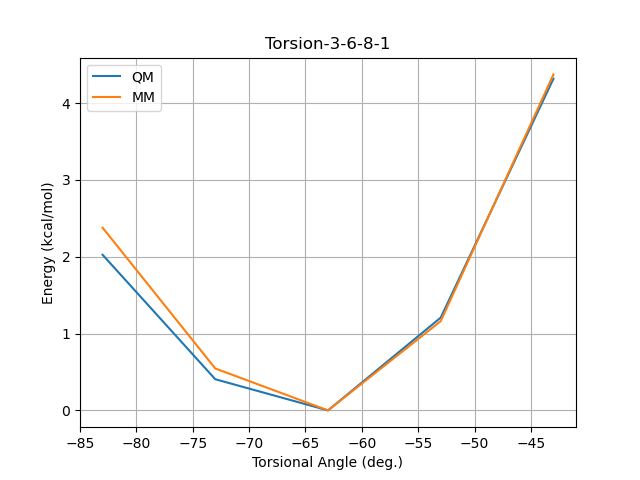

Supplement: SC-015-D4SC04364B-s002 [file SC-015-D4SC04364B-s002.zip › torsion_fit/AFUC/torsion_fitting-3-6-8-1.png]

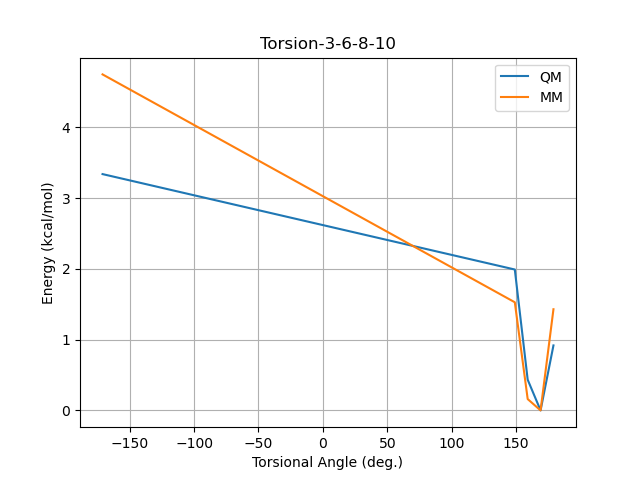

Supplement: SC-015-D4SC04364B-s002 [file SC-015-D4SC04364B-s002.zip › torsion_fit/AFUC/torsion_fitting-3-6-8-10.png]

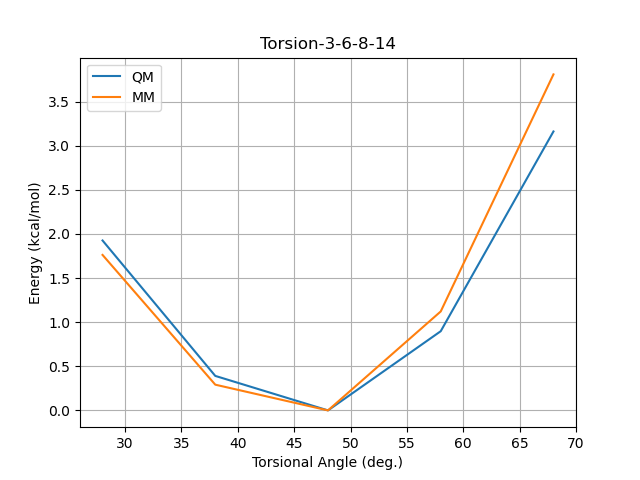

Supplement: SC-015-D4SC04364B-s002 [file SC-015-D4SC04364B-s002.zip › torsion_fit/AFUC/torsion_fitting-3-6-8-14.png]

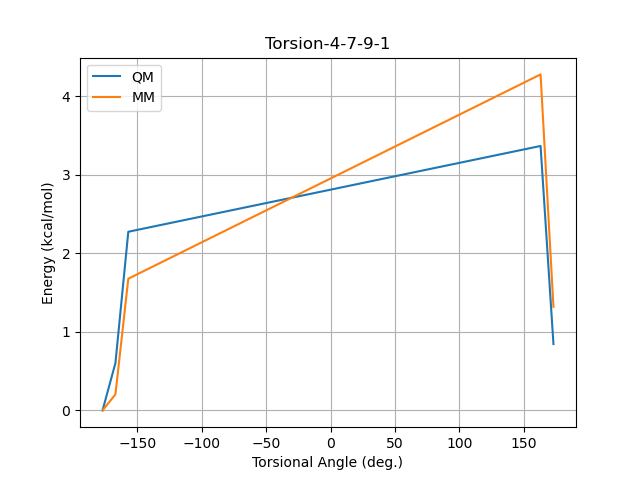

Supplement: SC-015-D4SC04364B-s002 [file SC-015-D4SC04364B-s002.zip › torsion_fit/AFUC/torsion_fitting-4-7-9-1.png]

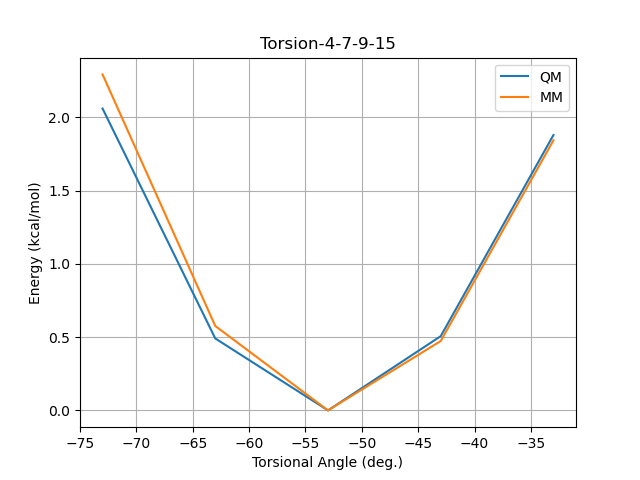

Supplement: SC-015-D4SC04364B-s002 [file SC-015-D4SC04364B-s002.zip › torsion_fit/AFUC/torsion_fitting-4-7-9-15.png]

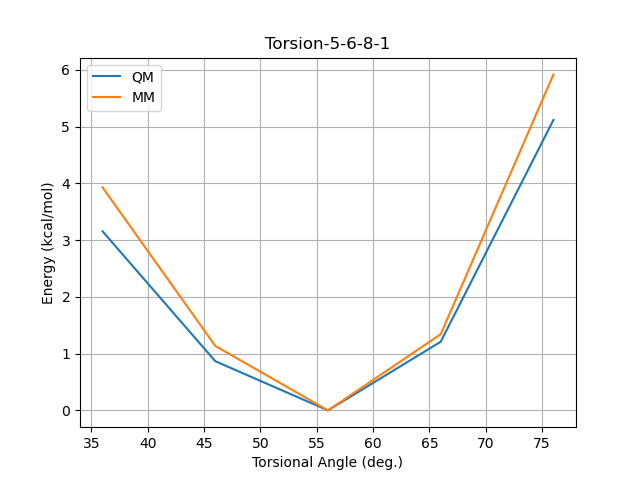

Supplement: SC-015-D4SC04364B-s002 [file SC-015-D4SC04364B-s002.zip › torsion_fit/AFUC/torsion_fitting-5-6-8-1.png]

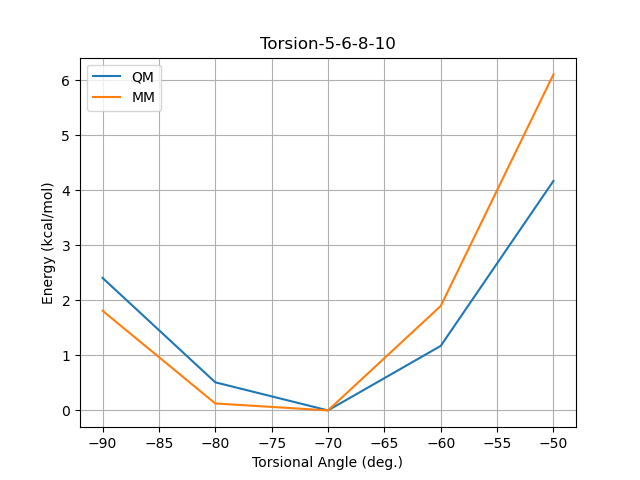

Supplement: SC-015-D4SC04364B-s002 [file SC-015-D4SC04364B-s002.zip › torsion_fit/AFUC/torsion_fitting-5-6-8-10.png]

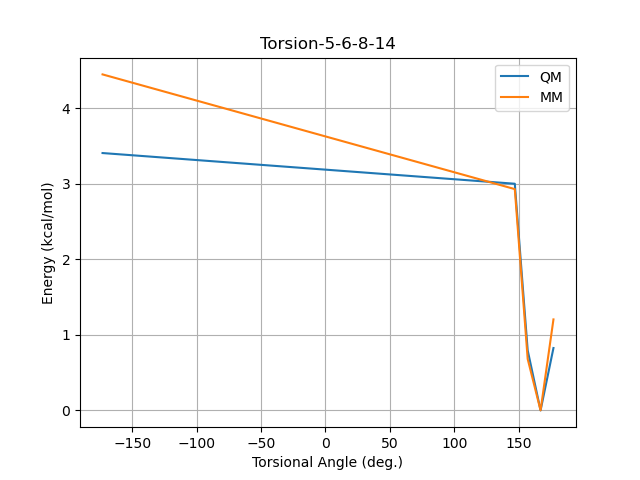

Supplement: SC-015-D4SC04364B-s002 [file SC-015-D4SC04364B-s002.zip › torsion_fit/AFUC/torsion_fitting-5-6-8-14.png]

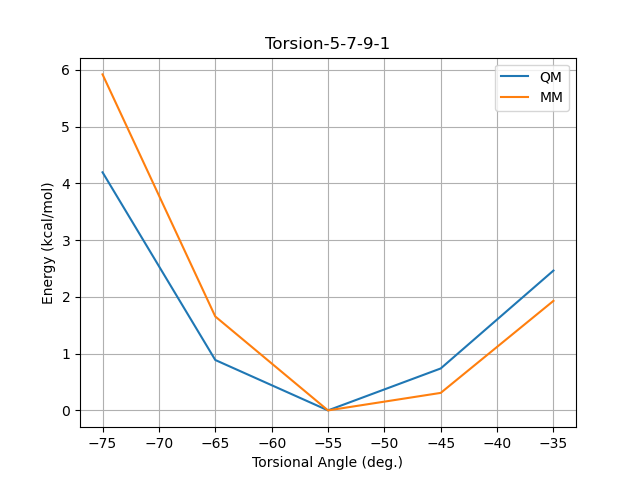

Supplement: SC-015-D4SC04364B-s002 [file SC-015-D4SC04364B-s002.zip › torsion_fit/AFUC/torsion_fitting-5-7-9-1.png]

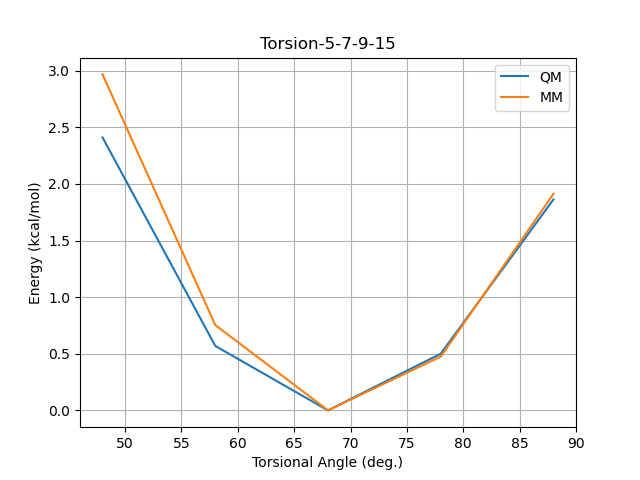

Supplement: SC-015-D4SC04364B-s002 [file SC-015-D4SC04364B-s002.zip › torsion_fit/AFUC/torsion_fitting-5-7-9-15.png]

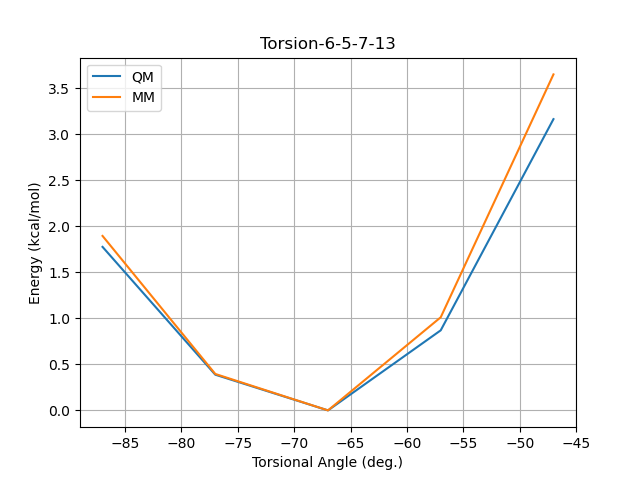

Supplement: SC-015-D4SC04364B-s002 [file SC-015-D4SC04364B-s002.zip › torsion_fit/AFUC/torsion_fitting-6-5-7-13.png]

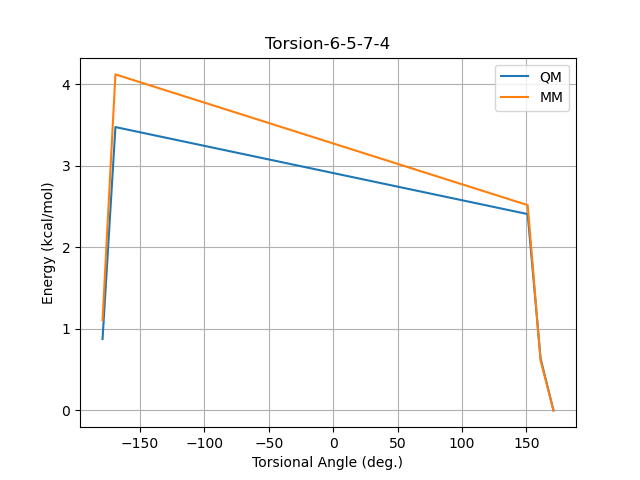

Supplement: SC-015-D4SC04364B-s002 [file SC-015-D4SC04364B-s002.zip › torsion_fit/AFUC/torsion_fitting-6-5-7-4.png]

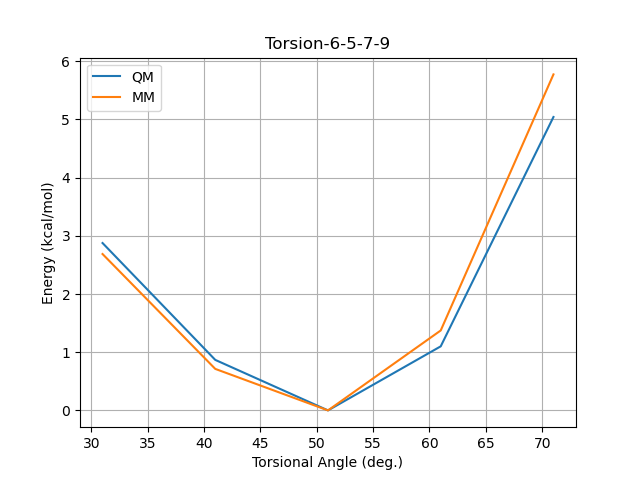

Supplement: SC-015-D4SC04364B-s002 [file SC-015-D4SC04364B-s002.zip › torsion_fit/AFUC/torsion_fitting-6-5-7-9.png]

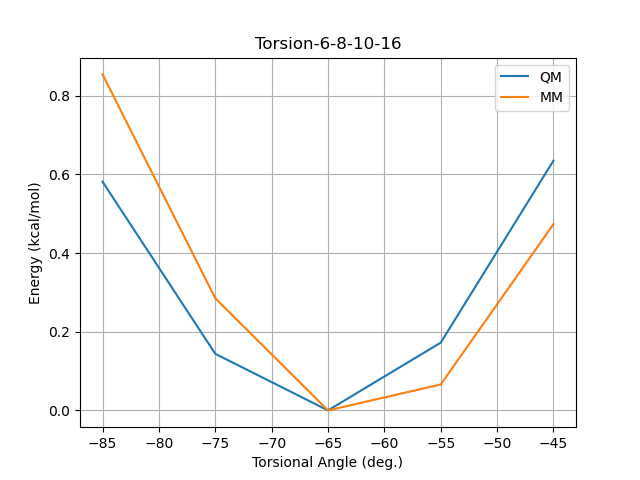

Supplement: SC-015-D4SC04364B-s002 [file SC-015-D4SC04364B-s002.zip › torsion_fit/AFUC/torsion_fitting-6-8-10-16.png]

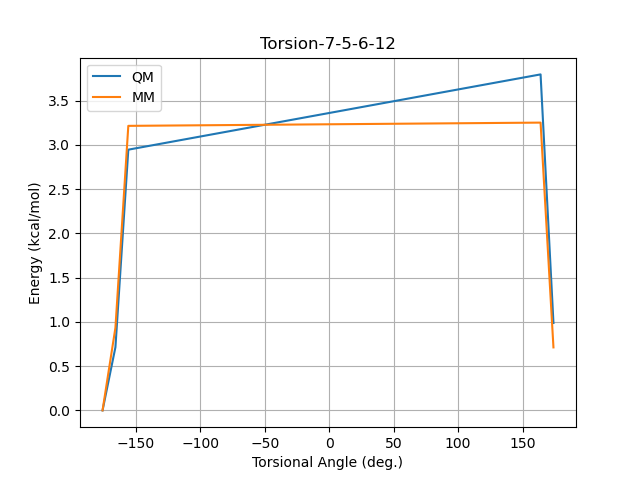

Supplement: SC-015-D4SC04364B-s002 [file SC-015-D4SC04364B-s002.zip › torsion_fit/AFUC/torsion_fitting-7-5-6-12.png]

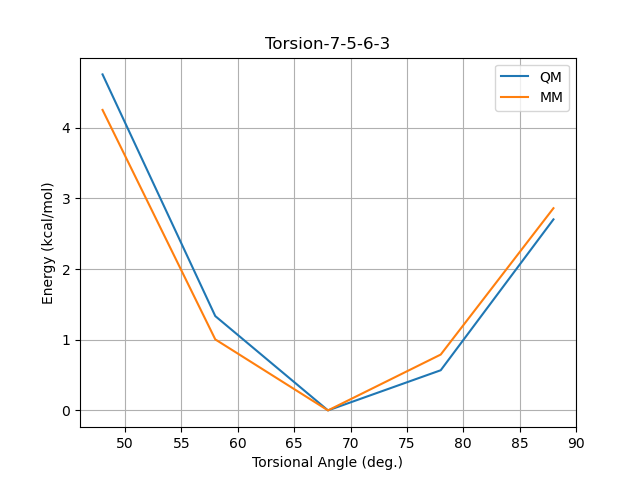

Supplement: SC-015-D4SC04364B-s002 [file SC-015-D4SC04364B-s002.zip › torsion_fit/AFUC/torsion_fitting-7-5-6-3.png]

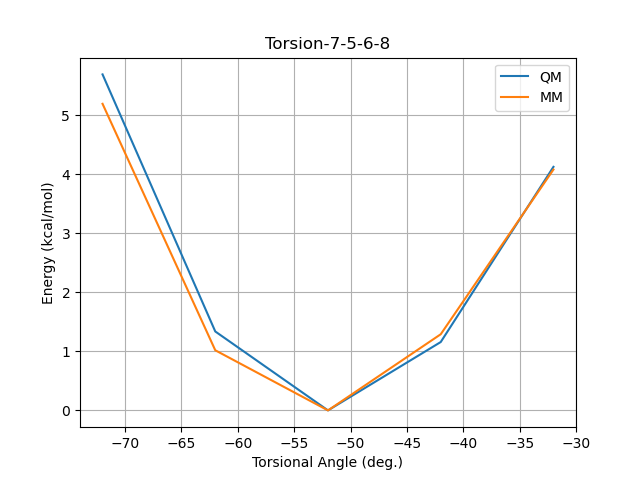

Supplement: SC-015-D4SC04364B-s002 [file SC-015-D4SC04364B-s002.zip › torsion_fit/AFUC/torsion_fitting-7-5-6-8.png]

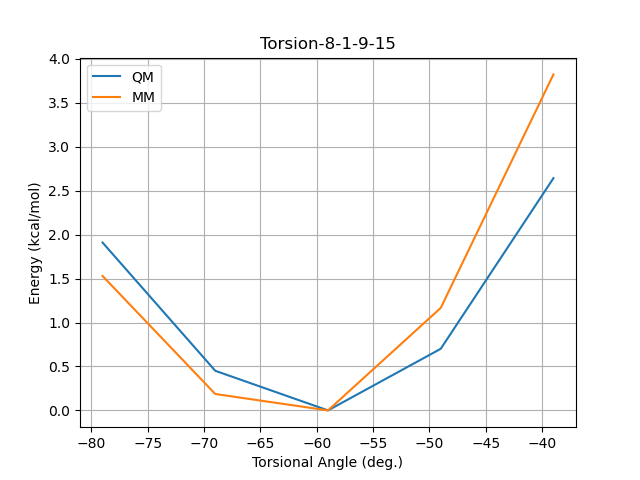

Supplement: SC-015-D4SC04364B-s002 [file SC-015-D4SC04364B-s002.zip › torsion_fit/AFUC/torsion_fitting-8-1-9-15.png]

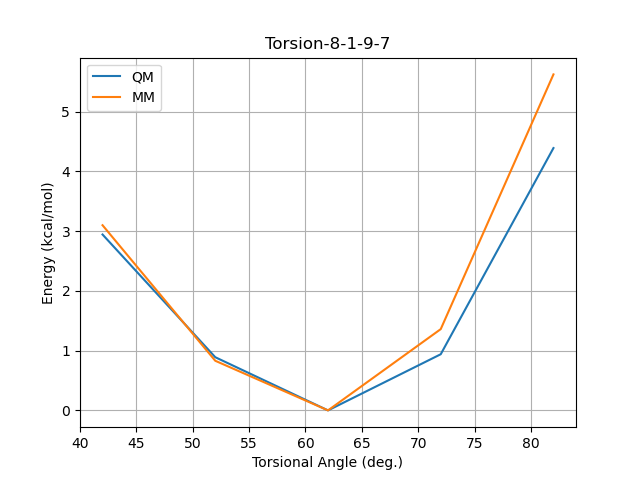

Supplement: SC-015-D4SC04364B-s002 [file SC-015-D4SC04364B-s002.zip › torsion_fit/AFUC/torsion_fitting-8-1-9-7.png]

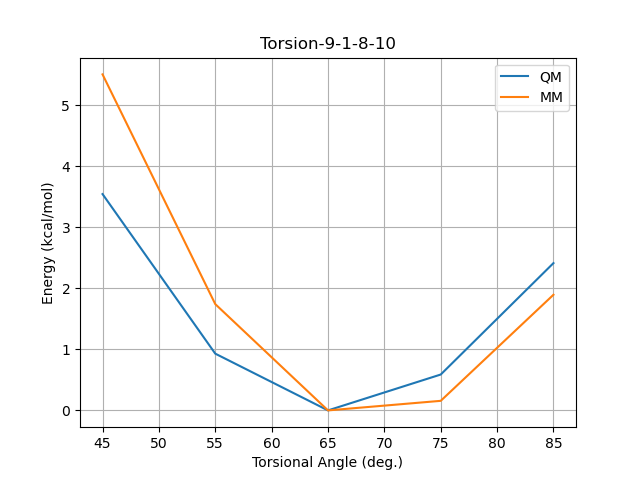

Supplement: SC-015-D4SC04364B-s002 [file SC-015-D4SC04364B-s002.zip › torsion_fit/AFUC/torsion_fitting-9-1-8-10.png]

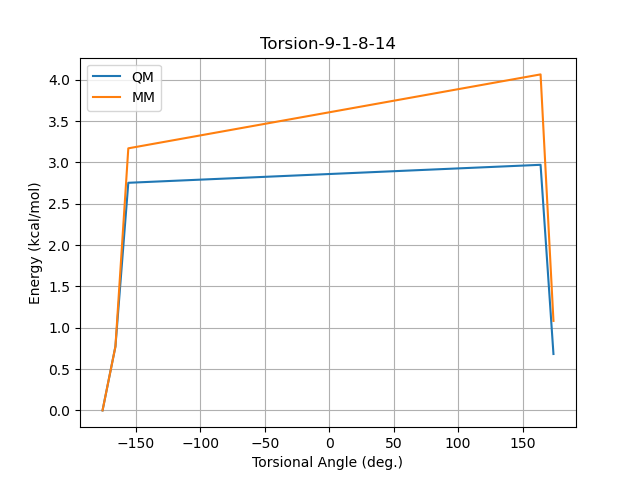

Supplement: SC-015-D4SC04364B-s002 [file SC-015-D4SC04364B-s002.zip › torsion_fit/AFUC/torsion_fitting-9-1-8-14.png]

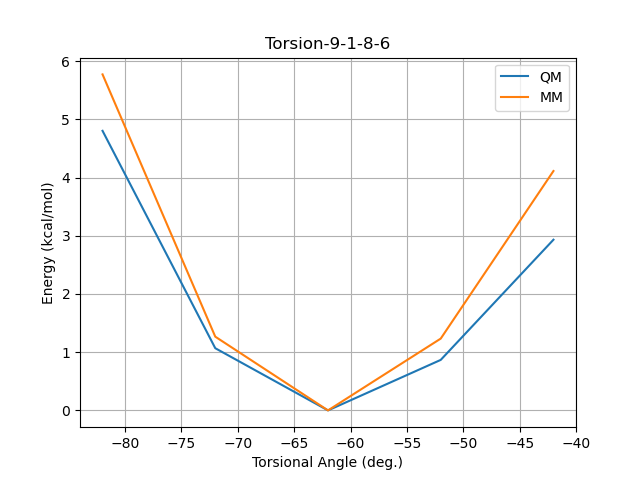

Supplement: SC-015-D4SC04364B-s002 [file SC-015-D4SC04364B-s002.zip › torsion_fit/AFUC/torsion_fitting-9-1-8-6.png]

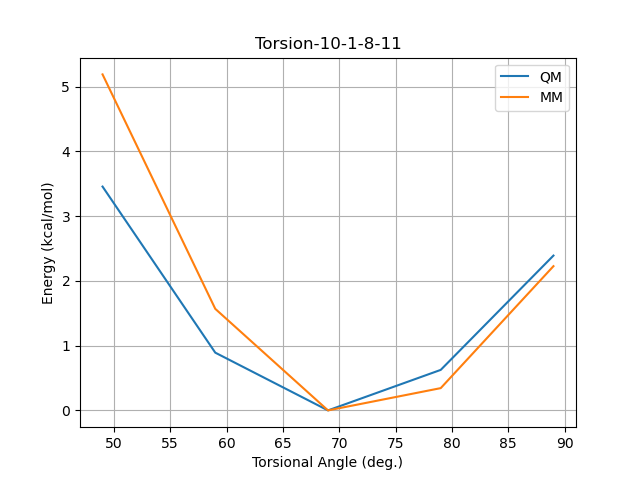

Supplement: SC-015-D4SC04364B-s002 [file SC-015-D4SC04364B-s002.zip › torsion_fit/BGAL/torsion_fitting-10-1-8-11.png]

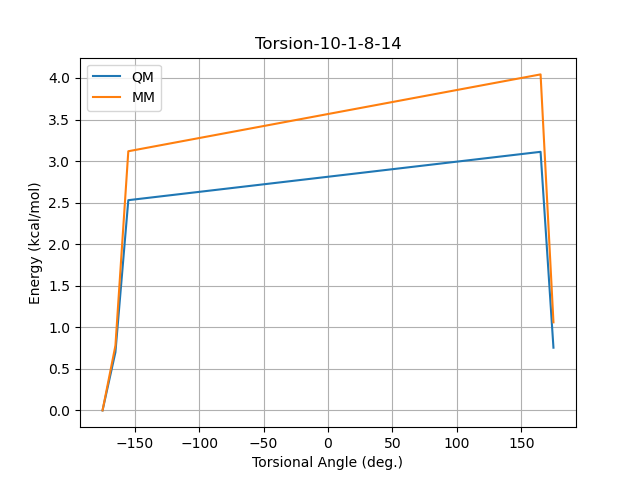

Supplement: SC-015-D4SC04364B-s002 [file SC-015-D4SC04364B-s002.zip › torsion_fit/BGAL/torsion_fitting-10-1-8-14.png]

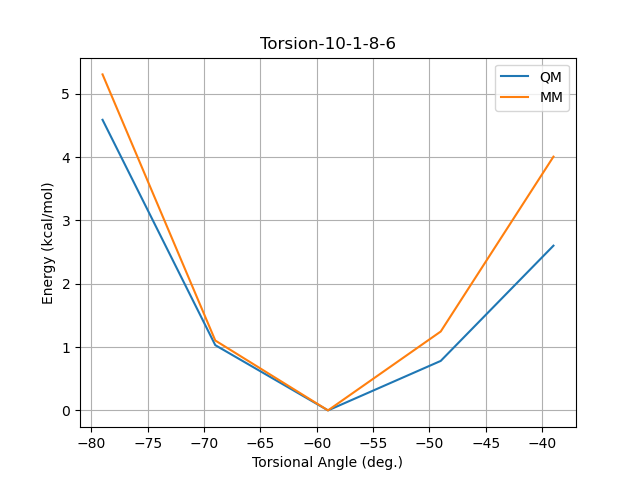

Supplement: SC-015-D4SC04364B-s002 [file SC-015-D4SC04364B-s002.zip › torsion_fit/BGAL/torsion_fitting-10-1-8-6.png]

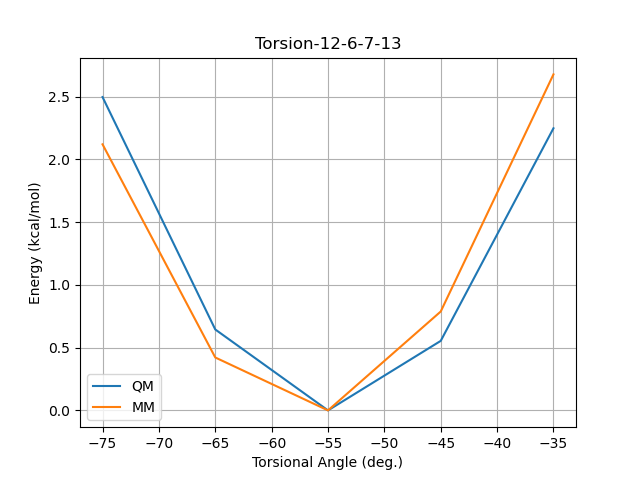

Supplement: SC-015-D4SC04364B-s002 [file SC-015-D4SC04364B-s002.zip › torsion_fit/BGAL/torsion_fitting-12-6-7-13.png]

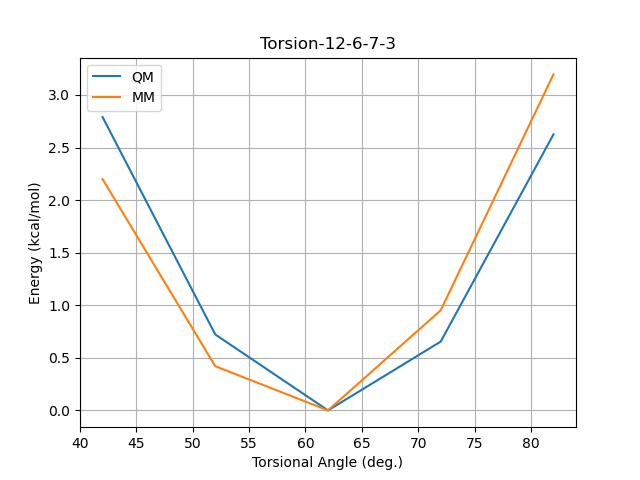

Supplement: SC-015-D4SC04364B-s002 [file SC-015-D4SC04364B-s002.zip › torsion_fit/BGAL/torsion_fitting-12-6-7-3.png]

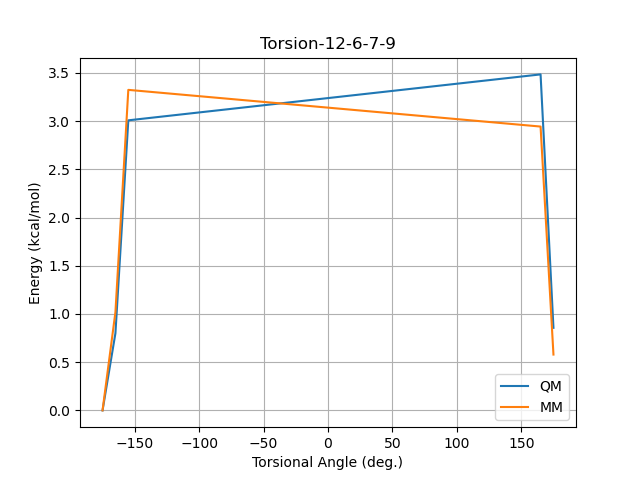

Supplement: SC-015-D4SC04364B-s002 [file SC-015-D4SC04364B-s002.zip › torsion_fit/BGAL/torsion_fitting-12-6-7-9.png]

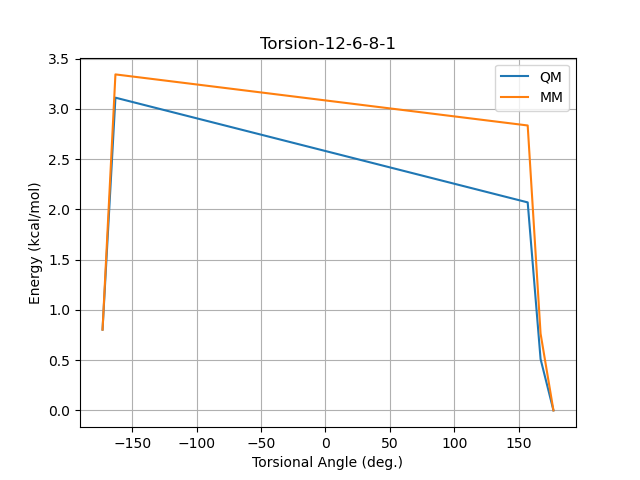

Supplement: SC-015-D4SC04364B-s002 [file SC-015-D4SC04364B-s002.zip › torsion_fit/BGAL/torsion_fitting-12-6-8-1.png]

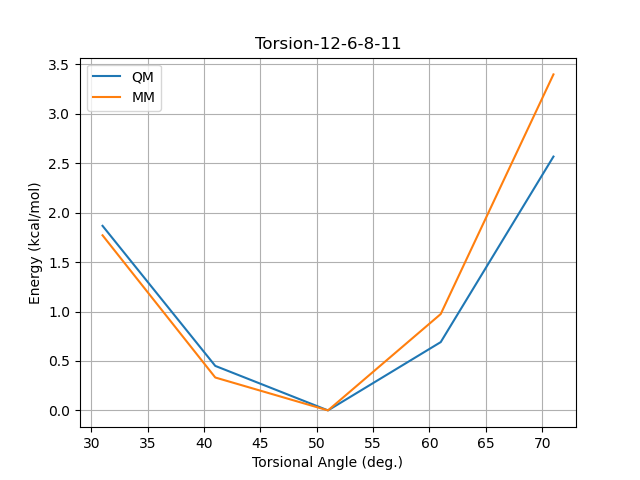

Supplement: SC-015-D4SC04364B-s002 [file SC-015-D4SC04364B-s002.zip › torsion_fit/BGAL/torsion_fitting-12-6-8-11.png]

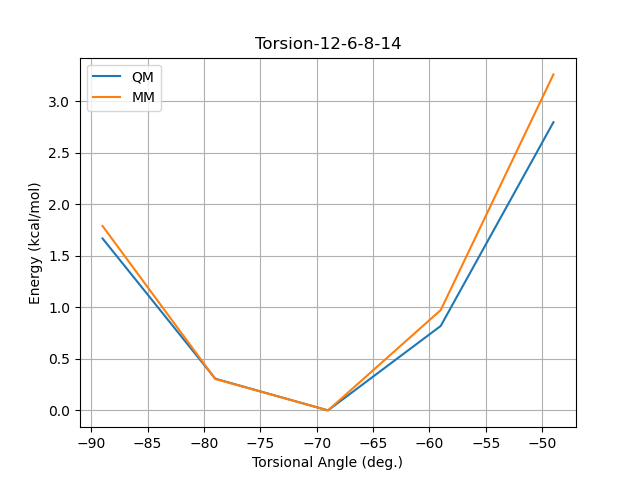

Supplement: SC-015-D4SC04364B-s002 [file SC-015-D4SC04364B-s002.zip › torsion_fit/BGAL/torsion_fitting-12-6-8-14.png]

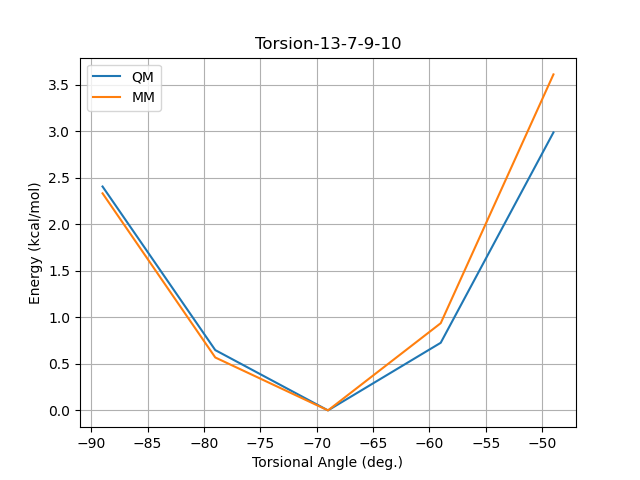

Supplement: SC-015-D4SC04364B-s002 [file SC-015-D4SC04364B-s002.zip › torsion_fit/BGAL/torsion_fitting-13-7-9-10.png]

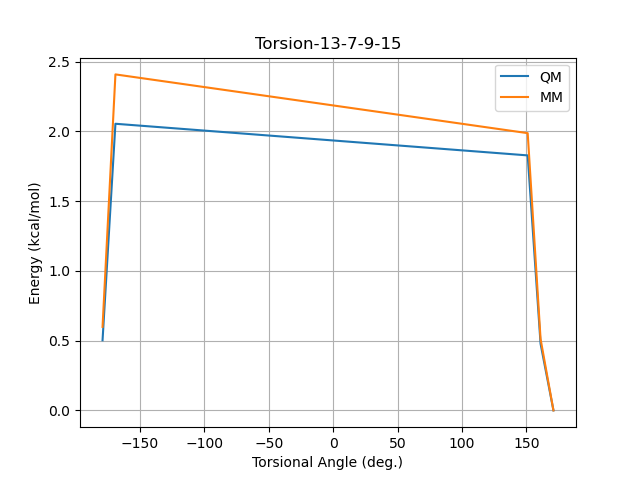

Supplement: SC-015-D4SC04364B-s002 [file SC-015-D4SC04364B-s002.zip › torsion_fit/BGAL/torsion_fitting-13-7-9-15.png]

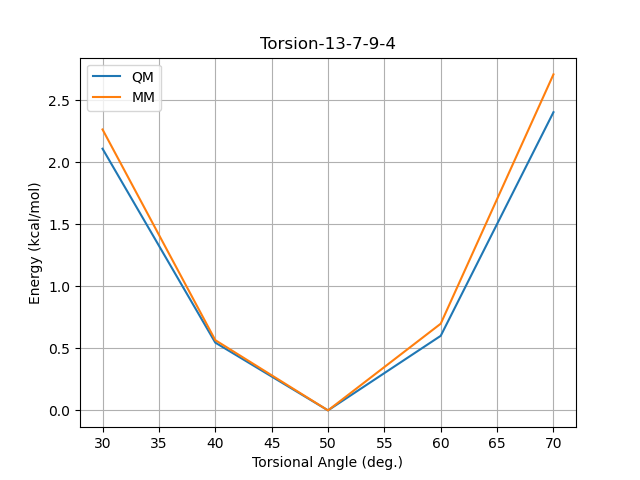

Supplement: SC-015-D4SC04364B-s002 [file SC-015-D4SC04364B-s002.zip › torsion_fit/BGAL/torsion_fitting-13-7-9-4.png]

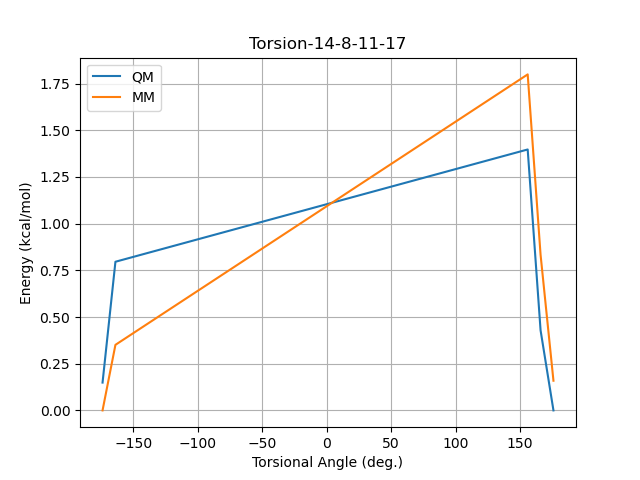

Supplement: SC-015-D4SC04364B-s002 [file SC-015-D4SC04364B-s002.zip › torsion_fit/BGAL/torsion_fitting-14-8-11-17.png]

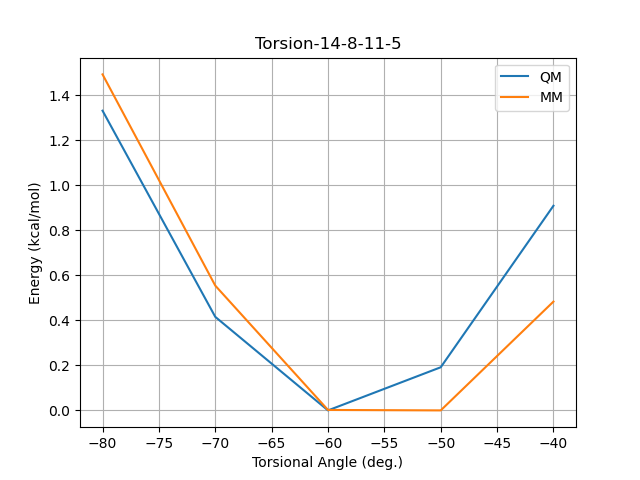

Supplement: SC-015-D4SC04364B-s002 [file SC-015-D4SC04364B-s002.zip › torsion_fit/BGAL/torsion_fitting-14-8-11-5.png]

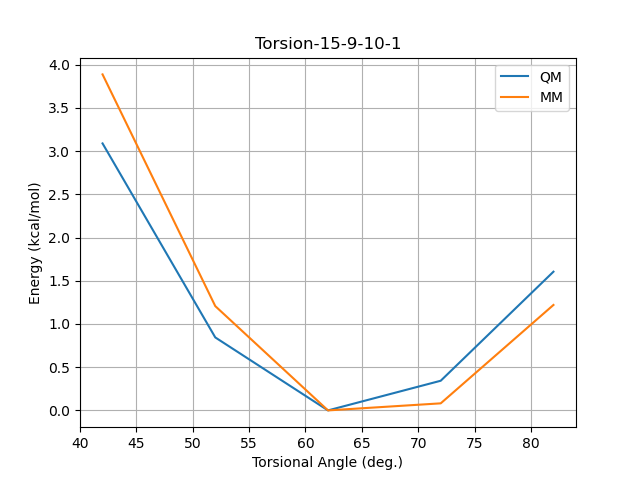

Supplement: SC-015-D4SC04364B-s002 [file SC-015-D4SC04364B-s002.zip › torsion_fit/BGAL/torsion_fitting-15-9-10-1.png]

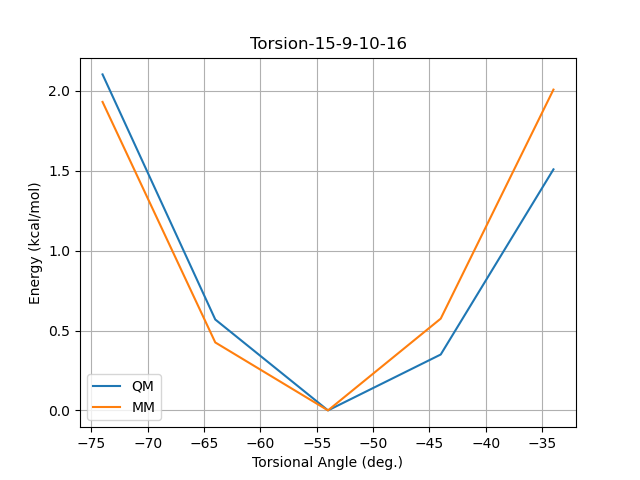

Supplement: SC-015-D4SC04364B-s002 [file SC-015-D4SC04364B-s002.zip › torsion_fit/BGAL/torsion_fitting-15-9-10-16.png]

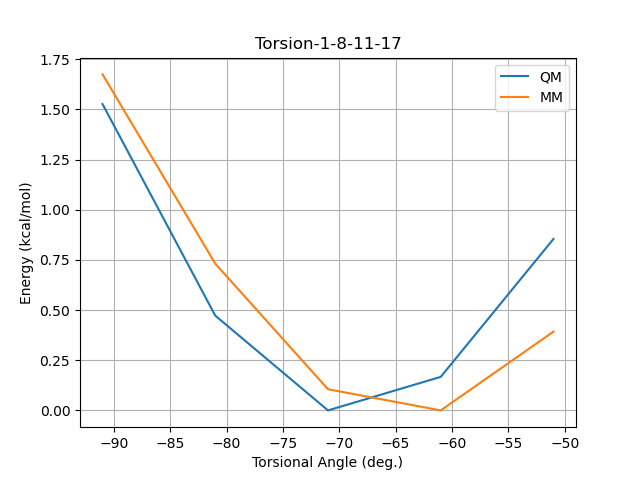

Supplement: SC-015-D4SC04364B-s002 [file SC-015-D4SC04364B-s002.zip › torsion_fit/BGAL/torsion_fitting-1-8-11-17.png]

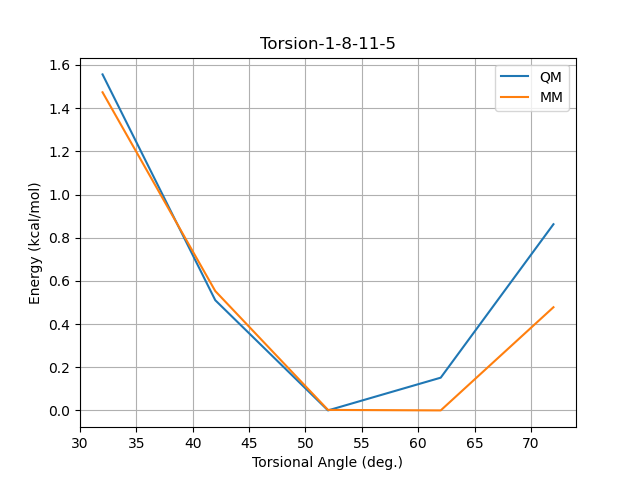

Supplement: SC-015-D4SC04364B-s002 [file SC-015-D4SC04364B-s002.zip › torsion_fit/BGAL/torsion_fitting-1-8-11-5.png]

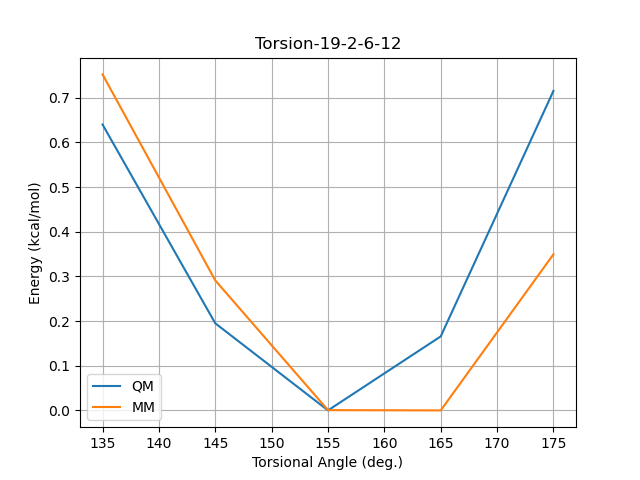

Supplement: SC-015-D4SC04364B-s002 [file SC-015-D4SC04364B-s002.zip › torsion_fit/BGAL/torsion_fitting-19-2-6-12.png]

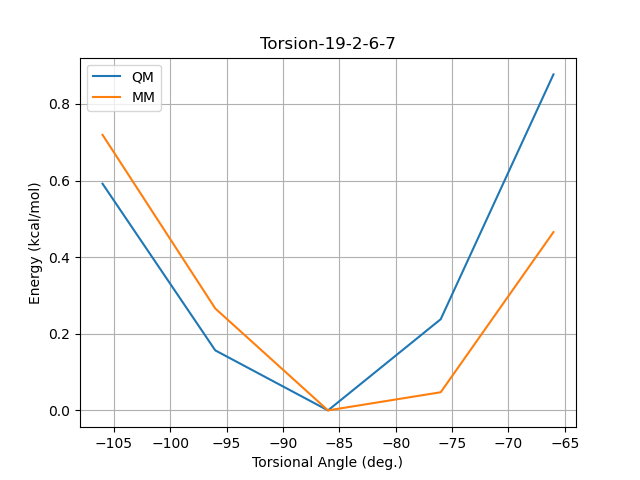

Supplement: SC-015-D4SC04364B-s002 [file SC-015-D4SC04364B-s002.zip › torsion_fit/BGAL/torsion_fitting-19-2-6-7.png]

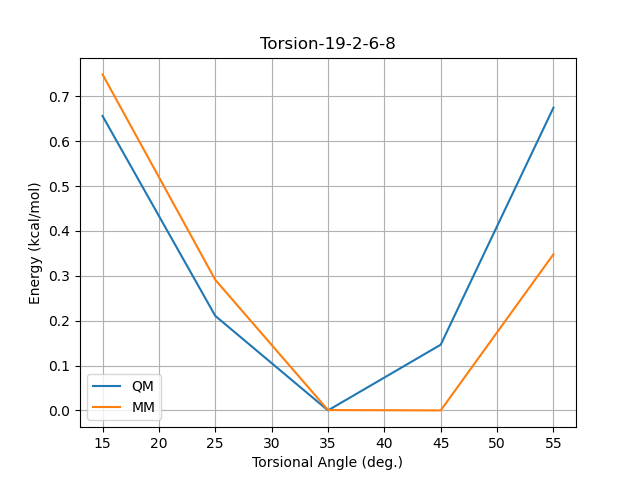

Supplement: SC-015-D4SC04364B-s002 [file SC-015-D4SC04364B-s002.zip › torsion_fit/BGAL/torsion_fitting-19-2-6-8.png]

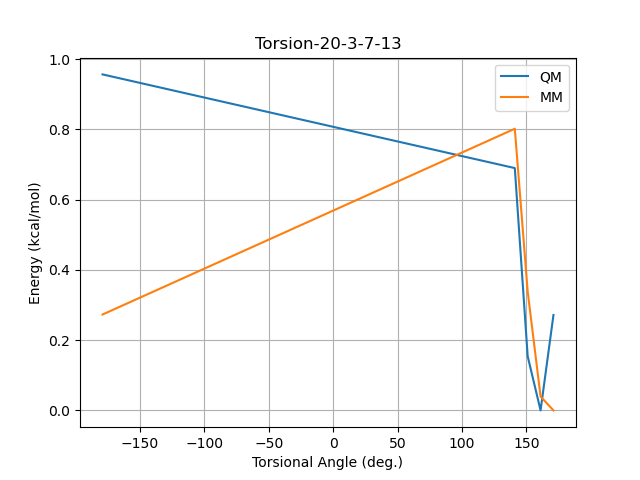

Supplement: SC-015-D4SC04364B-s002 [file SC-015-D4SC04364B-s002.zip › torsion_fit/BGAL/torsion_fitting-20-3-7-13.png]

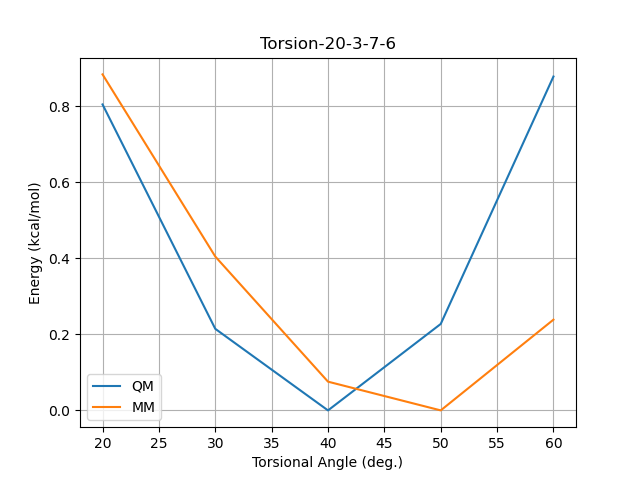

Supplement: SC-015-D4SC04364B-s002 [file SC-015-D4SC04364B-s002.zip › torsion_fit/BGAL/torsion_fitting-20-3-7-6.png]

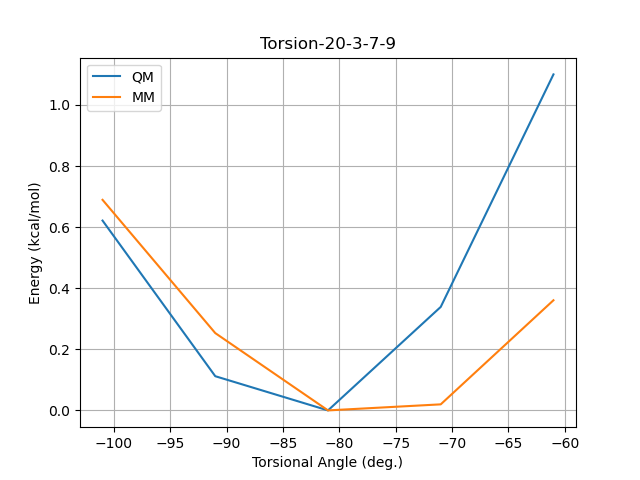

Supplement: SC-015-D4SC04364B-s002 [file SC-015-D4SC04364B-s002.zip › torsion_fit/BGAL/torsion_fitting-20-3-7-9.png]

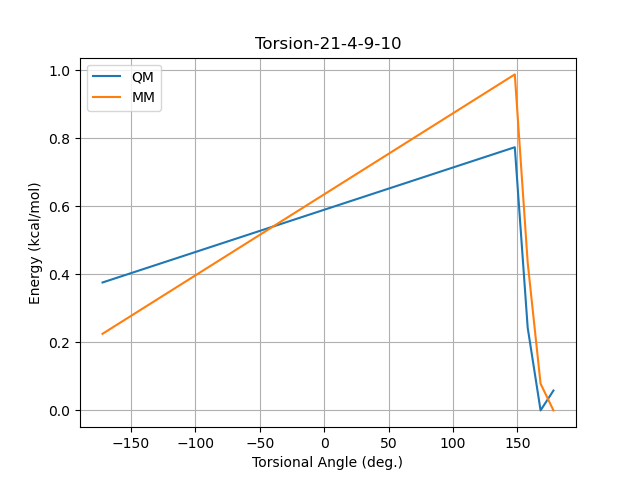

Supplement: SC-015-D4SC04364B-s002 [file SC-015-D4SC04364B-s002.zip › torsion_fit/BGAL/torsion_fitting-21-4-9-10.png]

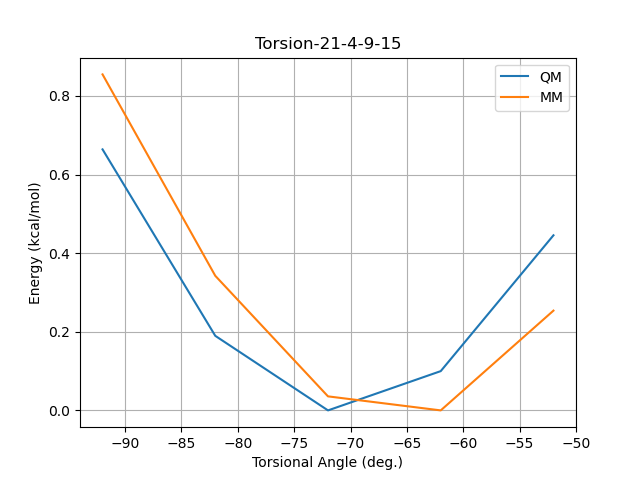

Supplement: SC-015-D4SC04364B-s002 [file SC-015-D4SC04364B-s002.zip › torsion_fit/BGAL/torsion_fitting-21-4-9-15.png]

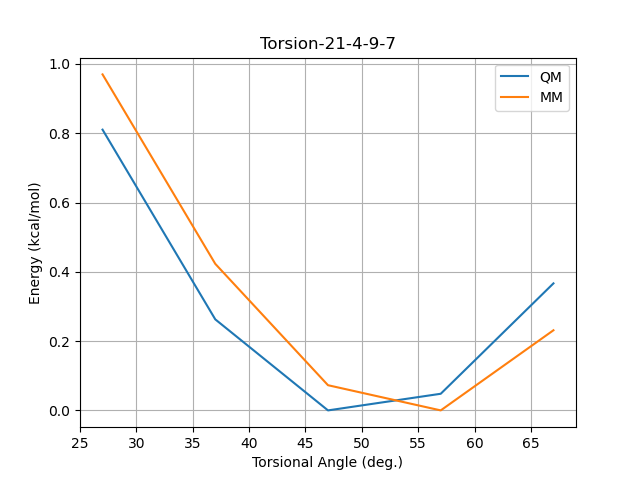

Supplement: SC-015-D4SC04364B-s002 [file SC-015-D4SC04364B-s002.zip › torsion_fit/BGAL/torsion_fitting-21-4-9-7.png]

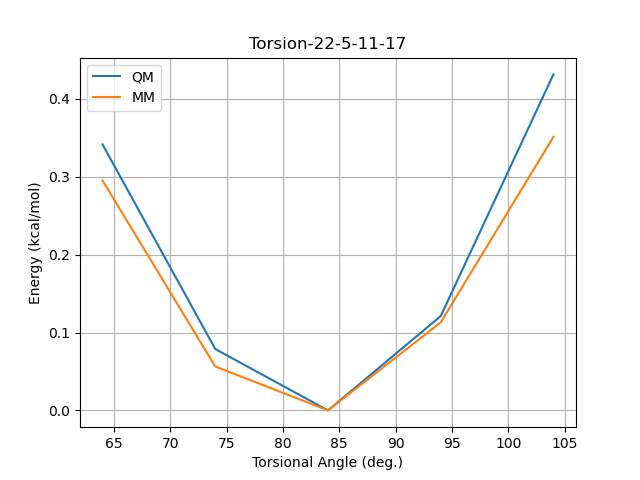

Supplement: SC-015-D4SC04364B-s002 [file SC-015-D4SC04364B-s002.zip › torsion_fit/BGAL/torsion_fitting-22-5-11-17.png]

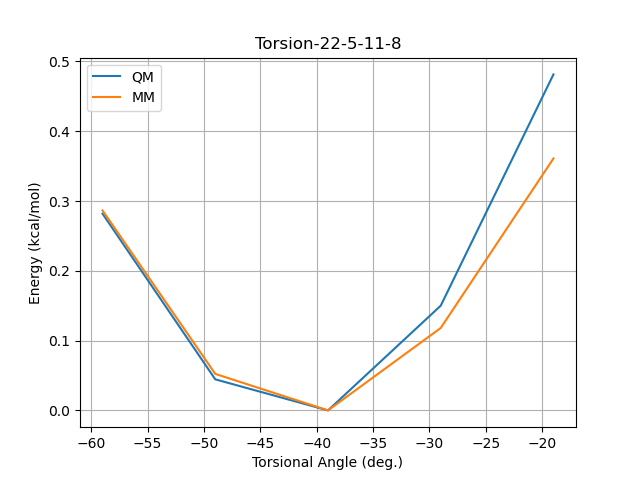

Supplement: SC-015-D4SC04364B-s002 [file SC-015-D4SC04364B-s002.zip › torsion_fit/BGAL/torsion_fitting-22-5-11-8.png]

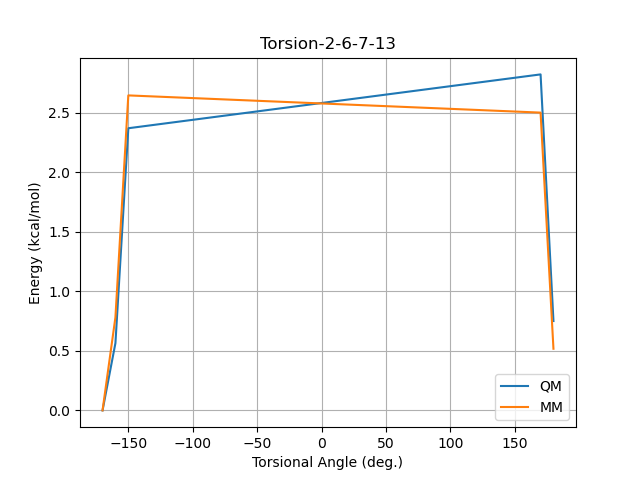

Supplement: SC-015-D4SC04364B-s002 [file SC-015-D4SC04364B-s002.zip › torsion_fit/BGAL/torsion_fitting-2-6-7-13.png]

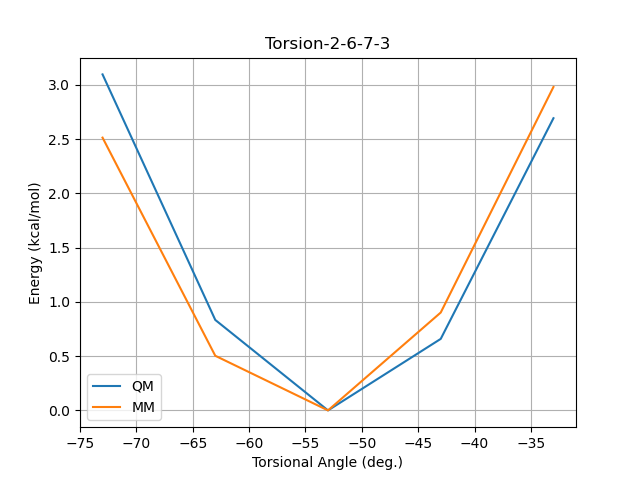

Supplement: SC-015-D4SC04364B-s002 [file SC-015-D4SC04364B-s002.zip › torsion_fit/BGAL/torsion_fitting-2-6-7-3.png]

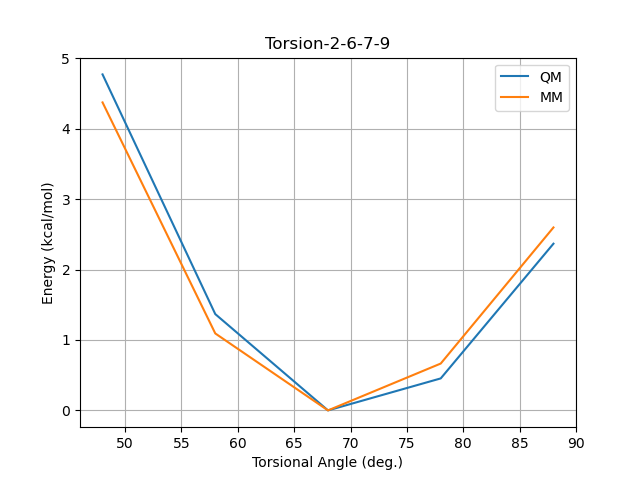

Supplement: SC-015-D4SC04364B-s002 [file SC-015-D4SC04364B-s002.zip › torsion_fit/BGAL/torsion_fitting-2-6-7-9.png]

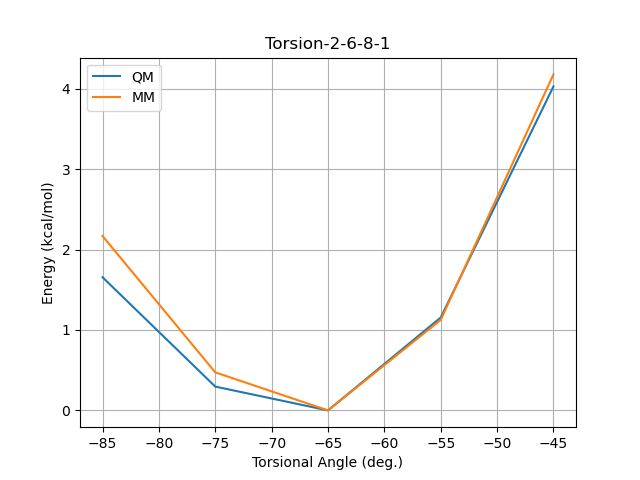

Supplement: SC-015-D4SC04364B-s002 [file SC-015-D4SC04364B-s002.zip › torsion_fit/BGAL/torsion_fitting-2-6-8-1.png]

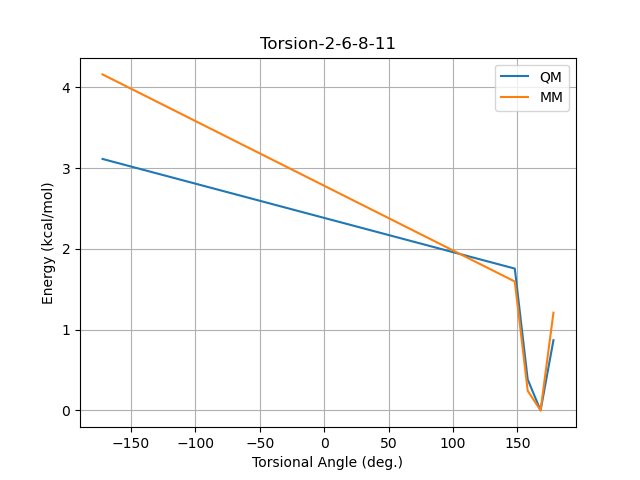

Supplement: SC-015-D4SC04364B-s002 [file SC-015-D4SC04364B-s002.zip › torsion_fit/BGAL/torsion_fitting-2-6-8-11.png]

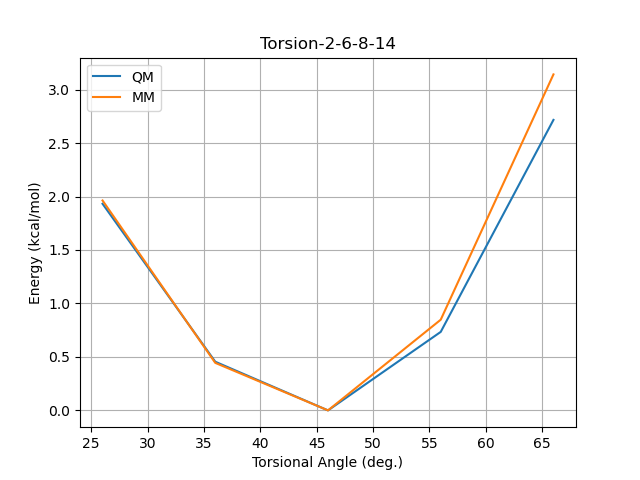

Supplement: SC-015-D4SC04364B-s002 [file SC-015-D4SC04364B-s002.zip › torsion_fit/BGAL/torsion_fitting-2-6-8-14.png]

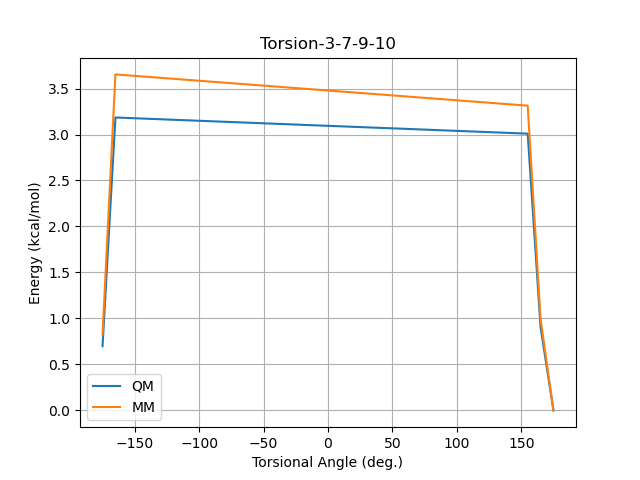

Supplement: SC-015-D4SC04364B-s002 [file SC-015-D4SC04364B-s002.zip › torsion_fit/BGAL/torsion_fitting-3-7-9-10.png]

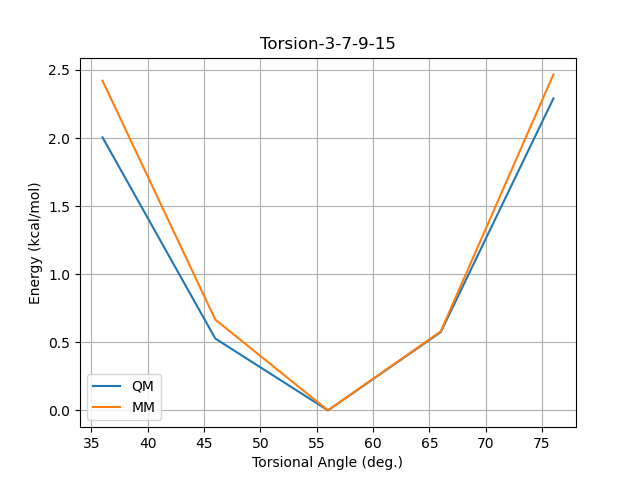

Supplement: SC-015-D4SC04364B-s002 [file SC-015-D4SC04364B-s002.zip › torsion_fit/BGAL/torsion_fitting-3-7-9-15.png]

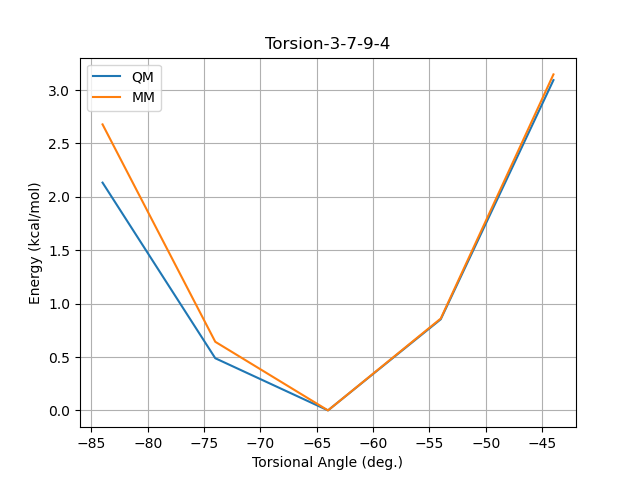

Supplement: SC-015-D4SC04364B-s002 [file SC-015-D4SC04364B-s002.zip › torsion_fit/BGAL/torsion_fitting-3-7-9-4.png]

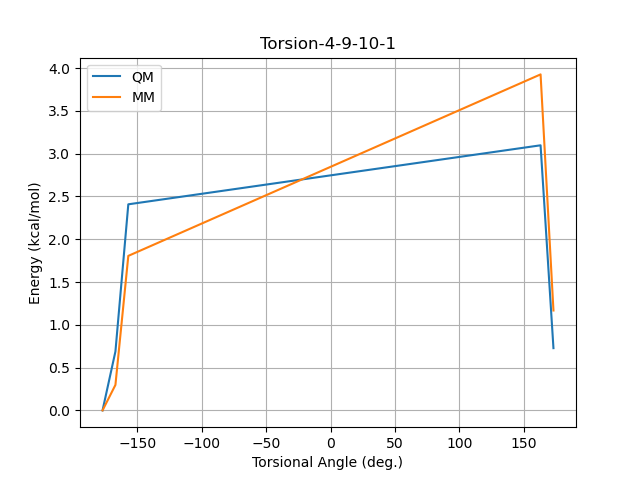

Supplement: SC-015-D4SC04364B-s002 [file SC-015-D4SC04364B-s002.zip › torsion_fit/BGAL/torsion_fitting-4-9-10-1.png]

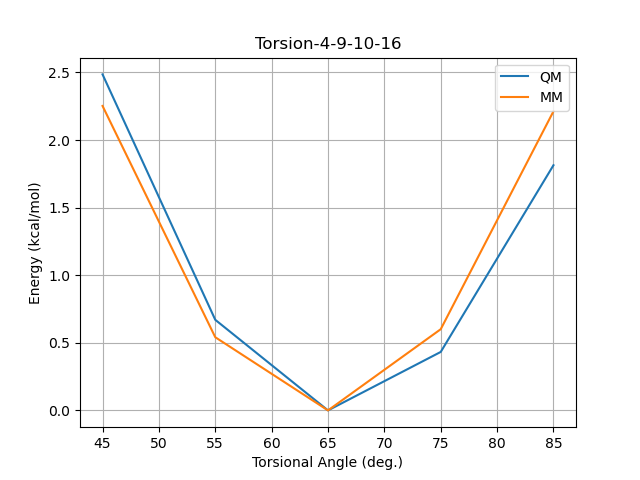

Supplement: SC-015-D4SC04364B-s002 [file SC-015-D4SC04364B-s002.zip › torsion_fit/BGAL/torsion_fitting-4-9-10-16.png]

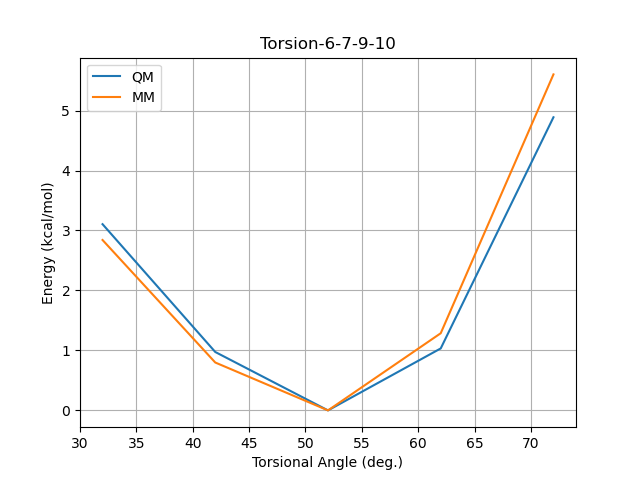

Supplement: SC-015-D4SC04364B-s002 [file SC-015-D4SC04364B-s002.zip › torsion_fit/BGAL/torsion_fitting-6-7-9-10.png]

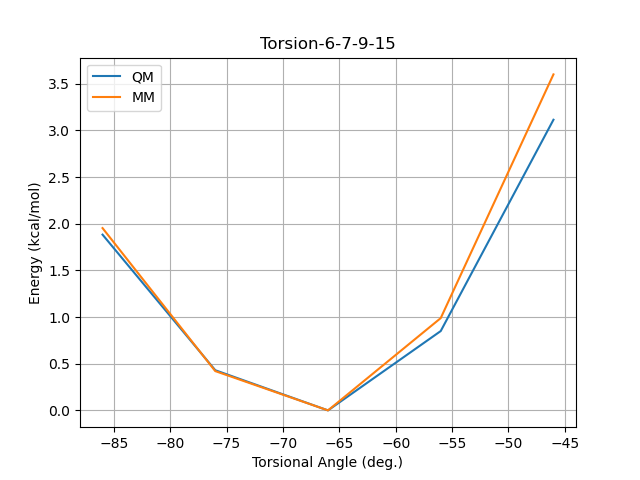

Supplement: SC-015-D4SC04364B-s002 [file SC-015-D4SC04364B-s002.zip › torsion_fit/BGAL/torsion_fitting-6-7-9-15.png]

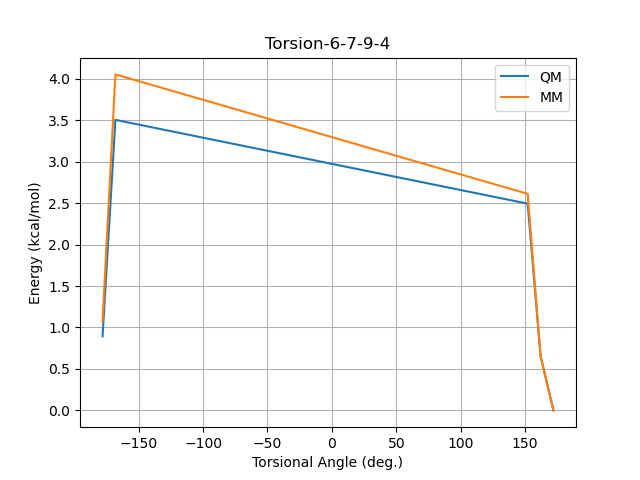

Supplement: SC-015-D4SC04364B-s002 [file SC-015-D4SC04364B-s002.zip › torsion_fit/BGAL/torsion_fitting-6-7-9-4.png]

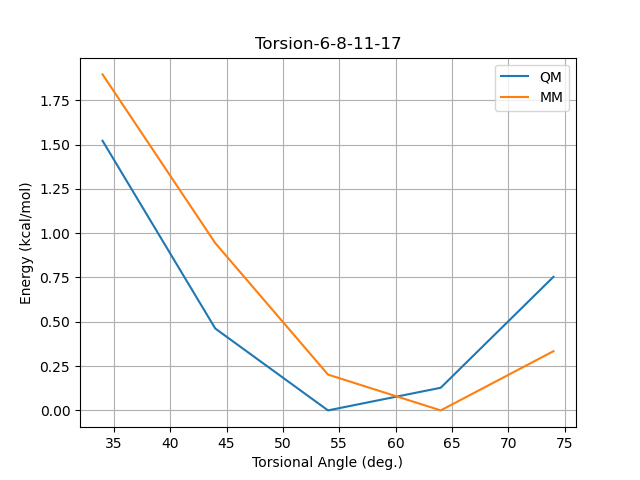

Supplement: SC-015-D4SC04364B-s002 [file SC-015-D4SC04364B-s002.zip › torsion_fit/BGAL/torsion_fitting-6-8-11-17.png]

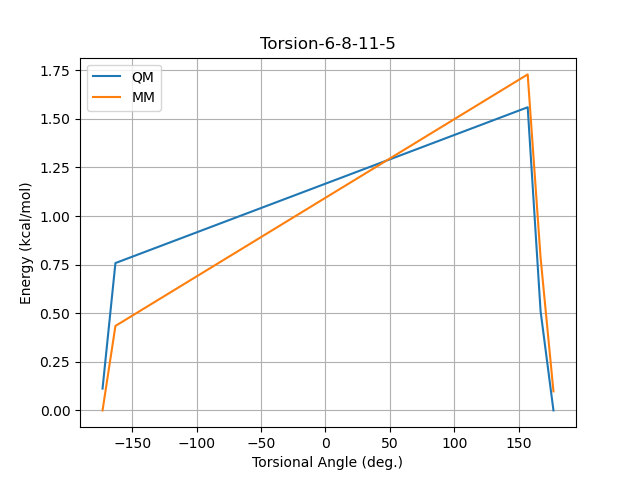

Supplement: SC-015-D4SC04364B-s002 [file SC-015-D4SC04364B-s002.zip › torsion_fit/BGAL/torsion_fitting-6-8-11-5.png]

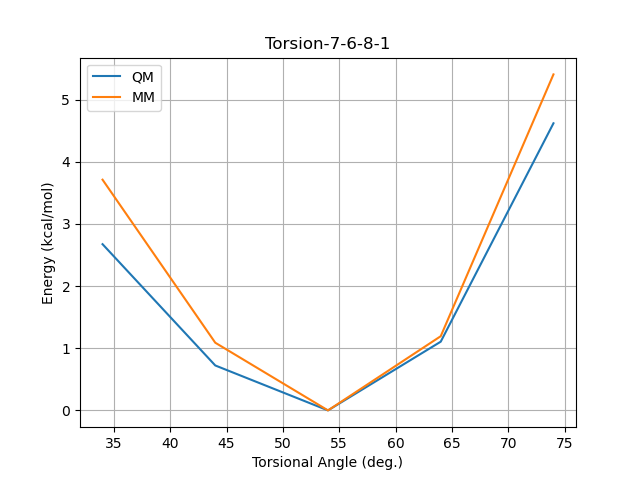

Supplement: SC-015-D4SC04364B-s002 [file SC-015-D4SC04364B-s002.zip › torsion_fit/BGAL/torsion_fitting-7-6-8-1.png]

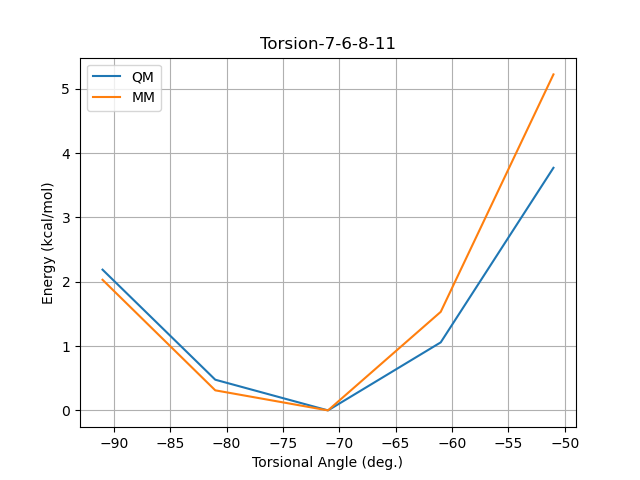

Supplement: SC-015-D4SC04364B-s002 [file SC-015-D4SC04364B-s002.zip › torsion_fit/BGAL/torsion_fitting-7-6-8-11.png]

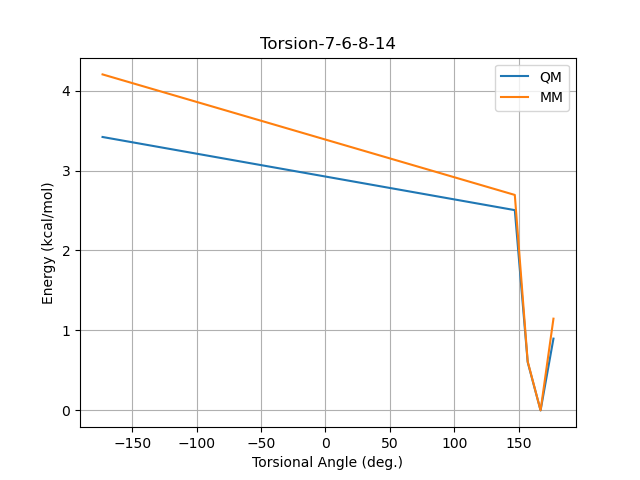

Supplement: SC-015-D4SC04364B-s002 [file SC-015-D4SC04364B-s002.zip › torsion_fit/BGAL/torsion_fitting-7-6-8-14.png]

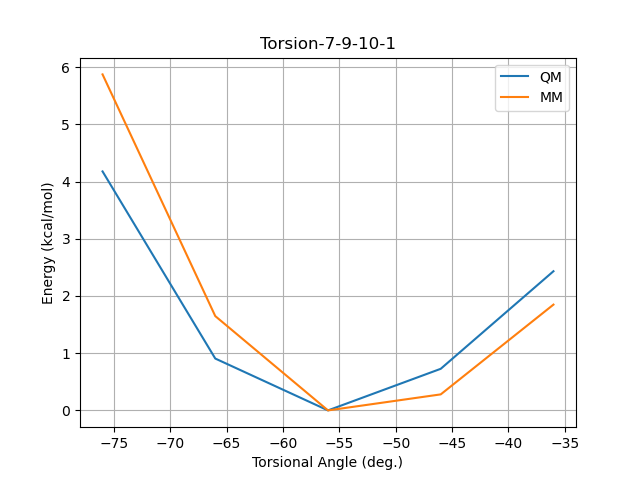

Supplement: SC-015-D4SC04364B-s002 [file SC-015-D4SC04364B-s002.zip › torsion_fit/BGAL/torsion_fitting-7-9-10-1.png]

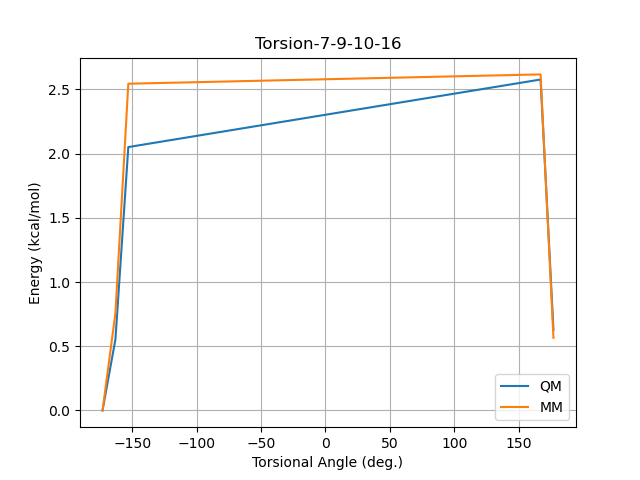

Supplement: SC-015-D4SC04364B-s002 [file SC-015-D4SC04364B-s002.zip › torsion_fit/BGAL/torsion_fitting-7-9-10-16.png]

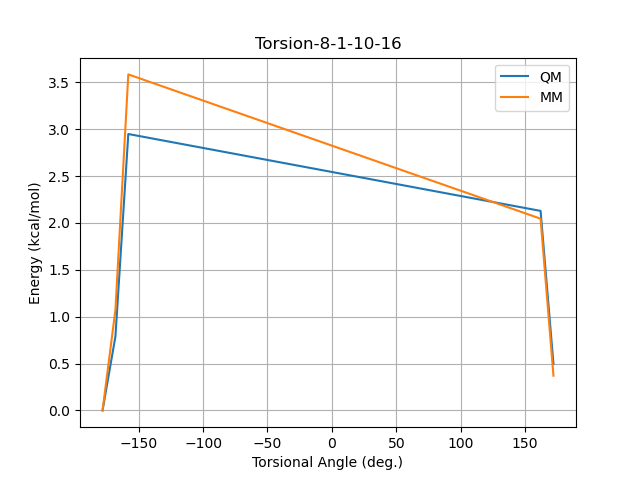

Supplement: SC-015-D4SC04364B-s002 [file SC-015-D4SC04364B-s002.zip › torsion_fit/BGAL/torsion_fitting-8-1-10-16.png]

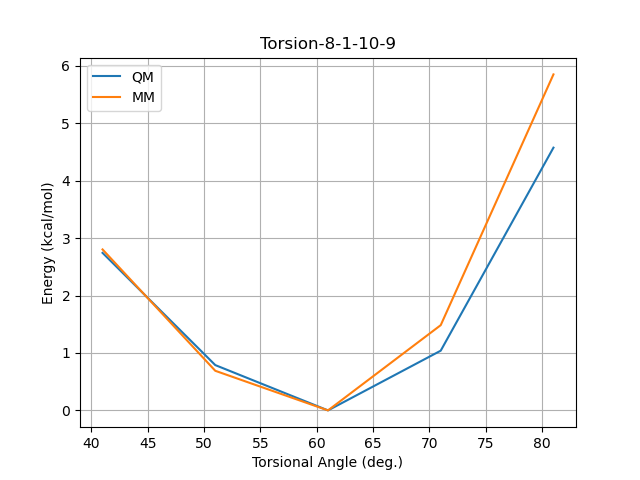

Supplement: SC-015-D4SC04364B-s002 [file SC-015-D4SC04364B-s002.zip › torsion_fit/BGAL/torsion_fitting-8-1-10-9.png]

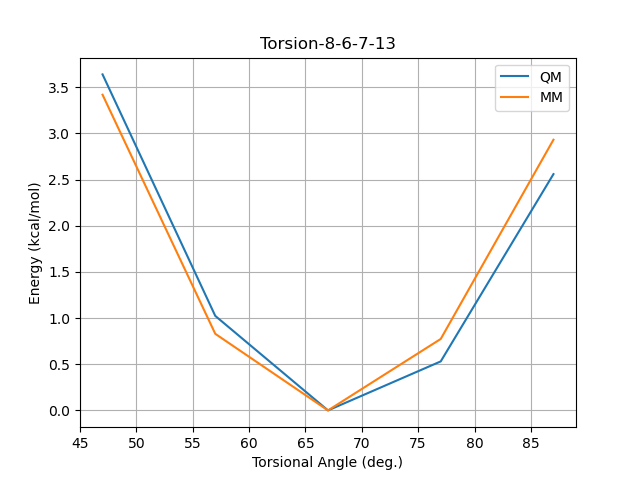

Supplement: SC-015-D4SC04364B-s002 [file SC-015-D4SC04364B-s002.zip › torsion_fit/BGAL/torsion_fitting-8-6-7-13.png]

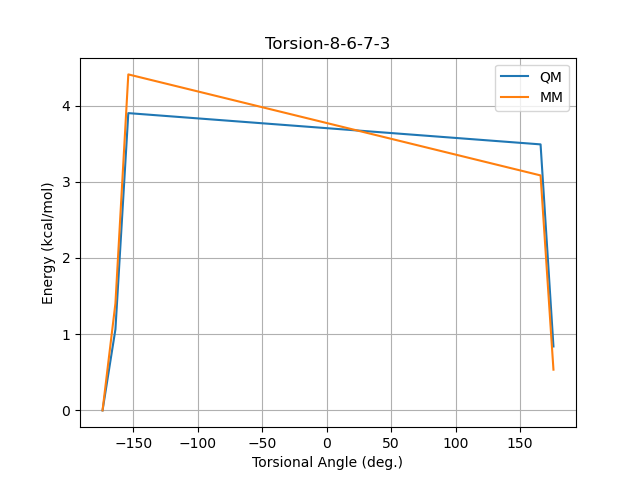

Supplement: SC-015-D4SC04364B-s002 [file SC-015-D4SC04364B-s002.zip › torsion_fit/BGAL/torsion_fitting-8-6-7-3.png]

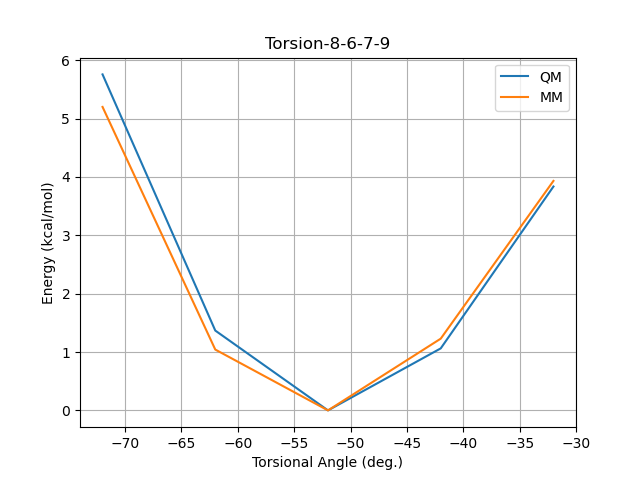

Supplement: SC-015-D4SC04364B-s002 [file SC-015-D4SC04364B-s002.zip › torsion_fit/BGAL/torsion_fitting-8-6-7-9.png]

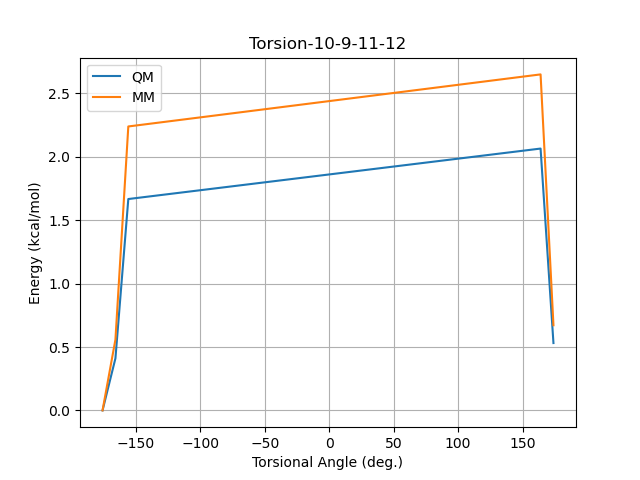

Supplement: SC-015-D4SC04364B-s002 [file SC-015-D4SC04364B-s002.zip › torsion_fit/BGLN/torsion_fitting-10-9-11-12.png]

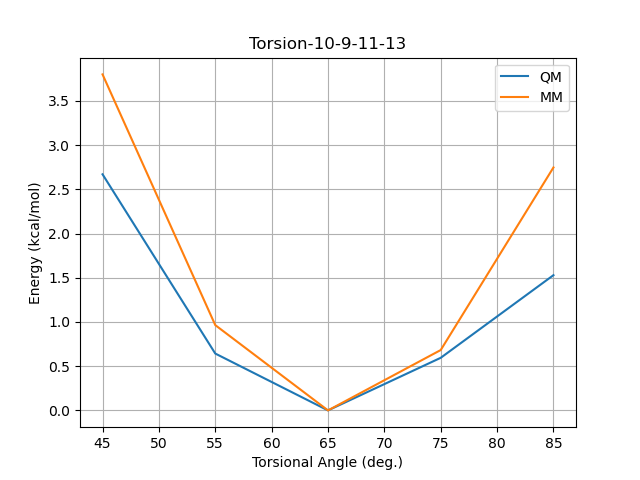

Supplement: SC-015-D4SC04364B-s002 [file SC-015-D4SC04364B-s002.zip › torsion_fit/BGLN/torsion_fitting-10-9-11-13.png]

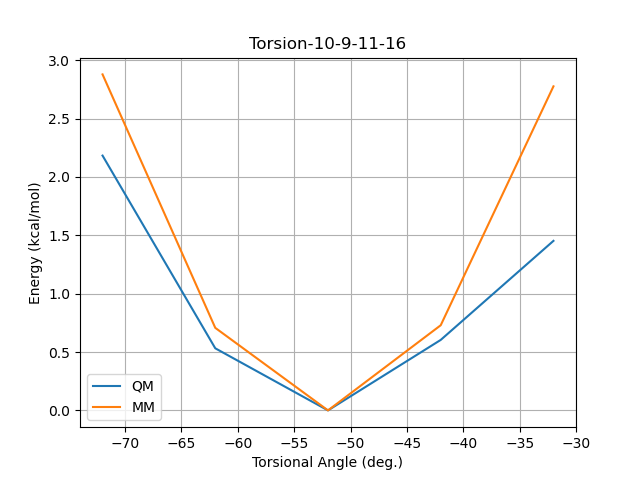

Supplement: SC-015-D4SC04364B-s002 [file SC-015-D4SC04364B-s002.zip › torsion_fit/BGLN/torsion_fitting-10-9-11-16.png]

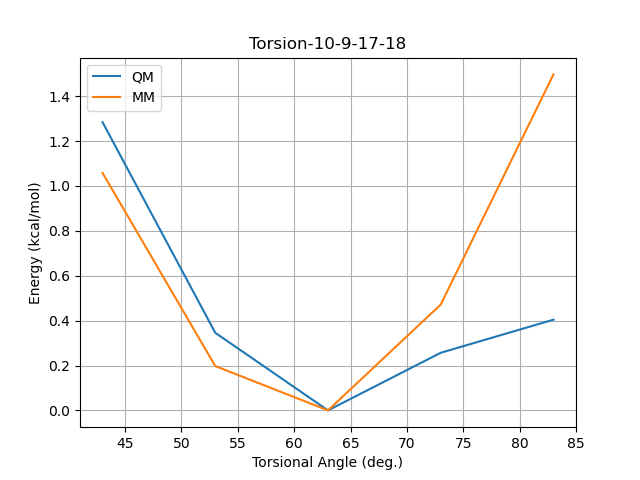

Supplement: SC-015-D4SC04364B-s002 [file SC-015-D4SC04364B-s002.zip › torsion_fit/BGLN/torsion_fitting-10-9-17-18.png]

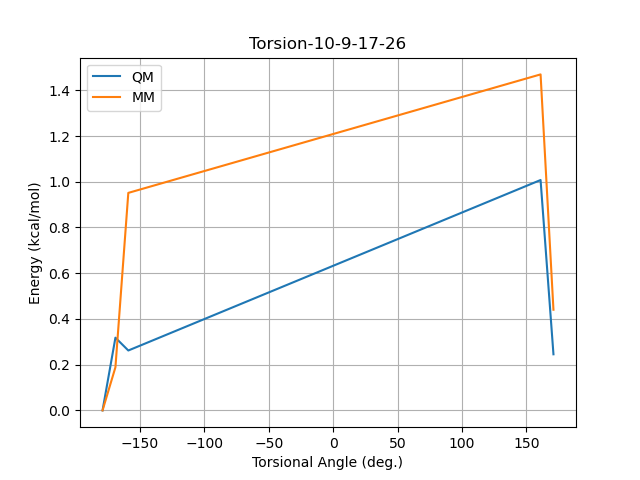

Supplement: SC-015-D4SC04364B-s002 [file SC-015-D4SC04364B-s002.zip › torsion_fit/BGLN/torsion_fitting-10-9-17-26.png]

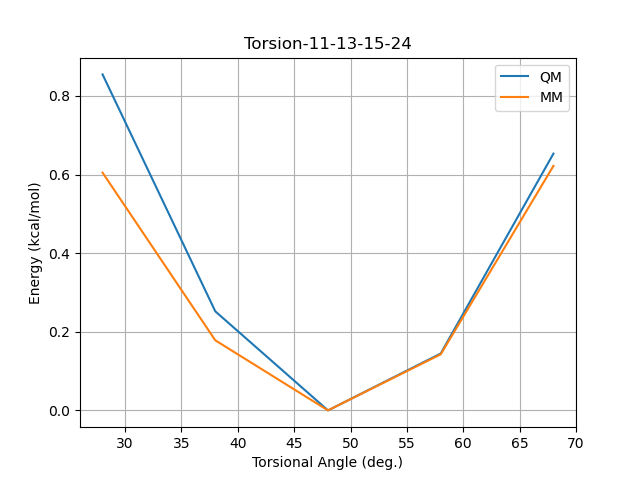

Supplement: SC-015-D4SC04364B-s002 [file SC-015-D4SC04364B-s002.zip › torsion_fit/BGLN/torsion_fitting-11-13-15-24.png]

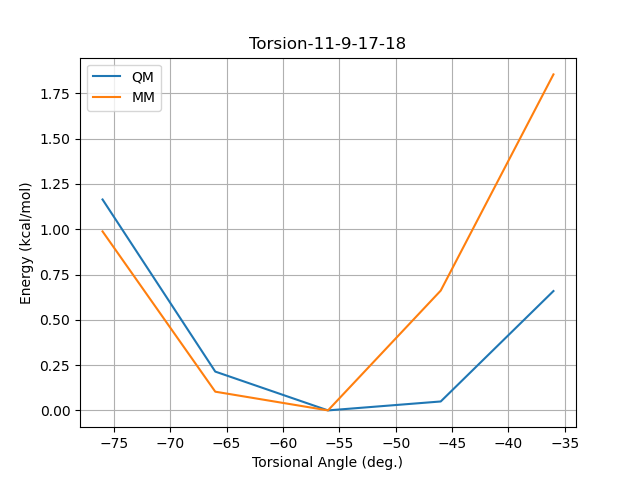

Supplement: SC-015-D4SC04364B-s002 [file SC-015-D4SC04364B-s002.zip › torsion_fit/BGLN/torsion_fitting-11-9-17-18.png]

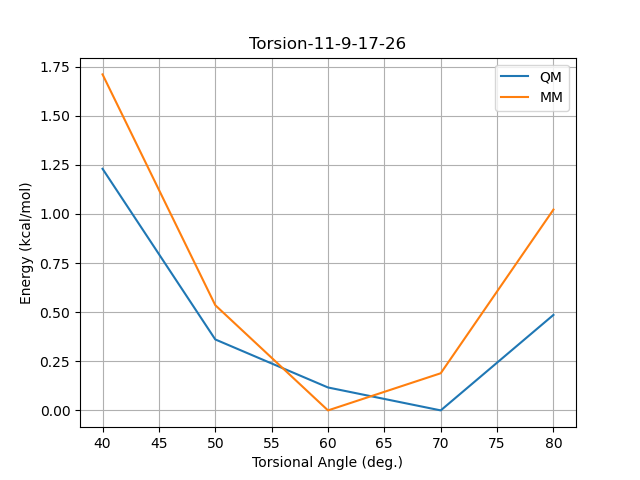

Supplement: SC-015-D4SC04364B-s002 [file SC-015-D4SC04364B-s002.zip › torsion_fit/BGLN/torsion_fitting-11-9-17-26.png]

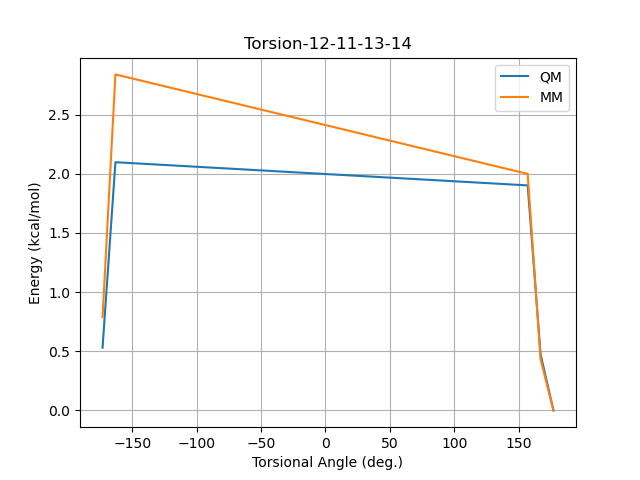

Supplement: SC-015-D4SC04364B-s002 [file SC-015-D4SC04364B-s002.zip › torsion_fit/BGLN/torsion_fitting-12-11-13-14.png]

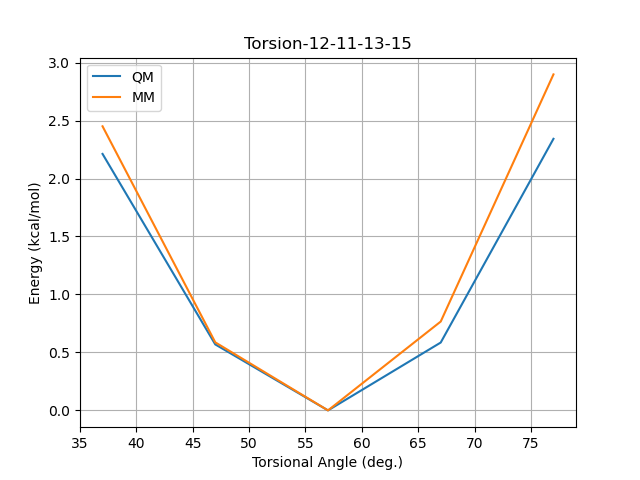

Supplement: SC-015-D4SC04364B-s002 [file SC-015-D4SC04364B-s002.zip › torsion_fit/BGLN/torsion_fitting-12-11-13-15.png]

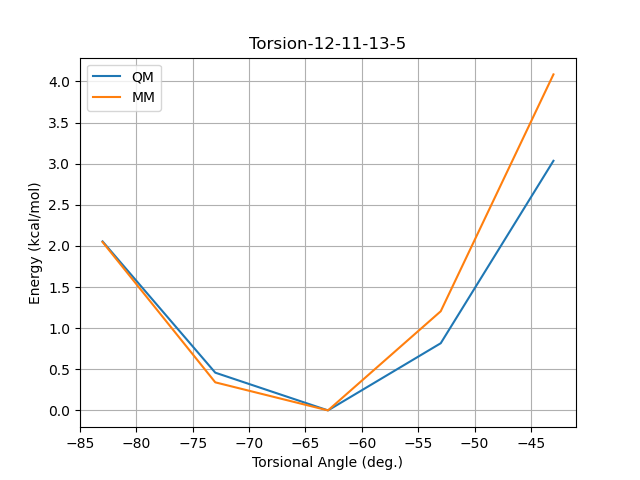

Supplement: SC-015-D4SC04364B-s002 [file SC-015-D4SC04364B-s002.zip › torsion_fit/BGLN/torsion_fitting-12-11-13-5.png]

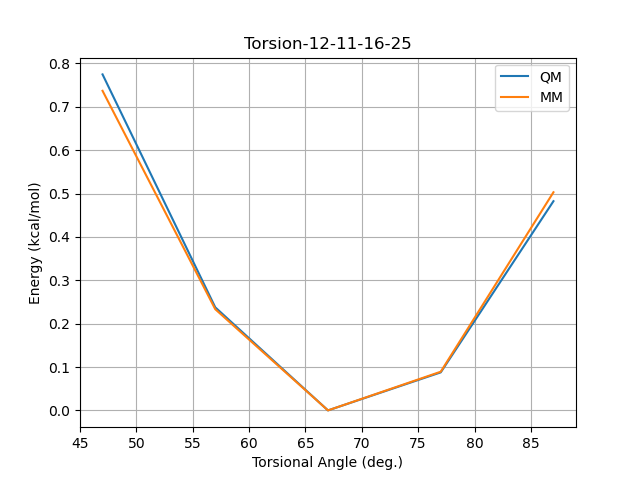

Supplement: SC-015-D4SC04364B-s002 [file SC-015-D4SC04364B-s002.zip › torsion_fit/BGLN/torsion_fitting-12-11-16-25.png]

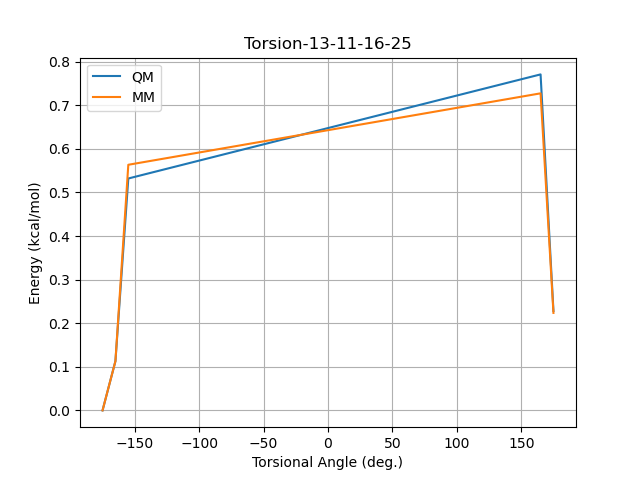

Supplement: SC-015-D4SC04364B-s002 [file SC-015-D4SC04364B-s002.zip › torsion_fit/BGLN/torsion_fitting-13-11-16-25.png]

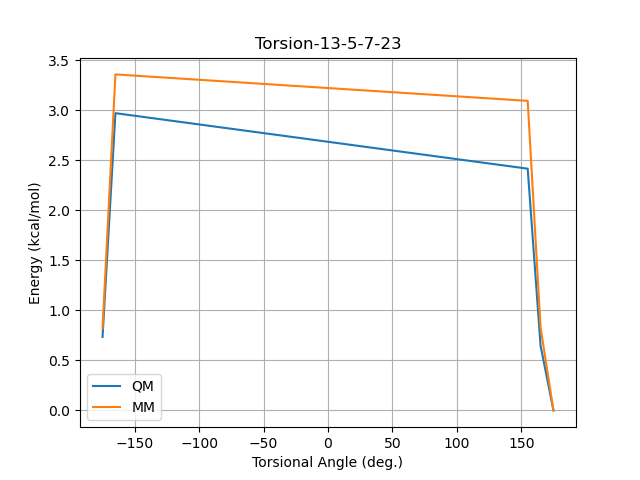

Supplement: SC-015-D4SC04364B-s002 [file SC-015-D4SC04364B-s002.zip › torsion_fit/BGLN/torsion_fitting-13-5-7-23.png]

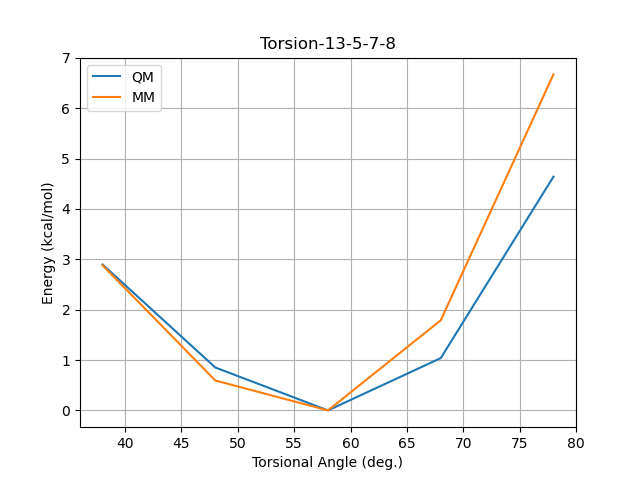

Supplement: SC-015-D4SC04364B-s002 [file SC-015-D4SC04364B-s002.zip › torsion_fit/BGLN/torsion_fitting-13-5-7-8.png]

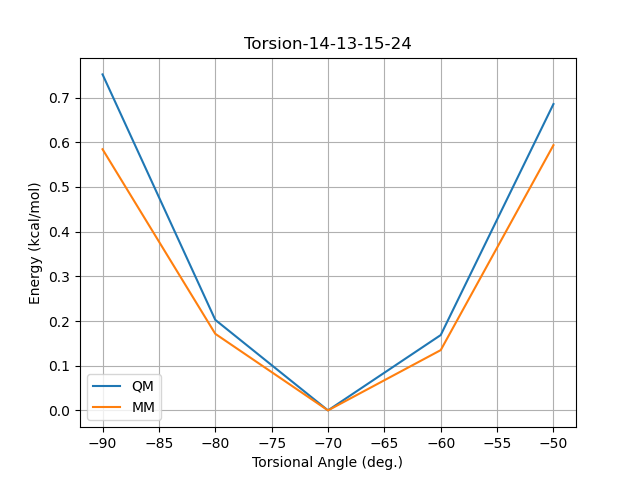

Supplement: SC-015-D4SC04364B-s002 [file SC-015-D4SC04364B-s002.zip › torsion_fit/BGLN/torsion_fitting-14-13-15-24.png]

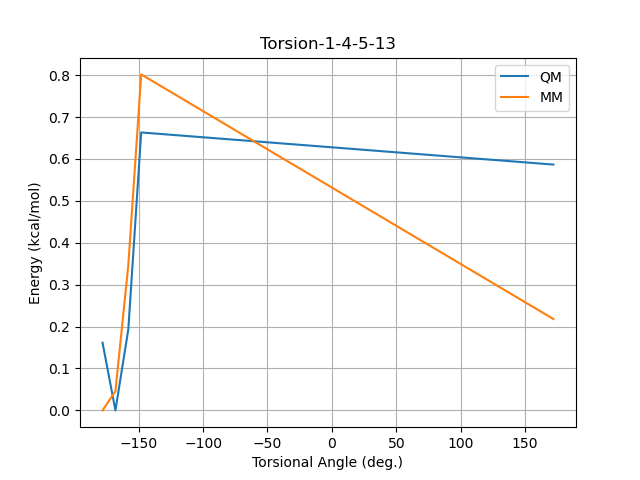

Supplement: SC-015-D4SC04364B-s002 [file SC-015-D4SC04364B-s002.zip › torsion_fit/BGLN/torsion_fitting-1-4-5-13.png]

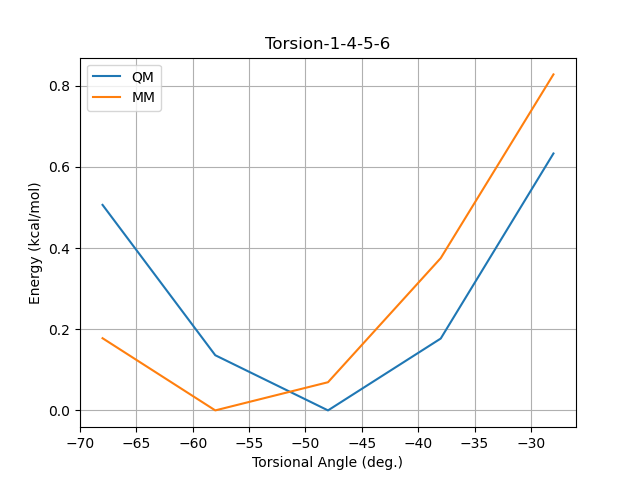

Supplement: SC-015-D4SC04364B-s002 [file SC-015-D4SC04364B-s002.zip › torsion_fit/BGLN/torsion_fitting-1-4-5-6.png]

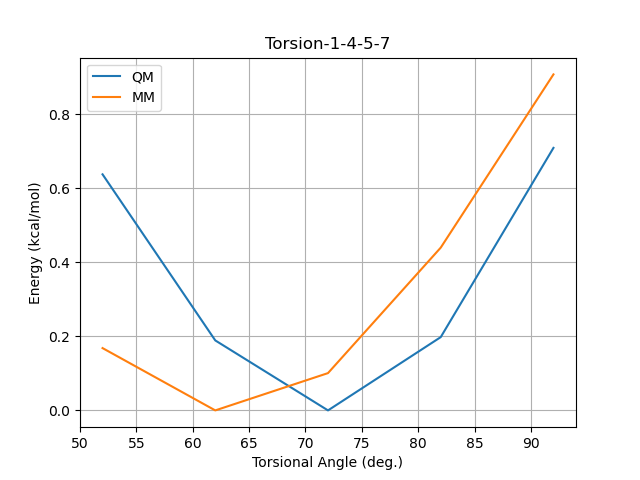

Supplement: SC-015-D4SC04364B-s002 [file SC-015-D4SC04364B-s002.zip › torsion_fit/BGLN/torsion_fitting-1-4-5-7.png]
