# Supplementary material for: Water–glycan interactions drive the SARS-CoV-2 spike dynamics: insights into glycan-gate control and camouflage mechanisms
Source: Chem Sci. 2024 Aug 23;15(35):14177–87. doi: 10.1039/d4sc04364b (PMC11359970; doi:10.1039/d4sc04364b)
Supplement: SC-015-D4SC04364B-s005 [file SC-015-D4SC04364B-s005.pdf]

Supplementary Information (SI) for Chemical Science.
This journal is © The Royal Society of Chemistry 2024

Supporting Information: Water-Glycan Interactions Drive the SARS-CoV-2 Spike Dynamics: Insights into Glycan-Gate Control and Camouflage Mechanisms

Marharyta Blazhynska,[†] Louis Lagardère,[*,†] Chengwen Liu,[‡,¶] Olivier Adjoua,[†] Pengyu Ren,[‡] and Jean-Philip Piquemal[*,†]

†*Laboratoire de chimie théorique, Sorbonne Université, UMR 7616 CNRS, 75005 Paris, France*

‡*Department of Biomedical Engineering, The University of Texas at Austin, Texas 78712, USA*

¶*Qubit Pharmaceuticals, 75014 Paris, France*

E-mail: louis.lagardere@sorbonne-universite.fr; jean-philip.piquemal@sorbonne-universite.fr

In addition to this **pdf**, the Supplementary Information includes:

- Data S1: Calculated inner protein hydrogen bonds for open and closed states.
- Data S2: Parametrization details for glycans, lipids.
- Data S3: Structure files for closed state.
- Data S4: Structure files for open state.
- Movie S1: Artistical visualization of the open and closed state systems used in our study.

Statistical Analysis Details

In this section, we share the details of statistical analysis presented in this work.

Principal Component Analysis

The PCA analysis described in Results section was performed using Python libraries such as NumPy[1], MDTraj[2], Matplotlib[3], Scikit-learn[4], and SciPy[5]. Initially, we obtained trajectories of the spike protein from simulations available on the Amaro Lab website[6]. Specifically, these trajectories were sourced from the work of Casalino et al.[7]. The protein topology and scaffold residues were defined based on the Amaro PDB files corresponding to both open and closed states. Subsequently, the spike protein trajectories were aligned to the central scaffold residues, with a focus on the C_α atom indices. Next, we applied PCA to the aligned trajectories to extract the dominant modes of motion exhibited by the spike protein. The PCA analysis was conducted using the Scikit-learn library, allowing us to compute the resulting principal components (PCs) and their corresponding explained variance ratios. The PCA coordinates were then saved for subsequent projection of our trajectories onto the obtained PCA space. To further characterize the distribution of conformations in the PCA space, kernel density estimation (KDE) was employed. Specifically, KDE was applied to each dimension of the PCA scores using the Gaussian KDE function from the

SciPy library, facilitating the estimation of the probability density function of the data, providing valuable insights into the spatial distribution of spike protein conformations. The overall density at each point in the PCA space was calculated by multiplying the densities along each dimension, after which the density values were normalized. These normalized density values were utilized to generate a heatmap-style PCA plot, where each point represented a distinct spike protein conformation. The color of each point on the plot corresponded to the normalized density at that location, offering visual cues regarding the density distribution across the PCA space. The resulting plot was visualized using Matplotlib, and a color bar was included to indicate the density scale.

Root Mean Square Fluctuation

The Root Mean Square Fluctuation (RMSF) of protein C_α atoms was computed to assess the dynamic behavior of the SARS-CoV-2 spike protein. Utilizing the Visual Molecular Dynamics (VMD) software package[8], the total number of frames in the molecular system was determined. The RMSF calculation was then performed using the **measure rmsf** utility available in VMD, which generated RMSF values for each C_α atom over the trajectory frames.

Hydrogen Bond and Salt Bridge Analysis

The hydrogen bond and salt bridge analysis were conducted using VMD software environment[8] throughout the concatenated simulation trajectories for both open and closed states. For the hydrogen bond analysis, hydrogen bonds between protein residues were identified based on geometric criteria such as donor-acceptor distance (3 Å) and hydrogen-donor-acceptor angle (20°). Similarly, for the salt bridge analysis, interactions between positively and negatively charged protein residues were examined with an oxygen-nitrogen distance cut-off of 3.2 Å.

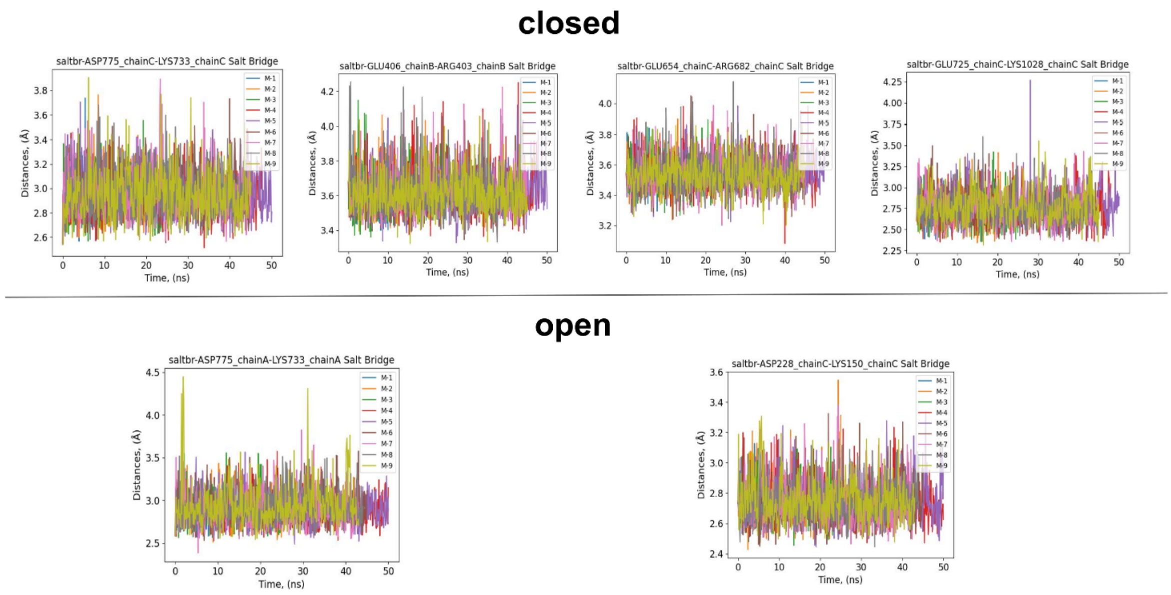

Fig. S1. Stable salt bridges in the SARS-CoV-2 spike protein structure, shown for the closed (upper panel) and open (lower panel) conformations across all macro-iterative simulations. These simulations were conducted using the polarizable AMOEBA force field and density-driven conformational sampling, as detailed in the Methods section of the main text (M- refers to the macroiteration). Chains A, B, and C represent the trimeric configuration of the protein.

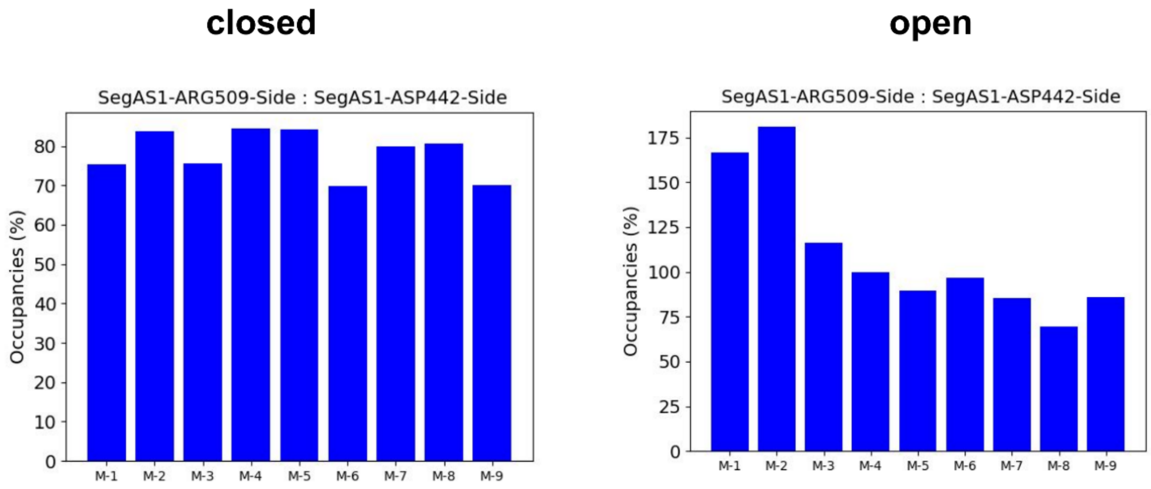

Fig. S2. Common stable inner hydrogen bonds found in the RBD domain of both closed and open states along all the macroiterative simulations.

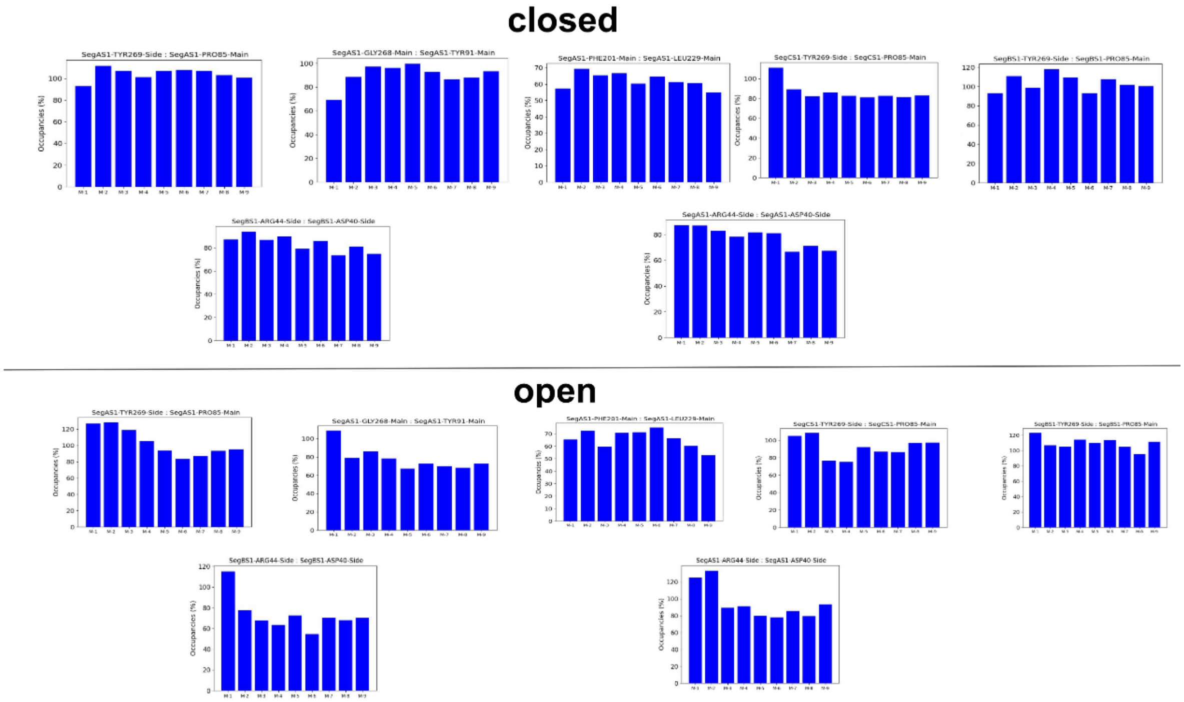

Fig. S3. Common stable inner hydrogen bonds found in the NTD domain of both closed and open states along all the macroiterative simulations.

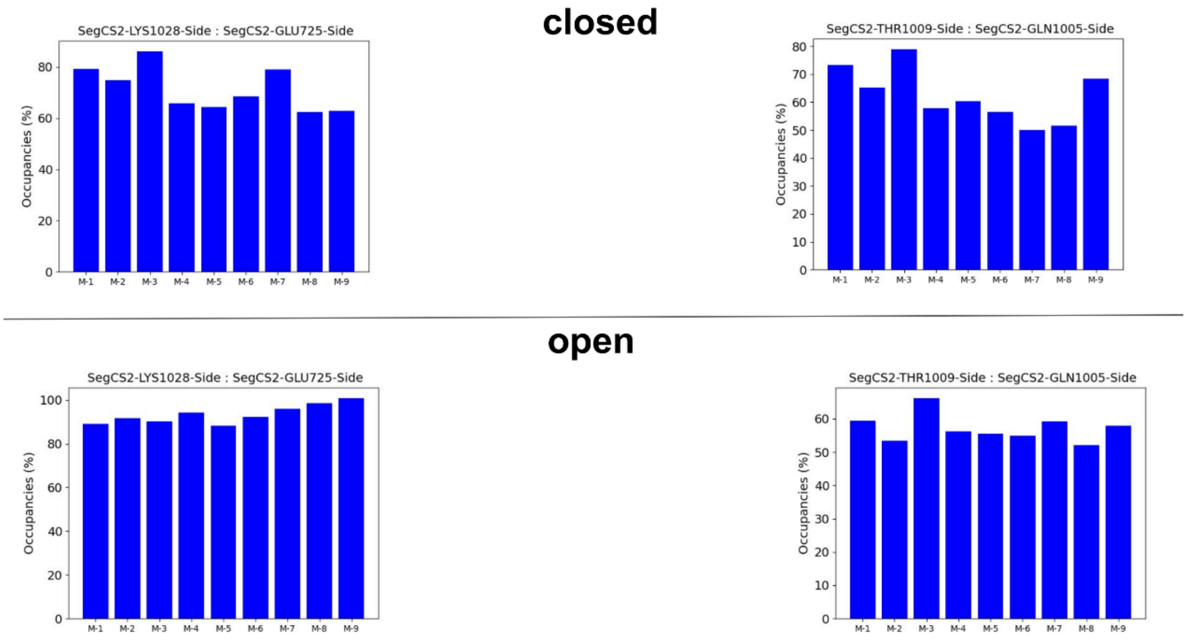

Fig. S4. Common stable inner hydrogen bonds found in the CH domain of both closed and open states along all the macroiterative simulations.

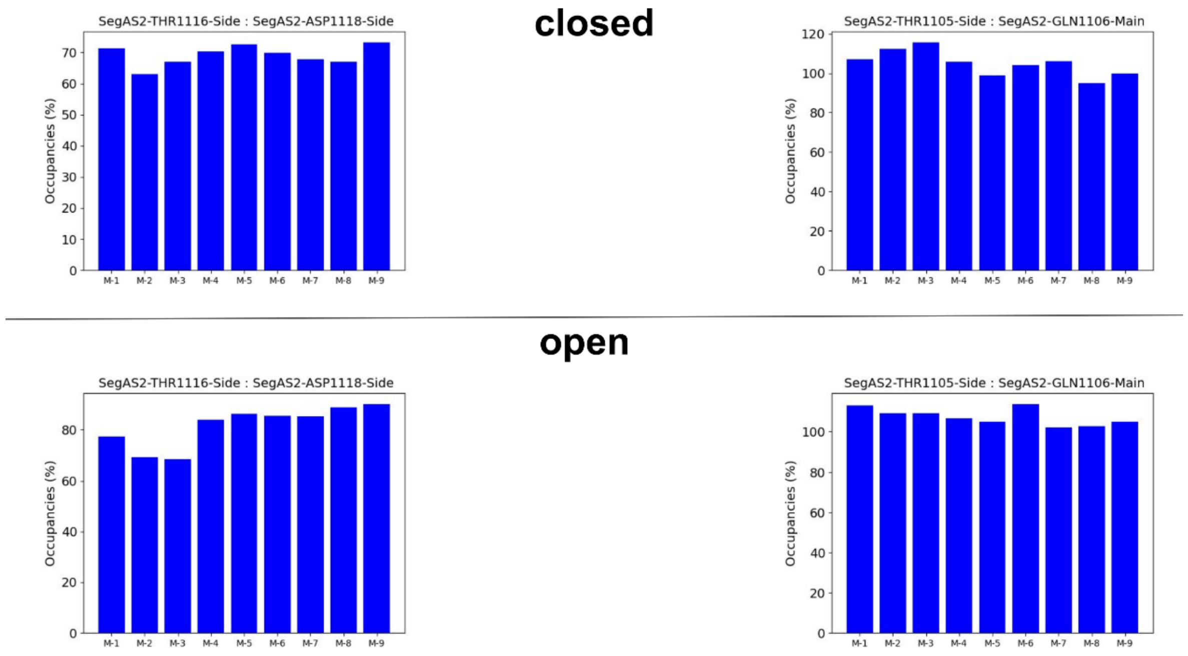

Fig. S5. Common stable inner hydrogen bonds found in the CD domain of both closed and open states along all the macroiterative simulations.

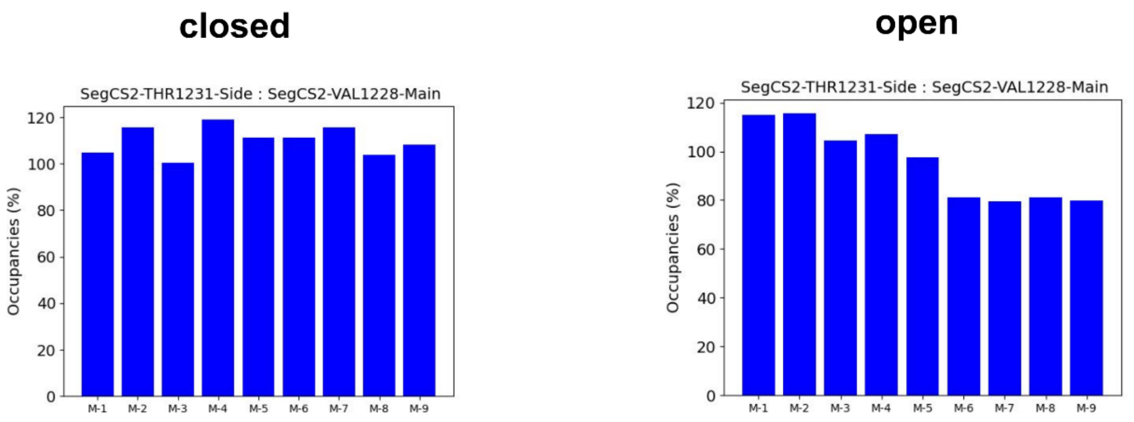

Fig. S6. Common stable inner hydrogen bonds found in the TM domain of both closed and open states along all the macroiterative simulations.

Solvent-Accessible Surface Area

The solvent-accessible surface area (SASA) of glycans was computed to investigate their solvent exposure throughout the simulation trajectory. The calculation was performed within the VMD software environment[8]. Initially, a list of glycan segment names was defined to facilitate the selection process. The total number of frames in the trajectory was determined to iterate over each frame and over each glycan segment considering only its non-hydrogen atoms during the calculation. The SASA of the selected segment, excluding hydrogen atoms, was calculated using the **measure sasa** utility available in VMD with a probe radius of 1.4 Å. The SASA values obtained for each frame were accumulated to compute the total SASA for the segment. Subsequently, the average SASA for the segment across all frames was determined by dividing the total SASA by the number of frames.

Dynamic Cross-Correlation Function

The analysis of dynamic cross-correlations between protein residues and glycan segments was conducted using a combination of Python libraries, including NumPy [1], Matplotlib [3], SciPy [5], MDAnalysis [9,10], and Seaborn [11]. The protein topology and trajectory were processed using the MDAnalysis library within Python. The protein residues and glycan segments of interest were selected based on predefined residue numbers and segment names. The center of mass (COM) for both the selected protein residues and glycan segments was calculated for each frame of the trajectory. The COM values were stored in NumPy arrays for further analysis. Using the **correlate2d** function from the SciPy library, the dynamic cross-correlation matrix was computed between each pair of residues and glycan segments throughout the trajectory. To ensure consistent visualization, the dynamic cross-correlation matrix was normalized using a min-max normalization function. The intensity of each cell in the heatmap indicates the strength of the correlation between the corresponding residue and glycan segment.

Center-of-Mass Distances between Protein and Glycan Residues

The selection of protein domains and corresponding glycans was based on Table S1 in Casalino et al. [7]. To compute the distances between these selected residues, the trajectory was iterated over. Utilizing VMD **measure center** utility, the center-of-mass (COM) coordinates for each selected protein and glycan group were determined, followed by the computation of the distance between their respective COMs.

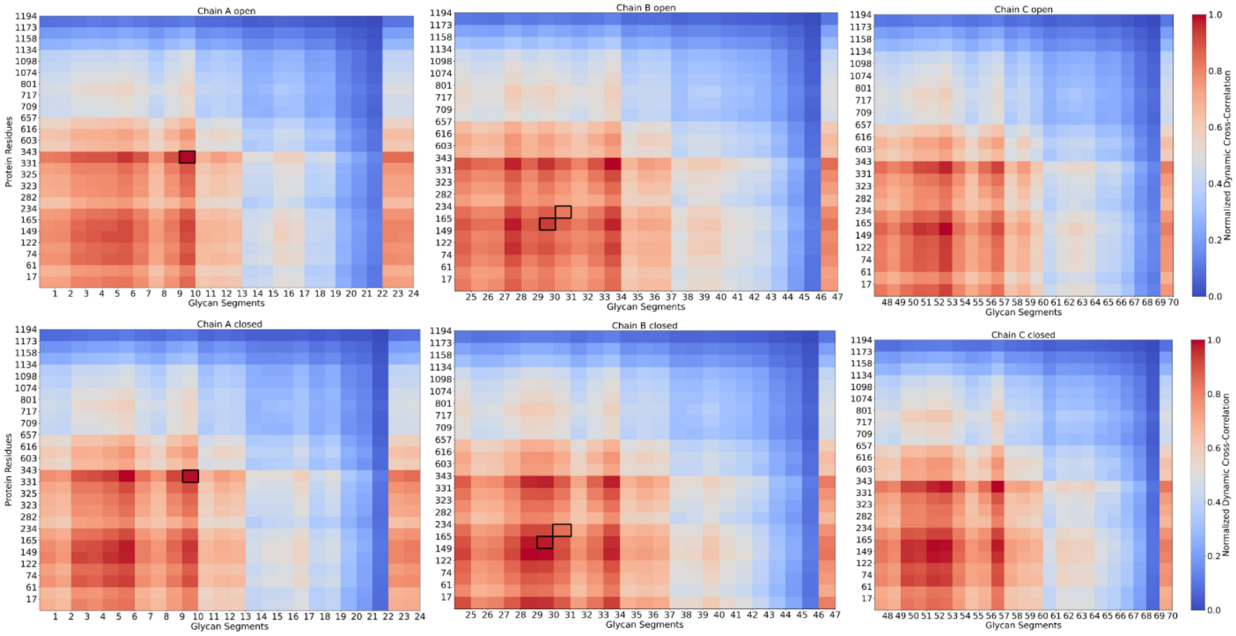

Fig. S7. Normalized dynamic cross-correlation function depicting the dynamic interactions between protein residues of chains A, B, C and surrounding glycan segments across open (top) and closed (bottom) states of the SARS-CoV-2 viral structure. The color intensity represents the strength of correlation, ranging from 0 (low correlation, shown in blue) to 1 (high correlation, shown in red). The residues responsible for the glycan gating are contoured with rectangle black boxes.

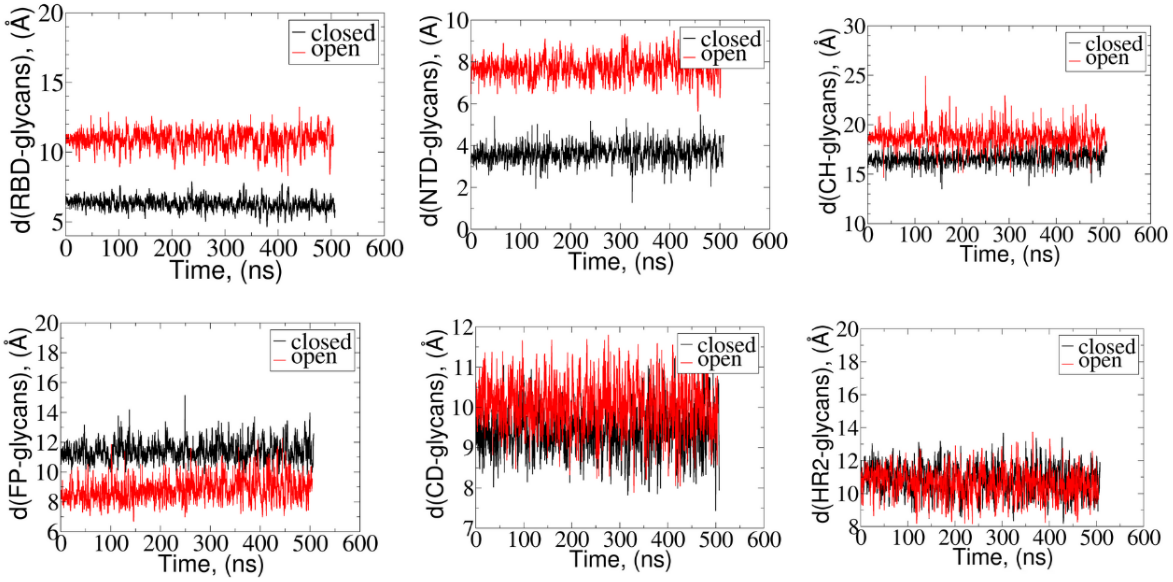

Fig. S8. Center-of-mass (COM)-to-COM distances between protein domains and surrounded glycans along combined simulations from all iterations for open (red) and closed (black) states.

Contact Maps

The contact maps analysis involved computing the distances between specific protein residues and glycan residues from the trajectories. Firstly, atoms corresponding to the target protein and glycan residues were selected from the trajectory data. Next, the center of mass (COM) coordinates were calculated for each protein residue and glycan residue across all frames of the trajectory. This step allowed us to represent each residue as a single point, simplifying the analysis of their spatial relationships. Subsequently, the Euclidean distance between the COM of each protein residue and the COM of each glycan residue was computed for every frame of the trajectory, using the NumPy **linalg.norm** and SciPy **spatial.distance.cdist** functions. This process resulted in a matrix where each row represented the protein residue and each column represented a glycan residue, with the values indicating the distances between corresponding protein-glycan residue pairs. Finally, the distance matrix was visualized as a heatmap, with color intensity reflecting the distance between protein-glycan pairs.

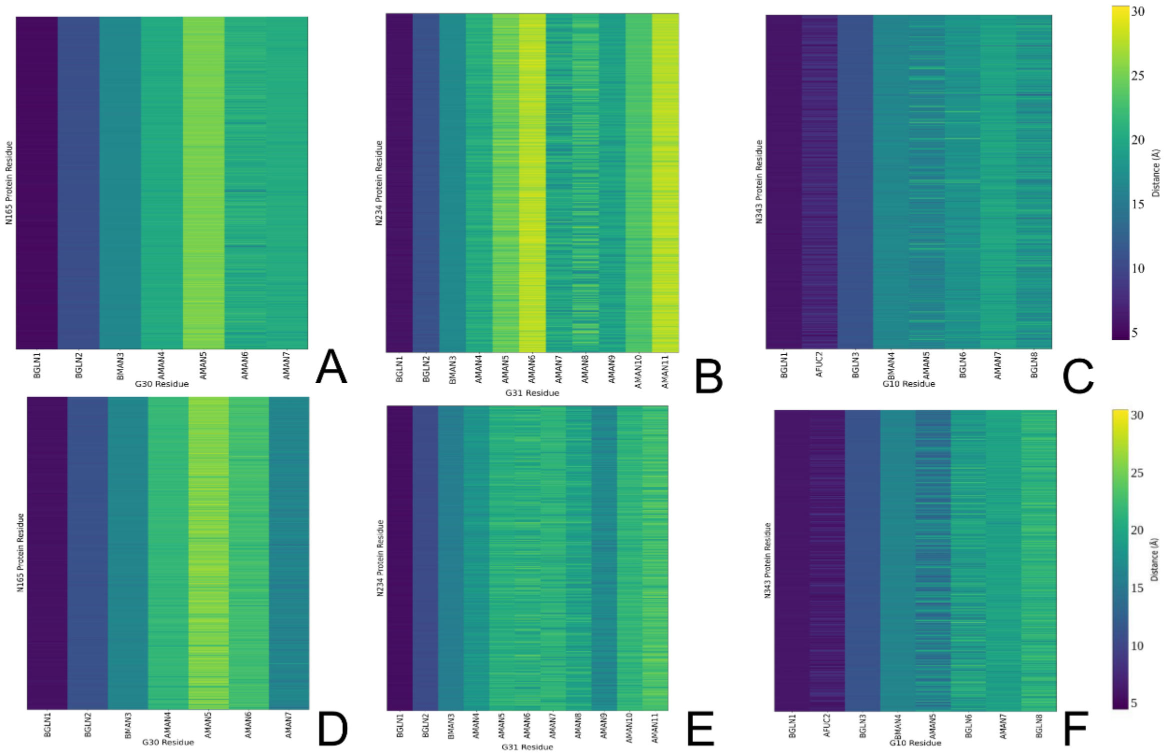

Fig. S9. Contact maps of interactions between center-of-mass (COM) of N165, N234, and N343 of the protein and corresponding glycans G30, G31, and G10 in open (A, B, C) and closed (D, E, F) states. The corresponding COM of glycan residues are BMAN (β-D-mannose), AMAN (α-D-mannose), BGAL (β-D-galactose), BGLN (β-D-glucoseamine), ANE5 (α-D-neuraminic acid (also known as sialic acid)), AGAN (α-D-galactose), AFUC (α-L-fucose).

Radial Distance Analysis of Glycans

To investigate the radial distribution of the glycan shield, we calculated the radial distances of glycan COM relative to the z-axis across all frames of the simulation. For each frame, we selected all glycan residues across specified segments (G1 to G70), excluding hydrogen atoms. The COM of the selected glycan residues was then computed within VMD **measure center** utility. The radial distance of the glycan COM from the z-axis was determined using the formula $\sqrt{x^2 + y^2}$, where x and y are the coordinates of the COM in the xy-plane.

Radial Distribution Analysis. Coordination Number. Pair Distribution Analysis

We leveraged Radial Distribution Function (RDF) analysis to unravel the intricate distribution patterns of interacting oxygen atoms across diverse molecular interactions, notably within protein-water and glycan-water interfaces. Similar to SASA, the RDF analysis was performed within the VMD software environment[8,12]. A bin size of 0.1 Å and a total radial distance of 10 Å were used. The selection of oxygen atoms from water, glycan, and protein was customized based on the specific aims of our investigation discussed in the main text. Besides, to evaluate the number of water molecules in the proximity to the selected protein and glycan residues, we meticulously evaluated coordination numbers through the integration of RDFs, providing nuanced insights into the degree of atomic coordination within the molecular system under scrutiny. Additionally, we conducted localized analyses akin to RDF, employing pair distribution function (PDF) calculations to delve deeper into the distribution profiles of specific atom pairs within the same tool.

Polarizable Water Indication

In the initial stage, we compiled lists of water molecules exhibiting high dipole moments (> 2.8 D) for each frame in every MD step of open-state simulations. Through careful observation, we then visually (i.e., by means of VMD software[8]) identified regions where these molecules clustered most frequently, particularly focusing on their proximity to specific protein residues. Subsequently, we established a threshold of 10 Å to investigate changes in solvent dynamics near the associated protein residues along all the accumulated trajectories. For each water molecule proximity to the target protein and glycan residues within each micro-iteration, we calculated the average dipole moment, average distance from the protein residue, and occupancy percentage throughout the simulation. The average dipole moment was computed by summing the dipole moments of all water molecules within the defined region and dividing them by the total count of water molecules. Similarly, the average distance from the protein and glycan residues was determined by calculating the distance between each water molecule and the COM of the target residues. The occupancy percentage was calculated to quantify the extent to which water molecules occupied the defined

region around the selected residues (at a distance less than 5 Å). In addition, each occupancy percentage was weighted based on the weights obtained from the density PCA of our conformational sampling method, ensuring that the contribution of each occupancy value to the overall analysis was proportional to its significance in the conformational space. The weighted occupancy for each water molecule was calculated by dividing the occupancy percentage by the total weight obtained from the density PCA. The obtained data was further filtered within the criteria defined in the main text in the Results section, ensuring that only interactions meeting specific thresholds were considered for subsequent analysis.

Given the absence of water molecules with high dipole moments (i.e., exceeding 2.8 D) in the closed-state simulations, we opted to analyze the average dipole moments of protein and glycan atoms known to undergo the previously described phases in the open configuration. Additionally, we calculated the average dipole moments of oxygen atoms of water molecules proximal to these residues to glean insights into their polarizability dynamics and compared them to those of the open state, thus offering a comprehensive understanding of solvent-protein interactions across various simulation conditions.

Dipole Moment Distribution Analysis

The dipole moment distribution analysis involved extracting dipole moment data from simulation output files and visualizing their distribution. Firstly, all relevant data files containing dipole moment information were located within the specified directory corresponding to each of the defined phases of water interaction. Next, the dipole moments were extracted from each file. The average dipole moment and its standard deviation were calculated across all frames of the simulation trajectories. Subsequently, a histogram with a kernel density estimate was plotted using the Seaborn library[11]. This visualization provided an overview of the dipole moment distribution, with the x-axis representing the dipole moment values and the y-axis representing probability density. Additionally, vertical lines indicating the mean, median, and mode of the dipole moments were overlaid on the histogram, offering further insights into the distribution characteristics.

N343RBD-A open state

Bridging

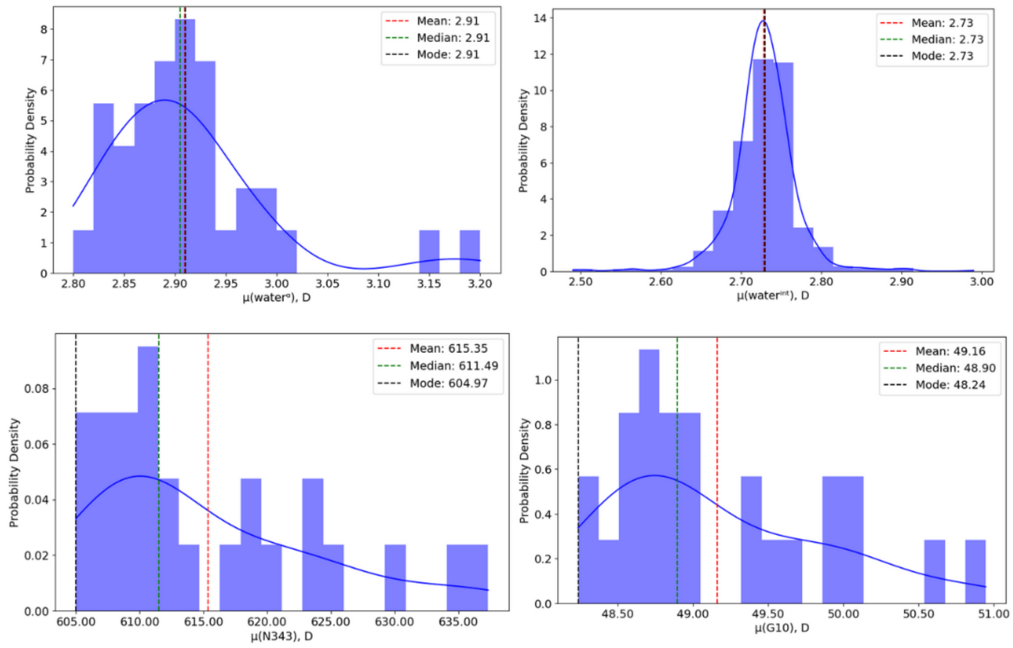

$N343_{RBD-A}$ open state

Clustering

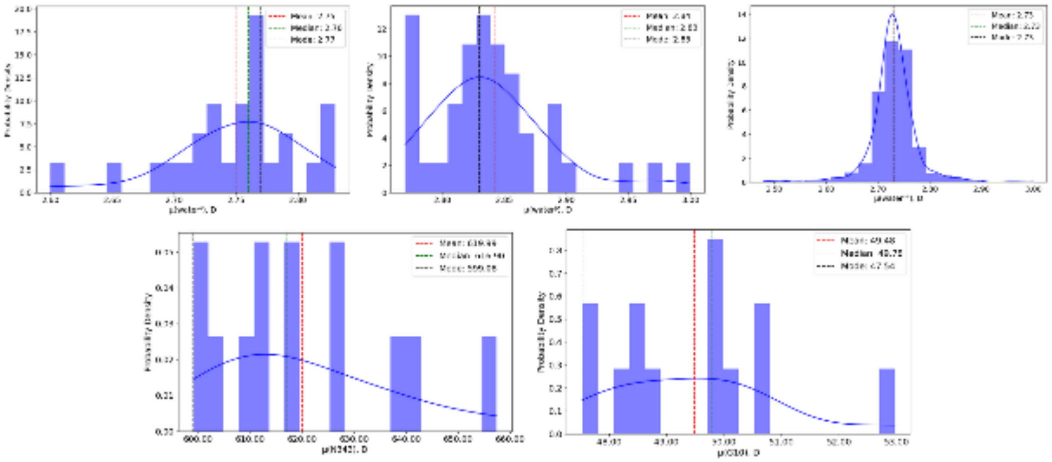

$N343_{RBD-A}$ open state

Replacement

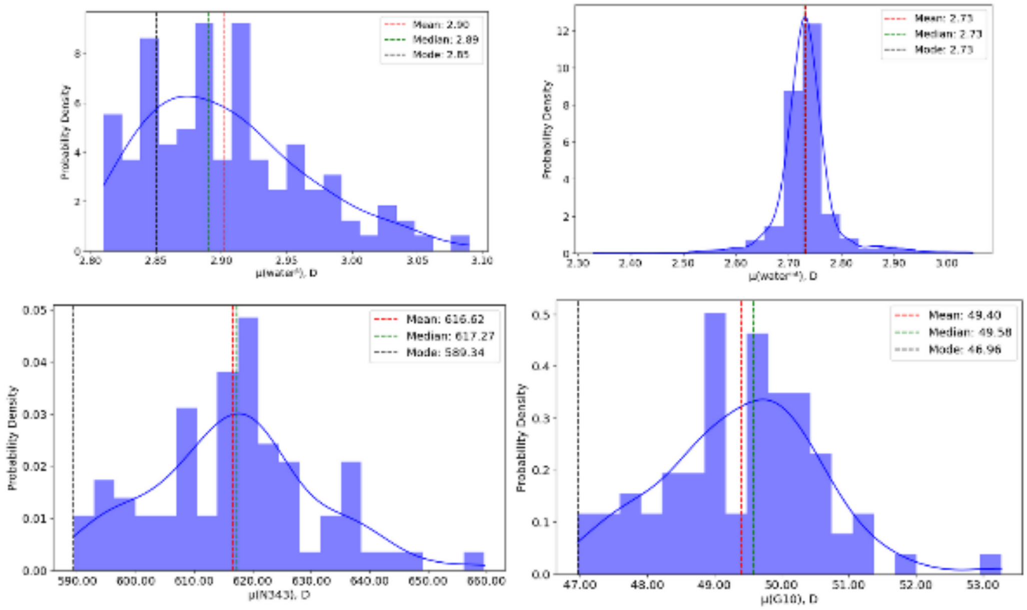

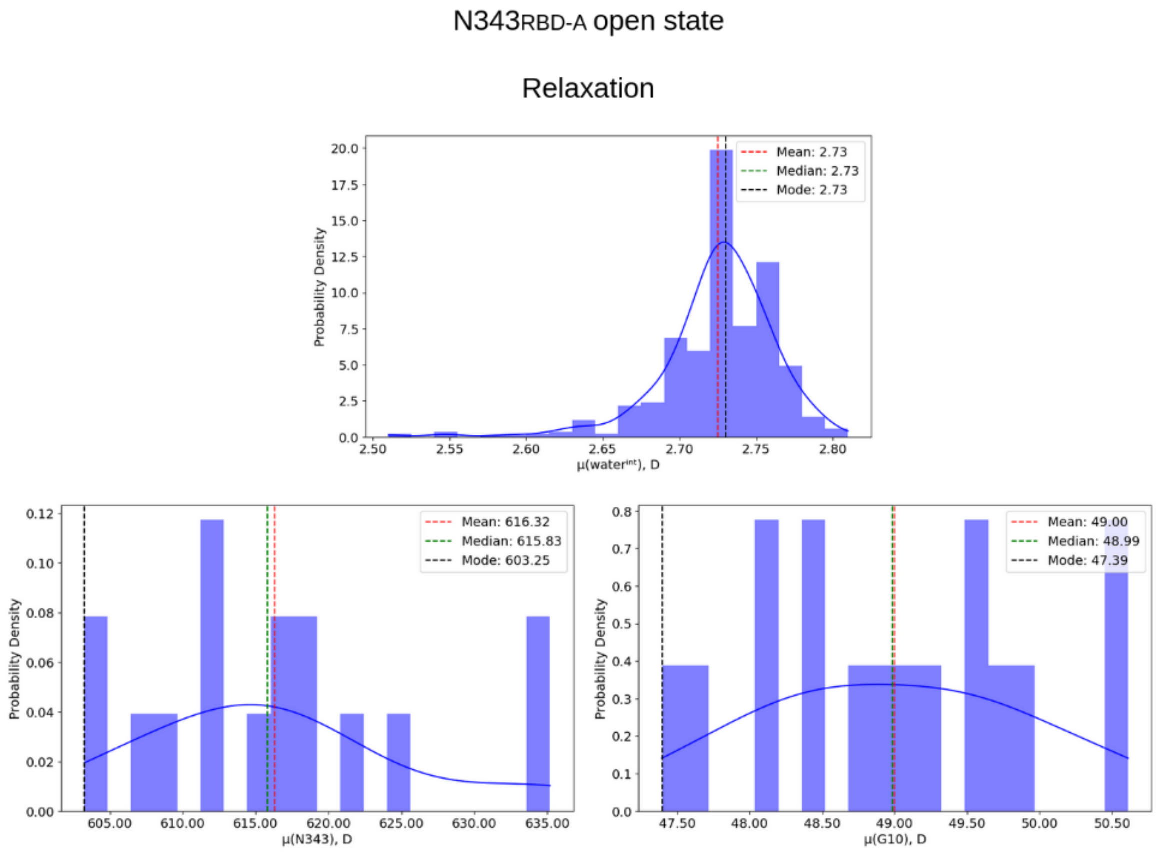

Fig. S10. Distribution of dipole moments along each phase for the N343$_{RBD-A}$ interaction pattern in the open state, including mode, mean, median values.

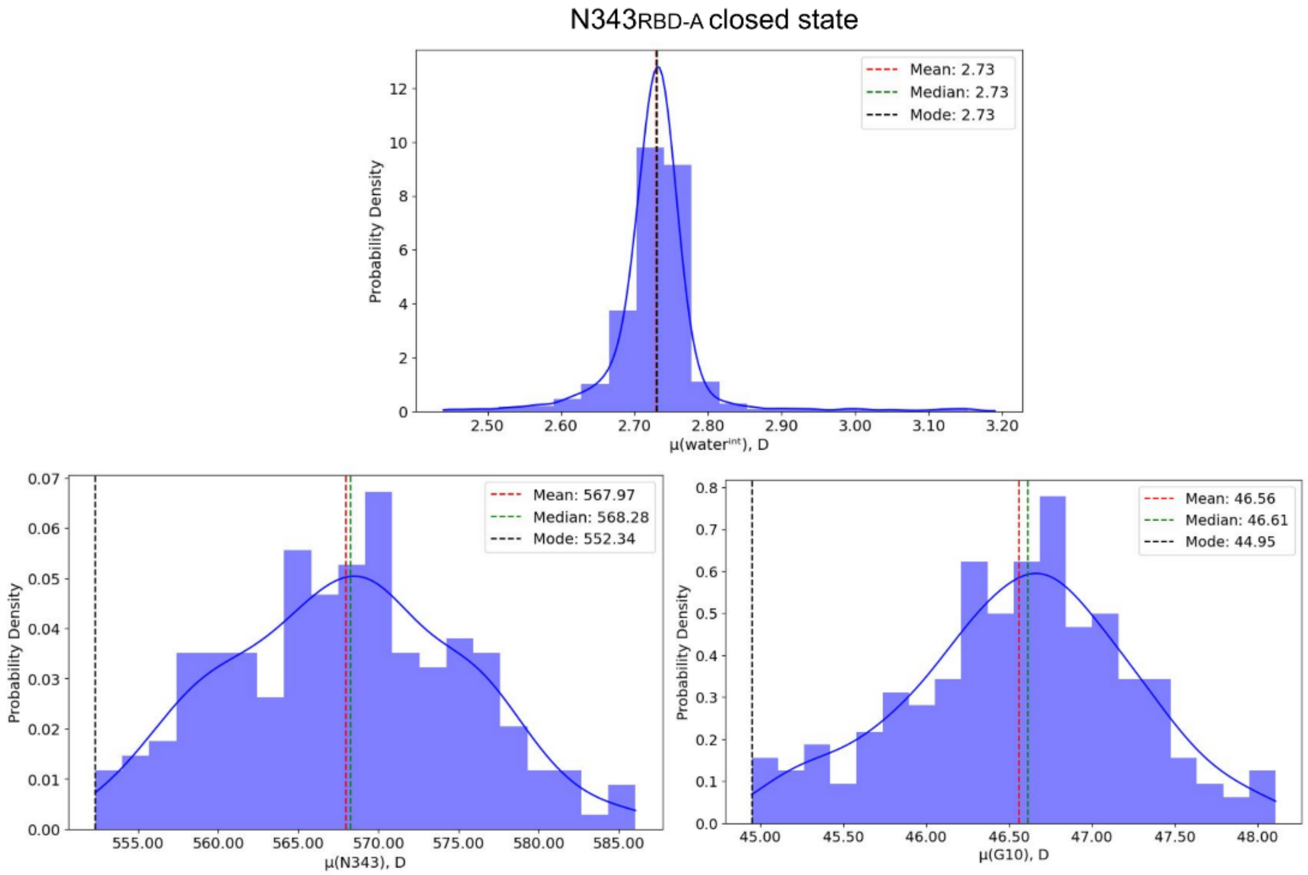

Fig. S11. Distribution of dipole moments along each phase for the $N343_{RBD\text{-}A}$ interaction pattern in the closed state, including mode, mean, median values.

N165NTD-B open state

Bridging

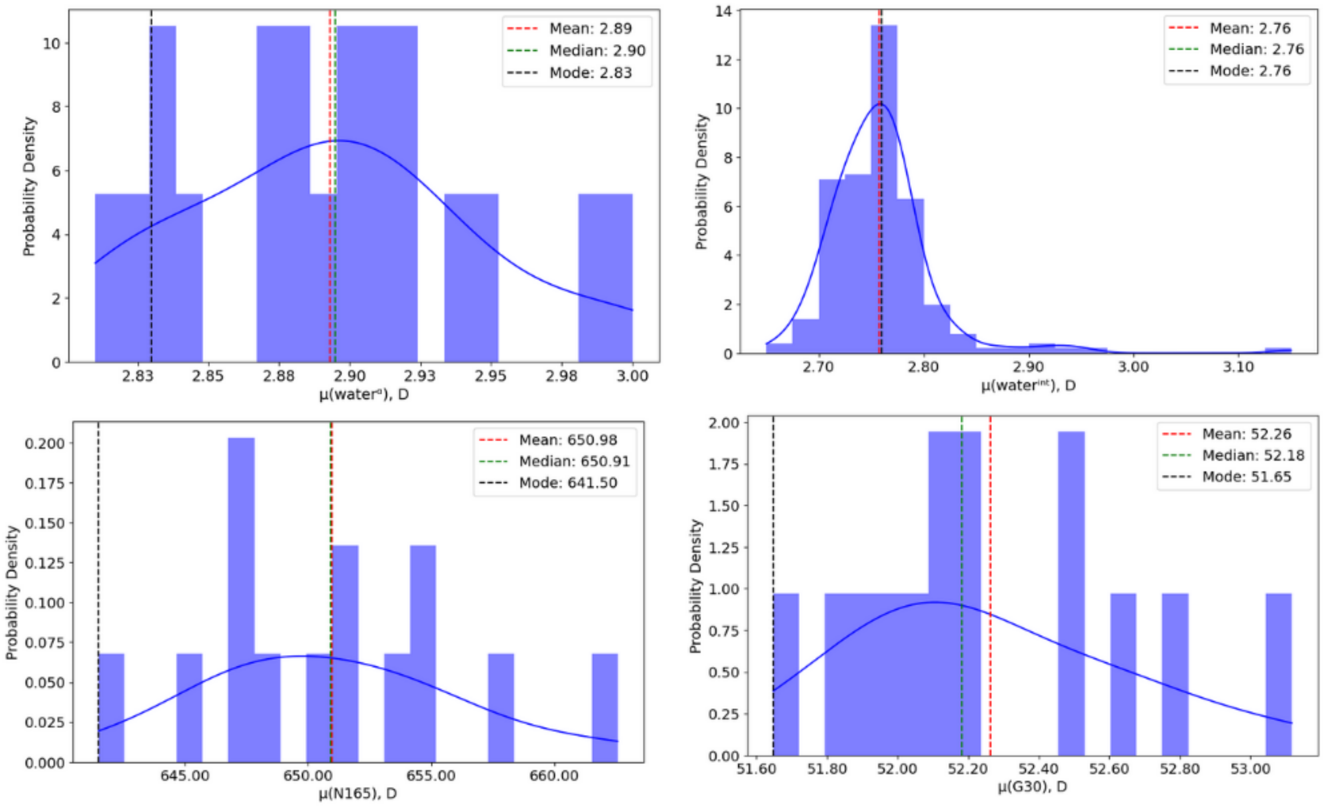

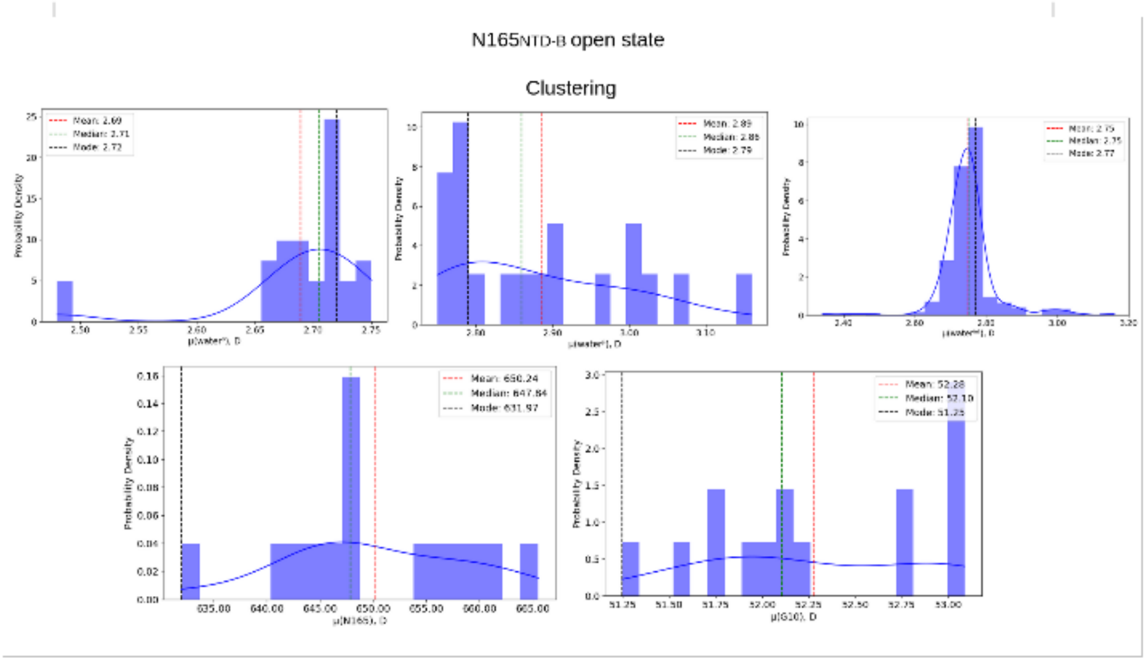
N165NTD-B open state
Clustering

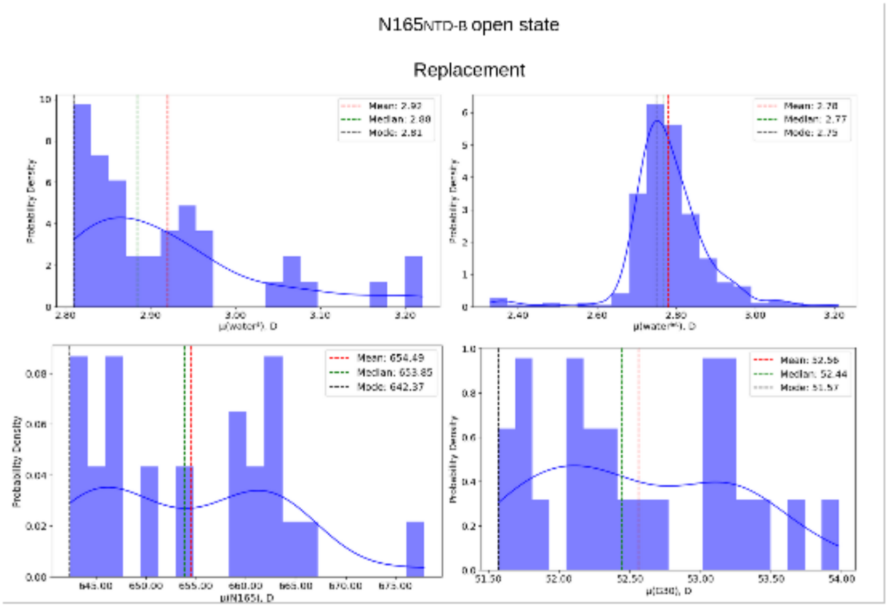
N165NTD-B open state
Replacement

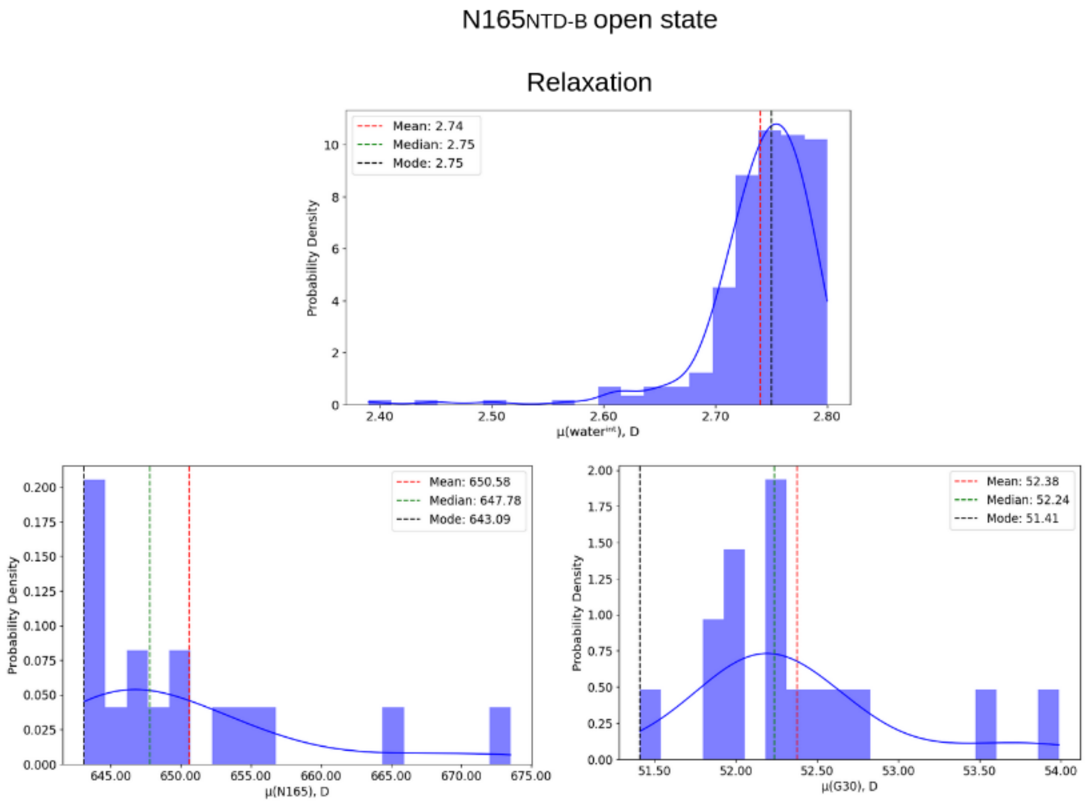

Fig. S12. Distribution of dipole moments along each phase for the N165$_{NTD-B}$ interaction pattern in the open state, including mode, mean, median values.

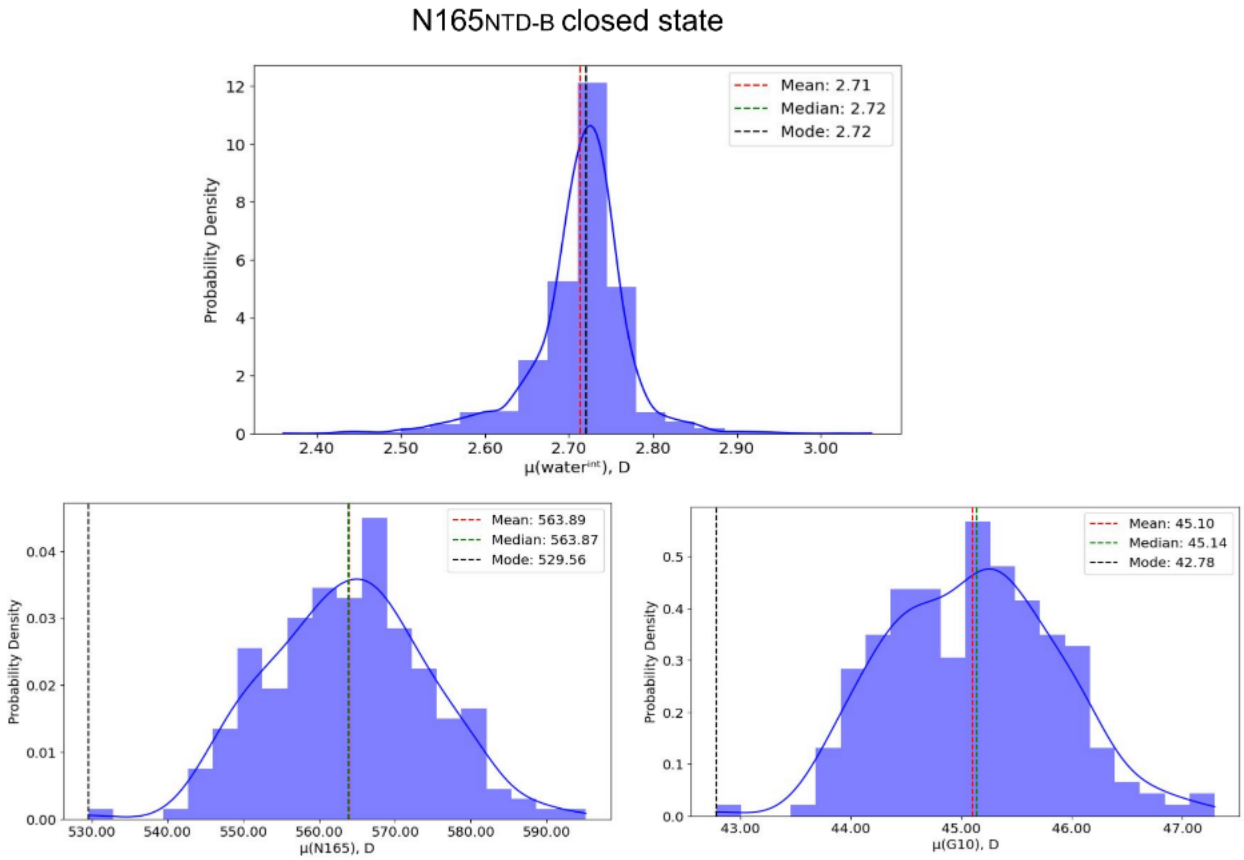

Fig. S13. Distribution of dipole moments along each phase for the $N165_{NTD\text{-}B}$ interaction pattern in the closed state, including mode, mean, median values.

Parametrization Details for Glycans and Lipid Molecules

AMOEBA parameters for the glycan molecules, including BMAN (beta-D-mannose); AMAN (alpha-D-mannose), BGAL (beta-D-galactose), BGLN (beta-N-acetyl-D-glucosamine), ANE5 (N-acetyl-alpha-neuraminic acid), AGAN (N-acetyl-alpha-D-galactosamine), AFUC (alpha-L-fucose), and lipid molecules, including POPC (1-palmitoyl-2-oleoyl-sn-glycero-3-phosphocholine), POPI (1-palmitoyl-2-oleoyl-sn-glycero-3-phosphoinositol), POPS (1-palmitoyl-2-oleoyl-sn-glycero -3-phosphatidylserine), and cholesterol were derived using the Poltype2 automation tool discussed in the main text. For POPE (1-palmitoyl-2-oleoyl-phosphatidyl ethanolamine), the parameters were taken from reference 13 as well as the parameters in common for all lipids oleoyl motifs. The sn-glycero-3-phosphocholine (in POPC), sn-glycero-3-phosphoinositol (in POPI), and sn-glycero-3-phosphatidylserine (in POPS) parts and cholesterol were parametrized separately. Each input glycan residue and individual non-oleoyl lipid fragments (in sdf format) was first optimized at MP2/6-31G* level of theory. Optimized geometry then was used for two single-point calculations at MP2/6-311G** (low-level) and MP2/aug-cc-pvtz (high-level) of theory, respectively. The electron density of the low-level calculations was used to derive the atomic multipoles, by employing the distributed multipole analysis method in GDMA program (available in open-source library https://gitlab.com/anthonyjs/gdma) [14]. These multipoles were further optimized by fitting to the electrostatic potential (ESP) generated by the high-level electron density. The valence and torsion parameters were matched to the existing database in Poltype2 by using SMARTS patterns (see https://www.daylight.com/dayhtml/doc/theory/theory.smarts.html) [15]. For the remaining torsion parameters that were not found in SMARTS database, the dihedral angle around the rotatable bond was spined over 360 degrees at interval of 30 degree (12 datapoints for each dihedral angle). For the torsional angle in the glycan rings, 5 datapoints (perturbed from the original angle value by -20, -10, 0, +10, +20 degrees). The geometry of each structure at a certain dihedral value was then optimized at PBE1PBE/6-31G level of theory by restraining the torsion angles of interest and followed by single point energy calculations at MP2/6-31+G* level of theory. The relative energy from the QM calculations then were targeted to fit the torsional parameters. All the QM

calculations were performed using Gaussian 09 package[16]. The poledit.x executable within Tinker software was used to convert the multipole values from GDMA output (in global frame) to Tinker format (in its local frame definition). The potential.x executable in Tinker was used to refine the multipoles by fitting to high-level ESP generated by QM. Parameter files and all the torsion fit plots have been included in the Supplementary Material with several exceptions: (1) ANE5 since all the torsional parameters have been matched from database and for AGAN the acetyl-amine fragment along with galactose core were merged from BGAL and BGLN parametrization outputs and (2) POPE parameters. Additionally, we included the final parameter file and the corresponding Tinker **xyz** structure files for both closed and open states.

References

(1) Harris, C. R.; Millman, K. J.; van der Walt, S. J.; Gommers, R.; Virtanen, P.; Cournapeau, D.; Wieser, E.; Taylor, J.; Berg, S.; Smith, N. J. et al. Array programming with NumPy. *Nature* **2020**, *585*, 357–362.

(2) McGibbon, R. T.; Beauchamp, K. A.; Harrigan, M. P.; Klein, C.; Swails, J. M.; Hernández, C. X.; Schwantes, C. R.; Wang, L.-P.; Lane, T. J.; Pande, V. S. MDTraj: A Modern Open Library for the Analysis of Molecular Dynamics Trajectories. *Biophys. J.* **2015**, *109*, 1528 – 1532.

(3) Hunter, J. D. Matplotlib: A 2D graphics environment. *Comput. Sci. Eng.* **2007**, *9*, 90–95.

(4) Pedregosa, F.; Varoquaux, G.; Gramfort, A.; Michel, V.; Thirion, B.; Grisel, O.; Blondel, M.; Prettenhofer, P.; Weiss, R.; Dubourg, V. et al. Scikit-learn: Machine Learning in Python. *J. Mach. Learn. Res.* **2011**, *12*, 2825–2830.

(5) Virtanen, P.; Gommers, R.; Oliphant, T. E.; Haberland, M.; Reddy, T.; Cournapeau, D.; Burovski, E.; Peterson, P.; Weckesser, W.; Bright, J. et al. SciPy 1.0: Fundamental Algorithms for Scientific Computing in Python. *Nat. Methods* **2020**, *17*, 261–272.

(6) Amaro, R. E. Amaro Lab - COVID-19. https://amarolab.ucsd.edu/covid19.php.

(7) Casalino, L.; Gaieb, Z.; Goldsmith, J. A.; Hjorth, C. K.; Dommer, A. C.; Harbison, A. M.; Fogarty, C. A.; Barros, E. P.; Taylor, B. C.; McLellan, J. S. et al. Beyond Shielding: The Roles of Glycans in the SARS-CoV-2 Spike Protein. *ACS Cent. Sci.* **2020**, *6*, 1722–1734.

(8) Humphrey, W.; Dalke, A.; Schulten, K. VMD – Visual Molecular Dynamics. *J. Mol. Graph.* **1996**, *14*, 33–38.

(9) Gowers, R. J.; Linke, M.; Barnoud, J.; Reddy, T. J. E.; Melo, M. N.; Seyler, S. L.; Dotson, D. L.; Domanski, J.; Buchoux, S.; Kenney, I. M. et al. MDAnalysis: A Python package for the rapid analysis of molecular dynamics simulations. Proc. Python Sci. Conf. 2016; pp 98–105.

(10) Michaud-Agrawal, N.; Denning, E. J.; Woolf, T. B.; Beckstein, O. MDAnalysis: A Toolkit for the Analysis of Molecular Dynamics Simulations. *J. Comput. Chem.* **2011**, *32*, 2319–2327.

(11) Waskom, M. L. seaborn: statistical data visualization. *J. Open Source Softw.* **2021**, *6*, 3021.

(12) Levine, B. G.; Stone, J. E.; Kohlmeyer, A. Fast analysis of molecular dynamics trajectories with graphics processing units—Radial distribution function histogramming. *J. Comput. Phys.* **2011**, *230*, 3556–3569.

(13) Chu, H.; others Polarizable atomic multipole-based force field for DOPC and POPE membrane lipids. *Mol. Phys.* **2018**, *116*, 1037–1050.

(14) Stone, A. J. Distributed Multipole Analysis: Stability for Large Basis Sets. *J. Chem. Theory Comput.* **2005**, *1*, 1128–1132.

(15) Ehrlich, H.-C.; Rarey, M. Systematic benchmark of substructure search in molecular graphs - From Ullmann to VF2. *J. Cheminform.* **2012**, *4*, 13.

(16) Frisch, M. J.; Trucks, G. W.; Schlegel, H. B.; Scuseria, G. E.; Robb, M. A.; Cheeseman, J. R.; Scalmani, G.; Barone, V.; Petersson, G. A.; Nakatsuji, H. et al. Gaussian. Gaussian, Inc.: Wallingford, CT, USA, 2009.

Movie_S1-CS.MOV
This file cannot be rendered in this PDF. Please download the source file.

Data_S1-CS.zip
This file cannot be rendered in this PDF. Please download the source file.

Data_S2-CS.zip
This file cannot be rendered in this PDF. Please download the source file.

Data_S3-CS.zip
This file cannot be rendered in this PDF. Please download the source file.

Data_S4-CS.zip
This file cannot be rendered in this PDF. Please download the source file.
